# Habit Expression and Disruption as a Function of Attention-Deficit/Hyperactivity Disorder Symptomology

**DOI:** 10.3389/fpsyg.2019.01997

**Published:** 2019-09-03

**Authors:** Ahmet O. Ceceli, Giavanna Esposito, Elizabeth Tricomi

**Affiliations:** ^1^Department of Psychology, Rutgers University-Newark, Newark, NJ, United States; ^2^New Jersey Institute of Technology, Newark, NJ, United States

**Keywords:** ADHD, reward, habit, goal-directed, motivation, control

## Abstract

Attention-deficit/hyperactivity disorder (ADHD) is associated with neurobehavioral reward system dysfunctions that pose debilitating impairments in adaptive decision-making. A candidate mechanism for such anomalies in ADHD may be a compromise in the control of motivated behaviors. Thus, demonstrating and restoring potential motivational control irregularities may serve significant clinical benefit. The motivational control of action guides goal-directed behaviors that are driven by outcome value, and habits that are inflexibly cue-triggered. We examined whether ADHD symptomology within the general population is linked to habitual control, and whether a motivation-based manipulation can break well-learned habits. We obtained symptom severity scores from 106 participants and administered a Go/NoGo task that capitalizes on familiar, well-learned associations (green-Go and red-NoGo) to demonstrate outcome-insensitivity when compared to newly learned Go/NoGo associations. We tested for outcome-insensitive habits by changing the Go and NoGo contingencies, such that Go signals became NoGo signals and vice versa. We found that generally, participants responded less accurately when green and red stimuli were mapped to color-response contingencies that were incongruent with daily experiences, whereas novel Go/NoGo stimuli evoked similar accuracy regardless of color-response mappings. Thus, our Go/NoGo task successfully elicited outcome-insensitive habits (i.e., persistent responses to familiar stimuli without regard for consequences); however, this effect was independent of ADHD symptomology. Nevertheless, we found an association between hyperactivity and congruent Go response latency, suggesting heightened pre-potency to perform habitual Go actions as hyperactivity increases. To examine habit disruption, participants returned to the lab and underwent the familiar version of the Go/NoGo task, but were given mid-experiment performance tracking information and a monetary incentive prior to contingency change. We found that this motivational boost via dual feedback prevented the incongruency-related accuracy impairment, effectively breaking the habit, albeit independent of ADHD symptomology. Our findings present only a modest link between ADHD symptomology and motivational control, which may be due to compensatory mechanisms in ADHD driving goal-directed control, or our task’s potential insensitivity to individual differences in ADHD symptomology. Further investigations may be crucial for determining whether ADHD is related to motivational impairments.

## Introduction

Individuals with attention deficit-hyperactivity disorder (ADHD) are known to exhibit cognitive impairments that span domains of attention and impulsivity (American Psychiatric Association, 2013). These hallmark symptoms are often accompanied by executive control irregularities, such as diminished inhibitory control and excessive distractibility that interfere with daily functioning (Willcutt et al., 2005). Additionally, behavioral and neurobiological reports have highlighted reward-related abnormalities in ADHD, in that individuals with ADHD display impairments in learning from, interacting with, and processing rewards (Ceceli et al., 2019). Children and adults with ADHD present heightened delay aversion, such that they choose immediate, less valuable rewards over delayed yet larger rewards (Sonuga-Barke et al., 1992; Kessler et al., 2005b; Antrop et al., 2006; Marx et al., 2013). In addition to such examples of suboptimal decision-making, individuals with ADHD also exhibit abnormal reward-related neural processing in the brain’s reward circuitry, such as decreased signaling in the ventral striatum during reward anticipation, and atypical orbitofrontal cortex (OFC) activity during reward delivery (Ströhle et al., 2008; Wilbertz et al., 2012; Furukawa et al., 2014; Plichta and Scheres, 2014; von Rhein et al., 2015). The affected regions of the brain that regulate reward anticipation and processing (i.e., the striatum and prefrontal cortex), are also known as integral areas for executing motivated behaviors (Balleine and O’Doherty, 2009; O’Doherty, 2016). These neurobehavioral dysfunctions in ADHD, when taken together with the cardinal presentations of inattention and impulsivity, suggest potential disparities in the control of motivated behaviors that have yet to be elucidated.

The motivational account of behavioral control posits that our actions can be either goal-directed, as in, performed deliberately in pursuit of a desirable outcome, or habitual, as in, triggered in response to a salient cue regardless of outcome value (Dickinson and Balleine, 1994). These components of motivational control have distinct neural signatures, such that the prefrontal cortex and caudate are known to be imperative for the execution of goal-directed behaviors, while cue-based habitual control is largely associated with the putamen and motor cortex (Haber, 2003; O’Doherty et al., 2004; Tricomi et al., 2009). Interestingly, a compelling body of work documents functional and structural abnormalities in ADHD when compared to neurotypicals (NTs) in these brain regions, suggesting a compromised corticostriatal system that could be indicative of motivational control deficits. For example, ADHD is associated with reduced gray matter volume in the caudate, expansion of the posterior putamen, and aberrant connectivity in the ventromedial prefrontal cortex (vmPFC) and anterior cingulate cortex (ACC) (Qiu et al., 2009; Frodl and Skokauskas, 2012; Costa Dias et al., 2013; Norman et al., 2016; von Rhein et al., 2017; Rosch et al., 2018). Studies in rodents have suggested that a rat model of ADHD, the spontaneously hypertensive rat, exhibits a habit-dominated motivational control system, in that these rats that possess ADHD-like symptoms also display outcome-insensitive behavioral patterns (i.e., pressing a lever that predicts a food outcome to which the rat is sated) (Natsheh and Shiflett, 2015). Neural evidence suggests that this behavioral deficit is linked to imbalances in dopamine receptor activation, supporting the idea that abnormalities in the striatal systems may also manifest as an over-reliance on habitual control in ADHD (Natsheh and Shiflett, 2018).

If ADHD is indeed associated with enhanced habitual control that favors outcome-insensitive behaviors, the next logical and translationally valuable step would be to identify strategies that can overcome this behavioral deficit. For instance, performance-contingent feedback is a frequently employed tool that has been shown to improve behavioral output (Montague and Webber, 1965; Kluger and DeNisi, 1996). The positive effects of feedback in the form of performance-tracking information, as well as primary and secondary incentives, have been well-documented in the cognitive flexibility domain – namely using task-switching paradigms. Indeed, even the promise of a future performance-contingent reward has been shown to amplify task-switching performance (Yee et al., 2016). Importantly, performance-contingent monetary feedback is associated with the engagement of top-down control of task-switching processes (Umemoto and Holroyd, 2015). Taken together, we believe that the benefits of feedback on behavioral output and control over actions may carry over to the restoration of goal-directed behaviors in ADHD. Specifically, we reason that amplifying the salience of the outcomes of one’s behaviors with feedback (e.g., tying task performance to monetary incentives and performance tracking) may reactivate goal-representations in otherwise stimulus-driven associations. In support of this hypothesis, we have previously demonstrated the beneficial effects of feedback on the motivational control of action (Ceceli et al., 2019).

Tackling the expression of habits and the restoration of goal-directed behaviors in potentially compromised populations may involve overcoming the methodological limitations of the traditional habit paradigm. A meaningful assessment of habit expression and disruption may require access to rigid habits with a strong association between the triggering stimulus and the behavioral response. Therefore, instead of relying on labile, newly learned habits that have been the subject of inquiry in most investigations of motivational control (Ceceli and Tricomi, 2018), it may be more effective to study habit expression and disruption via well-learned, existing S–R associations that do not require extensive training in the laboratory (Ceceli et al., 2019).

To this end, we developed a Go/NoGo task that capitalizes on familiar green and red traffic light stimuli that activate existing stimulus–response associations (Ceceli et al., 2019). If green-Go and red-NoGo associations are habit-driven, an incongruent Go/NoGo mapping (green-NoGo, red-Go) should produce significant decrements in accuracy. Importantly, Go/NoGo mappings that involve novel stimuli with no significant behavioral representations (i.e., blue and purple light stimuli) should evoke no mapping-related performance impairments. If ADHD is associated with heightened habitual control, symptom severity might track the mapping-related impairments elicited by the familiar Go/NoGo stimuli (e.g., higher symptom severity scores should predict heightened errors of commission – response execution when instructed to withhold). Furthermore, if performance and monetary feedback are effective in restoring goal-directed control, this dual feedback delivery should protect against the mapping-related accuracy impairment, preventing the increase in commission errors when Go and NoGo associations are incongruent with daily experiences. Similarly, such a disruption in habits may also be correlated to ADHD symptom severity, such that a more severe presentation of ADHD symptoms may be less affected by the beneficial effects of feedback. Alternatively, if feedback is a salient enough motivator, highly symptomatic individuals may also benefit from our feedback manipulation, resulting in habit disruption across the board. To reveal whether ADHD is associated with habitual control, and whether a habit-dominated motivational control system may be remediated, we administered our well-learned habit task over the course of 2 days on a large sample from the general population, from whom we collected ADHD-related symptomology information. On the first day, we examined the execution of well-learned habits in our sample, and on the second day, we introduced our motivational enhancement manipulation – a combined delivery of performance information and monetary feedback – to restore goal-directed control. Importantly, per our pre-registered analysis plan (document URL)^1^, we used ADHD-related measures to detect whether symptoms of the disorder tracked well-learned habit expression and disruption.

## Materials and Methods

### Participants

To determine the sample size for our study, we performed an *a priori* power analysis on data from an existing study that examined inhibitory control capacity and ADHD-related symptoms (Wodushek and Neumann, 2003). In this study, healthy adults were categorized into high vs. low ADHD symptom groups for inhibitory control comparisons. We extracted effect sizes from the correlations between inhibitory control and non-verbal inattention in both symptom severity groups, and averaged the two resulting projected sample sizes. The averaged sample size needed to reach 80% statistical power was determined to be 105. We recruited 106 participants to make up for one participant’s corrupted data. Thus, 106 undergraduate students (79 female, 27 male; *M*_age_ = 20.23, *SD*_age_ = 4.07) from the Rutgers University-Newark campus participated for course credit. Informed consent was provided by all subjects per Declaration of Helsinki human subject protection guidelines. The Rutgers University Institutional Review Board approved study protocols. Individuals were excluded from participation for self-reported color-blindness. Two participants’ data were excluded from analyses due to attrition (*n* = 1) and data corruption (*n* = 1). Thus, the statistical analyses were performed on the remaining 104 participants (77 female, 27 male participants; *M*_age_ = 20.20, *SD*_age_ = 4.10).

### Materials and Procedures

**FIGURE 1:**
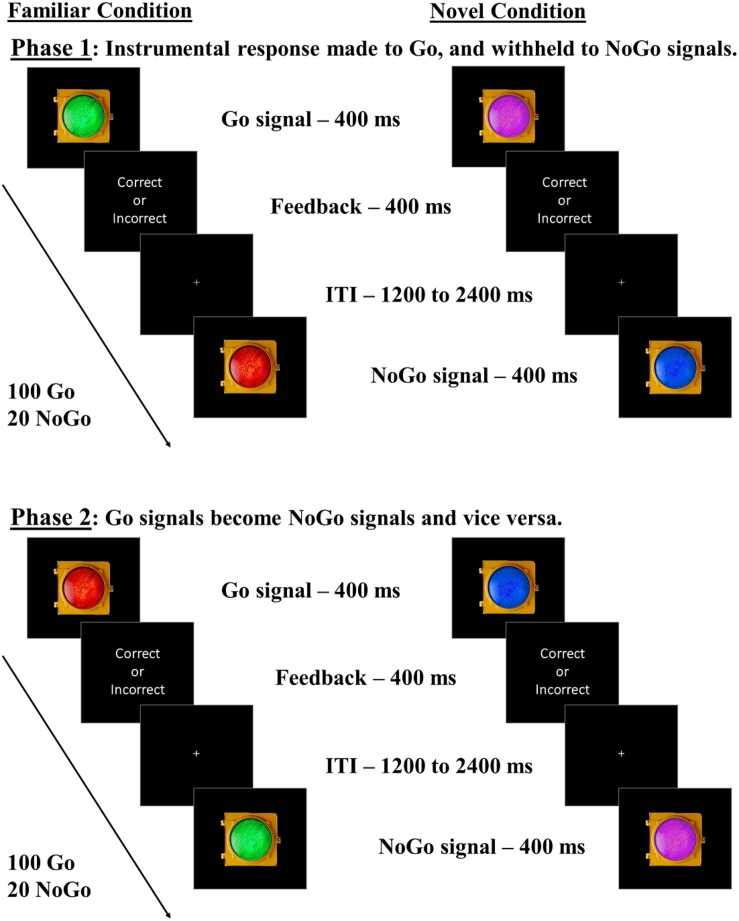
Go/NoGo task with familiar and novel lights. Participants undergo both Familiar and Novel conditions. In the Familiar condition, participants complete two phases: one in which green represents Go and red represents NoGo (“congruent” mapping), and one in which red represents Go and green represents NoGo (“incongruent” mapping). In the Novel condition, participants complete two similar phases, but the colors are blue and purple, for which we assume no strong pre-existing associations with go/stop responses. We predicted more commission errors in the Familiar condition for incongruent than congruent mappings, indicating outcome insensitivity, with no such within-subject differences expected in the Novel condition. Phase and Stim_Familiarity condition orders were counterbalanced across subjects.

Participants performed Go/NoGo tasks adapted from Ceceli et al. (2019) over 2 days. On day one, all participants underwent Go/NoGo tasks with familiar green and red traffic light stimuli (Familiar condition), and novel blue and purple traffic light stimuli (Novel condition) as Go and NoGo signals. Participants were instructed to respond as quickly and accurately to these stimuli as possible using the keyboard. A second phase followed in each Stim_Familiarity condition (Familiar/Novel conditions), where the color-response mappings were swapped (see Figure 1). In the Familiar condition, the Green-Go/Red–NoGo color-response mapping was considered “congruent” with daily experiences, while the Red–Go/Green–NoGo mapping was considered “incongruent,” in that it required the participant to override the well-established go and stop meanings of these stimuli. The Novel condition stimuli, however, are assumed to have no well-established Go or NoGo associations in daily life, in that the swapping of the color-response mappings should not require overriding associations that have been well-established. If familiar associations elicit habitual, cue-driven behavioral control, participants should experience a significant impairment in NoGo accuracy when green is mapped with NoGo. In the Novel condition, participants should perform similarly when managing either color-response mapping due to blue and purple not being strongly associated with Go/NoGo signals, reflecting goal-directed performance. We counterbalanced the order in which participants underwent the two phases within each Stim_Familiarity condition to ensure that our results were not due to a specific order of managing color-response contingencies. We also counterbalanced the order in which participants underwent the Familiar and Novel conditions. Lastly, participants completed the Adult ADHD Self-Report Scale (ASRS), a two-part survey that captures inattentive and hyperactive symptom manifestation associated with ADHD (Kessler et al., 2005a), and a demographic survey, concluding day one’s procedures.

Day two was completed within 3 days of day one and examined the potential habit-disrupting effect of a motivational enhancement. We separated these sessions by at least 1 day to minimize potential training effects. On day two, all participants underwent the Familiar condition of the Go/NoGo task, completing the “congruent” color-mapping first. Next, we induced motivational enhancement via the delivery of cumulative performance feedback and a monetary incentive. Specifically, participants’ cumulative task performance was displayed as a percentage score on the screen. Additionally, the experimenter briefly left the room, returning shortly after with a $5 cash bonus. The participants were informed that the $5 bonus was due to their performance on the task. The participants were then instructed to perform the “incongruent” color-mapping of the Familiar condition, and were informed that they may receive another performance-contingent cash bonus afterward. Unbeknownst to the participants, the mid-session cash bonus was not actually contingent on performance. We did not counterbalance color-mapping of Go/NoGo contingencies on day two to render the congruent color-mapping performance as baseline. Thus, we were able to test whether the presence of a mid-experiment motivational manipulation affected subsequent incongruent color-mapping performance (i.e., overriding the green-Go/red-NoGo habit). Lastly, participants completed the Creature of Habit Survey (COHS) (Ersche et al., 2017), quantifying the frequency of daily habitual tendencies, and a brief post-experiment questionnaire.

In each phase, there was a 5:1 Go/NoGo ratio, with 100 Go and 20 NoGo trials. Each Go/NoGo stimulus remained on the screen for 400 ms. Participants were required to respond to Go signals before the offset of the stimulus for a correct response. After offset, each response produced a brief “correct” or “incorrect” text slide. To ensure engagement with the task, inter-trial intervals varied randomly between 1200 and 2400 ms. Participants completed a practice session prior to each Stim_Familiarity condition, which consisted of six correct Go or NoGo responses using that condition’s stimuli. The experimenter remained present to ensure the instructions were understood during the practice sessions.

### Data Analysis

We pre-registered our task procedures and analyses prior to data collection via the Open Science Framework project registration portal (document URL: see text footnote 1). Analyses that were not outlined in our pre-registration document are marked as exploratory below. Data analysis was performed using the nlme package in R (version 3.5.1).

We used NoGo accuracy as our primary measure of outcome-sensitivity,as the moderate Go to NoGo ratio was hypothesized to produce prepotent Go responses (Young et al., 2018). NoGo accuracy has been the gold standard in studying behavioral control (Schulz et al., 2007; Meule, 2017). We selected this measure as our primary outcome of interest because our hypotheses are grounded in the idea that overriding the prepotent Go response will differ based on the real-world familiarity associated with color-response mappings in the task, and be further driven by ADHD symptom severity. As a secondary measure of outcome-sensitivity, we also performed all analyses using Go accuracy to supplement our assertions of differential outcome-sensitivity across Familiar and Novel conditions, and reveal the potential role of ADHD symptom severity in contributing to outcome-sensitivity. An alternative method of reporting Go/NoGo results is centered on the signal detection approach, in which Z-scored “hits” are subtracted from Z-scored “false alarms” to derive a sensitivity bias estimate for that particular run (Stanislaw and Todorov, 1999). However, this approach may complicate extracting color-specific accuracy information that is spread out over multiple runs—for example, extracting a sensitivity bias for green would require hits from the congruent, and false alarms from the incongruent run. Nonetheless, when sensitivity biases are derived on familiarity and congruency (e.g., when measured using Green-Go hits together with Red-NoGo false alarms to yield a sensitivity bias for the familiar-congruent mapping) the results mirror the analyses reported here using traditional accuracy rates. The corresponding signal detection analyses can be found in our shared analysis scripts and data output materials in the section Supplementary Data Sheet 1, “Signal Detection Analyses” in Supplementary Material.

Participants with standardized residuals less than −3.3 and greater than 3.3 were identified as outliers (Tabachnick and Fidell, 2007). Analyses excluding outliers are reported if data removal produces substantial changes in results (i.e., changes in statistical significance of any regressor). Bootstrapped 95% confidence interval values for all model regressors are included in their corresponding data tables (1000 bootstrap iterations in each model).

#### ADHD Symptom Severity and Well-Learned Habits

We performed an omnibus hierarchical multiple regression test to discern the contributions of symptom severity on outcome-sensitivity within Familiar and Novel condition data collected on day 1. This hierarchical structure permitted us to extract information about the amount of variance explained by groups of regressors (i.e., controlled variables, individual difference measures, and experimental variables), while also obtaining the predictive strengths of each individual regressor. Importantly, each additional step in the hierarchy updates the parameter estimates of the regressors in the previous steps, such that we are also able to detect how controlled variables may influence other regressors of interest. We used ΔNoGo_Accuracy (i.e., change in NoGo accuracy scores across mappings) as our dependent variable (DV) to measure the within-subject mapping-related change in accuracy. A greater mapping-related impairment represents greater outcome-insensitivity (e.g., heightened difficulty overriding a color-response mapping). In a hierarchical structure, we first input the regressors Age, Gender, Stim_Familiarity_Order (order in which participants underwent Familiar and Novel conditions), Phase_Order (order in which participants underwent color-response mappings within each Stim_Familiarity condition), and Driving (each participant’s experience driving, scaled in months), with Subject as a random factor into a linear mixed model. This model extracted the predictive strength of each of these controlled variables on outcome-sensitivity. In the next hierarchical step, we added the regressors ASRS_Inattentive (part A of the ASRS measure capturing symptoms of inattention), ASRS_Hyperactive (part B of the ASRS measure capturing symptoms of hyperactivity), and ASRS_Total (parts A and B aggregated to derive a composite score of ADHD symptom severity). Because our sample included six participants who had received ADHD diagnoses, we also input a Diagnosis regressor to determine whether clinical manifestation of ADHD – albeit in a small proportion of participants – affects outcome-sensitivity. We used COHS scores as a regressor to find potential correlations with tendency to behave habitually in daily life and outcome-sensitivity in our task. These regressors served to explain the main effects of each individual difference measure on outcome-sensitivity. In the third step of the hierarchical model, we input Stim_Familiarity (Familiar/Novel) as a regressor to specifically detect whether participants exhibited differential outcome-sensitivity across Familiar and Novel conditions. A significant contribution of this variable would confirm that the familiar red and green stimuli indeed elicit outcome-insensitive, habitual control, while the novel stimuli are labile, and thus controlled by goal-directed processes. We performed *post hoc t*-tests of NoGo accuracy between phases in each Stim_Familiarity condition to ascertain differential mapping-related impairment across Familiar and Novel conditions. Lastly, because of our specific focus on the influence of ADHD symptomology on habitual control, we also entered all individual difference measures’ interactions with Stim_Familiarity as regressors (e.g., ADHD_Inattentive × Stim_Familiarity) into step four of the model. Thus, we were able to distinguish the effects of each variable on outcome-sensitivity across Familiar and Novel conditions.

In brief, we expected the controlled demographic and counterbalancing variables (Age, Gender, Driving, Stim_Familiarity_Order, and Phase_Order) to be trivial in predicting outcome-sensitivity. We did not expect the Driving regressor to play a significant role in altering outcome-sensitivity, as we expect our well-learned habit task to capture well-established associations that extend beyond experience with these color-response mappings in a traffic context. We input both main effect and interaction regressors related to individual differences in ADHD symptomology and daily habitual tendencies to reveal potential associations with outcome-sensitivity. This way, we were able to inquire whether these individual difference regressors yielded strong associations with global outcome-sensitivity (i.e., main effects predicting mapping-related impairments independent of stimulus familiarity), and further interrogate whether such an association existed with well-learned habit expression in particular (i.e., ADHD-related measure × Stim_Familiarity interaction predicting an effect on outcome-sensitivity differentially across Familiar/Novel conditions). We also expected Stim_Familiarity to serve as a significant predictor in driving outcome-sensitivity, as the Familiar condition stimuli should selectively elicit outcome-insensitive habits, while the Novel condition stimuli should have no such effect on behavior.

#### ADHD Symptom Severity and Habit Disruption

We have previously shown the habit-disrupting effect of cumulative performance and monetary feedback (Ceceli et al., 2019). Here, we test via another omnibus regression whether ADHD symptom severity predicts habit disruption success. We performed a similar linear mixed model on the aggregate of Familiar data across 2 days, encompassing performance to the Familiar stimuli with and without feedback. We input our controlled variables of Age, Gender, Driving, Stim_Familiarity_Order, and Phase_Order, with Subject as a random factor into the first step. Our model similarly included ASRS_Inattentive, ASRS_Hyperactive, ASRS_Total, Diagnosis, and COHS in the second step to detect the main effects of individual differences on outcome-sensitivity. In the third step, our regression included a Feedback regressor that coded the availability of the mid-experiment dual-feedback manipulation. Because this analysis was performed only on the Familiar condition data (the Novel condition was not administered on the second day with feedback), we included no Stim_Familiarity regressor. Lastly, we included in step 4 our individual difference measures’ interactions with Feedback as regressors (e.g., ASRS_Inattentive × Feedback) to examine habit disruption per variations in ADHD-related behaviors and daily habitual tendencies.

Similar to our previous omnibus regression, we expected trivial contribution from our controlled variables, but a significant contribution from the Feedback regressor, as the delivery of dual feedback should disrupt the well-learned habit. We expected that symptom severity may affect outcome-sensitivity globally (significant main effects of individual difference measures), but also differentially across Feedback sessions (e.g., significant contribution of ADHD_Inattentive × Feedback). Additionally, we identified an alternative hypothesis – the possibility of habit disruption across the board (pre-registration document, Hypothesis 2b_alt). We expected no directionality in subtypes governing outcome-sensitivity (as in, inattentiveness or hyperactivity specifically driving habits), but we do note that if either subtype plays a major role in driving motivational control in the previous omnibus regression detecting the role of symptom severity on habitual control, that same subtype should predict habit disruption. We expected the frequency of habitual tendencies in daily life, as assayed by COHS, to yield a negative correlation with habit disruption (i.e., a significant COHS × Feedback result).

#### Supplementary Index of Outcome-Sensitivity: Go Accuracy

We used Go accuracy as a supplemental measure of outcome-sensitivity. Thus, we repeated all mixed models that examined ΔNoGo_Accuracy using ΔGo_Accuracy as DV.

#### Exploratory Analyses: Go RT and Individual Difference Measures

We extended our analyses beyond the pre-registered plans and explored the potential correlations between Go reaction time (RT) and our individual difference measures of symptom severity (ASRS_Inattentiveness and ASRS_Hyperactivity) and daily habitual tendencies (COHS). These variables were entered into a correlation matrix, and Pearson’s *r* values were corrected for multiple comparisons using the Holm–Bonferroni method. Specifically, we expected a negative correlation between RT and our individual difference measures. Most notably, we expected such an association between RT and ASRS_Hyperactivity, which would suggest quicker familiar Go actions to be associated with pronounced hyperactivity.

## Results

### ADHD Symptom Severity and Well-Learned Habits

We performed a linear mixed model using ΔNoGo_Accuracy as the DV and Subject as a random factor to determine whether ADHD symptom severity significantly predicts outcome-sensitivity in our well-learned habit task. Our proposed model violated the assumptions of non-multicollinearity, in that three pairs of fixed factors were highly correlated with each other (for the associated Variance Inflation Factors, see section “Supplementary Material”). Thus, we report the analyses as registered in the section “Supplementary Material,” and report below an adjusted model that meets the assumptions of non-multicollinearity, normality and homoscedasticity (see Table 1). Specifically, we revised our model to remove the regressors Age, Stim_Familiarity_Order, and ASRS_Total to prevent multicollinearity with the regressors Driving, Phase_Order, and ASRS_Inattentive/Hyperactive that are more crucial for our hypotheses.

**TABLE 1:** Hierarchical mixed model of ADHD symptomology and habit expression: ΔNoGo_Accuracy.

**Variable**	***VIF***	**β *(SE)***	***B [95% CI]***	***t***	***sig.***
**Model 1**					
Gender	**1.01**	−**0.15**(**0.07**)	−**0.06**[ −**0.11**, > −**0.01**]	**−2.13**	**0.036**
Phase_Order	1.01	−0.01(0.07)	> −0.01[ −0.02,0.02]	–0.09	0.931
Driving	1.00	0.08 (0.07)	< 0.01[ > −0.01, < 0.01]	1.15	0.252
**Model 2**					
Gender	**1.08**	−**0.14**(**0.07**)	−**0.05**[ −**0.11**, > −**0.01**]	**−2.00**	**0.049**
Phase_Order	1.04	−0.01(0.07)	> −0.01[ −0.02,0.02]	–0.15	0.877
Driving	1.30	0.08 (0.08)	< 0.01[ > −0.01, < 0.01]	1.08	0.283
ASRS_Inattentive	1.62	−0.01(0.09)	> −0.01[ −0.01, < 0.01]	–0.08	0.939
ASRS_Hyperactive	1.71	0.05 (0.09)	< 0.01[ > −0.01,0.01]	0.54	0.591
Diagnosis	1.30	0.01 (0.08)	0.01[ −0.11,0.12]	0.14	0.891
COHS	1.06	−0.04(0.07)	> −0.01[ > −0.01, < 0.01]	–0.60	0.548
**Model 3**					
Gender	**1.08**	−**0.14**(**0.07**)	−**0.05**[ −**0.10**, > −**0.01**]	**−2.10**	**0.039**
Phase_Order	1.04	−0.01(0.07)	> −0.01[ −0.02,0.02]	–0.16	0.871
Driving	1.30	0.09 (0.08)	< 0.01[ > 0.01, < 0.01]	1.13	0.260
ASRS_Inattentive	1.62	−0.01(0.08)	> −0.01[ > −0.01, < 0.01]	–0.08	0.936
ASRS_Hyperactive	1.71	0.05 (0.09)	< 0.01[ > −0.01,0.01]	0.57	0.573
Diagnosis	1.30	0.01 (0.08)	0.01[ −0.10,0.11]	0.14	0.885
COHS	1.06	−0.04(0.07)	> −0.01[ > −0.01, < 0.01]	–0.63	0.528
Stim_Familiarity	**1**	**0.31**(**0.07**)	**0.10**[**0.06**,**0.15**]	**4.66**	**> 0.001**
**Model 4**					
Gender	**1.08**	−**0.14**(**0.07**)	−**0.05**[ −**0.10**, > −**0.01**]	**−2.11**	**0.039**
Phase_Order	1.04	−0.01(0.07)	> −0.01[ −0.02,0.02]	–0.16	0.871
Driving	1.30	0.09 (0.08)	< 0.01[ > −0.01, < 0.01]	1.14	0.260
ASRS_Inattentive	3.17	−0.01(0.08)	< 0.01[ −0.01,0.01]	–0.08	0.936
ASRS_Hyperactive	3.31	0.05 (0.09)	< 0.01[ > −0.01,0.01]	0.57	0.573
Diagnosis	2.35	0.01 (0.08)	−0.06[ −0.20,0.08]	0.14	0.885
COHS	2.12	−0.04(0.07)	> −0.01[ > −0.01, < 0.01]	–0.64	0.528
Stim_Familiarity	**64.79**	**0.31**(**0.07**)	**0.13**[ −**0.47**,**0.22**]	**4.68**	**> 0.001**
ASRS_Inattentive × Stim_Familiarity	16.51	−0.02(0.08)	> −0.01[ −0.01,0.01]	–0.29	0.774
ASRS_Hyperactive × Stim_Familiarity	13.60	−0.05(0.08)	> −0.01[ −0.01,0.01]	–0.56	0.575
Diagnosis × Stim_Familiarity	2.16	0.10 (0.07)	0.15[ −0.04,0.34]	1.51	0.134
COHS × Stim_Familiarity	57.37	0.12 (0.07)	< 0.01[ > −0.01,0.01]	1.74	0.085

**Model comparisons**					

**Model**	***R*^2^**	***Log likel.***	**χ^2^**	**χ^2^*sig.***	**Δ*R*^2^**

Model 1	0.03	79.53			
Model 2	0.03	79.87	0.70	0.952	> 0.01
Model 3	**0.13**	**90.64**	**21.53**	**> 0.001**	**0.10**
Model 4	0.15	93.73	6.19	0.186	0.03

*Top layer of table depicts all regressors included in the hierarchical model. Model Comparisons layer depicts the predictive strength of each model, as compared to its previous step. VIF, Variance Inflation Factor; SE, Standard Error; CI, Confidence Interval; Log likel., Log likelihood. Significant *p*-values depicted in bold typeface. Analyses have been outlier corrected, with resulting deviations highlighted in the text. 95% confidence intervals were obtained by bootstrapping 1000 samples in each model.*

**FIGURE 2:**
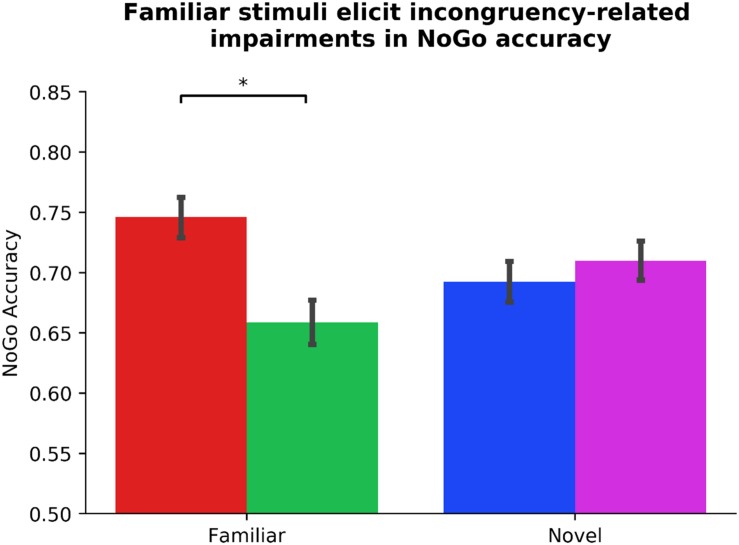
Familiar stimuli elicit incongruency-related impairments in NoGo accuracy. Participants exhibit outcome-insensitivity when managing familiar stimuli with color-response mappings that are incongruent with their daily experiences (*p* < 0.001). Newly learned Go/NoGo signals evoke no significant change in NoGo accuracy regardless of color-response mapping, indicating intact goal-directed performance (*p* = 0.279). The differential habit expression effect across Stim_Familiarity conditions depicted here is independent from ADHD symptom severity (see Table 1 for individual difference measure contributions to habit expression). Color of bars reflects NoGo stimulus colors.

Standard within-group residuals were within −3.3 and 3.3; thus, no participants were identified as outliers (Tabachnick and Fidell, 2007). In the first step of our hierarchical mixed model, contrary to our hypothesis, Gender significantly predicted outcome-sensitivity, β_Gender_ = −0.15, *p* = 0.036, in that female participants displayed significantly worse mapping-related impairments. Neither Driving experience nor the counterbalancing variable, Phase_Order, predicted outcome-sensitivity (*p*s > 0.252), model *R*^2^ = 0.03. In the second step of the model, we added the individual difference measures of ADHD symptom severity, clinical ADHD diagnosis, and frequency of habitual tendencies in daily life (COHS). We found no main effects of individual difference measures on outcome-sensitivity (all *p*s > 0.548). The log likelihood estimate derived by comparing first and second steps of our model yielded no significant global (as in, non-Stim_Familiarity specific) contribution attributable to the ASRS_Inattentive, ASRS_Hyperactive, Diagnosis, and COHS regressors, χ^2^(4) = 0.70, *p* = 0.952, *R*^2^ = 0.03, Δ*R*^2^ < 0.01. In the third step, we entered the Stim_Familiarity regressor, which significantly improved the predictive strength of the model, χ^2^(1) = 21.53, *p* < 0.001, *R*^2^ = 0.13, Δ*R*^2^ = 0.10, β_Stim_Familiarity_ = 0.31, *t*(103) = 4.66, *p* < 0.001, meaning outcome-sensitivity was differentially affected by whether participants managed the Familiar or Novel versions of the task. *Post hoc t*-tests confirmed that mapping-related NoGo accuracy impairments were evident only when managing Go/NoGo contingencies in the Familiar condition, *t*(103) = 5.33, *p* < 0.001, while performance in the Novel condition was comparable regardless of color-mapping associations, *t*(103) = −1.09, *p* = 0.279 (see Figure 2). In the fourth step of the model, we input the interaction of each individual difference regressor with Stim_Familiarity to detect their potentially differential effects on outcome-sensitivity across Familiar and Novel conditions, but found no significant contribution from any ADHD-related or daily habit frequency variable (all *p*s > 0.085, χ^2^(4) = 6.19, *p* = 0.186, *R*^2^ = 0.15, Δ*R*^2^ = 0.03). These results suggest that our sample exhibited outcome-insensitive well-learned habits across the board, but the degree of habitual control as assessed by change in NoGo accuracy was not significantly related to ADHD symptom severity.

### ADHD Symptom Severity and Habit Disruption

**FIGURE 3:**
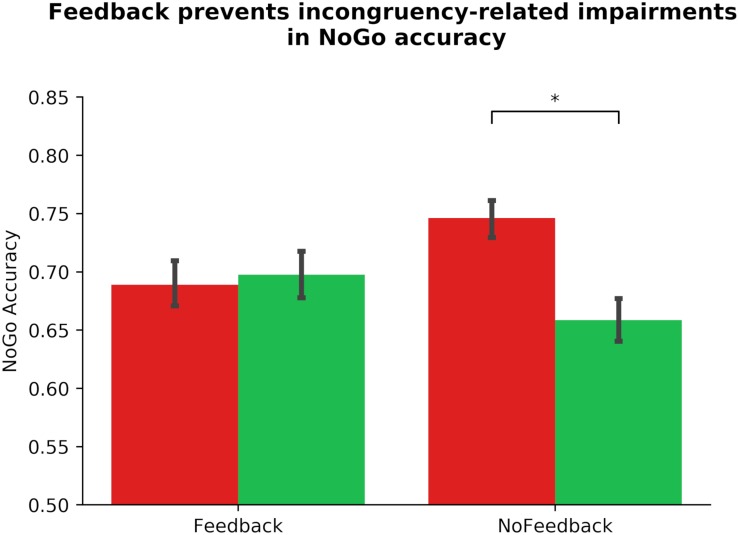
Dual monetary/performance feedback prevents the incongruency-related impairments in NoGo accuracy, breaking the habit. Participants exhibit no incongruency-related NoGo accuracy impairments after receiving cumulative performance and monetary feedback (*p* = 616). Without this feedback integration, participants exhibit a significant impairment in NoGo accuracy when the color-response mappings are incongruent with daily experiences (*p* < 0.001). The habit disruption effect of feedback is independent of ADHD symptom severity (see Table 2 for individual difference measure contributions to habit disruption). Color of bars reflects NoGo stimulus colors.

Similarly, we altered our pre-registered model to prevent multicollinearity, and performed a linear mixed model to examine the link between ADHD symptomology and habit disruption (see Table 2). The pre-registered analysis that violated assumptions of multicollinearity can be found in the section “Supplementary Material.” In our corrected model, we input Gender, Phase_Order, and Driving experience into step one, where none significantly predicted outcome-sensitivity (all *p*s > 0.142), model *R*^2^ = 0.01. In step two, we added ASRS_Inattentive, ASRS_Hyperactive, Diagnosis, and COHS into the model, and found that none of these regressors yielded main effects on outcome-sensitivity (all *p*s > 0.162), and they did not significantly improve the predictive strength of the model, χ^2^(4) = 3.19, *p* = 0.526, *R*^2^ = 0.03, Δ*R*^2^ = 0.01. We input Feedback as a regressor in step three, which contributed significantly to predicting outcome-sensitivity, β_Feedback_ = −0.28, *t*(103) = −4.13, *p* < 0.001, and rendered the model a significant predictor of ΔNoGo_Accuracy, χ^2^(1) = 17.10, *p* < 0.001, *R*^2^ = 0.11, Δ*R*^2^ = 0.08. We performed *post hoc* paired-samples *t*-tests to confirm the beneficial effect of dual feedback. We found that a significant NoGo accuracy impairment was evident in absence of dual feedback, *t*(103) = 5.33, *p* < 0.001, whereas the delivery of feedback yielded no significant accuracy impairments, *t*(103) = −0.50, *p* = 0.616 (see Figure 3). No individual difference measures’ interaction regressor in step four significantly predicted outcome-sensitivity (all *p*s > 0.391, χ^2^(4) = 1.56, *p* = 0.815, *R*^2^ = 0.11, Δ*R*^2^ = 0.01). These results suggest that the delivery of dual feedback indeed had a protective effect on outcome-sensitivity when managing familiar stimuli, albeit independent of ADHD symptom severity.

**TABLE 2:** Hierarchical mixed model of ADHD symptomology and habit disruption: ΔNoGo_Accuracy.

**Variable**	***VIF***	**β**	***B [95% CI]***	***t***	***sig.***
**Model 1**					
Gender	1.01	0.04 (0.07)	0.02[ −0.03,0.07]	0.60	0.553
Phase_Order	1.01	0.10 (0.07)	0.02[ −0.01,0.04]	1.48	0.142
Driving	1.00	−0.02(0.07)	> −0.01[ > −0.01, < 0.01]	–0.28	0.779
**Model 2**					
Gender	1.08	0.04 (0.07)	0.02[ −0.04,0.07]	0.62	0.537
Phase_Order	1.04	0.09 (0.07)	0.01[ −0.01,0.03]	1.25	0.215
Driving	1.30	0.02 (0.08)	> −0.01[ > −0.01, < 0.01]	0.24	0.807
ASRS_Inattentive	1.62	−0.06(0.09)	> −0.01[ −0.01, < 0.01]	–0.69	0.491
ASRS_Hyperactive	1.71	0.10 (0.09)	< 0.01[ > −0.01,0.01]	1.12	0.263
Diagnosis	1.30	−0.05(0.08)	−0.04[ −0.16,0.08]	–0.61	0.542
COHS	1.06	−0.10(0.07)	> −0.01[ > −0.01, < 0.01]	–1.41	0.162
**Model 3**					
Gender	1.08	0.04 (0.07)	0.02[ −0.03,0.07]	0.64	0.521
Phase_Order	1.04	0.09 (0.07)	0.01[ −0.01,0.03]	1.30	0.198
Driving	1.30	0.02 (0.08)	> −0.01[ > −0.01, < 0.01]	0.25	0.799
ASRS_Inattentive	1.62	−0.06(0.08)	> −0.01[ −0.01, < 0.01]	–0.72	0.474
ASRS_Hyperactive	1.71	0.10 (0.09)	< 0.01[ > −0.01,0.01]	1.17	0.245
Diagnosis	1.30	−0.05(0.08)	−0.04[ −0.15,0.07]	–0.64	0.526
COHS	1.06	−0.10(0.07)	> −0.01[ > −0.01, < 0.01]	–1.47	0.146
Feedback	**1**	−**0.28**(**0.07**)	−**0.09**[ −**0.14**,−**0.05**]	**−4.13**	**> 0.001**
**Model 4**					
Gender	1.08	0.04 (0.07)	0.02[ −0.03,0.07]	0.64	0.525
Phase_Order	1.04	0.09 (0.07)	0.01[ −0.01,0.07]	1.28	0.202
Driving	1.30	0.02 (0.08)	> −0.01[ −0.01,0.03]	0.25	0.801
ASRS_Inattentive	3.15	−0.06(0.08)	> −0.01[ −0.01, < 0.01]	–0.71	0.478
ASRS_Hyperactive	3.29	0.10 (0.09)	< 0.01[ > −0.01,0.01]	1.16	0.250
Diagnosis	2.34	−0.05(0.08)	−0.05[ −0.19,0.10]	–0.63	0.530
COHS	2.10	−0.10(0.07)	> −0.01[ > −0.01, < 0.01]	–1.45	0.150
Feedback	64.**79**	−**0.28**(**0.07**)	**0.01**[ −**0.34**,**0.37**]	**−4.12**	**> 0.001**
ASRS_Inattentive × Feedback	16.49	0.05 (0.08)	< 0.01[ −0.01,0.01]	0.62	0.539
ASRS_Hyperactive × Feedback	13.58	−0.01(0.08)	> −0.01[ −0.01,0.01]	–0.16	0.869
Diagnosis × Feedback	2.14	0.02 (0.07)	0.02[ −0.17,0.21]	0.24	0.811
COHS × Feedback	57.35	0.06 (0.07)	> −0.01[ > −0.01, < 0.01]	–0.86	0.391

**Model comparisons**					

**Model**	***R*^2^**	***Log likel.***	**χ^2^**	**χ^2^*sig.***	**Δ*R*^2^**

Model 1	0.01	72.53			
Model 2	0.03	74.13	3.19	0.526	0.01
Model 3	**0.11**	**82.68**	**17.10**	**> 0.001**	**0.08**
Model 4	0.11	83.46	1.56	0.815	0.01

*Top layer of table depicts all regressors included in the hierarchical model. Model Comparisons layer depicts the predictive strength of each model, as compared to its previous step. VIF, Variance Inflation Factor. SE, Standard Error. CI, Confidence Interval. Log likel., Log likelihood. Significant *p*-values depicted in bold typeface. Analyses have been outlier corrected, with resulting deviations highlighted in the text. 95% confidence intervals were obtained by bootstrapping 1000 samples in each model.*

### Supplementary Analysis of ADHD Symptom Severity and Well-Learned Habits

**FIGURE 4:**
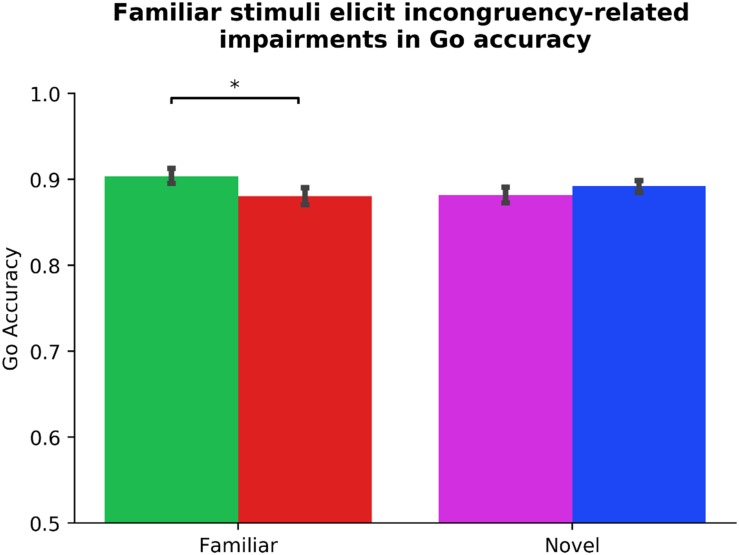
Familiar stimuli elicit incongruency-related impairments in Go accuracy. Analysis of our supplementary index of outcome-sensitivity, Go accuracy, yields evidence of habitual Go actions when managing familiar stimuli with color-response mappings that are incongruent with daily experiences (*p* < 0.001). In contrast, newly learned Go/NoGo contingencies evoke no significant change in Go accuracy regardless of color-response mapping, indicating intact goal-directed performance (*p* = 0.445). The differential habit expression effect across Stim_Familiarity conditions depicted here is independent from ADHD symptom severity (see Table 3 for individual difference measure contributions to habit expression). Color of bars reflects Go stimulus colors.

We performed identical analyses using ΔGo_Accuracy as DV and Subject as a random factor to capture the potential association between ADHD symptomology and a supplemental assay of outcome-sensitivity (see Table 3). Two participants’ data were identified as outliers. Due to changes in statistical significance following outlier correction, we report our outlier-removed dataset below, highlighting any change in statistical significance due to outlier correction. Neither Gender, Phase_Order, nor Driving experience predicted ΔGo_Accuracy (all *p*s > 0.323), model *R*^2^ = 0.01. In step two, the Diagnosis regressor, which codes for the presence of a clinical ADHD diagnosis, made a significant contribution, β_Diagnosis_ = 0.17, *t*(94) = 2.11, *p* = 0.038 (without outlier correction: β_Diagnosis_ = 0.14, *t*(96) = 1.80, *p* = 0.076). Specifically, the presence of a diagnosis predicted more flexible Go actions. No other step two regressor significantly predicted ΔGo_Accuracy (all *p*s > 0.259) The step two model was not significantly improved from step one, χ^2^(4) = 5.56, *p* = 0.235, *R*^2^ = 0.04, Δ*R*^2^ = 0.03. The Stim_Familiarity regressor in step three served as a significant predictor, β_Stim_Familiarity_ = 0.14, *t*(101) = 2.07, *p* = 0.010, improving the predictive strength of the model, χ^2^(1) = 4.44, *p* = 0.035, *R*^2^ = 0.06, Δ*R*^2^ = 0.02. Paired-samples *t*-tests revealed a significant Go accuracy impairment in the Familiar condition, *t*(101) = 3.80, *p* < 0.001, but not the Novel condition, *t*(101) = −0.77, *p* = 0.445 (see Figure 4). Lastly in step four, other than Diagnosis × Stim_Familiarity, β_Diagnosis × Stim_Familiarity_ = 0.19, *t*(97) = 2.71, *p* = 0.008, no individual difference measures significantly predicted ΔGo_Accuracy across the Familiar and Novel conditions (all other interaction *p*s > 0.125, χ^2^(4) = 10.43, *p* = 0.034, *R*^2^ = 0.10, Δ*R*^2^ = 0.05). Because we only had six individuals with an ADHD diagnosis, we refrain from further interpretations of the contribution of the Diagnosis regressor. These results suggest that Go accuracy is differentially affected by whether familiar or novel stimuli serve as Go/NoGo signals, and a significant impairment is evident when familiar contingencies are incongruent with daily experiences. However, the habitual Go actions elicited by our familiar stimuli are independent of ADHD symptom severity.

**TABLE 3:** Hierarchical mixed model of ADHD symptomology and habit expression: ΔGo_Accuracy.

**Variable**	***VIF***	**β**	***B [95% CI]***	***t***	***sig.***
**Model 1**					
Gender	1.01	< 0.01(0.07)	< 0.01[ −0.03,0.03]	–0.01	0.997
Phase_Order	1.01	0.07 (0.07)	< 0.01[ −0.01,0.01]	0.99	0.323
Driving	1.00	0.06 (0.07)	< 0.01[ > −0.01, < 0.01]	0.88	0.383
**Model 2**					
Gender	1.08	0.02 (0.07)	0.01[ −0.02,0.04]	0.34	0.731
Phase_Order	1.04	0.08 (0.07)	< 0.01[ −0.01,0.01]	1.13	0.260
Driving	1.30	−0.01(0.08)	> −0.01[ > −0.01, < 0.01]	–0.23	0.815
ASRS_Inattentive	1.59	0.02 (0.09)	> −0.01[ > −0.01, < 0.01]	0.22	0.828
ASRS_Hyperactive	1.66	−0.03(0.09)	> −0.01[ > −0.01, < 0.01]	–0.39	0.699
Diagnosis	**1.30**	**0.17**(**0.08**)	0.06[ −0.01,0.12]	**2.11**	**0.038**
COHS	1.06	−0.03(0.07)	> −0.01[ > −0.01, < 0.01]	–0.41	0.681
**Model 3**					
Gender	1.09	0.02 (0.07)	0.01[ −0.02,0.04]	0.35	0.729
Phase_Order	1.04	0.08 (0.07)	< 0.01[ −0.01,0.01]	1.14	0.256
Driving	1.30	−0.02(0.08)	> −0.01[ > −0.01, < 0.01]	–0.24	0.813
ASRS_Inattentive	1.59	0.02 (0.09)	> −0.01[ > −0.01, < 0.01]	0.22	0.826
ASRS_Hyperactive	1.66	−0.03(0.09)	> −0.01[ > −0.01, < 0.01]	–0.39	0.696
Diagnosis	1.30	**0.17**(**0.08**)	0.06[ > −0.01,0.12]	**2.13**	**0.036**
COHS	1.06	−0.03(0.07)	> −0.01[ > −0.01, < 0.01]	–0.42	0.678
Stim_Familiarity	**1**	**0.14**(**0.07**)	**0.03**[**0.01**,**0.06**]	**2.07**	**0.041**
**Model 4**					
Gender	1.09	0.02 (0.07)	0.01[ −0.02,0.04]	0.35	0.729
Phase_Order	1.04	0.08 (0.07)	< 0.01[ −0.01,0.01]	1.16	0.256
Driving	1.30	−0.02(0.08)	> −0.01[ > −0.01, < 0.01]	–0.24	0.813
ASRS_Inattentive	3.11	0.02 (0.09)	> −0.01[ > −0.01,0.01]	0.22	0.826
ASRS_Hyperactive	3.23	−0.03(0.09)	> −0.01[ −0.01, < 0.01]	–0.40	0.696
Diagnosis	**2.35**	**0.17**(**0.08**)	> −**0.01**[ −**0.08**,**0.07**]	**2.16**	**0.036**
COHS	2.12	−0.03(0.07)	> −0.01[ > −0.01, < 0.01]	–0.42	0.678
Stim_Familiarity	**65.83**	**0.14**(**0.07**)	< **0.01**[ −**0.19**,**0.21**]	**2.10**	**0.038**
ASRS_Inattentive × Stim_Familiarity	16.65	−0.08(0.08)	> −0.01[ −0.01, < 0.01]	–0.95	0.343
ASRS_Hyperactive × Stim_Familiarity	13.71	0.04 (0.09)	< 0.01[ > −0.01,0.01]	0.53	0.599
Diagnosis × Stim_Familiarity	**2.16**	**0.19**(**0.07**)	**0.12**[**0.01**,**0.23**]	**2.71**	**0.008**
COHS × Stim_Familiarity	57.08	0.11 (0.07)	< 0.01[ > −0.01, < 0.01]	1.55	0.125

**Model comparisons**					

**Model**	***R*^2^**	***Log likel.***	**χ^2^**	**χ^2^*sig.***	**Δ*R*^2^**

Model 1	0.01	218.44			
Model 2	0.04	221.22	5.56	0.235	0.03
Model 3	**0.06**	**223.44**	**4.44**	**0.035**	**0.02**
Model 4	**0.10**	**228.65**	**10.40**	**0.034**	**0.05**

*Top layer of table depicts all regressors included in the hierarchical model. Model Comparisons layer depicts the predictive strength of each model, as compared to its previous step. VIF, Variance Inflation Factor; SE, Standard Error; CI, Confidence Interval. Log likel., Log likelihood. Significant *p*-values depicted in bold typeface. Analyses have been outlier corrected, with resulting deviations highlighted in the text. 95% confidence intervals were obtained by bootstrapping 1000 samples in each model.*

### Supplementary Analysis of ADHD Symptom Severity and Habit Disruption

**FIGURE 5:**
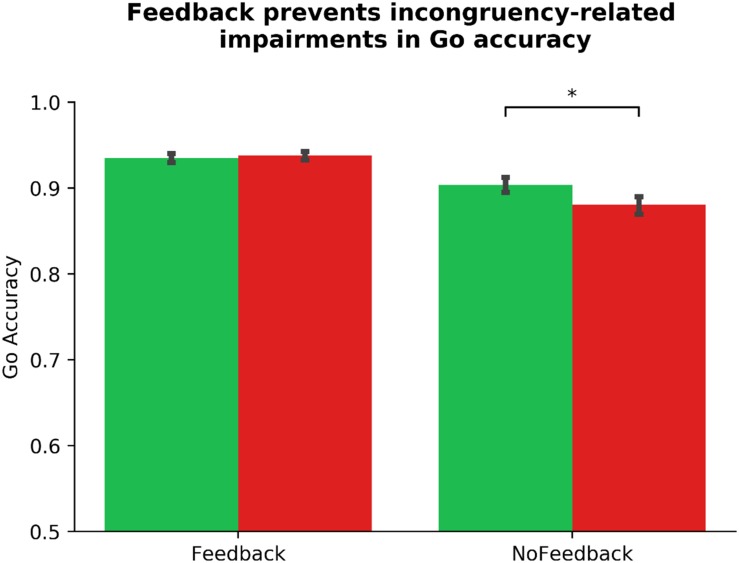
Dual monetary/performance feedback prevents the incongruency-related impairments in Go accuracy, breaking the habit. Similar to our NoGo accuracy results, analyses of the outcome-sensitivity measure of Go accuracy yield evidence for habit disruption due to cumulative performance and monetary feedback delivery. Participants exhibit no incongruency-related Go accuracy impairments after receiving dual feedback (*p* = 0.573). Without this feedback integration, participants exhibit a significant impairment in Go accuracy when the color-response mappings are incongruent with daily experiences (*p* < 0.001). The habit disruption effect of feedback is independent of ADHD symptom severity (see Table 4 for individual difference measure contributions to habit disruption). Color of bars reflects Go stimulus colors.

We investigated habit disruption via mapping-related changes in Go accuracy using a similar mixed model (see Table 4). Our multicollinearity-corrected model identified two outliers. We report outlier-removed results below, accompanied by any changes in statistical significance following outlier correction. In step one of the mixed model, no controlled regressors predicted ΔGo_Accuracy (all *p*s > 0.093), model *R*^2^ = 0.02. In step two, COHS was a near significant variable, β_COHS_ = −0.14, *t*(94) = −1.95, *p* = 0.054 (without outlier-correction: β_COHS_ = −0.08, *t*(96) = −1.05, *p* = 0.296), suggesting that a higher frequency of daily habits may predict more outcome-insensitive Go actions. Otherwise, no individual difference regressor served as a significant predictor of ΔGo_Accuracy (all other *p*s = 0.149), although the inclusion of step two regressors resulted in the Phase_Order variable to yield a near-significant *p*-value, *p* = 0.066. Step two regressors in aggregate yielded only a near-significant contribution on the DV, χ^2^(4) = 8.56, *p* < 0.073, *R*^2^ = 0.06, Δ*R*^2^ = 0.04. In step three, the Feedback regressor significantly predicted outcome-sensitivity as indexed by ΔGo_Accuracy, β_Feedback_ = −0.26, *t*(101) = −4.07, *p* < 0.001, improving the predictive strength of the model, χ^2^(1) = 16.01, *p* < 0.001, *R*^2^ = 0.13, Δ*R*^2^ = 0.07. This finding suggests that outcome-sensitivity as assessed by ΔGo_Accuracy is differentially impacted depending on the availability of dual feedback. Indeed, a *post hoc* paired-samples *t*-test confirms a significant impairment in Go accuracy when no feedback is delivered, *t*(103) = 3.85, *p* < 0.001, whereas with feedback, no such impairment is evident, *t*(103) = −0.56, *p* = 0.573 (see Figure 5). In step four, we found that COHS × Feedback significantly predicted habit disruption, β_COHS × Feedback_ = −0.16, *t*(97) = −2.46, *p* = 0.016 (without outlier-correction: *p* = 0.120), suggesting that an increased daily habit frequency predicts a reduction in the beneficial effects of dual feedback in restoring goal-directed control. No other individual difference × Feedback regressor predicted habit disruption (all *p*s > 0.188, χ^2^(4) = 9.70, *p* = 0.046, *R*^2^ = 0.16, Δ*R*^2^ = 0.04). Similar to our primary measure of outcome-sensitivity using NoGo accuracy, the protective effect of dual feedback on Go accuracy was independent from ADHD symptomology. However, we do observe a significant association between habitual tendencies in daily life and a difficulty in suppressing a well-learned habit.

**TABLE 4:** Hierarchical mixed model of ADHD symptomology and habit disruption: ΔGo_Accuracy.

**Variable**	***VIF***	**β**	***B [95% CI]***	***t***	***sig.***
**Model 1**					
Gender	1.02	−0.03(0.07)	−0.02[ −0.02,0.01]	–0.41	0.684
Phase_Order	1.02	0.12 (0.07)	< 0.01[ > −0.01,0.01]	1.69	0.093
Driving	1.00	−0.03(0.07)	> −0.01[ > −0.01, < 0.01]	–0.49	0.623
**Model 2**					
Gender	1.09	−0.03(0.07)	> −0.01[ −0.02,0.01]	–0.41	0.679
Phase_Order	1.04	0.13 (0.07)	0.01[ < 0.01,0.01]	1.86	0.066
Driving	1.30	−0.05(0.08)	> −0.01[ > −0.01, < 0.01]	–0.63	0.529
ASRS_Inattentive	1.59	0.02 (0.09)	> 0.01[ > −0.01, < 0.01]	0.27	0.788
ASRS_Hyperactive	1.66	−0.14(0.09)	> −0.01[ > −0.01, < 0.01]	–1.57	0.121
Diagnosis	1.30	−0.04(0.08)	−0.01[ −0.04,0.03]	–0.53	0.598
COHS	1.06	−0.14(0.07)	> −0.01[ > −0.01, < 0.01]	–1.95	0.054
**Model 3**					
Gender	1.09	−0.03(0.07)	> −0.01[ −0.02,0.01]	–0.42	0.678
Phase_Order	1.04	0.13 (0.07)	0.01[ < 0.01,0.01]	1.87	0.065
Driving	1.30	−0.05(0.08)	> −0.01[ > −0.01, < 0.01]	–0.63	0.527
ASRS_Inattentive	1.59	0.02 (0.09)	< 0.01[ > −0.01, < 0.01]	0.27	0.787
ASRS_Hyperactive	1.66	−0.14(0.09)	> −0.01[ > −0.01, < 0.01]	–1.57	0.119
Diagnosis	1.30	−0.04(0.08)	−0.01[ −0.04,0.03]	–0.53	0.596
COHS	1.06	−0.14(0.07)	> −0.01[ > −0.01, < 0.01]	–1.96	0.053
Feedback	**1**	−**0.26**(**0.06**)	−**0.03**[ −**0.04**,−**0.01**]	−**4.07**	**> 0.001**
**Model 4**					
Gender	1.09	−0.03(0.07)	> −0.01[ −0.02,0.01]	–0.41	0.681
Phase_Order	1.04	0.13 (0.07)	0.01[ < 0.01,0.01]	1.85	0.068
Driving	1.30	−0.05(0.08)	> −0.01[ > −0.01, < 0.01]	–0.62	0.531
ASRS_Inattentive	3.11	0.02 (0.09)	> −0.01[ > −0.01, < 0.01]	0.27	0.789
ASRS_Hyperactive	3.23	−0.14(0.09)	> −0.01[ > −0.01, < 0.01]	–1.56	0.123
Diagnosis	2.35	−0.04(0.08)	−0.01[ −0.05,0.03]	–0.53	0.600
COHS	2.12	−0.14(0.07)	< 0.01[ > 0.01, < 0.01]	–1.94	0.056
Feedback	**65.83**	−**0.26**(**0.06**)	**0.03**[ −**0.09**,**0.14**]	−**4.22**	**> 0.001**
ASRS_Inattentive × Feedback	16.65	0.10 (0.08)	< 0.01[ > −0.01,0.01]	1.33	0.188
ASRS_Hyperactive × Feedback	13.71	−0.07(0.08)	> −0.01[ > −0.01, < 0.01]	–0.83	0.410
Diagnosis × Feedback	2.16	< 0.01(0.06)	< 0.01[ −0.06,0.06]	0.02	0.984
COHS × Feedback	**57.08**	−**0.16**(**0.06**)	> −**0.01**[ > −**0.01**, < **0.01**]	−**2.46**	**0.016**

**Model comparisons**					

**Model**	***R*^2^**	***Log likel.***	**χ^2^**	**χ^2^*sig.***	**Δ*R*^2^**

Model 1	0.02	336.38			
Model 2	0.06	340.66	8.56	0.730	0.04
Model 3	**0.13**	**348.66**	**16.01**	**> 0.001**	**0.07**
Model 4	**0.16**	**353.52**	**9.70**	**0.046**	**0.04**

*Top layer of table depicts all regressors included in the hierarchical model. Model Comparisons layer depicts the predictive strength of each model, as compared to its previous step. VIF, Variance Inflation Factor; SE, Standard Error; CI, Confidence Interval; Log likel., Log likelihood. Significant *p*-values depicted in bold typeface. Analyses have been outlier corrected, with resulting deviations highlighted in the text. 95% confidence intervals were obtained by bootstrapping 1000 samples in each model.*

### Exploratory Analyses: Go RT and Individual Difference Measures

**FIGURE 6:**
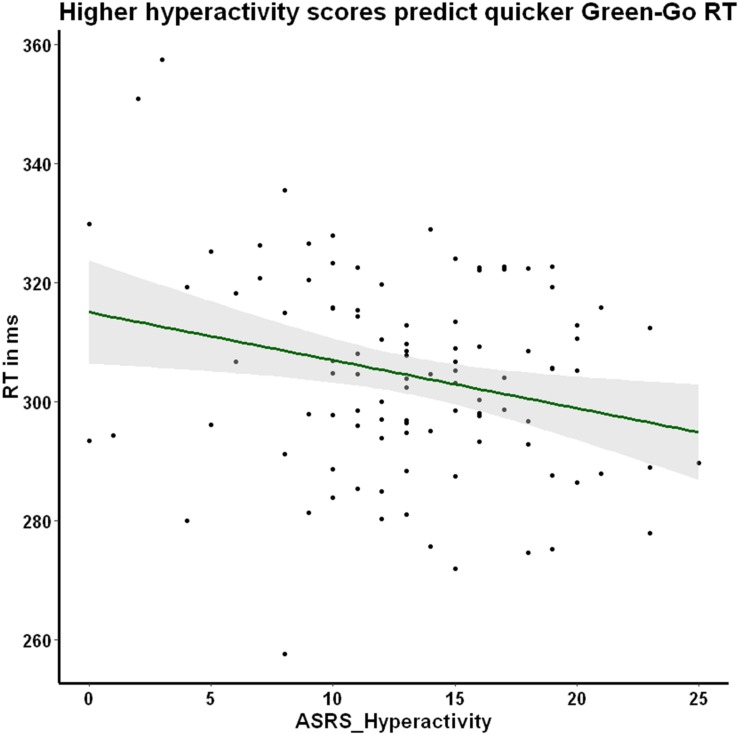
Hyperactivity symptom severity is negatively correlated with green-Go RT. Participants exhibit a significant negative correlation between hyperactivity symptoms and RT when responding to the familiar Go stimulus that is hypothesized to elicit prepotency. In other words, participants who score higher in hyperactivity make quicker Go responses when the contingencies are congruent with their daily representations. Pearson’s *r* = −0.25, *p* = 0.030, corrected for multiple comparisons using the Holm–Bonferroni method.

We explored the potential association between prepotency to respond to the familiar Go stimulus and our individual difference measures of ADHD symptom severity (ASRS_Inattentiveness and ASRS_Hyperactivity) and daily habit frequency (COHS). We reasoned that hyperactive individuals may exhibit a more pronounced prepotency to respond to Go stimuli, thus we were especially interested in the hyperactivity scale’s association with RT. As hypothesized, we found a significant negative correlation between Go RT to the familiar green-Go color-response mapping and ASRS_Hyperactivity, *r* = −0.25, *p* = 0.030, Holm–Bonferroni corrected (Figure 6), suggesting that higher hyperactivity scores are associated with faster Go responses. This relationship between hyperactivity and response latency was not apparent when the Go signal was incongruent with lifelong experiences (red-Go *r* = −0.05, *p* = 1, Holm–Bonferroni corrected), or when the Novel condition stimuli served as the Go signal (purple-Go *r* = −0.12, *p* = 0.630; blue-Go *r* = −0.10, *p* = 0.770, Holm–Bonferroni corrected). The association between familiar Go RT and ASRS_Hyperactivity may suggest that individuals high in hyperactive symptoms may be exhibiting abnormally pronounced prepotency to stimuli that evoke habitual control.

## Discussion

The neurobehavioral evidence of atypical reward-related processes in ADHD, and the scarcity of strategies to restore potential behavioral rigidities, motivated us to examine the expression and disruption of well-learned habits as a function of ADHD symptom severity. To this end, we collected ADHD symptom severity metrics from a wide sample of participants in the general population and administered our Go/NoGo task that capitalizes on familiar green-Go/red-NoGo associations. Importantly, our incorporation of a motivational enhancement manipulation (i.e., cumulative performance and monetary feedback) permitted the study of habit expression and disruption. Our results replicate our recent documentation of familiar Go/NoGo stimuli evoking rigid habitual control, which is also rendered more flexible (i.e., goal-directed) with motivational enhancement (Ceceli et al., 2019). However, we found only modest support for the hypothesis of ADHD symptomology tracking behavioral rigidity and habit disruption. No measure of ADHD significantly predicted outcome-insensitivity as assayed by color-response mapping-related NoGo or Go accuracy impairments. Our exploratory analyses, however, supported our hypothesis of a significant association between pre-potency of habitual Go actions (i.e., familiar green-Go RT) and hyperactivity presentation. Furthermore, although not directly associated with ADHD, we also found a link between the frequency of habitual tendencies in daily life and habit disruption as indexed by our supplementary measure of outcome-sensitivity: mapping-related Go accuracy impairments. This significant association between daily habit frequency and difficulty breaking well-learned Go associations lends further credence to the idea that the familiar associations we capitalize on are indeed related to well-established, ecologically relevant habits.

A cardinal indicator of habitual control is the performance of an action regardless of the outcome value (Dickinson and Balleine, 1994). Accordingly, we believe that our Go/NoGo task captures outcome-sensitivity, in that the contingency change requires the agent to update which action produces the desired outcome. An impairment in the ability to override the well-learned habit may cause difficulties in flexibly updating the associations between cues and actions (i.e., the color-response mappings) that yield desirable outcomes (e.g., the value of performing a correct action).

We assert that our familiar stimuli elicit outcome-insensitive habits due to their well-established nature. The newly formed associations (e.g., purple-Go) are more labile, allowing the agent to exert goal-directed control regardless of changes to the color-response mappings. By this logic, these novel associations should eventually elicit habitual control with sufficient exposure – similar to overtraining of S–R associations in rodents (Adams, 1982). The magnitude of training necessary for this switch in motivational control using a change in Go and NoGo contingencies remains unknown. Previous research has suggested that pre-training stimuli over the course of an extra training session can yield stronger S–R execution in comparison to new stimulus sets (McKim et al., 2016). Possibly, extensively training the novel associations in our paradigm may also produce habitual control, albeit not with the behavioral rigidity elicited by the familiar associations that have been associated with go and stop actions over the course of development.

In both scientific reports and diagnostic criteria, ADHD is characterized by pronounced deficits in inhibitory control (Wodka et al., 2007; American Psychiatric Association, 2013). When taken together with the reward-related irregularities, we posited that ADHD may also be associated with an impaired motivational control system favoring habits over goal-directed behaviors. Our results do not support this hypothesis with our primary analyses, which could be due to a few key factors.

First, our study recruited participants from the general population and obtained a normal distribution of ADHD-related symptom severity, such that most participants in our sample did not reach the clinical threshold for an ADHD diagnosis. This approach contextualizes any potential ADHD-related impairment in motivational processes to a wider audience, thus expanding the applicability of our research. Consequentially, we are unable to sufficiently represent those who are most debilitated by the symptoms in question: individuals who meet the clinical threshold for ADHD. Any potential ADHD-related effect may therefore be weakened by the large proportion of individuals who present symptoms below the clinical threshold at magnitudes that do not impair daily functioning. Indeed, a study that recruited adults from the general population to examine ADHD symptomology-related inhibitory control disparities found only a modest association between symptom severity and Go/NoGo task accuracy with 440 participants (Polner et al., 2015). A study with a larger sample size (*n* = 1156) obtained from the general population pinpointed Go/NoGo impairments due to high ADHD-like symptoms, though these effects were sensitive to variations in task structure (e.g., speed and reward structure) (Kuntsi et al., 2009). Taken together with our results, although the ADHD–Go/NoGo impairment association is well-documented in clinical presentations of ADHD, symptom-based approaches may not be sensitive to such effects in the general population. Nonetheless, although there may be disorder-specific factors playing a role in behavioral flexibility that are undetected here, we had reasoned that sampling indiscriminately – that is, without diagnostic cutoffs – could expand the generalizability of potential symptom-related anomalies to the public.

An alternative explanation for the absence of a strong link between motivational control and ADHD symptomology is the notion that individuals with ADHD-like symptoms may also have compensatory mechanisms that promote adaptive behavioral output. For instance, despite the strong evidence of response inhibition deficits in ADHD, attention compensation supported by parietal brain activity has been documented, resulting in comparable Go/NoGo task performance (Ersche et al., 2017). Another possibility is that individuals with ADHD may adopt habitual or goal-directed control in different circumstances. A design that capitalizes on varying task difficulty or cognitive demands may be able to reflect such shifts in habitual and goal-directed processes that are sensitive to individual differences. Brain maturation is another candidate for behavioral similarities in ADHD and NT populations. ADHD is associated with a delayed maturation of the prefrontal cortex (Shaw et al., 2007), a region that is critical for error detection, reversal learning, and conflict monitoring. These processes are crucial for optimal Go/NoGo task performance (Garavan et al., 2002; Zhang et al., 2016), especially one involving changes to color-response mappings. Accordingly, adults with ADHD may produce signs of intact Go/NoGo performance due to the maturations in prefrontal regions, compensating for potential impairments that may have been evident with a less mature cortex (Carmona et al., 2012). Another potential compensatory mechanism may be driven by ADHD medications that act on the brain’s dopaminergic systems. We did not ascertain whether our participants – with or without ADHD – were taking ADHD medication. Methylphenidate, for instance, has been reported to enhance executive function in individuals with ADHD, as well as in NTs (Schweitzer et al., 2004; Linssen et al., 2014; Moeller et al., 2014). These beneficial effects of ADHD medication on executive function have also been shown to extend beyond methylphenidate (Hosenbocus and Chahal, 2012). Our sample of adults with varying degrees of ADHD-related symptoms may be recruiting similar compensatory mechanisms that aid in maintaining goal-directed control. Future research that captures developmental and pharmacological aspects of ADHD and goal-directed control may elucidate which of these mechanisms plays a critical role in adaptive motivational control.

We reasoned that because hyperactive ADHD presentation is associated with the number of impulsivity-related items endorsed on the ASRS (Kessler et al., 2005a), participants exhibiting high hyperactivity may execute quicker, impulsive Go actions. Our green-Go RT data supported our hypothesis, in that hyperactivity scores correlated with quicker responses to the well-learned habit eliciting stimulus. It should be noted that this finding was the result of an exploratory analysis. Nonetheless, our finding of a significant response latency and hyperactivity association bridges the fields of motivation and ADHD. Impulsivity, a core element of the hyperactive presentation of ADHD, is also associated with reflexive behaviors to cues and heightened variability in response latency (Kirkeby and Robinson, 2005). The heightened pre-potency to respond to habitual cues tracked by our hyperactivity scale may suggest an overlap in the motivational and inhibitory mechanisms underlying hyperactivity in ADHD, potentially explaining the lapses in behavioral output that result in higher RT and accuracy variability (Kirkeby and Robinson, 2005; Tamm et al., 2012). In other words, if hyperactivity predicts quicker responses to well-learned stimuli and high RT variability, this effect may be due to motivational and motor processes that are activated depending on past experience with the cue at hand. Future research will be imperative in effectively dissociating the motivational, attentional, and inhibitory processes that underlie response latency variability in ADHD.

In addition to the analyses reported here, an alternative method of exploring Go and NoGo performance is via signal detection. In a typical signal detection analysis, hits, misses, false alarms, and correct rejection values are used to derive d’ – an estimate of response bias (Stanislaw and Todorov, 1999). Importantly, in each run of our task, a color-response mapping (e.g., green-Go) would only provide two of the four values that comprise a d’ score (i.e., hits and misses, but not false alarms or correct rejections for this color). The remaining parameters would need to be extracted from the “incongruent” run (green-NoGo), which would make it difficult to obtain accurate response bias information. However, one can indeed investigate signal detection based on familiarity and congruency of the color-response mappings (e.g., where green-Go and red-NoGo together are coded as d’_familiar_congruent, and green-NoGo, red-Go are together coded as d’_familiar_incongruent). When performed as such, response bias results mirror our NoGo and Go accuracy findings reported here, in that (1) participants show high response bias when the color-response mappings are familiar and congruent with daily experiences, (2) response bias is significantly lower when familiar stimuli are mapped onto incongruent responses, (3) the two novel color-response mappings are similar in the elicited response bias, and (4) response bias does not show significant associations with ADHD-related individual difference measures.

### Limitations

We acknowledge several limitations in the present study that should be considered in future investigations.

Although we were able to generalize our findings to a wider audience by recruiting without diagnostic cutoffs, we did not survey participants for history of psychiatric illnesses or psychoactive medication use. Several psychiatric conditions have been documented to affect motivational control (Griffiths et al., 2014). Furthermore, ADHD medications have been shown to improve executive function (Hosenbocus and Chahal, 2012; Linssen et al., 2014; Moeller et al., 2014), which may be related to the expansion of cognitive resources necessary to maintain goal-directed control. An interesting avenue to explore in future ADHD research may be the roles of psychiatric comorbidities and treatment history in the expression of habits.

Our study’s primary hypotheses regarding habitual control and ADHD symptomology were motivated by reports of reward circuitry dysfunction in ADHD (Ceceli et al., 2019). However, we did not collect neural data that may speak to the potential links between ADHD symptomology and habitual control as mediated by neural function. The brain systems of reward processing and learning are outside the scope of our study, but the mechanisms underlying motivational control as related to ADHD symptoms may be effectively elucidated by a neurobiological approach. Future research that examines the potential disparities in the ADHD brain related to motivational control may advance our understanding of the disorder’s pathophysiology.

We adopted a within-subject design to tackle the expression and disruption of habits over the course of two sessions. This design permitted us to compare habit expression and disruption at an individual basis while improving statistical power. However, it can be argued that administering a task twice to the same set of participants may introduce training effects. Our second session data suggest that participants did not significantly improve their performance in the face of congruent associations by merely undergoing the task in the previous session. However, the definitive method to circumvent potential training effects would be to apply a between-subjects design, in which separate sets of participants undergo the feedback and no-feedback sessions. We report in another study that adopts a between-subject design a similar pattern of results – motivational enhancement indeed disrupts the expression of well-learned habits (Ceceli et al., 2019).

## Conclusion

Attention deficit-hyperactivity disorder is a heterogenous psychiatric condition with debilitating consequences to behavior, neural processing, and well-being. In this study, we aimed to reveal the potential irregularities in managing well-learned habits by sampling symptom severity information from the general population. Although we did not find a strong association between motivational control deficits and ADHD-related symptoms, our data replicate a previous report of well-learned habit expression and disruption, and allude to a link between hyperactivity and pre-potency to respond to well-learned Go stimuli. Taken together with previous reports of compensatory mechanisms aiding in Go/NoGo task performance in ADHD, delay in cortical maturation in ADHD yielding differential inhibitory processes across children and adults, and our sample largely comprising subclinical ADHD presentations, a full understanding of the potential link between ADHD and motivational control may require a neurobehavioral and developmental approach.

## Data Availability

The datasets and scripts associated with this study can be found in the Supplementary Materials.

## Ethics Statement

This study was carried out in accordance with the recommendations of the Rutgers University Institutional Review Board with written informed consent from all subjects. All subjects gave written informed consent in accordance with the Declaration of Helsinki. The protocol was approved by the Rutgers University Institutional Review Board.

## Author Contributions

AC and ET designed the experiments. AC and GE coded the experimental paradigm and collected the data. All authors contributed to the data analysis and manuscript preparation.

## Conflict of Interest Statement

The authors declare that the research was conducted in the absence of any commercial or financial relationships that could be construed as a potential conflict of interest.

## Footnotes

1 https://osf.io/fjcbw

## References

[B1] Adams C. D. (1982). Variations in the sensitivity of instrumental responding to reinforcer devaluation. *Q. J. Exp. Psychol. Sect. B* 34 77–98. 10.1080/14640748208400878

[B2] American Psychiatric Association. (2013). *Diagnostic and Statistical Manual of Mental Disorders*, Fifth Edn Washington, DC: American Psychiatric Association.

[B3] Antrop I. Stock P. Verté S. Wiersema J. R. Baeyens D. Roeyers H. (2006). ADHD and delay aversion: the influence of non-temporal stimulation on choice for delayed rewards. *J. Child Psychol. Psychiatry* 47 1152–1158. 10.1111/j.1469-7610.2006.01619.x 17076754

[B4] Balleine B. W. O’Doherty J. P. (2009). Human and rodent homologies in action control: corticostriatal determinants of goal-directed and habitual action. *Neuropsychopharmacology* 35 48–69. 10.1038/npp.2009.131 19776734 PMC3055420

[B5] Carmona S. Hoekzema E. Ramos-Quiroga J. A. Richarte V. Canals C. Bosch R. (2012). Response inhibition and reward anticipation in medication-naïve adults with attention-deficit/hyperactivity disorder: a within-subject case-control neuroimaging study. *Hum. Brain Mapp.* 33 2350–2361. 10.1002/hbm.21368 21826761 PMC6870239

[B6] Castellanos F. X. Tannock R. (2002). Neuroscience of attention-deficit/hyperactivity disorder: the search for endophenotypes. *Nat. Rev. Neurosci.* 3 617–628. 10.1038/nrn896 12154363

[B7] Ceceli A. O. Myers C. E. Tricomi E. (2019). Demonstrating and disrupting well-learned habits. *bioRxiv* [Preprint] 10.1101/745356 PMC7292414 32530930

[B8] Ceceli A. O. Tricomi E. (2018). Habits and goals: a motivational perspective on action control. *Curr. Opin. Behav. Sci.* 20 110–116. 10.1016/j.cobeha.2017.12.005

[B9] Costa Dias T. G. Wilson V. B. Bathula D. R. Iyer S. P. Mills K. L. Thurlow B. L. (2013). Reward circuit connectivity relates to delay discounting in children with attention-deficit/hyperactivity disorder. *Eur. Neuropsychopharmacol.* 23 33–45. 10.1016/j.euroneuro.2012.10.015 23206930 PMC3581744

[B10] Dickinson A. Balleine B. (1994). Motivational control of goal-directed action. *Anim. Learn. Behav.* 22 1–18. 10.3758/BF03199951

[B11] Dillo W. Göke A. Prox-Vagedes V. Szycik G. R. Roy M. Donnerstag F. (2010). Neuronal correlates of ADHD in adults with evidence for compensation strategies – a functional MRI study with a Go/No-Go paradigm. *Ger. Med. Sci.* 8:Doc09. 10.3205/000098 20421953 PMC2858877

[B12] Ersche K. D. Lim T.-V. Ward L. H. E. Robbins T. W. Stochl J. (2017). Creature of habit: a self-report measure of habitual routines and automatic tendencies in everyday life. *Pers. Individ. Differ.* 116 73–85. 10.1016/j.paid.2017.04.024 28974825 PMC5473478

[B13] Frodl T. Skokauskas N. (2012). Meta-analysis of structural MRI studies in children and adults with attention deficit hyperactivity disorder indicates treatment effects. *Acta Psychiatr. Scand.* 125 114–126. 10.1111/j.1600-0447.2011.01786.x 22118249

[B14] Furukawa E. Bado P. Tripp G. Mattos P. Wickens J. R. Bramati I. E. (2014). Abnormal striatal BOLD responses to reward anticipation and reward delivery in ADHD. *PLoS One* 9:e89129. 10.1371/journal.pone.0089129 24586543 PMC3935853

[B15] Garavan H. Ross T. J. Murphy K. Roche R. A. P. Stein E. A. (2002). Dissociable executive functions in the dynamic control of behavior: inhibition, error detection, and correction. *Neuroimage* 17 1820–1829. 10.1006/nimg.2002.1326 12498755

[B16] Griffiths K. R. Morris R. W. Balleine B. W. (2014). Translational studies of goal-directed action as a framework for classifying deficits across psychiatric disorders. *Front. Syst. Neurosci.* 8:101. 10.3389/fnsys.2014.00101 24904322 PMC4033402

[B17] Haber S. N. (2003). The primate basal ganglia: parallel and integrative networks. *J. Chem. Neuroanat.* 26 317–330. 10.1016/j.jchemneu.2003.10.003 14729134

[B18] Hosenbocus S. Chahal R. (2012). A review of executive function deficits and pharmacological management in children and adolescents. *J. Can. Acad. Child Adolesc. Psychiatry* 21 223–229. 22876270 PMC3413474

[B19] Kessler R. C. Adler L. Ames M. Demler O. Faraone S. Hiripi E. (2005a). The World Health Organization adult ADHD self-report scale (ASRS): a short screening scale for use in the general population. *Psychol. Med.* 35 245–256. 10.1017/S0033291704002892 15841682

[B20] Kessler R. C. Adler L. A. Barkley R. A. Biederman J. Conners C. K. Faraone S. V. (2005b). Patterns and predictors of attention-deficit/hyperactivity disorder persistence into adulthood: results from the national comorbidity survey replication. *Biol. Psychiatry* 57 1442–1451. 10.1016/j.biopsych.2005.04.001 15950019 PMC2847347

[B21] Kirkeby B. S. Robinson M. D. (2005). Impulsive behavior and stimulus–response variability in choice reaction time. *J. Res. Personal.* 39 263–277. 10.1016/j.jrp.2004.04.001 1558571

[B22] Kluger A. N. DeNisi A. (1996). The effects of feedback interventions on performance: a historical review, a meta-analysis, and a preliminary feedback intervention theory. *Psychol. Bull.* 119 254–284. 10.1037/0033-2909.119.2.254

[B23] Kuntsi J. Wood A. C. Meere J. V. D. Asherson P. (2009). Why cognitive performance in ADHD may not reveal true potential: findings from a large population-based sample. *J. Int. Neuropsychol. Soc.* 15 570–579. 10.1017/S135561770909081X 19573275 PMC2844935

[B24] Linssen A. M. W. Sambeth A. Vuurman E. F. P. M. Riedel W. J. (2014). Cognitive effects of methylphenidate in healthy volunteers: a review of single dose studies. *Int. J. Neuropsychopharmacol.* 17 961–977. 10.1017/S1461145713001594 24423151

[B25] Marx I. Höpcke C. Berger C. Wandschneider R. Herpertz S. C. (2013). The impact of financial reward contingencies on cognitive function profiles in adult ADHD. *PLoS One* 8:e67002. 10.1371/journal.pone.0067002 23840573 PMC3688618

[B26] McKim T. H. Bauer D. J. Boettiger C. A. (2016). Addiction history associates with the propensity to form habits. *J. Cogn. Neurosci.* 28 1024–1038. 10.1162/jocn_a_00953 26967944 PMC5046041

[B27] Meule A. (2017). Reporting and interpreting task performance in go/no-go affective shifting tasks. *Front. Psychol.* 8:701. 10.3389/fpsyg.2017.00701 28536544 PMC5422529

[B28] Moeller S. J. Honorio J. Tomasi D. Parvaz M. A. Woicik P. A. Volkow N. D. (2014). Methylphenidate enhances executive function and optimizes prefrontal function in both health and cocaine addiction. *Cereb. Cortex* 24 643–653. 10.1093/cercor/bhs345 23162047 PMC3920764

[B29] Montague W. E. Webber C. E. (1965). Effects of knowledge of results and differential monetary reward on six uninterrupted hours of monitoring. *Hum. Factors* 7 173–180. 10.1177/001872086500700209 5861128

[B30] Natsheh J. Y. Shiflett M. W. (2015). The effects of methylphenidate on goal-directed behavior in a rat model of ADHD. *Front. Behav. Neurosci.* 9:326. 10.3389/fnbeh.2015.00326 26635568 PMC4659329

[B31] Natsheh J. Y. Shiflett M. W. (2018). Dopaminergic modulation of goal-directed behavior in a rodent model of attention-deficit/hyperactivity disorder. *Front. Integr. Neurosci.* 12:45. 10.3389/fnint.2018.00045 30344481 PMC6182263

[B32] Norman L. J. Carlisi C. Lukito S. Hart H. Mataix-Cols D. Radua J. (2016). Structural and functional brain abnormalities in attention-deficit/hyperactivity disorder and obsessive-compulsive disorder: a comparative meta-analysis. *JAMA Psychiatry* 73 815–825. 10.1001/jamapsychiatry.2016.0700 27276220

[B33] O’Doherty J. P. (2016). Multiple systems for the motivational control of behavior and associated neural substrates in humans. *Curr. Top. Behav. Neurosci.* 27 291–312. 10.1007/7854_2015_386 26370947

[B34] O’Doherty J. P. Dayan P. Schultz J. Deichmann R. Friston K. Dolan R. J. (2004). Dissociable roles of ventral and dorsal striatum in instrumental conditioning. *Science* 304 452–454. 10.1126/science.1094285 15087550

[B35] Plichta M. M. Scheres A. (2014). Ventral–striatal responsiveness during reward anticipation in ADHD and its relation to trait impulsivity in the healthy population: a meta-analytic review of the fMRI literature. *Neurosci. Biobehav. Rev.* 38 125–134. 10.1016/j.neubiorev.2013.07.012 23928090 PMC3989497

[B36] Polner B. Aichert D. Macare C. Costa A. Ettinger U. (2015). Gently restless: association of ADHD-like traits with response inhibition and interference control. *Eur. Arch. Psychiatry Clin. Neurosci.* 265 689–699. 10.1007/s00406-014-0531-7 25209569

[B37] Qiu A. Crocetti D. Adler M. Mahone E. M. Denckla M. B. Miller M. I. (2009). Basal ganglia volume and shape in children with attention deficit hyperactivity disorder. *Am. J. Psychiatry* 166 74–82. 10.1176/appi.ajp.2008.08030426 19015232 PMC2890266

[B38] Rosch K. S. Mostofsky S. H. Nebel M. B. (2018). ADHD-related sex differences in fronto-subcortical intrinsic functional connectivity and associations with delay discounting. *J. Neurodev. Disord.* 10:34. 10.1186/s11689-018-9254-9 30541434 PMC6292003

[B39] Schulz K. P. Fan J. Magidina O. Marks D. J. Hahn B. Halperin J. M. (2007). Does the emotional go/no-go task really measure behavioral inhibition? convergence with measures on a non-emotional analog. *Arch. Clin. Neuropsychol. Off. J. Natl. Acad. Neuropsychol.* 22 151–160. 10.1016/j.acn.2006.12.001 17207962 PMC2562664

[B40] Schweitzer J. B. Lee D. O. Hanford R. B. Zink C. F. Ely T. D. Tagamets M. A. (2004). Effect of methylphenidate on executive functioning in adults with attention-deficit/hyperactivity disorder: normalization of behavior but not related brain activity. *Biol. Psychiatry* 56 597–606. 10.1016/j.biopsych.2004.07.011 15476690

[B41] Shaw P. Eckstrand K. Sharp W. Blumenthal J. Lerch J. P. Greenstein D. (2007). Attention-deficit/hyperactivity disorder is characterized by a delay in cortical maturation. *Proc. Natl. Acad. Sci. U.S.A.* 104 19649–19654. 10.1073/pnas.0707741104 18024590 PMC2148343

[B42] Sonuga-Barke E. J. Taylor E. Sembi S. Smith J. (1992). Hyperactivity and delay aversion–I. The effect of delay on choice. *J. Child Psychol. Psychiatry* 33 387–398. 10.1111/j.1469-7610.1992.tb00874.x 1564081

[B43] Stanislaw H. Todorov N. (1999). Calculation of signal detection theory measures. *Behav. Res. Methods Instrum. Comput.* 31 137–149. 10.3758/BF03207704 10495845

[B44] Ströhle A. Stoy M. Wrase J. Schwarzer S. Schlagenhauf F. Huss M. (2008). Reward anticipation and outcomes in adult males with attention-deficit/hyperactivity disorder. *Neuroimage* 39 966–972. 10.1016/j.neuroimage.2007.09.044 17996464

[B45] Tabachnick B. G. Fidell L. S. (2007). *Using Multivariate Statistics*, 5th Edn Boston, MA: Allyn & Bacon.

[B46] Tamm L. Narad M. E. Antonini T. N. O’Brien K. M. Hawk L. W. Epstein J. N. (2012). Reaction time variability in ADHD: a review. *Neurotherapeutics* 9 500–508. 10.1007/s13311-012-0138-5 22930417 PMC3441931

[B47] Tricomi E. Balleine B. W. O’Doherty J. P. (2009). A specific role for posterior dorsolateral striatum in human habit learning. *Eur. J. Neurosci.* 29 2225–2232. 10.1111/j.1460-9568.2009.06796.x 19490086 PMC2758609

[B48] Umemoto A. Holroyd C. B. (2015). Task-specific effects of reward on task switching. *Psychol. Res.* 79 698–707. 10.1007/s00426-014-0595-z 24984832

[B49] von Rhein D. Beckmann C. F. Franke B. Oosterlaan J. Heslenfeld D. J. Hoekstra P. J. (2017). Network-level assessment of reward-related activation in patients with ADHD and healthy individuals. *Hum. Brain Mapp.* 38 2359–2369. 10.1002/hbm.23522 28176434 PMC6584954

[B50] von Rhein D. Cools R. Zwiers M. P. van der Schaaf M. Franke B. Luman M. (2015). Increased neural responses to reward in adolescents and young adults with attention-deficit/hyperactivity disorder and their unaffected siblings. *J. Am. Acad. Child Adolesc. Psychiatry* 54 394–402. 10.1016/j.jaac.2015.02.012 25901776 PMC4417499

[B51] Wilbertz G. Tebartz van Elst L. Delgado M. R. Maier S. Feige B. Philipsen A. (2012). Orbitofrontal reward sensitivity and impulsivity in adult attention deficit hyperactivity disorder. *Neuroimage* 60 353–361. 10.1016/j.neuroimage.2011.12.011 22197790

[B52] Willcutt E. G. Doyle A. E. Nigg J. T. Faraone S. V. Pennington B. F. (2005). Validity of the executive function theory of attention-deficit/hyperactivity disorder: a meta-analytic review. *Biol. Psychiatry* 57 1336–1346. 10.1016/j.biopsych.2005.02.006 15950006

[B53] Wodka E. L. Mahone E. M. Blankner J. G. Larson J. C. G. Fotedar S. Denckla M. B. (2007). Evidence that response inhibition is a primary deficit in ADHD. *J. Clin. Exp. Neuropsychol.* 29 345–356. 10.1080/13803390600678046 17497558

[B54] Wodushek T. R. Neumann C. S. (2003). Inhibitory capacity in adults with symptoms of attention deficit/hyperactivity disorder (ADHD). *Arch. Clin. Neuropsychol.* 18 317–330. 10.1016/S0887-6177(02)00152-X 14591462

[B55] Yee D. M. Krug M. K. Allen A. Z. Braver T. S. (2016). Humans integrate monetary and liquid incentives to motivate cognitive task performance. *Front. Psychol.* 6:2037. 10.3389/fpsyg.2015.02037 26834668 PMC4721208

[B56] Young M. E. Sutherland S. C. McCoy A. W. (2018). Optimal go/no-go ratios to maximize false alarms. *Behav. Res. Methods* 50 1020–1029. 10.3758/s13428-017-0923-5 28664243

[B57] Zhang Z. Mendelsohn A. Manson K. F. Schiller D. Levy I. (2016). Dissociating value representation and inhibition of inappropriate affective response during reversal learning in the ventromedial prefrontal cortex. *eNeuro* 2:ENEURO.72–15.2015. 10.1523/ENEURO.0072-15.2015 26730406 PMC4698540

