# Supplementary material for: Habit Expression and Disruption as a Function of Attention-Deficit/Hyperactivity Disorder Symptomology
Source: Front Psychol. 2019 Sep 3;10:1997. doi: 10.3389/fpsyg.2019.01997 (PMC6733985; doi:10.3389/fpsyg.2019.01997)
Supplement: Supplementary file 1 [file Data_Sheet_1.ZIP › ASRS_Data_Compile.html]

ASRS\_Data\_Compile


# Habit Demonstration and Disruption as a Function of ADHD Symptom Severity study¶

# Data compilation and plotting notebook¶

In [1]:

```
import pandas as pd
import matplotlib as mpl
import matplotlib.pyplot as plt
import seaborn as sns
import numpy as np
from scipy import stats
from matplotlib.patches import Patch
import statsmodels as sm
from statsmodels.stats.anova import AnovaRM
```

In [2]:

```
#Read Day 1 file and remove "." from column names, then rename the confusing edat column names
```

In [31]:

```
df=pd.read_csv("day1merge_full.csv")
df.columns=df.columns.str.replace('.','')
df.rename(columns={"SubTrial": "TrialNumber", "Procedure[SubTrial]": "Signal"}, inplace=True)
df.head()
```

Out[31]:

|  | ExperimentName | Subject | TrialNumber | Signal | totalpoints | totalpoints2 | GoBlueRevACC | GoBlueRev1ACC | GoGreenACC | GoGreen1ACC | ... | GoVioletRT | GoViolet1RT | NoGoBlueRT | NoGoBlue1RT | NoGoGreenRevRT | NoGoGreenRev1RT | NoGoRedRT | NoGoRed1RT | NoGoVioletRevRT | NoGoVioletRev1RT |
| --- | --- | --- | --- | --- | --- | --- | --- | --- | --- | --- | --- | --- | --- | --- | --- | --- | --- | --- | --- | --- | --- |
| 0 | FamCongFirst | 1 | 2 | XGo | 70.0 | NaN | NaN | NaN | 1.0 | NaN | ... | NaN | NaN | NaN | NaN | NaN | NaN | NaN | NaN | NaN | NaN |
| 1 | FamCongFirst | 1 | 3 | XGo | 70.0 | NaN | NaN | NaN | 1.0 | NaN | ... | NaN | NaN | NaN | NaN | NaN | NaN | NaN | NaN | NaN | NaN |
| 2 | FamCongFirst | 1 | 4 | XGo | 70.0 | NaN | NaN | NaN | 0.0 | NaN | ... | NaN | NaN | NaN | NaN | NaN | NaN | NaN | NaN | NaN | NaN |
| 3 | FamCongFirst | 1 | 5 | XGo | 70.0 | NaN | NaN | NaN | 1.0 | NaN | ... | NaN | NaN | NaN | NaN | NaN | NaN | NaN | NaN | NaN | NaN |
| 4 | FamCongFirst | 1 | 6 | XGo | 70.0 | NaN | NaN | NaN | 1.0 | NaN | ... | NaN | NaN | NaN | NaN | NaN | NaN | NaN | NaN | NaN | NaN |

5 rows × 38 columns

In [4]:

```
#merge the columns that refer to the same variable (GoGreenACC and GoGreen1ACC, for example)
```

In [5]:

```
df["GoGreen_ACC"] = df['GoGreenACC'].combine_first(df['GoGreen1ACC'])
df["GoRedRev_ACC"] = df['GoRedRevACC'].combine_first(df['GoRedRev1ACC'])
df["GoViolet_ACC"] = df['GoVioletACC'].combine_first(df['GoViolet1ACC'])
df["GoBlueRev_ACC"] = df['GoBlueRevACC'].combine_first(df['GoBlueRev1ACC'])
df["NoGoRed_ACC"] = df['NoGoRedACC'].combine_first(df['NoGoRed1ACC'])
df["NoGoGreenRev_ACC"] = df['NoGoGreenRevACC'].combine_first(df['NoGoGreenRev1ACC'])
df["NoGoBlue_ACC"] = df['NoGoBlueACC'].combine_first(df['NoGoBlue1ACC'])
df["NoGoVioletRev_ACC"] = df['NoGoVioletRevACC'].combine_first(df['NoGoVioletRev1ACC'])
df["GoGreen_RT"] = df['GoGreenRT'].combine_first(df['GoGreen1RT'])
df["GoRedRev_RT"] = df['GoRedRevRT'].combine_first(df['GoRedRev1RT'])
df["GoViolet_RT"] = df['GoVioletRT'].combine_first(df['GoViolet1RT'])
df["GoBlueRev_RT"] = df['GoBlueRevRT'].combine_first(df['GoBlueRev1RT'])
df["NoGoRed_RT"] = df['NoGoRedRT'].combine_first(df['NoGoRed1RT'])
df["NoGoGreenRev_RT"] = df['NoGoGreenRevRT'].combine_first(df['NoGoGreenRev1RT'])
df["NoGoBlue_RT"] = df['NoGoBlueRT'].combine_first(df['NoGoBlue1RT'])
df["NoGoVioletRev_RT"] = df['NoGoVioletRevRT'].combine_first(df['NoGoVioletRev1RT'])
concat_df=df.iloc[:, np.r_[0:6,38:len(df.columns)]]
```

In [32]:

```
#Now replace NaN cells with blanks, and replace the RT 0 values with blanks
df_clean=concat_df.replace(np.nan, '', regex=True)
replacezeros=df_clean.iloc[:,14:len(df_clean.columns)]
df_cleanedzeros=replacezeros.replace(0, "")
#apply zero-replaced version to main dataframe
df_clean.iloc[:,14:len(df_clean.columns)]=df_cleanedzeros
#save cleaned up Day 1 raw data as a csv file in case you want to repeat analyses in Excel with pivot tables
#df_clean.to_csv("Exp8Data_Day1_full.csv")
df_clean.head()
```

Out[32]:

|  | ExperimentName | Subject | TrialNumber | Signal | totalpoints | totalpoints2 | GoGreen\_ACC | GoRedRev\_ACC | GoViolet\_ACC | GoBlueRev\_ACC | ... | NoGoBlue\_ACC | NoGoVioletRev\_ACC | GoGreen\_RT | GoRedRev\_RT | GoViolet\_RT | GoBlueRev\_RT | NoGoRed\_RT | NoGoGreenRev\_RT | NoGoBlue\_RT | NoGoVioletRev\_RT |
| --- | --- | --- | --- | --- | --- | --- | --- | --- | --- | --- | --- | --- | --- | --- | --- | --- | --- | --- | --- | --- | --- |
| 0 | FamCongFirst | 1 | 2 | XGo | 70 |  | 1 |  |  |  | ... |  |  | 343 |  |  |  |  |  |  |  |
| 1 | FamCongFirst | 1 | 3 | XGo | 70 |  | 1 |  |  |  | ... |  |  | 372 |  |  |  |  |  |  |  |
| 2 | FamCongFirst | 1 | 4 | XGo | 70 |  | 0 |  |  |  | ... |  |  |  |  |  |  |  |  |  |  |
| 3 | FamCongFirst | 1 | 5 | XGo | 70 |  | 1 |  |  |  | ... |  |  | 349 |  |  |  |  |  |  |  |
| 4 | FamCongFirst | 1 | 6 | XGo | 70 |  | 1 |  |  |  | ... |  |  | 276 |  |  |  |  |  |  |  |

5 rows × 22 columns

In [7]:

```
#Now we need to "melt" the data so that it's suitable for plotting and stats. We'll group the data by "Subject" and "ExperimentName"
#Another perk to this approach is that instead of numerous columns for subject performance data, we can group by "Congruency" and "StimulusType"
#Set your identifying variables, value variables, and specify the columns they should go under, i.e., "DV" and "Accuracy"
#Next, make it so that each data column returns its associated category (Familiar, Novel, Congruent, Incongruent)
```

In [8]:

```
stacked_data=pd.melt(df_clean, id_vars=["Subject", "ExperimentName"], value_vars=["NoGoRed_ACC", "NoGoGreenRev_ACC", "NoGoBlue_ACC", "NoGoVioletRev_ACC"], 
        var_name="DV", value_name="Accuracy")
def conditions_phase(x):
    if x == "NoGoRed_ACC":
        return "Congruent"
    elif x == "NoGoGreenRev_ACC":
        return "Incongruent"
    elif x == "NoGoBlue_ACC":
        return "Congruent"
    elif x == "NoGoVioletRev_ACC":
        return "Incongruent"
func = np.vectorize(conditions_phase)
stacked_data["Congruency"] = func(stacked_data["DV"])
def StimCond(x):
    if x == "FamCongFirst":
        return "Familiar"
    elif x == "FamIncongFirst":
        return "Familiar"
    elif x == "NovCongFirst":
        return "Novel"
    elif x == "NovIncongFirst":
        return "Novel"
func = np.vectorize(StimCond)
stacked_data["StimulusType"] = func(stacked_data["ExperimentName"])
#add a column for Feedback group, since that will come in handy when we merge both days' dataframes
stacked_data["FeedbackCond"]="NoFeedback"
#stacked_data.to_csv("teststacked.csv")
#the dataframe is full of rows with empty Accuracy values because we're only listing NoGo trials and not Go trials.
#Let's make the Go rows invisible
stacked_data_truncated = stacked_data[stacked_data.Accuracy != '']
#stacked_data_truncated.to_csv("testtruncated.csv")
```

In [33]:

```
#We're ready to create our pivot table. Start by converting the Accuracy column into a numeric column so that it can be plotted
stacked_data_truncated['Accuracy'] = pd.to_numeric(stacked_data_truncated['Accuracy'])
#create the pivot table by assigning the columns for each factor, and identify the value, in this case "Accuracy"
piv_tab = pd.pivot_table(stacked_data_truncated, index=["Subject"], columns=["StimulusType", "Congruency"], values=["Accuracy"])
piv_tab.head()
```

```
C:\Users\ahmet\Anaconda3\lib\site-packages\ipykernel_launcher.py:2: SettingWithCopyWarning: 
A value is trying to be set on a copy of a slice from a DataFrame.
Try using .loc[row_indexer,col_indexer] = value instead

See the caveats in the documentation: http://pandas.pydata.org/pandas-docs/stable/indexing.html#indexing-view-versus-copy
```

Out[33]:

|  | Accuracy | | | |
| --- | --- | --- | --- | --- |
| StimulusType | Familiar | | Novel | |
| Congruency | Congruent | Incongruent | Congruent | Incongruent |
| Subject |  |  |  |  |
| 1 | 0.70 | 0.25 | 0.55 | 0.40 |
| 2 | 0.85 | 0.75 | 0.75 | 0.75 |
| 3 | 0.55 | 0.45 | 0.60 | 0.65 |
| 4 | 0.70 | 0.75 | 0.70 | 0.55 |
| 5 | 0.85 | 0.75 | 0.75 | 0.90 |

In [10]:

```
#Let's turn this into a plot. First, python gets mad when you refer to index names as plot axes, so make sure the grouping plots are not seen as indices
forplot = stacked_data_truncated.groupby(['StimulusType', "Congruency", "Subject"], as_index=False).Accuracy.mean()
#create the plot. I like using seaborn, but it could be done with matplotlib's pyplot. Pulling data from a pivot table has its downsides with regard to freedom in customizing seaborn plots, but it'll do for now.
ax=sns.barplot(x="StimulusType", y='Accuracy', hue="Congruency", palette=["#ff0000", "#03d547"], ci=68, capsize=0.01, data=forplot)
#notice that ci (confidence interval) is set to 68. That's because mathematically, the standard error of the mean is when you set 68% CI
#hue only takes 2 colors, so I manually add 2 more colors for novel condition using patches:
ax.patches[1].set_facecolor("#1d47f5")
ax.patches[3].set_facecolor("#d12fdf")
#fun fact: these hex codes are extracted from the actual color of the stimuli we use.
#remove unnecessary plot elements and make it pretty
ax.legend_.remove()
ax.set_xlabel("")
ax.set_ylabel("NoGo Accuracy")
plt.title("Familiar stimuli elicit incongruency-related \nimpairments in NoGo accuracy", weight="bold", y=1.08)
ax.set_ylim(0.5, 0.85)
sns.despine()
#add significance asterisk and line
x1, x2 = -0.20, 0.18   # only two columns, so they would be 0, 1
y, h, col = forplot['Accuracy'].mean()+0.14, 0.005, "k" #y will be the height of the line and star(2 points above the mean), h will be the height of the two lines
                                          #pointing down--0.2, and col is the color--black coded as k 
plt.plot([x1, x1, x2, x2], [y, y+h, y+h, y], lw=1.25, c=col) #here we plot this line and star on top of our barplot
plt.text((x1+x2)*0.5, y+h, "*", ha='center', va='bottom', color=col)
#plt.savefig("Exp8_NoGo_graph.tiff", bbox_inches="tight", dpi=500)
plt.show()
```

```
C:\Users\ahmet\Anaconda3\lib\site-packages\scipy\stats\stats.py:1713: FutureWarning: Using a non-tuple sequence for multidimensional indexing is deprecated; use `arr[tuple(seq)]` instead of `arr[seq]`. In the future this will be interpreted as an array index, `arr[np.array(seq)]`, which will result either in an error or a different result.
  return np.add.reduce(sorted[indexer] * weights, axis=axis) / sumval
```

In [34]:

```
#Repeat above for Go accuracy
#first melt with Go values
stacked_data_go=pd.melt(df_clean, id_vars=["Subject", "ExperimentName"], value_vars=["GoGreen_ACC", "GoRedRev_ACC", "GoViolet_ACC", "GoBlueRev_ACC"], 
        var_name="DV", value_name="Accuracy")
def conditions_phase_go(x):
    if x == "GoGreen_ACC":
        return "Congruent"
    elif x == "GoRedRev_ACC":
        return "Incongruent"
    elif x == "GoViolet_ACC":
        return "Congruent"
    elif x == "GoBlueRev_ACC":
        return "Incongruent"
func_go = np.vectorize(conditions_phase_go)
stacked_data_go["Congruency"] = func_go(stacked_data_go["DV"])
def StimCond_go(x):
    if x == "FamCongFirst":
        return "Familiar"
    elif x == "FamIncongFirst":
        return "Familiar"
    elif x == "NovCongFirst":
        return "Novel"
    elif x == "NovIncongFirst":
        return "Novel"
func_go = np.vectorize(StimCond_go)
stacked_data_go["StimulusType"] = func_go(stacked_data_go["ExperimentName"])
#add a column for Feedback group, since that will come in handy when we merge both days' dataframes
stacked_data_go["FeedbackCond"]="NoFeedback"
#the dataframe is full of rows with empty Accuracy values because we're only listing Go trials and not NoGo trials.
#Let's make the NoGo rows invisible
stacked_data_go_truncated = stacked_data_go[stacked_data_go.Accuracy != '']
stacked_data_go_truncated.head()
```

Out[34]:

|  | Subject | ExperimentName | DV | Accuracy | Congruency | StimulusType | FeedbackCond |
| --- | --- | --- | --- | --- | --- | --- | --- |
| 0 | 1 | FamCongFirst | GoGreen\_ACC | 1 | Congruent | Familiar | NoFeedback |
| 1 | 1 | FamCongFirst | GoGreen\_ACC | 1 | Congruent | Familiar | NoFeedback |
| 2 | 1 | FamCongFirst | GoGreen\_ACC | 0 | Congruent | Familiar | NoFeedback |
| 3 | 1 | FamCongFirst | GoGreen\_ACC | 1 | Congruent | Familiar | NoFeedback |
| 4 | 1 | FamCongFirst | GoGreen\_ACC | 1 | Congruent | Familiar | NoFeedback |

In [35]:

```
#turn DV into a numeric type
stacked_data_go_truncated['Accuracy'] = pd.to_numeric(stacked_data_go_truncated['Accuracy'])
#create the pivot table by assigning columns for each factor, and identify the value, in this case "Accuracy"
piv_tab_go = pd.pivot_table(stacked_data_go_truncated, index=["Subject"], columns=["StimulusType", "Congruency"], values=["Accuracy"])
piv_tab_go.head()
```

```
C:\Users\ahmet\Anaconda3\lib\site-packages\ipykernel_launcher.py:2: SettingWithCopyWarning: 
A value is trying to be set on a copy of a slice from a DataFrame.
Try using .loc[row_indexer,col_indexer] = value instead

See the caveats in the documentation: http://pandas.pydata.org/pandas-docs/stable/indexing.html#indexing-view-versus-copy
```

Out[35]:

|  | Accuracy | | | |
| --- | --- | --- | --- | --- |
| StimulusType | Familiar | | Novel | |
| Congruency | Congruent | Incongruent | Congruent | Incongruent |
| Subject |  |  |  |  |
| 1 | 0.81 | 0.77 | 0.77 | 0.83 |
| 2 | 0.97 | 0.96 | 0.95 | 1.00 |
| 3 | 0.96 | 0.93 | 0.93 | 0.91 |
| 4 | 0.92 | 0.95 | 0.92 | 0.91 |
| 5 | 0.97 | 0.95 | 0.93 | 0.95 |

In [13]:

```
forplot_go = stacked_data_go_truncated.groupby(["StimulusType", "Congruency", "Subject"], as_index=False).Accuracy.mean()
#create the plot. I like using seaborn, but it could be done with matplotlib's pyplot. Pulling data from a pivot table has its downsides with regard to freedom in customizing seaborn plots, but it'll do for now.
ax_go=sns.barplot(x="StimulusType", y='Accuracy', hue="Congruency", palette=["#03d547", "#ff0000"], ci=68, capsize=0.01, data=forplot_go)
#notice that ci (confidence interval) is set to 68. That's because mathematically, the standard error of the mean is when you set 68% CI
#hue only takes 2 colors, so I manually add 2 more colors for novel condition using patches:
ax_go.patches[1].set_facecolor("#d12fdf")
ax_go.patches[3].set_facecolor("#1d47f5")
#fun fact: these color codes are extracted from the actual color of the stimuli we use.
#remove unnecessary plot elements and make it pretty
ax_go.legend_.remove()
ax_go.set_xlabel("")
ax_go.set_ylabel("Go Accuracy")
plt.title("Familiar stimuli elicit incongruency-related \nimpairments in Go accuracy", weight="bold", y=1.08)
ax_go.set_ylim(0.5, 1)
sns.despine()
#add significance asterisk and line
x1, x2 = -0.20, 0.18   # only two columns, so they would be 0, 1
y, h, col = forplot_go['Accuracy'].mean()+0.1, 0.005, "k" #y will be the height of the line and star(2 points above the mean), h will be the height of the two lines
                                          #pointing down--0.2, and col is the color--black coded as k 
plt.plot([x1, x1, x2, x2], [y, y+h, y+h, y], lw=1.25, c=col) #here we plot this line and star on top of our barplot
plt.text((x1+x2)*0.5, y+h, "*", ha='center', va='bottom', color=col)
#plt.savefig("Exp8_Go_graph.tiff", bbox_inches="tight", dpi=500)
plt.show()
```

```
C:\Users\ahmet\Anaconda3\lib\site-packages\scipy\stats\stats.py:1713: FutureWarning: Using a non-tuple sequence for multidimensional indexing is deprecated; use `arr[tuple(seq)]` instead of `arr[seq]`. In the future this will be interpreted as an array index, `arr[np.array(seq)]`, which will result either in an error or a different result.
  return np.add.reduce(sorted[indexer] * weights, axis=axis) / sumval
```

In [36]:

```
#next, we read day 2 data, where participants receive the feedback manipulation
df_fb=pd.read_csv("day2merge_full.csv")
df_fb.columns=df_fb.columns.str.replace('.','')
df_fb.rename(columns={"SubTrial": "TrialNumber", "Procedure[SubTrial]": "Signal"}, inplace=True)
df_fb.head()
```

Out[36]:

|  | ExperimentName | Subject | LogLevel5 | Procedure[LogLevel5] | totalpoints | totalpoints2 | GoGreenACC | GoGreen1ACC | GoRedRevACC | GoRedRev1ACC | ... | NoGoRedACC | NoGoRed1ACC | GoGreenRT | GoGreen1RT | GoRedRevRT | GoRedRev1RT | NoGoGreenRevRT | NoGoGreenRev1RT | NoGoRedRT | NoGoRed1RT |
| --- | --- | --- | --- | --- | --- | --- | --- | --- | --- | --- | --- | --- | --- | --- | --- | --- | --- | --- | --- | --- | --- |
| 0 | Day2\_FamFB | 1 | 1 | XGo | 80 | 35.0 | 1.0 | NaN | NaN | NaN | ... | NaN | NaN | 270.0 | NaN | NaN | NaN | NaN | NaN | NaN | NaN |
| 1 | Day2\_FamFB | 1 | 2 | XGo | 80 | 35.0 | 1.0 | NaN | NaN | NaN | ... | NaN | NaN | 296.0 | NaN | NaN | NaN | NaN | NaN | NaN | NaN |
| 2 | Day2\_FamFB | 1 | 3 | XGo | 80 | 35.0 | 1.0 | NaN | NaN | NaN | ... | NaN | NaN | 296.0 | NaN | NaN | NaN | NaN | NaN | NaN | NaN |
| 3 | Day2\_FamFB | 1 | 4 | XGo | 80 | 35.0 | 1.0 | NaN | NaN | NaN | ... | NaN | NaN | 272.0 | NaN | NaN | NaN | NaN | NaN | NaN | NaN |
| 4 | Day2\_FamFB | 1 | 5 | XGo | 80 | 35.0 | 1.0 | NaN | NaN | NaN | ... | NaN | NaN | 338.0 | NaN | NaN | NaN | NaN | NaN | NaN | NaN |

5 rows × 22 columns

In [37]:

```
#Redo data cleanup for the day 2 datasheet. remember, familiar stim only. adjust length of column accordingly!
df_fb["GoGreen_ACC"] = df_fb['GoGreenACC'].combine_first(df_fb['GoGreen1ACC'])
df_fb["GoRedRev_ACC"] = df_fb['GoRedRevACC'].combine_first(df_fb['GoRedRev1ACC'])
df_fb["NoGoRed_ACC"] = df_fb['NoGoRedACC'].combine_first(df_fb['NoGoRed1ACC'])
df_fb["NoGoGreenRev_ACC"] = df_fb['NoGoGreenRevACC'].combine_first(df_fb['NoGoGreenRev1ACC'])
df_fb["GoGreen_RT"] = df_fb['GoGreenRT'].combine_first(df_fb['GoGreen1RT'])
df_fb["GoRedRev_RT"] = df_fb['GoRedRevRT'].combine_first(df_fb['GoRedRev1RT'])
df_fb["NoGoRed_RT"] = df_fb['NoGoRedRT'].combine_first(df_fb['NoGoRed1RT'])
df_fb["NoGoGreenRev_RT"] = df_fb['NoGoGreenRevRT'].combine_first(df_fb['NoGoGreenRev1RT'])
concat_df_fb=df_fb.iloc[:, np.r_[0:6,22:len(df_fb.columns)]]
concat_df_fb
#Now replace NaN cells with blanks, and replace the RT 0 values with blanks
df_fb_clean=concat_df_fb.replace(np.nan, '', regex=True)
replacezeros_fb=df_fb_clean.iloc[:,10:len(df_fb_clean.columns)]
df_fb_cleanedzeros=replacezeros_fb.replace(0, "")
#apply zero-replaced version to main dataframe
df_fb_clean.iloc[:,10:len(df_fb_clean.columns)]=df_fb_cleanedzeros
#save cleaned up Day 1 raw data as a csv file in case you want to repeat analyses in Excel with pivot tables
#df_fb_clean.to_csv("Exp8Data_Day2.csv")
#melt the fb data
stacked_data_fb=pd.melt(df_fb_clean, id_vars=["Subject", "ExperimentName"], value_vars=["NoGoRed_ACC", "NoGoGreenRev_ACC"], 
        var_name="DV", value_name="Accuracy")
def conditions_phase_fb(x):
    if x == "NoGoRed_ACC":
        return "Congruent"
    elif x == "NoGoGreenRev_ACC":
        return "Incongruent"
func_fb = np.vectorize(conditions_phase_fb)
stacked_data_fb["Congruency"] = func_fb(stacked_data_fb["DV"])
def StimCond_fb(x):
    if x == "Day2_FamFB":
        return "Familiar"
func_fb = np.vectorize(StimCond_fb)
stacked_data_fb["StimulusType"] = func_fb(stacked_data_fb["ExperimentName"])
#add a column for Feedback group
stacked_data_fb["FeedbackCond"]="Feedback"
#the dataframe is full of rows with empty Accuracy values because we're only listing NoGo trials and not Go trials.
#Let's make the Go rows invisible
stacked_data_fb_truncated = stacked_data_fb[stacked_data_fb.Accuracy != '']
stacked_data_fb_truncated.head()
```

Out[37]:

|  | Subject | ExperimentName | DV | Accuracy | Congruency | StimulusType | FeedbackCond |
| --- | --- | --- | --- | --- | --- | --- | --- |
| 6 | 1 | Day2\_FamFB | NoGoRed\_ACC | 1 | Congruent | Familiar | Feedback |
| 9 | 1 | Day2\_FamFB | NoGoRed\_ACC | 1 | Congruent | Familiar | Feedback |
| 15 | 1 | Day2\_FamFB | NoGoRed\_ACC | 0 | Congruent | Familiar | Feedback |
| 18 | 1 | Day2\_FamFB | NoGoRed\_ACC | 1 | Congruent | Familiar | Feedback |
| 27 | 1 | Day2\_FamFB | NoGoRed\_ACC | 1 | Congruent | Familiar | Feedback |

In [38]:

```
#turn DV into a numeric type
stacked_data_fb_truncated['Accuracy'] = pd.to_numeric(stacked_data_fb_truncated['Accuracy'])
#create the pivot table by assigning the columns for each factor, and identify the value, in this case "Accuracy"
piv_tab_fb = pd.pivot_table(stacked_data_fb_truncated, index=["Subject"], columns=["StimulusType", "Congruency"], values=["Accuracy"])
piv_tab_fb.head()
```

```
C:\Users\ahmet\Anaconda3\lib\site-packages\ipykernel_launcher.py:2: SettingWithCopyWarning: 
A value is trying to be set on a copy of a slice from a DataFrame.
Try using .loc[row_indexer,col_indexer] = value instead

See the caveats in the documentation: http://pandas.pydata.org/pandas-docs/stable/indexing.html#indexing-view-versus-copy
```

Out[38]:

|  | Accuracy | |
| --- | --- | --- |
| StimulusType | Familiar | |
| Congruency | Congruent | Incongruent |
| Subject |  |  |
| 1 | 0.80 | 0.35 |
| 2 | 0.90 | 0.85 |
| 3 | 0.55 | 0.70 |
| 4 | 0.80 | 0.50 |
| 5 | 0.75 | 0.55 |

In [17]:

```
#A simple day 2 familiar stim graph would be nice. we'll do the side by side with day 1 data later.
forplot_fb = stacked_data_fb_truncated.groupby(["Congruency", "Subject"], as_index=False).Accuracy.mean()
#create the plot. I like using seaborn, but it could be done with matplotlib's pyplot. Pulling data from a pivot table has its downsides with regard to freedom in customizing seaborn plots, but it'll do for now.
ax_fb=sns.barplot(x="Congruency", y='Accuracy', palette=["#ff0000", "#03d547"], ci=68, capsize=0.01, data=forplot_fb)
ax_fb.set_xlabel("")
ax_fb.set_ylabel("NoGo Accuracy")
plt.title("Feedback prevents the congruency-related \nimpairments in NoGo accuracy", weight="bold", y=1.08)
ax_fb.set_ylim(0.5, 0.85)
sns.despine()
```

```
C:\Users\ahmet\Anaconda3\lib\site-packages\scipy\stats\stats.py:1713: FutureWarning: Using a non-tuple sequence for multidimensional indexing is deprecated; use `arr[tuple(seq)]` instead of `arr[seq]`. In the future this will be interpreted as an array index, `arr[np.array(seq)]`, which will result either in an error or a different result.
  return np.add.reduce(sorted[indexer] * weights, axis=axis) / sumval
```

In [39]:

```
#Go accuracy from the day 2 data
#melt the day 2 go data
stacked_data_fb_go=pd.melt(df_fb_clean, id_vars=["Subject", "ExperimentName"], value_vars=["GoGreen_ACC", "GoRedRev_ACC"], 
        var_name="DV", value_name="Accuracy")
def conditions_phase_fb_go(x):
    if x == "GoGreen_ACC":
        return "Congruent"
    elif x == "GoRedRev_ACC":
        return "Incongruent"
func_fb_go = np.vectorize(conditions_phase_fb_go)
stacked_data_fb_go["Congruency"] = func_fb_go(stacked_data_fb_go["DV"])
def StimCond_fb_go(x):
    if x == "Day2_FamFB":
        return "Familiar"
func_fb_go = np.vectorize(StimCond_fb_go)
stacked_data_fb_go["StimulusType"] = func_fb_go(stacked_data_fb_go["ExperimentName"])
#add a column for Feedback group
stacked_data_fb_go["FeedbackCond"]="Feedback"
#the dataframe is full of rows with empty Accuracy values because we're only listing Go trials and not NoGo trials.
#make the NoGo rows invisible
stacked_data_fb_go_truncated = stacked_data_fb_go[stacked_data_fb_go.Accuracy != '']
stacked_data_fb_go_truncated.head()
```

Out[39]:

|  | Subject | ExperimentName | DV | Accuracy | Congruency | StimulusType | FeedbackCond |
| --- | --- | --- | --- | --- | --- | --- | --- |
| 0 | 1 | Day2\_FamFB | GoGreen\_ACC | 1 | Congruent | Familiar | Feedback |
| 1 | 1 | Day2\_FamFB | GoGreen\_ACC | 1 | Congruent | Familiar | Feedback |
| 2 | 1 | Day2\_FamFB | GoGreen\_ACC | 1 | Congruent | Familiar | Feedback |
| 3 | 1 | Day2\_FamFB | GoGreen\_ACC | 1 | Congruent | Familiar | Feedback |
| 4 | 1 | Day2\_FamFB | GoGreen\_ACC | 1 | Congruent | Familiar | Feedback |

In [40]:

```
#turn DV into a numeric type
stacked_data_fb_go_truncated['Accuracy'] = pd.to_numeric(stacked_data_fb_go_truncated['Accuracy'])
#create the pivot table by assigning columns for each factor, and identify the value, in this case "Accuracy"
piv_tab_fb_go = pd.pivot_table(stacked_data_fb_go_truncated, index=["Subject"], columns=["StimulusType", "Congruency"], values=["Accuracy"])
piv_tab_fb_go.head()
```

```
C:\Users\ahmet\Anaconda3\lib\site-packages\ipykernel_launcher.py:2: SettingWithCopyWarning: 
A value is trying to be set on a copy of a slice from a DataFrame.
Try using .loc[row_indexer,col_indexer] = value instead

See the caveats in the documentation: http://pandas.pydata.org/pandas-docs/stable/indexing.html#indexing-view-versus-copy
```

Out[40]:

|  | Accuracy | |
| --- | --- | --- |
| StimulusType | Familiar | |
| Congruency | Congruent | Incongruent |
| Subject |  |  |
| 1 | 0.92 | 0.88 |
| 2 | 0.98 | 0.99 |
| 3 | 0.99 | 0.94 |
| 4 | 0.89 | 0.98 |
| 5 | 1.00 | 0.96 |

In [20]:

```
#And a graph for fb go accuracy. again, will add side by side with day 1 later
forplot_fb_go = stacked_data_fb_go_truncated.groupby(["Congruency", "Subject"], as_index=False).Accuracy.mean()
#create the plot. I like using seaborn, but it could be done with matplotlib's pyplot. Pulling data from a pivot table has its downsides with regard to freedom in customizing seaborn plots, but it'll do for now.
ax_fb_go=sns.barplot(x="Congruency", y='Accuracy', palette=["#ff0000", "#03d547"], ci=68, capsize=0.01, data=forplot_fb_go)
ax_fb_go.set_xlabel("")
ax_fb_go.set_ylabel("Go Accuracy")
plt.title("Feedback prevents the congruency-related \nimpairments in Go accuracy", weight="bold", y=1.08)
ax_fb_go.set_ylim(0.5, 1)
sns.despine()
```

```
C:\Users\ahmet\Anaconda3\lib\site-packages\scipy\stats\stats.py:1713: FutureWarning: Using a non-tuple sequence for multidimensional indexing is deprecated; use `arr[tuple(seq)]` instead of `arr[seq]`. In the future this will be interpreted as an array index, `arr[np.array(seq)]`, which will result either in an error or a different result.
  return np.add.reduce(sorted[indexer] * weights, axis=axis) / sumval
```

In [21]:

```
#So far we created pivot tables for NoGo and Go accuracy in both day 1 and day 2 spreadsheets, and plotted them
#Now depict how Feedback actually compares to NoFeedback.
```

In [22]:

```
#We'll need to concatenate the day 1 and 2 dataframes. 
#create one dataframe for Go and another for NoGo data
df_clean_all_nogo = pd.concat([stacked_data_truncated, stacked_data_fb_truncated])
#df_clean_all_nogo.to_csv("Exp8Data_All_NoGo.csv")

df_clean_all_go = pd.concat([stacked_data_go_truncated, stacked_data_fb_go_truncated])
#df_clean_all_go.to_csv("Exp8Data_All_Go.csv")
```

In [41]:

```
#first pivot table will extract data from all nogo information
piv_tab_nogo_all = pd.pivot_table(df_clean_all_nogo, index=["Subject"], columns=["FeedbackCond", "StimulusType", "Congruency"], values=["Accuracy"])
piv_tab_nogo_all.head()
```

Out[41]:

|  | Accuracy | | | | | |
| --- | --- | --- | --- | --- | --- | --- |
| FeedbackCond | Feedback | | NoFeedback | | | |
| StimulusType | Familiar | | Familiar | | Novel | |
| Congruency | Congruent | Incongruent | Congruent | Incongruent | Congruent | Incongruent |
| Subject |  |  |  |  |  |  |
| 1 | 0.80 | 0.35 | 0.70 | 0.25 | 0.55 | 0.40 |
| 2 | 0.90 | 0.85 | 0.85 | 0.75 | 0.75 | 0.75 |
| 3 | 0.55 | 0.70 | 0.55 | 0.45 | 0.60 | 0.65 |
| 4 | 0.80 | 0.50 | 0.70 | 0.75 | 0.70 | 0.55 |
| 5 | 0.75 | 0.55 | 0.85 | 0.75 | 0.75 | 0.90 |

In [24]:

```
#we can now plot our omnibus nogo data
```

In [25]:

```
forplot_all_nogo = df_clean_all_nogo.groupby(["StimulusType", "Congruency", "FeedbackCond", "Subject"], as_index=False).Accuracy.mean()
#create the plot. I like using seaborn, but it could be done with matplotlib's pyplot. Pulling data from a pivot table has its downsides with regard to freedom in customizing seaborn plots, but it'll do for now.
ax_all_nogo=sns.barplot(x="FeedbackCond", y='Accuracy', hue="Congruency", palette=["#ff0000", "#03d547"], ci=68, capsize=0.01, data=forplot_all_nogo[forplot_all_nogo.StimulusType=="Familiar"])
ax_all_nogo.legend_.remove()
ax_all_nogo.set_xlabel("")
ax_all_nogo.set_ylabel("NoGo Accuracy")
plt.title("Feedback prevents incongruency-related impairments \nin NoGo accuracy", weight="bold", y=1.1)
ax_all_nogo.set_ylim(0.5, 0.85)
sns.despine()
#add significance asterisk and line
x2, x3 = 0.8, 1.2
y, h, col = forplot_all_nogo[forplot_all_nogo.StimulusType=="Familiar"]["Accuracy"].mean()+0.13, 0.01, "k"
plt.plot([x2, x2, x3, x3], [y, y+h, y+h, y], lw=1, c=col)
plt.text((x2+x3)*0.5, y+h, "*", ha="center", va="bottom", color=col)
#plt.savefig("Exp8_FB_NoGo_graph.tiff", bbox_inches="tight", dpi=500)
plt.show()
```

```
C:\Users\ahmet\Anaconda3\lib\site-packages\scipy\stats\stats.py:1713: FutureWarning: Using a non-tuple sequence for multidimensional indexing is deprecated; use `arr[tuple(seq)]` instead of `arr[seq]`. In the future this will be interpreted as an array index, `arr[np.array(seq)]`, which will result either in an error or a different result.
  return np.add.reduce(sorted[indexer] * weights, axis=axis) / sumval
```

In [42]:

```
#same sequence of events for Go accuracy across feedback conditions
#this pivot table will extract go information
piv_tab_go_all = pd.pivot_table(df_clean_all_go, index=["Subject"], columns=["FeedbackCond", "StimulusType", "Congruency"], values=["Accuracy"])
piv_tab_go_all.head()
```

Out[42]:

|  | Accuracy | | | | | |
| --- | --- | --- | --- | --- | --- | --- |
| FeedbackCond | Feedback | | NoFeedback | | | |
| StimulusType | Familiar | | Familiar | | Novel | |
| Congruency | Congruent | Incongruent | Congruent | Incongruent | Congruent | Incongruent |
| Subject |  |  |  |  |  |  |
| 1 | 0.92 | 0.88 | 0.81 | 0.77 | 0.77 | 0.83 |
| 2 | 0.98 | 0.99 | 0.97 | 0.96 | 0.95 | 1.00 |
| 3 | 0.99 | 0.94 | 0.96 | 0.93 | 0.93 | 0.91 |
| 4 | 0.89 | 0.98 | 0.92 | 0.95 | 0.92 | 0.91 |
| 5 | 1.00 | 0.96 | 0.97 | 0.95 | 0.93 | 0.95 |

In [27]:

```
forplot_all_go = df_clean_all_go.groupby(["StimulusType", "Congruency", "FeedbackCond", "Subject"], as_index=False).Accuracy.mean()
#make sure to only plot within the "Familiar" stimulus set, since there's no novel condition with feedback.
ax_all_go=sns.barplot(x="FeedbackCond", y='Accuracy', hue="Congruency", palette=["#03d547", "#ff0000"], ci=68, capsize=0.01, data=forplot_all_go[forplot_all_go.StimulusType=="Familiar"])
ax_all_go.legend_.remove()
ax_all_go.set_xlabel("")
ax_all_go.set_ylabel("Go Accuracy")
plt.title("Feedback prevents incongruency-related \nimpairments in Go accuracy", weight="bold", y=1.1)
ax_all_go.set_ylim(0.5, 1)
sns.despine()
#add significance asterisk and line
x2, x3 = 0.8, 1.2
y, h, col = forplot_all_go[forplot_all_go.StimulusType=="Familiar"]["Accuracy"].mean()+0.07, 0.01, "k"
plt.plot([x2, x2, x3, x3], [y, y+h, y+h, y], lw=1, c=col)
plt.text((x2+x3)*0.5, y+h, "*", ha="center", va="bottom", color=col)
#plt.savefig("Exp8_FB_Go_graph.tiff", bbox_inches="tight", dpi=500)
plt.show()
```

```
C:\Users\ahmet\Anaconda3\lib\site-packages\scipy\stats\stats.py:1713: FutureWarning: Using a non-tuple sequence for multidimensional indexing is deprecated; use `arr[tuple(seq)]` instead of `arr[seq]`. In the future this will be interpreted as an array index, `arr[np.array(seq)]`, which will result either in an error or a different result.
  return np.add.reduce(sorted[indexer] * weights, axis=axis) / sumval
```

In [28]:

```
#create R friendly dataframes for posthoc t-tests to accompany the regressions
```

In [29]:

```
for_nogo_stats = df_clean_all_nogo.groupby(["StimulusType", "Congruency", "FeedbackCond", "Subject"], as_index=False).Accuracy.mean()
#for_nogo_stats.to_csv("nogostatssheet_full.csv")
```

In [30]:

```
for_go_stats = df_clean_all_go.groupby(["StimulusType", "Congruency", "FeedbackCond", "Subject"], as_index=False).Accuracy.mean()
#for_go_stats.to_csv("gostatssheet_full.csv")
```
