# Supplementary material for: Habit Expression and Disruption as a Function of Attention-Deficit/Hyperactivity Disorder Symptomology
Source: Front Psychol. 2019 Sep 3;10:1997. doi: 10.3389/fpsyg.2019.01997 (PMC6733985; doi:10.3389/fpsyg.2019.01997)
Supplement: Supplementary file 1 [file Data_Sheet_1.ZIP › ASRS_Data_R_Share_Supplement.html]

ASRS\_Data\_R\_Share\_Supplement


# Habit Demonstration and Disruption as a Function of ADHD Symptom Severity study¶

# Supplemental analysis notebook (as pre-registered)¶

### Analyses included here violate assumptions of independence. See main text analysis notebook for adjusted regressions¶

In [1]:

```
library(nlme)
#install.packages("MuMIn")
library(MuMIn)
#install.packages("reghelper")
library(reghelper)
#install.packages("car")
library(car)
#install.packages("stats")
library(stats)
#install.packages("lsr")
library(lsr)
#install.packages("psych")
library(psych)
#install.packages("devtools")
library(devtools)
#install_github("easyGgplot2", "kassambara")
library(easyGgplot2)
#install.packages("lme4")
library(lme4)
```

```
Warning message:
"package 'MuMIn' was built under R version 3.5.2"Warning message:
"package 'reghelper' was built under R version 3.5.2"
Attaching package: 'reghelper'

The following object is masked from 'package:base':

    beta

Warning message:
"package 'car' was built under R version 3.5.2"Loading required package: carData
Warning message:
"package 'carData' was built under R version 3.5.2"Warning message:
"package 'lsr' was built under R version 3.5.2"
Attaching package: 'psych'

The following object is masked from 'package:car':

    logit

The following object is masked from 'package:reghelper':

    ICC

Loading required package: ggplot2

Attaching package: 'ggplot2'

The following objects are masked from 'package:psych':

    %+%, alpha

Warning message:
"package 'lme4' was built under R version 3.5.2"Loading required package: Matrix

Attaching package: 'lme4'

The following object is masked from 'package:nlme':

    lmList
```

In [2]:

```
#Read csv with NoGo information (Days 1 and 2)
myNoGoData <- read.csv('Exp8_NoGo_Full.csv')
```

## First set of analyses will involve habit expression and ADHD symptomology. We need Day 1 data (as in, the data where participants receive no motivation enhancement)¶

In [3]:

```
#Take subset of dataframe to analyze Day 1 data (Familiar and Novel NoFeedback)
myNoGoDay1Data <- subset(myNoGoData, FeedbackCond=="NoFeedback")
```

### Run a linear mixed model in a hierarchical structure. We will determine whether each additional step of the model significantly contributes to predicting outcome-sensitivity¶

In [4]:

```
#Use ML instead of REML becuase we're concerned with comparing fixed effects between models.
nogo_nofb_model1 <- lme(Acc_Diff ~ Age + Gender + Cong_Order + Stim_Order + Drive, random=~1|Subject, method="ML", data=myNoGoDay1Data)
nogo_nofb_model2 <- lme(Acc_Diff ~ Age + Gender + Cong_Order + Stim_Order + Drive + ASRS_A + ASRS_B + ASRS_Total + Diagnosis + COHS, random=~1|Subject, method="ML", data=myNoGoDay1Data)
nogo_nofb_model3 <- lme(Acc_Diff ~ Age + Gender + Cong_Order + Stim_Order + Drive + ASRS_A + ASRS_B + ASRS_Total + Diagnosis + COHS + StimulusType, random=~1|Subject, method="ML", data=myNoGoDay1Data)
nogo_nofb_model4 <- lme(Acc_Diff ~ Age + Gender + Cong_Order + Stim_Order + Drive + ASRS_A + ASRS_B + ASRS_Total + Diagnosis + COHS + StimulusType + ASRS_A*StimulusType + ASRS_B*StimulusType + ASRS_Total*StimulusType + Diagnosis*StimulusType + COHS*StimulusType, random=~1|Subject, method="ML", data=myNoGoDay1Data)
```

In [5]:

```
#Check for outliers, beyond -3.3<x<3.3. No output means no outliers.
which(abs(residuals(nogo_nofb_model1, type="normalized")) > 3.3)
which(abs(residuals(nogo_nofb_model2, type="normalized")) > 3.3)
which(abs(residuals(nogo_nofb_model3, type="normalized")) > 3.3)
which(abs(residuals(nogo_nofb_model4, type="normalized")) > 3.3)
```

In [6]:

```
#Diagnostics. plot() checks for homoscedasticity violation, qqplot() checks for normality, vif() checks for multicollinearity
qqnorm(resid(nogo_nofb_model1))
qqnorm(resid(nogo_nofb_model2))
qqnorm(resid(nogo_nofb_model3))
qqnorm(resid(nogo_nofb_model4))
plot(nogo_nofb_model1)
plot(nogo_nofb_model2)
plot(nogo_nofb_model3)
plot(nogo_nofb_model4)
```

In [7]:

```
vif(nogo_nofb_model1)
vif(nogo_nofb_model2)
vif(nogo_nofb_model3)
vif(nogo_nofb_model4)
```

Age
:   6.76773370364327

Gender
:   1.10278256254609

Cong\_Order
:   3.97919186192501

Stim\_Order
:   4.11103980111859

Drive
:   6.71293269748102

Age
:   7.21191297797032

Gender
:   1.20958269153334

Cong\_Order
:   4.21013485029772

Stim\_Order
:   4.36358183746819

Drive
:   6.98750136278286

ASRS\_A
:   8.99829578776257

ASRS\_B
:   8.74379792221155

ASRS\_Total
:   23.5340071905796

Diagnosis
:   1.32905065526235

COHS
:   1.07951043474563

Age
:   7.21191297797037

Gender
:   1.20958269153334

Cong\_Order
:   4.21013485029776

Stim\_Order
:   4.36358183746824

Drive
:   6.98750136278285

ASRS\_A
:   8.99829578776264

ASRS\_B
:   8.74379792221164

ASRS\_Total
:   23.5340071905799

Diagnosis
:   1.32905065526235

COHS
:   1.07951043474563

StimulusType
:   1

Age
:   7.21191297797028

Gender
:   1.20958269153333

Cong\_Order
:   4.21013485029767

Stim\_Order
:   4.36358183746796

Drive
:   6.98750136278289

ASRS\_A
:   17.5573961430902

ASRS\_B
:   17.2239515086857

ASRS\_Total
:   46.3105936934366

Diagnosis
:   2.376908533832

COHS
:   2.13331112869411

StimulusType
:   64.9691076182565

ASRS\_A:StimulusType
:   91.1111347176646

ASRS\_B:StimulusType
:   71.6557128518684

ASRS\_Total:StimulusType
:   265.779061643565

Diagnosis:StimulusType
:   2.15987032249533

COHS:StimulusType
:   57.3687507995524

In [8]:

```
#Use beta from reghelper, otherwise beta coefs won't be standardized
beta(nogo_nofb_model1)
beta(nogo_nofb_model2)
beta(nogo_nofb_model3)
beta(nogo_nofb_model4)
```

```
Linear mixed-effects model fit by maximum likelihood
 Data: data 
       AIC      BIC    logLik
  595.4846 622.1849 -289.7423

Random effects:
 Formula: ~1 | Subject
         (Intercept)  Residual
StdDev: 4.700403e-05 0.9743869

Fixed effects: Acc_Diff.z ~ Age.z + Gender.z + Cong_Order.z + Stim_Order.z +      Drive.z 
                  Value  Std.Error  DF   t-value p-value
(Intercept)   0.0000000 0.06855762 104  0.000000  1.0000
Age.z        -0.2532351 0.17878204  98 -1.416446  0.1598
Gender.z     -0.1073427 0.07216842  98 -1.487391  0.1401
Cong_Order.z  0.1745247 0.13708808  98  1.273084  0.2060
Stim_Order.z -0.2128891 0.13934073  98 -1.527831  0.1298
Drive.z       0.3186428 0.17805673  98  1.789557  0.0766
 Correlation: 
             (Intr) Age.z  Gndr.z Cng_O. Stm_O.
Age.z         0.000                            
Gender.z      0.000 -0.206                     
Cong_Order.z  0.000 -0.082  0.129              
Stim_Order.z  0.000  0.096 -0.212 -0.863       
Drive.z       0.000 -0.922  0.178  0.090 -0.114

Standardized Within-Group Residuals:
        Min          Q1         Med          Q3         Max 
-2.86476099 -0.61500983  0.04413854  0.66880549  2.59487867 

Number of Observations: 208
Number of Groups: 104
```

```
Linear mixed-effects model fit by maximum likelihood
 Data: data 
       AIC      BIC    logLik
  604.5982 647.9862 -289.2991

Random effects:
 Formula: ~1 | Subject
         (Intercept)  Residual
StdDev: 5.855382e-05 0.9723131

Fixed effects: Acc_Diff.z ~ Age.z + Gender.z + Cong_Order.z + Stim_Order.z +      Drive.z + ASRS_A.z + ASRS_B.z + ASRS_Total.z + Diagnosis.z +      COHS.z 
                  Value Std.Error  DF    t-value p-value
(Intercept)   0.0000000 0.0692744 104  0.0000000  1.0000
Age.z        -0.2725880 0.1864854  93 -1.4617124  0.1472
Gender.z     -0.0943841 0.0763725  93 -1.2358377  0.2196
Cong_Order.z  0.1766069 0.1424845  93  1.2394818  0.2183
Stim_Order.z -0.2208710 0.1450578  93 -1.5226411  0.1312
Drive.z       0.3283236 0.1835610  93  1.7886345  0.0769
ASRS_A.z     -0.0470717 0.2083050  93 -0.2259751  0.8217
ASRS_B.z      0.0169484 0.2053381  93  0.0825388  0.9344
ASRS_Total.z  0.0352486 0.3368740  93  0.1046343  0.9169
Diagnosis.z   0.0318194 0.0800553  93  0.3974681  0.6919
COHS.z       -0.0556694 0.0721494  93 -0.7715842  0.4423
 Correlation: 
             (Intr) Age.z  Gndr.z Cng_O. Stm_O. Driv.z ASRS_A ASRS_B ASRS_T
Age.z         0.000                                                        
Gender.z      0.000 -0.224                                                 
Cong_Order.z  0.000 -0.094  0.163                                          
Stim_Order.z  0.000  0.103 -0.256 -0.864                                   
Drive.z       0.000 -0.902  0.157  0.070 -0.094                            
ASRS_A.z      0.000  0.048 -0.135  0.055  0.045 -0.046                     
ASRS_B.z      0.000  0.126 -0.015  0.082 -0.055 -0.086  0.692              
ASRS_Total.z  0.000 -0.069  0.051 -0.116  0.045  0.068 -0.901 -0.895       
Diagnosis.z   0.000 -0.078  0.216  0.113 -0.115 -0.110 -0.055  0.062 -0.020
COHS.z        0.000  0.124 -0.078  0.015  0.006 -0.138  0.077 -0.075 -0.014
             Dgnss.
Age.z              
Gender.z           
Cong_Order.z       
Stim_Order.z       
Drive.z            
ASRS_A.z           
ASRS_B.z           
ASRS_Total.z       
Diagnosis.z        
COHS.z        0.056

Standardized Within-Group Residuals:
        Min          Q1         Med          Q3         Max 
-2.74605833 -0.63187614  0.02859003  0.70225267  2.57491418 

Number of Observations: 208
Number of Groups: 104
```

```
Linear mixed-effects model fit by maximum likelihood
 Data: data 
       AIC      BIC    logLik
  584.6008 631.3264 -278.3004

Random effects:
 Formula: ~1 | Subject
         (Intercept)  Residual
StdDev: 7.690086e-05 0.9222345

Fixed effects: Acc_Diff.z ~ Age.z + Gender.z + Cong_Order.z + Stim_Order.z +      Drive.z + ASRS_A.z + ASRS_B.z + ASRS_Total.z + Diagnosis.z +      COHS.z + StimulusTypeNovel.z 
                         Value Std.Error  DF   t-value p-value
(Intercept)          0.0000000 0.0658739 103  0.000000  1.0000
Age.z               -0.2725880 0.1773312  93 -1.537169  0.1276
Gender.z            -0.0943841 0.0726236  93 -1.299634  0.1969
Cong_Order.z         0.1766069 0.1354902  93  1.303466  0.1956
Stim_Order.z        -0.2208710 0.1379372  93 -1.601243  0.1127
Drive.z              0.3283236 0.1745504  93  1.880968  0.0631
ASRS_A.z            -0.0470717 0.1980797  93 -0.237640  0.8127
ASRS_B.z             0.0169484 0.1952585  93  0.086800  0.9310
ASRS_Total.z         0.0352486 0.3203375  93  0.110036  0.9126
Diagnosis.z          0.0318194 0.0761256  93  0.417986  0.6769
COHS.z              -0.0556694 0.0686078  93 -0.811415  0.4192
StimulusTypeNovel.z  0.3087631 0.0660328 103  4.675904  0.0000
 Correlation: 
                    (Intr) Age.z  Gndr.z Cng_O. Stm_O. Driv.z ASRS_A ASRS_B
Age.z                0.000                                                 
Gender.z             0.000 -0.224                                          
Cong_Order.z         0.000 -0.094  0.163                                   
Stim_Order.z         0.000  0.103 -0.256 -0.864                            
Drive.z              0.000 -0.902  0.157  0.070 -0.094                     
ASRS_A.z             0.000  0.048 -0.135  0.055  0.045 -0.046              
ASRS_B.z             0.000  0.126 -0.015  0.082 -0.055 -0.086  0.692       
ASRS_Total.z         0.000 -0.069  0.051 -0.116  0.045  0.068 -0.901 -0.895
Diagnosis.z          0.000 -0.078  0.216  0.113 -0.115 -0.110 -0.055  0.062
COHS.z               0.000  0.124 -0.078  0.015  0.006 -0.138  0.077 -0.075
StimulusTypeNovel.z  0.000  0.000  0.000  0.000  0.000  0.000  0.000  0.000
                    ASRS_T Dgnss. COHS.z
Age.z                                   
Gender.z                                
Cong_Order.z                            
Stim_Order.z                            
Drive.z                                 
ASRS_A.z                                
ASRS_B.z                                
ASRS_Total.z                            
Diagnosis.z         -0.020              
COHS.z              -0.014  0.056       
StimulusTypeNovel.z  0.000  0.000  0.000

Standardized Within-Group Residuals:
        Min          Q1         Med          Q3         Max 
-3.22916619 -0.60850687  0.02559481  0.63312222  2.70553352 

Number of Observations: 208
Number of Groups: 104
```

```
Linear mixed-effects model fit by maximum likelihood
 Data: data 
       AIC      BIC    logLik
  586.9089 650.3222 -274.4545

Random effects:
 Formula: ~1 | Subject
         (Intercept)  Residual
StdDev: 8.475628e-05 0.9053389

Fixed effects: Acc_Diff.z ~ Age.z + Gender.z + Cong_Order.z + Stim_Order.z +      Drive.z + ASRS_A.z + ASRS_B.z + ASRS_Total.z + Diagnosis.z +      COHS.z + StimulusTypeNovel.z + ASRS_A.z * StimulusTypeNovel.z +      ASRS_B.z * StimulusTypeNovel.z + ASRS_Total.z * StimulusTypeNovel.z +      Diagnosis.z * StimulusTypeNovel.z + COHS.z * StimulusTypeNovel.z 
                                      Value Std.Error DF   t-value p-value
(Intercept)                       0.0000000 0.0655080 98  0.000000  1.0000
Age.z                            -0.2725880 0.1763463 93 -1.545754  0.1256
Gender.z                         -0.0943841 0.0722202 93 -1.306893  0.1945
Cong_Order.z                      0.1766069 0.1347377 93  1.310746  0.1932
Stim_Order.z                     -0.2208710 0.1371711 93 -1.610186  0.1107
Drive.z                           0.3283236 0.1735809 93  1.891473  0.0617
ASRS_A.z                         -0.0470717 0.1969796 93 -0.238968  0.8117
ASRS_B.z                          0.0169484 0.1941740 93  0.087284  0.9306
ASRS_Total.z                      0.0352486 0.3185583 93  0.110650  0.9121
Diagnosis.z                       0.0318194 0.0757028 93  0.420321  0.6752
COHS.z                           -0.0556694 0.0682267 93 -0.815947  0.4166
StimulusTypeNovel.z               0.3087631 0.0656661 98  4.702019  0.0000
ASRS_A.z:StimulusTypeNovel.z      0.1714261 0.1925757 98  0.890175  0.3756
ASRS_B.z:StimulusTypeNovel.z      0.1459982 0.1916855 98  0.761655  0.4481
ASRS_Total.z:StimulusTypeNovel.z -0.3518080 0.3141462 98 -1.119886  0.2655
Diagnosis.z:StimulusTypeNovel.z   0.1038756 0.0673812 98  1.541611  0.1264
COHS.z:StimulusTypeNovel.z        0.1177012 0.0675720 98  1.741863  0.0847
 Correlation: 
                                 (Intr) Age.z  Gndr.z Cng_O. Stm_O. Driv.z
Age.z                             0.000                                   
Gender.z                          0.000 -0.224                            
Cong_Order.z                      0.000 -0.094  0.163                     
Stim_Order.z                      0.000  0.103 -0.256 -0.864              
Drive.z                           0.000 -0.902  0.157  0.070 -0.094       
ASRS_A.z                          0.000  0.048 -0.135  0.055  0.045 -0.046
ASRS_B.z                          0.000  0.126 -0.015  0.082 -0.055 -0.086
ASRS_Total.z                      0.000 -0.069  0.051 -0.116  0.045  0.068
Diagnosis.z                       0.000 -0.078  0.216  0.113 -0.115 -0.110
COHS.z                            0.000  0.124 -0.078  0.015  0.006 -0.138
StimulusTypeNovel.z               0.000  0.000  0.000  0.000  0.000  0.000
ASRS_A.z:StimulusTypeNovel.z      0.000  0.000  0.000  0.000  0.000  0.000
ASRS_B.z:StimulusTypeNovel.z      0.000  0.000  0.000  0.000  0.000  0.000
ASRS_Total.z:StimulusTypeNovel.z  0.000  0.000  0.000  0.000  0.000  0.000
Diagnosis.z:StimulusTypeNovel.z   0.000  0.000  0.000  0.000  0.000  0.000
COHS.z:StimulusTypeNovel.z        0.000  0.000  0.000  0.000  0.000  0.000
                                 ASRS_A.z ASRS_B.z ASRS_Tt. Dgnss. COHS.z
Age.z                                                                    
Gender.z                                                                 
Cong_Order.z                                                             
Stim_Order.z                                                             
Drive.z                                                                  
ASRS_A.z                                                                 
ASRS_B.z                          0.692                                  
ASRS_Total.z                     -0.901   -0.895                         
Diagnosis.z                      -0.055    0.062   -0.020                
COHS.z                            0.077   -0.075   -0.014    0.056       
StimulusTypeNovel.z               0.000    0.000    0.000    0.000  0.000
ASRS_A.z:StimulusTypeNovel.z      0.000    0.000    0.000    0.000  0.000
ASRS_B.z:StimulusTypeNovel.z      0.000    0.000    0.000    0.000  0.000
ASRS_Total.z:StimulusTypeNovel.z  0.000    0.000    0.000    0.000  0.000
Diagnosis.z:StimulusTypeNovel.z   0.000    0.000    0.000    0.000  0.000
COHS.z:StimulusTypeNovel.z        0.000    0.000    0.000    0.000  0.000
                                 StmTN. ASRS_A.: ASRS_B.: ASRS_T.: D.:STN
Age.z                                                                    
Gender.z                                                                 
Cong_Order.z                                                             
Stim_Order.z                                                             
Drive.z                                                                  
ASRS_A.z                                                                 
ASRS_B.z                                                                 
ASRS_Total.z                                                             
Diagnosis.z                                                              
COHS.z                                                                   
StimulusTypeNovel.z                                                      
ASRS_A.z:StimulusTypeNovel.z      0.000                                  
ASRS_B.z:StimulusTypeNovel.z      0.000  0.704                           
ASRS_Total.z:StimulusTypeNovel.z  0.000 -0.905   -0.900                  
Diagnosis.z:StimulusTypeNovel.z   0.000 -0.040    0.101   -0.022         
COHS.z:StimulusTypeNovel.z        0.000  0.060   -0.091    0.004    0.054

Standardized Within-Group Residuals:
        Min          Q1         Med          Q3         Max 
-3.25400713 -0.56730420  0.03134097  0.62224816  2.67395533 

Number of Observations: 208
Number of Groups: 104
```

In [9]:

```
#Extract the R^2 value of each model
r.squaredGLMM(nogo_nofb_model1)
r.squaredGLMM(nogo_nofb_model2)
r.squaredGLMM(nogo_nofb_model3)
r.squaredGLMM(nogo_nofb_model4)
```

```
Warning message:
"'r.squaredGLMM' now calculates a revised statistic. See the help page."
```

| R2m | R2c |
| --- | --- |
| 0.04619542 | 0.04619542 |

| R2m | R2c |
| --- | --- |
| 0.05026976 | 0.05026976 |

| R2m | R2c |
| --- | --- |
| 0.1459746 | 0.1459746 |

| R2m | R2c |
| --- | --- |
| 0.1771031 | 0.1771031 |

In [10]:

```
#Subtract from each other to derive delta R^2. First will be 2-1, next 3-2. 
r.squaredGLMM(nogo_nofb_model2) - r.squaredGLMM(nogo_nofb_model1)
r.squaredGLMM(nogo_nofb_model3) - r.squaredGLMM(nogo_nofb_model2)
r.squaredGLMM(nogo_nofb_model4) - r.squaredGLMM(nogo_nofb_model3)
```

| R2m | R2c |
| --- | --- |
| 0.004074338 | 0.004074339 |

| R2m | R2c |
| --- | --- |
| 0.09570482 | 0.09570482 |

| R2m | R2c |
| --- | --- |
| 0.03112849 | 0.03112848 |

In [11]:

```
#Compare the models to each other to extract log likelihood ratio Chi^2 values and the associated p-values. 
#Df is how many new variables are added to next model.
anova(nogo_nofb_model1, nogo_nofb_model2, nogo_nofb_model3, nogo_nofb_model4)
```

|  | call | Model | df | AIC | BIC | logLik | Test | L.Ratio | p-value |
| --- | --- | --- | --- | --- | --- | --- | --- | --- | --- |
| nogo\_nofb\_model1 | lme.formula(fixed = Acc\_Diff ~ Age + Gender + Cong\_Order + Stim\_Order + Drive, data = myNoGoDay1Data, random = ~1 | Subject, method = "ML") | 1 | 8 | -147.0928 | -120.39246 | 81.54638 |  | NA | NA |
| nogo\_nofb\_model2 | lme.formula(fixed = Acc\_Diff ~ Age + Gender + Cong\_Order + Stim\_Order + Drive + ASRS\_A + ASRS\_B + ASRS\_Total + Diagnosis + COHS, data = myNoGoDay1Data, random = ~1 | Subject, method = "ML") | 2 | 13 | -137.9791 | -94.59111 | 81.98955 | 1 vs 2 | 0.8863352 | 9.712030e-01 |
| nogo\_nofb\_model3 | lme.formula(fixed = Acc\_Diff ~ Age + Gender + Cong\_Order + Stim\_Order + Drive + ASRS\_A + ASRS\_B + ASRS\_Total + Diagnosis + COHS + StimulusType, data = myNoGoDay1Data, random = ~1 | Subject, method = "ML") | 3 | 14 | -157.9765 | -111.25096 | 92.98825 | 2 vs 3 | 21.9973927 | 2.730211e-06 |
| nogo\_nofb\_model4 | lme.formula(fixed = Acc\_Diff ~ Age + Gender + Cong\_Order + Stim\_Order + Drive + ASRS\_A + ASRS\_B + ASRS\_Total + Diagnosis + COHS + StimulusType + ASRS\_A \* StimulusType + ASRS\_B \* StimulusType + ASRS\_Total \* StimulusType + Diagnosis \* StimulusType + COHS \* StimulusType, data = myNoGoDay1Data, random = ~1 | Subject, method = "ML") | 4 | 19 | -155.6684 | -92.25516 | 96.83419 | 3 vs 4 | 7.6918869 | 1.740538e-01 |

## Second set of analysis will involve habit disruption, therefore data from both days, but Familiar condition only¶

In [12]:

```
#Take subset of dataframe to analyze Familiar data (Feedback and NoFeedback). We'll call it "myNoGoDay2Data" for consistency.
myNoGoDay2Data <- subset(myNoGoData, StimulusType=="Familiar")
```

### Similarly, run mixed models with ML estimations to compare fixed effects, using subject as the random factor.¶

In [13]:

```
nogo_fb_model1 <- lme(Acc_Diff ~ Age + Gender + Cong_Order + Stim_Order + Drive, random=~1|Subject, method="ML", data=myNoGoDay2Data)
nogo_fb_model2 <- lme(Acc_Diff ~ Age + Gender + Cong_Order + Stim_Order + Drive + ASRS_A + ASRS_B + ASRS_Total + Diagnosis + COHS, random=~1|Subject, method="ML", data=myNoGoDay2Data)
nogo_fb_model3 <- lme(Acc_Diff ~ Age + Gender + Cong_Order + Stim_Order + Drive + ASRS_A + ASRS_B + ASRS_Total + Diagnosis + COHS + FeedbackCond, random=~1|Subject, method="ML", data=myNoGoDay2Data)
nogo_fb_model4 <- lme(Acc_Diff ~ Age + Gender + Cong_Order + Stim_Order + Drive + ASRS_A + ASRS_B + ASRS_Total + Diagnosis + COHS + FeedbackCond + ASRS_A*FeedbackCond + ASRS_B*FeedbackCond + ASRS_Total*FeedbackCond + Diagnosis*FeedbackCond + COHS*FeedbackCond, random=~1|Subject, method="ML", data=myNoGoDay2Data)
```

In [14]:

```
#Check for outliers, beyond -3.3<x<3.3. No output means no outliers.
which(abs(residuals(nogo_fb_model1, type="normalized")) > 3.3)
which(abs(residuals(nogo_fb_model2, type="normalized")) > 3.3)
which(abs(residuals(nogo_fb_model3, type="normalized")) > 3.3)
which(abs(residuals(nogo_fb_model4, type="normalized")) > 3.3)
```

In [15]:

```
#Diagnostics. plot() checks for homoscedasticity violation, qqplot() checks for normality, vif() checks for multicollinearity
qqnorm(resid(nogo_fb_model1))
qqnorm(resid(nogo_fb_model2))
qqnorm(resid(nogo_fb_model3))
qqnorm(resid(nogo_fb_model4))
plot(nogo_fb_model1)
plot(nogo_fb_model2)
plot(nogo_fb_model3)
plot(nogo_fb_model4)
```

In [16]:

```
vif(nogo_fb_model1)
vif(nogo_fb_model2)
vif(nogo_fb_model3)
vif(nogo_fb_model4)
```

Age
:   6.76773370364329

Gender
:   1.10278256254609

Cong\_Order
:   3.97919186192501

Stim\_Order
:   4.1110398011186

Drive
:   6.71293269748105

Age
:   7.21191297797025

Gender
:   1.20958269153333

Cong\_Order
:   4.2101348502977

Stim\_Order
:   4.36358183746821

Drive
:   6.98750136278286

ASRS\_A
:   8.99829578776253

ASRS\_B
:   8.74379792221156

ASRS\_Total
:   23.5340071905795

Diagnosis
:   1.32905065526235

COHS
:   1.07951043474563

Age
:   7.21191297797028

Gender
:   1.20958269153335

Cong\_Order
:   4.21013485029776

Stim\_Order
:   4.36358183746822

Drive
:   6.98750136278284

ASRS\_A
:   8.99829578776265

ASRS\_B
:   8.74379792221161

ASRS\_Total
:   23.5340071905798

Diagnosis
:   1.32905065526235

COHS
:   1.07951043474563

FeedbackCond
:   1

Age
:   7.21191297797043

Gender
:   1.20958269153336

Cong\_Order
:   4.21013485029789

Stim\_Order
:   4.36358183746843

Drive
:   6.98750136278304

ASRS\_A
:   17.5573961248027

ASRS\_B
:   17.2239514905662

ASRS\_Total
:   46.3105936447702

Diagnosis
:   2.376908531593

COHS
:   2.13331112644241

FeedbackCond
:   64.9691076182593

ASRS\_A:FeedbackCond
:   91.1111346993793

ASRS\_B:FeedbackCond
:   71.6557128337506

ASRS\_Total:FeedbackCond
:   265.779061594907

Diagnosis:FeedbackCond
:   2.1598703202563

COHS:FeedbackCond
:   57.3687507973028

In [17]:

```
beta(nogo_fb_model1)
beta(nogo_fb_model2)
beta(nogo_fb_model3)
beta(nogo_fb_model4)
```

```
Linear mixed-effects model fit by maximum likelihood
 Data: data 
       AIC      BIC    logLik
  596.4015 623.1018 -290.2008

Random effects:
 Formula: ~1 | Subject
         (Intercept) Residual
StdDev: 8.405175e-05 0.976537

Fixed effects: Acc_Diff.z ~ Age.z + Gender.z + Cong_Order.z + Stim_Order.z +      Drive.z 
                   Value  Std.Error  DF    t-value p-value
(Intercept)   0.00000000 0.06870891 104  0.0000000  1.0000
Age.z         0.28797508 0.17917655  98  1.6072141  0.1112
Gender.z      0.04337723 0.07232767  98  0.5997321  0.5501
Cong_Order.z  0.30199227 0.13739058  98  2.1980565  0.0303
Stim_Order.z -0.23322151 0.13964821  98 -1.6700645  0.0981
Drive.z      -0.27559361 0.17844964  98 -1.5443775  0.1257
 Correlation: 
             (Intr) Age.z  Gndr.z Cng_O. Stm_O.
Age.z         0.000                            
Gender.z      0.000 -0.206                     
Cong_Order.z  0.000 -0.082  0.129              
Stim_Order.z  0.000  0.096 -0.212 -0.863       
Drive.z       0.000 -0.922  0.178  0.090 -0.114

Standardized Within-Group Residuals:
        Min          Q1         Med          Q3         Max 
-2.66320360 -0.62693736  0.08214435  0.65977841  2.67481999 

Number of Observations: 208
Number of Groups: 104
```

```
Linear mixed-effects model fit by maximum likelihood
 Data: data 
       AIC      BIC    logLik
  602.0547 645.4427 -288.0273

Random effects:
 Formula: ~1 | Subject
         (Intercept)  Residual
StdDev: 7.560095e-05 0.9663862

Fixed effects: Acc_Diff.z ~ Age.z + Gender.z + Cong_Order.z + Stim_Order.z +      Drive.z + ASRS_A.z + ASRS_B.z + ASRS_Total.z + Diagnosis.z +      COHS.z 
                  Value Std.Error  DF    t-value p-value
(Intercept)   0.0000000 0.0688522 104  0.0000000  1.0000
Age.z         0.3197986 0.1853486  93  1.7253900  0.0878
Gender.z      0.0470799 0.0759070  93  0.6202313  0.5366
Cong_Order.z  0.3030269 0.1416159  93  2.1397800  0.0350
Stim_Order.z -0.2464764 0.1441736  93 -1.7095812  0.0907
Drive.z      -0.2643304 0.1824421  93 -1.4488455  0.1507
ASRS_A.z      0.0501675 0.2070352  93  0.2423141  0.8091
ASRS_B.z      0.2797611 0.2040864  93  1.3707971  0.1737
ASRS_Total.z -0.2696833 0.3348205  93 -0.8054564  0.4226
Diagnosis.z  -0.0424021 0.0795673  93 -0.5329083  0.5954
COHS.z       -0.0849211 0.0717096  93 -1.1842360  0.2393
 Correlation: 
             (Intr) Age.z  Gndr.z Cng_O. Stm_O. Driv.z ASRS_A ASRS_B ASRS_T
Age.z         0.000                                                        
Gender.z      0.000 -0.224                                                 
Cong_Order.z  0.000 -0.094  0.163                                          
Stim_Order.z  0.000  0.103 -0.256 -0.864                                   
Drive.z       0.000 -0.902  0.157  0.070 -0.094                            
ASRS_A.z      0.000  0.048 -0.135  0.055  0.045 -0.046                     
ASRS_B.z      0.000  0.126 -0.015  0.082 -0.055 -0.086  0.692              
ASRS_Total.z  0.000 -0.069  0.051 -0.116  0.045  0.068 -0.901 -0.895       
Diagnosis.z   0.000 -0.078  0.216  0.113 -0.115 -0.110 -0.055  0.062 -0.020
COHS.z        0.000  0.124 -0.078  0.015  0.006 -0.138  0.077 -0.075 -0.014
             Dgnss.
Age.z              
Gender.z           
Cong_Order.z       
Stim_Order.z       
Drive.z            
ASRS_A.z           
ASRS_B.z           
ASRS_Total.z       
Diagnosis.z        
COHS.z        0.056

Standardized Within-Group Residuals:
        Min          Q1         Med          Q3         Max 
-2.68979349 -0.67229584  0.06596359  0.69253014  2.56578443 

Number of Observations: 208
Number of Groups: 104
```

```
Linear mixed-effects model fit by maximum likelihood
 Data: data 
       AIC      BIC    logLik
  586.3289 633.0544 -279.1644

Random effects:
 Formula: ~1 | Subject
         (Intercept)  Residual
StdDev: 0.0001048736 0.9260734

Fixed effects: Acc_Diff.z ~ Age.z + Gender.z + Cong_Order.z + Stim_Order.z +      Drive.z + ASRS_A.z + ASRS_B.z + ASRS_Total.z + Diagnosis.z +      COHS.z + FeedbackCondNoFeedback.z 
                              Value Std.Error  DF   t-value p-value
(Intercept)               0.0000000 0.0661481 103  0.000000  1.0000
Age.z                     0.3197986 0.1780693  93  1.795922  0.0758
Gender.z                  0.0470799 0.0729259  93  0.645586  0.5201
Cong_Order.z              0.3030269 0.1360542  93  2.227252  0.0283
Stim_Order.z             -0.2464764 0.1385114  93 -1.779467  0.0784
Drive.z                  -0.2643304 0.1752770  93 -1.508073  0.1349
ASRS_A.z                  0.0501675 0.1989042  93  0.252220  0.8014
ASRS_B.z                  0.2797611 0.1960712  93  1.426834  0.1570
ASRS_Total.z             -0.2696833 0.3216709  93 -0.838383  0.4040
Diagnosis.z              -0.0424021 0.0764425  93 -0.554693  0.5804
COHS.z                   -0.0849211 0.0688933  93 -1.232646  0.2208
FeedbackCondNoFeedback.z -0.2768735 0.0663077 103 -4.175587  0.0001
 Correlation: 
                         (Intr) Age.z  Gndr.z Cng_O. Stm_O. Driv.z ASRS_A
Age.z                     0.000                                          
Gender.z                  0.000 -0.224                                   
Cong_Order.z              0.000 -0.094  0.163                            
Stim_Order.z              0.000  0.103 -0.256 -0.864                     
Drive.z                   0.000 -0.902  0.157  0.070 -0.094              
ASRS_A.z                  0.000  0.048 -0.135  0.055  0.045 -0.046       
ASRS_B.z                  0.000  0.126 -0.015  0.082 -0.055 -0.086  0.692
ASRS_Total.z              0.000 -0.069  0.051 -0.116  0.045  0.068 -0.901
Diagnosis.z               0.000 -0.078  0.216  0.113 -0.115 -0.110 -0.055
COHS.z                    0.000  0.124 -0.078  0.015  0.006 -0.138  0.077
FeedbackCondNoFeedback.z  0.000  0.000  0.000  0.000  0.000  0.000  0.000
                         ASRS_B ASRS_T Dgnss. COHS.z
Age.z                                               
Gender.z                                            
Cong_Order.z                                        
Stim_Order.z                                        
Drive.z                                             
ASRS_A.z                                            
ASRS_B.z                                            
ASRS_Total.z             -0.895                     
Diagnosis.z               0.062 -0.020              
COHS.z                   -0.075 -0.014  0.056       
FeedbackCondNoFeedback.z  0.000  0.000  0.000  0.000

Standardized Within-Group Residuals:
        Min          Q1         Med          Q3         Max 
-2.50862643 -0.63690698  0.04735982  0.65185355  2.96649730 

Number of Observations: 208
Number of Groups: 104
```

```
Linear mixed-effects model fit by maximum likelihood
 Data: data 
       AIC      BIC    logLik
  591.8593 655.2725 -276.9296

Random effects:
 Formula: ~1 | Subject
         (Intercept)  Residual
StdDev: 0.0001758412 0.9161766

Fixed effects: Acc_Diff.z ~ Age.z + Gender.z + Cong_Order.z + Stim_Order.z +      Drive.z + ASRS_A.z + ASRS_B.z + ASRS_Total.z + Diagnosis.z +      COHS.z + FeedbackCondNoFeedback.z + ASRS_A.z * FeedbackCondNoFeedback.z +      ASRS_B.z * FeedbackCondNoFeedback.z + ASRS_Total.z * FeedbackCondNoFeedback.z +      Diagnosis.z * FeedbackCondNoFeedback.z + COHS.z * FeedbackCondNoFeedback.z 
                                           Value Std.Error DF   t-value p-value
(Intercept)                            0.0000000 0.0662922 98  0.000000  1.0000
Age.z                                  0.3197986 0.1784573 93  1.792018  0.0764
Gender.z                               0.0470799 0.0730848 93  0.644182  0.5210
Cong_Order.z                           0.3030269 0.1363506 93  2.222410  0.0287
Stim_Order.z                          -0.2464764 0.1388131 93 -1.775599  0.0791
Drive.z                               -0.2643304 0.1756588 93 -1.504794  0.1358
ASRS_A.z                               0.0501675 0.1993376 93  0.251671  0.8019
ASRS_B.z                               0.2797611 0.1964984 93  1.423732  0.1579
ASRS_Total.z                          -0.2696833 0.3223717 93 -0.836560  0.4050
Diagnosis.z                           -0.0424021 0.0766090 93 -0.553487  0.5813
COHS.z                                -0.0849211 0.0690434 93 -1.229967  0.2218
FeedbackCondNoFeedback.z              -0.2768735 0.0664521 98 -4.166510  0.0001
ASRS_A.z:FeedbackCondNoFeedback.z     -0.2345784 0.1948810 98 -1.203700  0.2316
ASRS_B.z:FeedbackCondNoFeedback.z     -0.2975861 0.1939802 98 -1.534105  0.1282
ASRS_Total.z:FeedbackCondNoFeedback.z  0.5161074 0.3179068 98  1.623455  0.1077
Diagnosis.z:FeedbackCondNoFeedback.z   0.0140595 0.0681878 98  0.206187  0.8371
COHS.z:FeedbackCondNoFeedback.z       -0.0592250 0.0683809 98 -0.866105  0.3885
 Correlation: 
                                      (Intr) Age.z  Gndr.z Cng_O. Stm_O. Driv.z
Age.z                                  0.000                                   
Gender.z                               0.000 -0.224                            
Cong_Order.z                           0.000 -0.094  0.163                     
Stim_Order.z                           0.000  0.103 -0.256 -0.864              
Drive.z                                0.000 -0.902  0.157  0.070 -0.094       
ASRS_A.z                               0.000  0.048 -0.135  0.055  0.045 -0.046
ASRS_B.z                               0.000  0.126 -0.015  0.082 -0.055 -0.086
ASRS_Total.z                           0.000 -0.069  0.051 -0.116  0.045  0.068
Diagnosis.z                            0.000 -0.078  0.216  0.113 -0.115 -0.110
COHS.z                                 0.000  0.124 -0.078  0.015  0.006 -0.138
FeedbackCondNoFeedback.z               0.000  0.000  0.000  0.000  0.000  0.000
ASRS_A.z:FeedbackCondNoFeedback.z      0.000  0.000  0.000  0.000  0.000  0.000
ASRS_B.z:FeedbackCondNoFeedback.z      0.000  0.000  0.000  0.000  0.000  0.000
ASRS_Total.z:FeedbackCondNoFeedback.z  0.000  0.000  0.000  0.000  0.000  0.000
Diagnosis.z:FeedbackCondNoFeedback.z   0.000  0.000  0.000  0.000  0.000  0.000
COHS.z:FeedbackCondNoFeedback.z        0.000  0.000  0.000  0.000  0.000  0.000
                                      ASRS_A.z ASRS_B.z ASRS_Tt. Dgnss. COHS.z
Age.z                                                                         
Gender.z                                                                      
Cong_Order.z                                                                  
Stim_Order.z                                                                  
Drive.z                                                                       
ASRS_A.z                                                                      
ASRS_B.z                               0.692                                  
ASRS_Total.z                          -0.901   -0.895                         
Diagnosis.z                           -0.055    0.062   -0.020                
COHS.z                                 0.077   -0.075   -0.014    0.056       
FeedbackCondNoFeedback.z               0.000    0.000    0.000    0.000  0.000
ASRS_A.z:FeedbackCondNoFeedback.z      0.000    0.000    0.000    0.000  0.000
ASRS_B.z:FeedbackCondNoFeedback.z      0.000    0.000    0.000    0.000  0.000
ASRS_Total.z:FeedbackCondNoFeedback.z  0.000    0.000    0.000    0.000  0.000
Diagnosis.z:FeedbackCondNoFeedback.z   0.000    0.000    0.000    0.000  0.000
COHS.z:FeedbackCondNoFeedback.z        0.000    0.000    0.000    0.000  0.000
                                      FdCNF. ASRS_A.: ASRS_B.: ASRS_T.: D.:FCN
Age.z                                                                         
Gender.z                                                                      
Cong_Order.z                                                                  
Stim_Order.z                                                                  
Drive.z                                                                       
ASRS_A.z                                                                      
ASRS_B.z                                                                      
ASRS_Total.z                                                                  
Diagnosis.z                                                                   
COHS.z                                                                        
FeedbackCondNoFeedback.z                                                      
ASRS_A.z:FeedbackCondNoFeedback.z      0.000                                  
ASRS_B.z:FeedbackCondNoFeedback.z      0.000  0.704                           
ASRS_Total.z:FeedbackCondNoFeedback.z  0.000 -0.905   -0.900                  
Diagnosis.z:FeedbackCondNoFeedback.z   0.000 -0.040    0.101   -0.022         
COHS.z:FeedbackCondNoFeedback.z        0.000  0.060   -0.091    0.004    0.054

Standardized Within-Group Residuals:
        Min          Q1         Med          Q3         Max 
-2.56157612 -0.60411184  0.03389155  0.66644709  2.98005406 

Number of Observations: 208
Number of Groups: 104
```

In [18]:

```
#Extract the R^2 value of each model
r.squaredGLMM(nogo_fb_model1)
r.squaredGLMM(nogo_fb_model2)
r.squaredGLMM(nogo_fb_model3)
r.squaredGLMM(nogo_fb_model4)
```

| R2m | R2c |
| --- | --- |
| 0.04196185 | 0.04196185 |

| R2m | R2c |
| --- | --- |
| 0.06186521 | 0.06186521 |

| R2m | R2c |
| --- | --- |
| 0.1388202 | 0.1388202 |

| R2m | R2c |
| --- | --- |
| 0.1572029 | 0.1572029 |

In [19]:

```
#Subtract from each other to derive delta R^2. First will be 2-1, next 3-2. 
r.squaredGLMM(nogo_fb_model2) - r.squaredGLMM(nogo_fb_model1)
r.squaredGLMM(nogo_fb_model3) - r.squaredGLMM(nogo_fb_model2)
r.squaredGLMM(nogo_fb_model4) - r.squaredGLMM(nogo_fb_model3)
```

| R2m | R2c |
| --- | --- |
| 0.01990336 | 0.01990336 |

| R2m | R2c |
| --- | --- |
| 0.07695498 | 0.07695498 |

| R2m | R2c |
| --- | --- |
| 0.01838267 | 0.01838267 |

In [20]:

```
#Compare models
anova(nogo_fb_model1, nogo_fb_model2, nogo_fb_model3, nogo_fb_model4)
```

|  | call | Model | df | AIC | BIC | logLik | Test | L.Ratio | p-value |
| --- | --- | --- | --- | --- | --- | --- | --- | --- | --- |
| nogo\_fb\_model1 | lme.formula(fixed = Acc\_Diff ~ Age + Gender + Cong\_Order + Stim\_Order + Drive, data = myNoGoDay2Data, random = ~1 | Subject, method = "ML") | 1 | 8 | -135.0926 | -108.39226 | 75.54628 |  | NA | NA |
| nogo\_fb\_model2 | lme.formula(fixed = Acc\_Diff ~ Age + Gender + Cong\_Order + Stim\_Order + Drive + ASRS\_A + ASRS\_B + ASRS\_Total + Diagnosis + COHS, data = myNoGoDay2Data, random = ~1 | Subject, method = "ML") | 2 | 13 | -129.4394 | -86.05141 | 77.71970 | 1 vs 2 | 4.346836 | 5.006339e-01 |
| nogo\_fb\_model3 | lme.formula(fixed = Acc\_Diff ~ Age + Gender + Cong\_Order + Stim\_Order + Drive + ASRS\_A + ASRS\_B + ASRS\_Total + Diagnosis + COHS + FeedbackCond, data = myNoGoDay2Data, random = ~1 | Subject, method = "ML") | 3 | 14 | -145.1652 | -98.43966 | 86.58260 | 2 vs 3 | 17.725793 | 2.551435e-05 |
| nogo\_fb\_model4 | lme.formula(fixed = Acc\_Diff ~ Age + Gender + Cong\_Order + Stim\_Order + Drive + ASRS\_A + ASRS\_B + ASRS\_Total + Diagnosis + COHS + FeedbackCond + ASRS\_A \* FeedbackCond + ASRS\_B \* FeedbackCond + ASRS\_Total \* FeedbackCond + Diagnosis \* FeedbackCond + COHS \* FeedbackCond, data = myNoGoDay2Data, random = ~1 | Subject, method = "ML") | 4 | 19 | -139.6348 | -76.22160 | 88.81741 | 3 vs 4 | 4.469627 | 4.839575e-01 |

## Same analyses with Go accuracy difference as DV. For that, we'll need to read the csv with Go information.¶

In [21]:

```
#Read csv with Go information (Days 1 and 2)
myGoData <- read.csv('Exp8_Go_Full.csv')
```

### First, habit expression and ADHD symptomology analyses using Day 1 data.¶

In [22]:

```
#Take subset of dataframe to analyze Day 1 data (Familiar and Novel NoFeedback)
#Having inspected the day 1 standardized residuals, subjects 76, 79, 101, and 106 are outside the -3.3<x<3.3 range, thus day 1 outliers
myGoDay1Data <- subset(myGoData, FeedbackCond=="NoFeedback")
```

In [23]:

```
go_nofb_model1 <- lme(Acc_Diff ~ Age + Gender + Cong_Order + Stim_Order + Drive, random=~1|Subject, method="ML", data=myGoDay1Data)
go_nofb_model2 <- lme(Acc_Diff ~ Age + Gender + Cong_Order + Stim_Order + Drive + ASRS_A + ASRS_B + ASRS_Total + Diagnosis + COHS, random=~1|Subject, method="ML", data=myGoDay1Data)
go_nofb_model3 <- lme(Acc_Diff ~ Age + Gender + Cong_Order + Stim_Order + Drive + ASRS_A + ASRS_B + ASRS_Total + Diagnosis + COHS + StimulusType, random=~1|Subject, method="ML", data=myGoDay1Data)
go_nofb_model4 <- lme(Acc_Diff ~ Age + Gender + Cong_Order + Stim_Order + Drive + ASRS_A + ASRS_B + ASRS_Total + Diagnosis + COHS + StimulusType + ASRS_A*StimulusType + ASRS_B*StimulusType + ASRS_Total*StimulusType + Diagnosis*StimulusType + COHS*StimulusType, random=~1|Subject, method="ML", data=myGoDay1Data)
```

In [24]:

```
#Check for outliers, beyond -3.3<x<3.3. No output means no outliers.
which(abs(residuals(go_nofb_model1, type="normalized")) > 3.3)
which(abs(residuals(go_nofb_model2, type="normalized")) > 3.3)
which(abs(residuals(go_nofb_model3, type="normalized")) > 3.3)
which(abs(residuals(go_nofb_model4, type="normalized")) > 3.3)
```

79
:   181

106
:   208

79
:   181

106
:   208

76
:   178

79
:   181

106
:   208

76
:   178

79
:   181

106
:   208

In [25]:

```
#Ran the following analyses with and without these outliers, and saw no difference. Therefore, the below outliers include complete dataset.
#myGoDay1Data <- subset(myGoDay1Data, Subject!=76 & Subject!=79 & Subject!=106)
go_nofb_model1 <- lme(Acc_Diff ~ Age + Gender + Cong_Order + Stim_Order + Drive, random=~1|Subject, method="ML", data=myGoDay1Data)
go_nofb_model2 <- lme(Acc_Diff ~ Age + Gender + Cong_Order + Stim_Order + Drive + ASRS_A + ASRS_B + ASRS_Total + Diagnosis + COHS, random=~1|Subject, method="ML", data=myGoDay1Data)
go_nofb_model3 <- lme(Acc_Diff ~ Age + Gender + Cong_Order + Stim_Order + Drive + ASRS_A + ASRS_B + ASRS_Total + Diagnosis + COHS + StimulusType, random=~1|Subject, method="ML", data=myGoDay1Data)
go_nofb_model4 <- lme(Acc_Diff ~ Age + Gender + Cong_Order + Stim_Order + Drive + ASRS_A + ASRS_B + ASRS_Total + Diagnosis + COHS + StimulusType + ASRS_A*StimulusType + ASRS_B*StimulusType + ASRS_Total*StimulusType + Diagnosis*StimulusType + COHS*StimulusType, random=~1|Subject, method="ML", data=myGoDay1Data)
```

In [26]:

```
#Diagnostics. plot() checks for homoscedasticity violation, qqplot() checks for normality, vif() checks for multicollinearity
qqnorm(resid(go_nofb_model1))
qqnorm(resid(go_nofb_model2))
qqnorm(resid(go_nofb_model3))
qqnorm(resid(go_nofb_model4))
plot(go_nofb_model1)
plot(go_nofb_model2)
plot(go_nofb_model3)
plot(go_nofb_model4)
```

In [27]:

```
vif(go_nofb_model1)
vif(go_nofb_model2)
vif(go_nofb_model3)
vif(go_nofb_model4)
```

Age
:   6.76773370364327

Gender
:   1.10278256254609

Cong\_Order
:   3.97919186192501

Stim\_Order
:   4.11103980111859

Drive
:   6.71293269748103

Age
:   7.21191297797033

Gender
:   1.20958269153334

Cong\_Order
:   4.21013485029773

Stim\_Order
:   4.36358183746823

Drive
:   6.98750136278284

ASRS\_A
:   8.99829578776265

ASRS\_B
:   8.74379792221166

ASRS\_Total
:   23.5340071905799

Diagnosis
:   1.32905065526235

COHS
:   1.07951043474563

Age
:   7.21191297797029

Gender
:   1.20958269153334

Cong\_Order
:   4.21013485029773

Stim\_Order
:   4.36358183746821

Drive
:   6.98750136278287

ASRS\_A
:   8.9982957877626

ASRS\_B
:   8.74379792221159

ASRS\_Total
:   23.5340071905797

Diagnosis
:   1.32905065526235

COHS
:   1.07951043474563

StimulusType
:   1

Age
:   7.21191297796994

Gender
:   1.20958269153328

Cong\_Order
:   4.21013485029758

Stim\_Order
:   4.36358183746813

Drive
:   6.98750136278259

ASRS\_A
:   17.5573961490221

ASRS\_B
:   17.2239515145636

ASRS\_Total
:   46.3105937092239

Diagnosis
:   2.37690853455821

COHS
:   2.13331112942441

StimulusType
:   64.9691076182534

ASRS\_A:StimulusType
:   91.1111347235926

ASRS\_B:StimulusType
:   71.6557128577423

ASRS\_Total:StimulusType
:   265.779061659338

Diagnosis:StimulusType
:   2.15987032322153

COHS:StimulusType
:   57.3687508002802

In [28]:

```
beta(go_nofb_model1)
beta(go_nofb_model2)
beta(go_nofb_model3)
beta(go_nofb_model4)
```

```
Linear mixed-effects model fit by maximum likelihood
 Data: data 
      AIC      BIC    logLik
  604.107 630.8073 -294.0535

Random effects:
 Formula: ~1 | Subject
         (Intercept)  Residual
StdDev: 5.836766e-05 0.9947938

Fixed effects: Acc_Diff.z ~ Age.z + Gender.z + Cong_Order.z + Stim_Order.z +      Drive.z 
                   Value  Std.Error  DF    t-value p-value
(Intercept)   0.00000000 0.06999345 104  0.0000000  1.0000
Age.z        -0.07408585 0.18252633  98 -0.4058913  0.6857
Gender.z      0.02912284 0.07367987  98  0.3952618  0.6935
Cong_Order.z  0.04360765 0.13995916  98  0.3115741  0.7560
Stim_Order.z -0.01049453 0.14225899  98 -0.0737706  0.9413
Drive.z       0.11949893 0.18178583  98  0.6573611  0.5125
 Correlation: 
             (Intr) Age.z  Gndr.z Cng_O. Stm_O.
Age.z         0.000                            
Gender.z      0.000 -0.206                     
Cong_Order.z  0.000 -0.082  0.129              
Stim_Order.z  0.000  0.096 -0.212 -0.863       
Drive.z       0.000 -0.922  0.178  0.090 -0.114

Standardized Within-Group Residuals:
        Min          Q1         Med          Q3         Max 
-3.01171378 -0.40331764 -0.08290877  0.38128143  5.13999461 

Number of Observations: 208
Number of Groups: 104
```

```
Linear mixed-effects model fit by maximum likelihood
 Data: data 
       AIC      BIC    logLik
  602.5203 645.9083 -288.2602

Random effects:
 Formula: ~1 | Subject
         (Intercept)  Residual
StdDev: 4.832084e-05 0.9674686

Fixed effects: Acc_Diff.z ~ Age.z + Gender.z + Cong_Order.z + Stim_Order.z +      Drive.z + ASRS_A.z + ASRS_B.z + ASRS_Total.z + Diagnosis.z +      COHS.z 
                  Value Std.Error  DF    t-value p-value
(Intercept)   0.0000000 0.0689293 104  0.0000000  1.0000
Age.z        -0.1928827 0.1855562  93 -1.0394840  0.3013
Gender.z      0.0806437 0.0759920  93  1.0612132  0.2913
Cong_Order.z  0.0554230 0.1417745  93  0.3909236  0.6967
Stim_Order.z -0.0417606 0.1443351  93 -0.2893312  0.7730
Drive.z       0.1477204 0.1826464  93  0.8087781  0.4207
ASRS_A.z     -0.4656776 0.2072671  93 -2.2467509  0.0270
ASRS_B.z     -0.4846481 0.2043150  93 -2.3720630  0.0197
ASRS_Total.z  0.7820574 0.3351955  93  2.3331382  0.0218
Diagnosis.z   0.1474151 0.0796565  93  1.8506355  0.0674
COHS.z       -0.0611066 0.0717900  93 -0.8511864  0.3969
 Correlation: 
             (Intr) Age.z  Gndr.z Cng_O. Stm_O. Driv.z ASRS_A ASRS_B ASRS_T
Age.z         0.000                                                        
Gender.z      0.000 -0.224                                                 
Cong_Order.z  0.000 -0.094  0.163                                          
Stim_Order.z  0.000  0.103 -0.256 -0.864                                   
Drive.z       0.000 -0.902  0.157  0.070 -0.094                            
ASRS_A.z      0.000  0.048 -0.135  0.055  0.045 -0.046                     
ASRS_B.z      0.000  0.126 -0.015  0.082 -0.055 -0.086  0.692              
ASRS_Total.z  0.000 -0.069  0.051 -0.116  0.045  0.068 -0.901 -0.895       
Diagnosis.z   0.000 -0.078  0.216  0.113 -0.115 -0.110 -0.055  0.062 -0.020
COHS.z        0.000  0.124 -0.078  0.015  0.006 -0.138  0.077 -0.075 -0.014
             Dgnss.
Age.z              
Gender.z           
Cong_Order.z       
Stim_Order.z       
Drive.z            
ASRS_A.z           
ASRS_B.z           
ASRS_Total.z       
Diagnosis.z        
COHS.z        0.056

Standardized Within-Group Residuals:
        Min          Q1         Med          Q3         Max 
-3.09794636 -0.45633592 -0.06737972  0.40435218  5.24372457 

Number of Observations: 208
Number of Groups: 104
```

```
Linear mixed-effects model fit by maximum likelihood
 Data: data 
       AIC      BIC    logLik
  597.2374 643.9629 -284.6187

Random effects:
 Formula: ~1 | Subject
         (Intercept)  Residual
StdDev: 5.011307e-05 0.9506784

Fixed effects: Acc_Diff.z ~ Age.z + Gender.z + Cong_Order.z + Stim_Order.z +      Drive.z + ASRS_A.z + ASRS_B.z + ASRS_Total.z + Diagnosis.z +      COHS.z + StimulusTypeNovel.z 
                         Value Std.Error  DF    t-value p-value
(Intercept)          0.0000000 0.0679056 103  0.0000000  1.0000
Age.z               -0.1928827 0.1828005  93 -1.0551543  0.2941
Gender.z             0.0806437 0.0748635  93  1.0772110  0.2842
Cong_Order.z         0.0554230 0.1396690  93  0.3968168  0.6924
Stim_Order.z        -0.0417606 0.1421915  93 -0.2936929  0.7696
Drive.z              0.1477204 0.1799339  93  0.8209705  0.4138
ASRS_A.z            -0.4656776 0.2041890  93 -2.2806208  0.0249
ASRS_B.z            -0.4846481 0.2012807  93 -2.4078219  0.0180
ASRS_Total.z         0.7820574 0.3302175  93  2.3683103  0.0199
Diagnosis.z          0.1474151 0.0784735  93  1.8785340  0.0634
COHS.z              -0.0611066 0.0707238  93 -0.8640181  0.3898
StimulusTypeNovel.z  0.1798932 0.0680694 103  2.6427904  0.0095
 Correlation: 
                    (Intr) Age.z  Gndr.z Cng_O. Stm_O. Driv.z ASRS_A ASRS_B
Age.z                0.000                                                 
Gender.z             0.000 -0.224                                          
Cong_Order.z         0.000 -0.094  0.163                                   
Stim_Order.z         0.000  0.103 -0.256 -0.864                            
Drive.z              0.000 -0.902  0.157  0.070 -0.094                     
ASRS_A.z             0.000  0.048 -0.135  0.055  0.045 -0.046              
ASRS_B.z             0.000  0.126 -0.015  0.082 -0.055 -0.086  0.692       
ASRS_Total.z         0.000 -0.069  0.051 -0.116  0.045  0.068 -0.901 -0.895
Diagnosis.z          0.000 -0.078  0.216  0.113 -0.115 -0.110 -0.055  0.062
COHS.z               0.000  0.124 -0.078  0.015  0.006 -0.138  0.077 -0.075
StimulusTypeNovel.z  0.000  0.000  0.000  0.000  0.000  0.000  0.000  0.000
                    ASRS_T Dgnss. COHS.z
Age.z                                   
Gender.z                                
Cong_Order.z                            
Stim_Order.z                            
Drive.z                                 
ASRS_A.z                                
ASRS_B.z                                
ASRS_Total.z                            
Diagnosis.z         -0.020              
COHS.z              -0.014  0.056       
StimulusTypeNovel.z  0.000  0.000  0.000

Standardized Within-Group Residuals:
        Min          Q1         Med          Q3         Max 
-3.34143067 -0.50059972 -0.04141968  0.46390871  5.14756449 

Number of Observations: 208
Number of Groups: 104
```

```
Linear mixed-effects model fit by maximum likelihood
 Data: data 
       AIC      BIC    logLik
  598.7935 662.2068 -280.3968

Random effects:
 Formula: ~1 | Subject
         (Intercept)  Residual
StdDev: 5.296124e-05 0.9315763

Fixed effects: Acc_Diff.z ~ Age.z + Gender.z + Cong_Order.z + Stim_Order.z +      Drive.z + ASRS_A.z + ASRS_B.z + ASRS_Total.z + Diagnosis.z +      COHS.z + StimulusTypeNovel.z + ASRS_A.z * StimulusTypeNovel.z +      ASRS_B.z * StimulusTypeNovel.z + ASRS_Total.z * StimulusTypeNovel.z +      Diagnosis.z * StimulusTypeNovel.z + COHS.z * StimulusTypeNovel.z 
                                      Value Std.Error DF    t-value p-value
(Intercept)                       0.0000000 0.0674065 98  0.0000000  1.0000
Age.z                            -0.1928827 0.1814569 93 -1.0629671  0.2905
Gender.z                          0.0806437 0.0743132 93  1.0851871  0.2806
Cong_Order.z                      0.0554230 0.1386425 93  0.3997549  0.6903
Stim_Order.z                     -0.0417606 0.1411464 93 -0.2958676  0.7680
Drive.z                           0.1477204 0.1786114 93  0.8270493  0.4103
ASRS_A.z                         -0.4656776 0.2026882 93 -2.2975074  0.0238
ASRS_B.z                         -0.4846481 0.1998013 93 -2.4256505  0.0172
ASRS_Total.z                      0.7820574 0.3277904 93  2.3858463  0.0191
Diagnosis.z                       0.1474151 0.0778967 93  1.8924434  0.0615
COHS.z                           -0.0611066 0.0702040 93 -0.8704156  0.3863
StimulusTypeNovel.z               0.1798932 0.0675691 98  2.6623587  0.0091
ASRS_A.z:StimulusTypeNovel.z     -0.2155653 0.1981567 98 -1.0878527  0.2793
ASRS_B.z:StimulusTypeNovel.z     -0.0233703 0.1972407 98 -0.1184863  0.9059
ASRS_Total.z:StimulusTypeNovel.z  0.1400107 0.3232504 98  0.4331340  0.6659
Diagnosis.z:StimulusTypeNovel.z   0.1521622 0.0693340 98  2.1946269  0.0306
COHS.z:StimulusTypeNovel.z        0.0537176 0.0695303 98  0.7725784  0.4416
 Correlation: 
                                 (Intr) Age.z  Gndr.z Cng_O. Stm_O. Driv.z
Age.z                             0.000                                   
Gender.z                          0.000 -0.224                            
Cong_Order.z                      0.000 -0.094  0.163                     
Stim_Order.z                      0.000  0.103 -0.256 -0.864              
Drive.z                           0.000 -0.902  0.157  0.070 -0.094       
ASRS_A.z                          0.000  0.048 -0.135  0.055  0.045 -0.046
ASRS_B.z                          0.000  0.126 -0.015  0.082 -0.055 -0.086
ASRS_Total.z                      0.000 -0.069  0.051 -0.116  0.045  0.068
Diagnosis.z                       0.000 -0.078  0.216  0.113 -0.115 -0.110
COHS.z                            0.000  0.124 -0.078  0.015  0.006 -0.138
StimulusTypeNovel.z               0.000  0.000  0.000  0.000  0.000  0.000
ASRS_A.z:StimulusTypeNovel.z      0.000  0.000  0.000  0.000  0.000  0.000
ASRS_B.z:StimulusTypeNovel.z      0.000  0.000  0.000  0.000  0.000  0.000
ASRS_Total.z:StimulusTypeNovel.z  0.000  0.000  0.000  0.000  0.000  0.000
Diagnosis.z:StimulusTypeNovel.z   0.000  0.000  0.000  0.000  0.000  0.000
COHS.z:StimulusTypeNovel.z        0.000  0.000  0.000  0.000  0.000  0.000
                                 ASRS_A.z ASRS_B.z ASRS_Tt. Dgnss. COHS.z
Age.z                                                                    
Gender.z                                                                 
Cong_Order.z                                                             
Stim_Order.z                                                             
Drive.z                                                                  
ASRS_A.z                                                                 
ASRS_B.z                          0.692                                  
ASRS_Total.z                     -0.901   -0.895                         
Diagnosis.z                      -0.055    0.062   -0.020                
COHS.z                            0.077   -0.075   -0.014    0.056       
StimulusTypeNovel.z               0.000    0.000    0.000    0.000  0.000
ASRS_A.z:StimulusTypeNovel.z      0.000    0.000    0.000    0.000  0.000
ASRS_B.z:StimulusTypeNovel.z      0.000    0.000    0.000    0.000  0.000
ASRS_Total.z:StimulusTypeNovel.z  0.000    0.000    0.000    0.000  0.000
Diagnosis.z:StimulusTypeNovel.z   0.000    0.000    0.000    0.000  0.000
COHS.z:StimulusTypeNovel.z        0.000    0.000    0.000    0.000  0.000
                                 StmTN. ASRS_A.: ASRS_B.: ASRS_T.: D.:STN
Age.z                                                                    
Gender.z                                                                 
Cong_Order.z                                                             
Stim_Order.z                                                             
Drive.z                                                                  
ASRS_A.z                                                                 
ASRS_B.z                                                                 
ASRS_Total.z                                                             
Diagnosis.z                                                              
COHS.z                                                                   
StimulusTypeNovel.z                                                      
ASRS_A.z:StimulusTypeNovel.z      0.000                                  
ASRS_B.z:StimulusTypeNovel.z      0.000  0.704                           
ASRS_Total.z:StimulusTypeNovel.z  0.000 -0.905   -0.900                  
Diagnosis.z:StimulusTypeNovel.z   0.000 -0.040    0.101   -0.022         
COHS.z:StimulusTypeNovel.z        0.000  0.060   -0.091    0.004    0.054

Standardized Within-Group Residuals:
        Min          Q1         Med          Q3         Max 
-3.50738067 -0.46632070 -0.03624827  0.42873206  5.21130899 

Number of Observations: 208
Number of Groups: 104
```

In [29]:

```
#Extract the R^2 value of each model
r.squaredGLMM(go_nofb_model1)
r.squaredGLMM(go_nofb_model2)
r.squaredGLMM(go_nofb_model3)
r.squaredGLMM(go_nofb_model4)
```

| R2m | R2c |
| --- | --- |
| 0.005631469 | 0.005631469 |

| R2m | R2c |
| --- | --- |
| 0.05975298 | 0.05975298 |

| R2m | R2c |
| --- | --- |
| 0.09224713 | 0.09224713 |

| R2m | R2c |
| --- | --- |
| 0.1285119 | 0.1285119 |

In [30]:

```
#Subtract from each other to derive delta R^2. First will be 2-1, next 3-2. 
r.squaredGLMM(go_nofb_model2) - r.squaredGLMM(go_nofb_model1)
r.squaredGLMM(go_nofb_model3) - r.squaredGLMM(go_nofb_model2)
r.squaredGLMM(go_nofb_model4) - r.squaredGLMM(go_nofb_model3)
```

| R2m | R2c |
| --- | --- |
| 0.05412151 | 0.05412151 |

| R2m | R2c |
| --- | --- |
| 0.03249415 | 0.03249415 |

| R2m | R2c |
| --- | --- |
| 0.03626475 | 0.03626475 |

In [31]:

```
#Compare models
anova(go_nofb_model1, go_nofb_model2, go_nofb_model3, go_nofb_model4)
```

|  | call | Model | df | AIC | BIC | logLik | Test | L.Ratio | p-value |
| --- | --- | --- | --- | --- | --- | --- | --- | --- | --- |
| go\_nofb\_model1 | lme.formula(fixed = Acc\_Diff ~ Age + Gender + Cong\_Order + Stim\_Order + Drive, data = myGoDay1Data, random = ~1 | Subject, method = "ML") | 1 | 8 | -374.6533 | -347.9530 | 195.3266 |  | NA | NA |
| go\_nofb\_model2 | lme.formula(fixed = Acc\_Diff ~ Age + Gender + Cong\_Order + Stim\_Order + Drive + ASRS\_A + ASRS\_B + ASRS\_Total + Diagnosis + COHS, data = myGoDay1Data, random = ~1 | Subject, method = "ML") | 2 | 13 | -376.2399 | -332.8519 | 201.1200 | 1 vs 2 | 11.586645 | 0.040912348 |
| go\_nofb\_model3 | lme.formula(fixed = Acc\_Diff ~ Age + Gender + Cong\_Order + Stim\_Order + Drive + ASRS\_A + ASRS\_B + ASRS\_Total + Diagnosis + COHS + StimulusType, data = myGoDay1Data, random = ~1 | Subject, method = "ML") | 3 | 14 | -381.5229 | -334.7973 | 204.7614 | 2 vs 3 | 7.282949 | 0.006961216 |
| go\_nofb\_model4 | lme.formula(fixed = Acc\_Diff ~ Age + Gender + Cong\_Order + Stim\_Order + Drive + ASRS\_A + ASRS\_B + ASRS\_Total + Diagnosis + COHS + StimulusType + ASRS\_A \* StimulusType + ASRS\_B \* StimulusType + ASRS\_Total \* StimulusType + Diagnosis \* StimulusType + COHS \* StimulusType, data = myGoDay1Data, random = ~1 | Subject, method = "ML") | 4 | 19 | -379.9667 | -316.5535 | 208.9834 | 3 vs 4 | 8.443863 | 0.133410699 |

### Second set of Go analysis: habit disruption, days 1 and 2, Familiar condition only¶

In [32]:

```
#Take subset of dataframe to analyze Familiar data (Feedback and NoFeedback). We'll call it "myGoDay2Data" for consistency.
myGoDay2Data <- subset(myGoData, StimulusType=="Familiar")
```

In [33]:

```
go_fb_model1 <- lme(Acc_Diff ~ Age + Gender + Cong_Order + Stim_Order + Drive, random=~1|Subject, method="ML", data=myGoDay2Data)
go_fb_model2 <- lme(Acc_Diff ~ Age + Gender + Cong_Order + Stim_Order + Drive + ASRS_A + ASRS_B + ASRS_Total + Diagnosis + COHS, random=~1|Subject, method="ML", data=myGoDay2Data)
go_fb_model3 <- lme(Acc_Diff ~ Age + Gender + Cong_Order + Stim_Order + Drive + ASRS_A + ASRS_B + ASRS_Total + Diagnosis + COHS + FeedbackCond, random=~1|Subject, method="ML", data=myGoDay2Data)
go_fb_model4 <- lme(Acc_Diff ~ Age + Gender + Cong_Order + Stim_Order + Drive + ASRS_A + ASRS_B + ASRS_Total + Diagnosis + COHS + FeedbackCond + ASRS_A*FeedbackCond + ASRS_B*FeedbackCond + ASRS_Total*FeedbackCond + Diagnosis*FeedbackCond + COHS*FeedbackCond, random=~1|Subject, method="ML", data=myGoDay2Data)
```

In [34]:

```
#Check for outliers
which(abs(residuals(go_fb_model1, type="normalized"))>3.3)
which(abs(residuals(go_fb_model2, type="normalized"))>3.3)
which(abs(residuals(go_fb_model3, type="normalized"))>3.3)
which(abs(residuals(go_fb_model4, type="normalized"))>3.3)
```

72
:   174

79
:   181

72
:   174

79
:   181

72
:   174

79
:   181

72
:   174

79
:   181

In [35]:

```
#Outliers make no difference - include in model
#myGoDay2Data <- subset(myGoDay2Data, Subject!=72 & Subject!=79)
go_fb_model1 <- lme(Acc_Diff ~ Age + Gender + Cong_Order + Stim_Order + Drive, random=~1|Subject, method="ML", data=myGoDay2Data)
go_fb_model2 <- lme(Acc_Diff ~ Age + Gender + Cong_Order + Stim_Order + Drive + ASRS_A + ASRS_B + ASRS_Total + Diagnosis + COHS, random=~1|Subject, method="ML", data=myGoDay2Data)
go_fb_model3 <- lme(Acc_Diff ~ Age + Gender + Cong_Order + Stim_Order + Drive + ASRS_A + ASRS_B + ASRS_Total + Diagnosis + COHS + FeedbackCond, random=~1|Subject, method="ML", data=myGoDay2Data)
go_fb_model4 <- lme(Acc_Diff ~ Age + Gender + Cong_Order + Stim_Order + Drive + ASRS_A + ASRS_B + ASRS_Total + Diagnosis + COHS + FeedbackCond + ASRS_A*FeedbackCond + ASRS_B*FeedbackCond + ASRS_Total*FeedbackCond + Diagnosis*FeedbackCond + COHS*FeedbackCond, random=~1|Subject, method="ML", data=myGoDay2Data)
```

In [36]:

```
#Diagnostics. plot() checks for homoscedasticity violation, qqplot() checks for normality, vif() checks for multicollinearity
qqnorm(resid(go_fb_model1))
qqnorm(resid(go_fb_model2))
qqnorm(resid(go_fb_model3))
qqnorm(resid(go_fb_model4))
plot(go_fb_model1)
plot(go_fb_model2)
plot(go_fb_model3)
plot(go_fb_model4)
```

In [37]:

```
vif(go_nofb_model1)
vif(go_nofb_model2)
vif(go_nofb_model3)
vif(go_nofb_model4)
```

Age
:   6.76773370364327

Gender
:   1.10278256254609

Cong\_Order
:   3.97919186192501

Stim\_Order
:   4.11103980111859

Drive
:   6.71293269748103

Age
:   7.21191297797033

Gender
:   1.20958269153334

Cong\_Order
:   4.21013485029773

Stim\_Order
:   4.36358183746823

Drive
:   6.98750136278284

ASRS\_A
:   8.99829578776265

ASRS\_B
:   8.74379792221166

ASRS\_Total
:   23.5340071905799

Diagnosis
:   1.32905065526235

COHS
:   1.07951043474563

Age
:   7.21191297797029

Gender
:   1.20958269153334

Cong\_Order
:   4.21013485029773

Stim\_Order
:   4.36358183746821

Drive
:   6.98750136278287

ASRS\_A
:   8.9982957877626

ASRS\_B
:   8.74379792221159

ASRS\_Total
:   23.5340071905797

Diagnosis
:   1.32905065526235

COHS
:   1.07951043474563

StimulusType
:   1

Age
:   7.21191297796994

Gender
:   1.20958269153328

Cong\_Order
:   4.21013485029758

Stim\_Order
:   4.36358183746813

Drive
:   6.98750136278259

ASRS\_A
:   17.5573961490221

ASRS\_B
:   17.2239515145636

ASRS\_Total
:   46.3105937092239

Diagnosis
:   2.37690853455821

COHS
:   2.13331112942441

StimulusType
:   64.9691076182534

ASRS\_A:StimulusType
:   91.1111347235926

ASRS\_B:StimulusType
:   71.6557128577423

ASRS\_Total:StimulusType
:   265.779061659338

Diagnosis:StimulusType
:   2.15987032322153

COHS:StimulusType
:   57.3687508002802

In [38]:

```
beta(go_fb_model1)
beta(go_fb_model2)
beta(go_fb_model3)
beta(go_fb_model4)
```

```
Linear mixed-effects model fit by maximum likelihood
 Data: data 
       AIC      BIC    logLik
  597.6792 624.3795 -290.8396

Random effects:
 Formula: ~1 | Subject
        (Intercept)  Residual
StdDev:   0.1707384 0.9647754

Fixed effects: Acc_Diff.z ~ Age.z + Gender.z + Cong_Order.z + Stim_Order.z +      Drive.z 
                  Value  Std.Error  DF    t-value p-value
(Intercept)   0.0000000 0.06997506 104  0.0000000  1.0000
Age.z        -0.0613929 0.18247836  98 -0.3364392  0.7373
Gender.z      0.0066434 0.07366050  98  0.0901888  0.9283
Cong_Order.z  0.3560088 0.13992238  98  2.5443307  0.0125
Stim_Order.z -0.2599243 0.14222161  98 -1.8276003  0.0707
Drive.z       0.0332854 0.18173806  98  0.1831503  0.8551
 Correlation: 
             (Intr) Age.z  Gndr.z Cng_O. Stm_O.
Age.z         0.000                            
Gender.z      0.000 -0.206                     
Cong_Order.z  0.000 -0.082  0.129              
Stim_Order.z  0.000  0.096 -0.212 -0.863       
Drive.z       0.000 -0.922  0.178  0.090 -0.114

Standardized Within-Group Residuals:
        Min          Q1         Med          Q3         Max 
-4.99623818 -0.47395858  0.08169038  0.58812210  4.44836472 

Number of Observations: 208
Number of Groups: 104
```

```
Linear mixed-effects model fit by maximum likelihood
 Data: data 
       AIC      BIC    logLik
  600.6138 644.0017 -287.3069

Random effects:
 Formula: ~1 | Subject
         (Intercept)  Residual
StdDev: 0.0003616944 0.9630446

Fixed effects: Acc_Diff.z ~ Age.z + Gender.z + Cong_Order.z + Stim_Order.z +      Drive.z + ASRS_A.z + ASRS_B.z + ASRS_Total.z + Diagnosis.z +      COHS.z 
                  Value Std.Error  DF    t-value p-value
(Intercept)   0.0000000 0.0686141 104  0.0000000  1.0000
Age.z        -0.1639613 0.1847077  93 -0.8876799  0.3770
Gender.z      0.0148724 0.0756445  93  0.1966094  0.8446
Cong_Order.z  0.3445313 0.1411263  93  2.4412979  0.0165
Stim_Order.z -0.2491802 0.1436751  93 -1.7343318  0.0862
Drive.z       0.1090113 0.1818113  93  0.5995849  0.5502
ASRS_A.z     -0.2333807 0.2063194  93 -1.1311622  0.2609
ASRS_B.z     -0.4210830 0.2033808  93 -2.0704168  0.0412
ASRS_Total.z  0.5230298 0.3336628  93  1.5675400  0.1204
Diagnosis.z  -0.0236383 0.0792922  93 -0.2981167  0.7663
COHS.z       -0.0836844 0.0714617  93 -1.1710385  0.2446
 Correlation: 
             (Intr) Age.z  Gndr.z Cng_O. Stm_O. Driv.z ASRS_A ASRS_B ASRS_T
Age.z         0.000                                                        
Gender.z      0.000 -0.224                                                 
Cong_Order.z  0.000 -0.094  0.163                                          
Stim_Order.z  0.000  0.103 -0.256 -0.864                                   
Drive.z       0.000 -0.902  0.157  0.070 -0.094                            
ASRS_A.z      0.000  0.048 -0.135  0.055  0.045 -0.046                     
ASRS_B.z      0.000  0.126 -0.015  0.082 -0.055 -0.086  0.692              
ASRS_Total.z  0.000 -0.069  0.051 -0.116  0.045  0.068 -0.901 -0.895       
Diagnosis.z   0.000 -0.078  0.216  0.113 -0.115 -0.110 -0.055  0.062 -0.020
COHS.z        0.000  0.124 -0.078  0.015  0.006 -0.138  0.077 -0.075 -0.014
             Dgnss.
Age.z              
Gender.z           
Cong_Order.z       
Stim_Order.z       
Drive.z            
ASRS_A.z           
ASRS_B.z           
ASRS_Total.z       
Diagnosis.z        
COHS.z        0.056

Standardized Within-Group Residuals:
         Min           Q1          Med           Q3          Max 
-5.131198387 -0.560063813  0.008018203  0.588561848  4.771032780 

Number of Observations: 208
Number of Groups: 104
```

```
Linear mixed-effects model fit by maximum likelihood
 Data: data 
       AIC      BIC    logLik
  588.9856 635.7111 -280.4928

Random effects:
 Formula: ~1 | Subject
        (Intercept)  Residual
StdDev:   0.2319847 0.9035954

Fixed effects: Acc_Diff.z ~ Age.z + Gender.z + Cong_Order.z + Stim_Order.z +      Drive.z + ASRS_A.z + ASRS_B.z + ASRS_Total.z + Diagnosis.z +      COHS.z + FeedbackCondNoFeedback.z 
                              Value Std.Error  DF   t-value p-value
(Intercept)               0.0000000 0.0686651 103  0.000000  1.0000
Age.z                    -0.1639613 0.1848449  93 -0.887021  0.3774
Gender.z                  0.0148724 0.0757007  93  0.196463  0.8447
Cong_Order.z              0.3445313 0.1412311  93  2.439486  0.0166
Stim_Order.z             -0.2491802 0.1437818  93 -1.733045  0.0864
Drive.z                   0.1090113 0.1819463  93  0.599140  0.5505
ASRS_A.z                 -0.2333807 0.2064726  93 -1.130323  0.2612
ASRS_B.z                 -0.4210830 0.2035318  93 -2.068880  0.0413
ASRS_Total.z              0.5230298 0.3339106  93  1.566377  0.1207
Diagnosis.z              -0.0236383 0.0793511  93 -0.297895  0.7664
COHS.z                   -0.0836844 0.0715148  93 -1.170169  0.2449
FeedbackCondNoFeedback.z -0.2396447 0.0646982 103 -3.704037  0.0003
 Correlation: 
                         (Intr) Age.z  Gndr.z Cng_O. Stm_O. Driv.z ASRS_A
Age.z                     0.000                                          
Gender.z                  0.000 -0.224                                   
Cong_Order.z              0.000 -0.094  0.163                            
Stim_Order.z              0.000  0.103 -0.256 -0.864                     
Drive.z                   0.000 -0.902  0.157  0.070 -0.094              
ASRS_A.z                  0.000  0.048 -0.135  0.055  0.045 -0.046       
ASRS_B.z                  0.000  0.126 -0.015  0.082 -0.055 -0.086  0.692
ASRS_Total.z              0.000 -0.069  0.051 -0.116  0.045  0.068 -0.901
Diagnosis.z               0.000 -0.078  0.216  0.113 -0.115 -0.110 -0.055
COHS.z                    0.000  0.124 -0.078  0.015  0.006 -0.138  0.077
FeedbackCondNoFeedback.z  0.000  0.000  0.000  0.000  0.000  0.000  0.000
                         ASRS_B ASRS_T Dgnss. COHS.z
Age.z                                               
Gender.z                                            
Cong_Order.z                                        
Stim_Order.z                                        
Drive.z                                             
ASRS_A.z                                            
ASRS_B.z                                            
ASRS_Total.z             -0.895                     
Diagnosis.z               0.062 -0.020              
COHS.z                   -0.075 -0.014  0.056       
FeedbackCondNoFeedback.z  0.000  0.000  0.000  0.000

Standardized Within-Group Residuals:
        Min          Q1         Med          Q3         Max 
-4.94074809 -0.46778961  0.03488434  0.47408152  4.96207009 

Number of Observations: 208
Number of Groups: 104
```

```
Linear mixed-effects model fit by maximum likelihood
 Data: data 
       AIC      BIC    logLik
  588.8555 652.2687 -275.4277

Random effects:
 Formula: ~1 | Subject
        (Intercept)  Residual
StdDev:   0.3028307 0.8606425

Fixed effects: Acc_Diff.z ~ Age.z + Gender.z + Cong_Order.z + Stim_Order.z +      Drive.z + ASRS_A.z + ASRS_B.z + ASRS_Total.z + Diagnosis.z +      COHS.z + FeedbackCondNoFeedback.z + ASRS_A.z * FeedbackCondNoFeedback.z +      ASRS_B.z * FeedbackCondNoFeedback.z + ASRS_Total.z * FeedbackCondNoFeedback.z +      Diagnosis.z * FeedbackCondNoFeedback.z + COHS.z * FeedbackCondNoFeedback.z 
                                           Value Std.Error DF   t-value p-value
(Intercept)                            0.0000000 0.0695580 98  0.000000  1.0000
Age.z                                 -0.1639613 0.1872487 93 -0.875634  0.3835
Gender.z                               0.0148724 0.0766852 93  0.193941  0.8466
Cong_Order.z                           0.3445313 0.1430677 93  2.408169  0.0180
Stim_Order.z                          -0.2491802 0.1456516 93 -1.710797  0.0905
Drive.z                                0.1090113 0.1843124 93  0.591448  0.5557
ASRS_A.z                              -0.2333807 0.2091577 93 -1.115812  0.2674
ASRS_B.z                              -0.4210830 0.2061786 93 -2.042321  0.0440
ASRS_Total.z                           0.5230298 0.3382529 93  1.546268  0.1254
Diagnosis.z                           -0.0236383 0.0803830 93 -0.294071  0.7694
COHS.z                                -0.0836844 0.0724448 93 -1.155147  0.2510
FeedbackCondNoFeedback.z              -0.2396447 0.0624241 98 -3.838974  0.0002
ASRS_A.z:FeedbackCondNoFeedback.z     -0.1250251 0.1830683 98 -0.682942  0.4963
ASRS_B.z:FeedbackCondNoFeedback.z     -0.3398210 0.1822221 98 -1.864873  0.0652
ASRS_Total.z:FeedbackCondNoFeedback.z  0.4852807 0.2986369 98  1.624986  0.1074
Diagnosis.z:FeedbackCondNoFeedback.z  -0.0003312 0.0640546 98 -0.005171  0.9959
COHS.z:FeedbackCondNoFeedback.z       -0.1007172 0.0642360 98 -1.567925  0.1201
 Correlation: 
                                      (Intr) Age.z  Gndr.z Cng_O. Stm_O. Driv.z
Age.z                                  0.000                                   
Gender.z                               0.000 -0.224                            
Cong_Order.z                           0.000 -0.094  0.163                     
Stim_Order.z                           0.000  0.103 -0.256 -0.864              
Drive.z                                0.000 -0.902  0.157  0.070 -0.094       
ASRS_A.z                               0.000  0.048 -0.135  0.055  0.045 -0.046
ASRS_B.z                               0.000  0.126 -0.015  0.082 -0.055 -0.086
ASRS_Total.z                           0.000 -0.069  0.051 -0.116  0.045  0.068
Diagnosis.z                            0.000 -0.078  0.216  0.113 -0.115 -0.110
COHS.z                                 0.000  0.124 -0.078  0.015  0.006 -0.138
FeedbackCondNoFeedback.z               0.000  0.000  0.000  0.000  0.000  0.000
ASRS_A.z:FeedbackCondNoFeedback.z      0.000  0.000  0.000  0.000  0.000  0.000
ASRS_B.z:FeedbackCondNoFeedback.z      0.000  0.000  0.000  0.000  0.000  0.000
ASRS_Total.z:FeedbackCondNoFeedback.z  0.000  0.000  0.000  0.000  0.000  0.000
Diagnosis.z:FeedbackCondNoFeedback.z   0.000  0.000  0.000  0.000  0.000  0.000
COHS.z:FeedbackCondNoFeedback.z        0.000  0.000  0.000  0.000  0.000  0.000
                                      ASRS_A.z ASRS_B.z ASRS_Tt. Dgnss. COHS.z
Age.z                                                                         
Gender.z                                                                      
Cong_Order.z                                                                  
Stim_Order.z                                                                  
Drive.z                                                                       
ASRS_A.z                                                                      
ASRS_B.z                               0.692                                  
ASRS_Total.z                          -0.901   -0.895                         
Diagnosis.z                           -0.055    0.062   -0.020                
COHS.z                                 0.077   -0.075   -0.014    0.056       
FeedbackCondNoFeedback.z               0.000    0.000    0.000    0.000  0.000
ASRS_A.z:FeedbackCondNoFeedback.z      0.000    0.000    0.000    0.000  0.000
ASRS_B.z:FeedbackCondNoFeedback.z      0.000    0.000    0.000    0.000  0.000
ASRS_Total.z:FeedbackCondNoFeedback.z  0.000    0.000    0.000    0.000  0.000
Diagnosis.z:FeedbackCondNoFeedback.z   0.000    0.000    0.000    0.000  0.000
COHS.z:FeedbackCondNoFeedback.z        0.000    0.000    0.000    0.000  0.000
                                      FdCNF. ASRS_A.: ASRS_B.: ASRS_T.: D.:FCN
Age.z                                                                         
Gender.z                                                                      
Cong_Order.z                                                                  
Stim_Order.z                                                                  
Drive.z                                                                       
ASRS_A.z                                                                      
ASRS_B.z                                                                      
ASRS_Total.z                                                                  
Diagnosis.z                                                                   
COHS.z                                                                        
FeedbackCondNoFeedback.z                                                      
ASRS_A.z:FeedbackCondNoFeedback.z      0.000                                  
ASRS_B.z:FeedbackCondNoFeedback.z      0.000  0.704                           
ASRS_Total.z:FeedbackCondNoFeedback.z  0.000 -0.905   -0.900                  
Diagnosis.z:FeedbackCondNoFeedback.z   0.000 -0.040    0.101   -0.022         
COHS.z:FeedbackCondNoFeedback.z        0.000  0.060   -0.091    0.004    0.054

Standardized Within-Group Residuals:
       Min         Q1        Med         Q3        Max 
-4.9262141 -0.4826979  0.0380961  0.4935479  4.8900729 

Number of Observations: 208
Number of Groups: 104
```

In [39]:

```
#Extract the R^2 value of each model
r.squaredGLMM(go_fb_model1)
r.squaredGLMM(go_fb_model2)
r.squaredGLMM(go_fb_model3)
r.squaredGLMM(go_fb_model4)
```

| R2m | R2c |
| --- | --- |
| 0.03558451 | 0.06487185 |

| R2m | R2c |
| --- | --- |
| 0.06837082 | 0.06837083 |

| R2m | R2c |
| --- | --- |
| 0.1260239 | 0.180068 |

| R2m | R2c |
| --- | --- |
| 0.1642271 | 0.2563038 |

In [40]:

```
#Subtract from each other to derive delta R^2. First will be 2-1, next 3-2. 
r.squaredGLMM(go_fb_model2) - r.squaredGLMM(go_fb_model1)
r.squaredGLMM(go_fb_model3) - r.squaredGLMM(go_fb_model2)
r.squaredGLMM(go_fb_model4) - r.squaredGLMM(go_fb_model3)
```

| R2m | R2c |
| --- | --- |
| 0.03278631 | 0.003498984 |

| R2m | R2c |
| --- | --- |
| 0.0576531 | 0.1116972 |

| R2m | R2c |
| --- | --- |
| 0.03820317 | 0.07623578 |

In [41]:

```
#Compare models
anova(go_fb_model1, go_fb_model2, go_fb_model3, go_fb_model4)
```

|  | call | Model | df | AIC | BIC | logLik | Test | L.Ratio | p-value |
| --- | --- | --- | --- | --- | --- | --- | --- | --- | --- |
| go\_fb\_model1 | lme.formula(fixed = Acc\_Diff ~ Age + Gender + Cong\_Order + Stim\_Order + Drive, data = myGoDay2Data, random = ~1 | Subject, method = "ML") | 1 | 8 | -620.4539 | -593.7536 | 318.2269 |  | NA | NA |
| go\_fb\_model2 | lme.formula(fixed = Acc\_Diff ~ Age + Gender + Cong\_Order + Stim\_Order + Drive + ASRS\_A + ASRS\_B + ASRS\_Total + Diagnosis + COHS, data = myGoDay2Data, random = ~1 | Subject, method = "ML") | 2 | 13 | -617.5193 | -574.1313 | 321.7596 | 1 vs 2 | 7.065421 | 0.2158201829 |
| go\_fb\_model3 | lme.formula(fixed = Acc\_Diff ~ Age + Gender + Cong\_Order + Stim\_Order + Drive + ASRS\_A + ASRS\_B + ASRS\_Total + Diagnosis + COHS + FeedbackCond, data = myGoDay2Data, random = ~1 | Subject, method = "ML") | 3 | 14 | -629.1475 | -582.4219 | 328.5737 | 2 vs 3 | 13.628184 | 0.0002228153 |
| go\_fb\_model4 | lme.formula(fixed = Acc\_Diff ~ Age + Gender + Cong\_Order + Stim\_Order + Drive + ASRS\_A + ASRS\_B + ASRS\_Total + Diagnosis + COHS + FeedbackCond + ASRS\_A \* FeedbackCond + ASRS\_B \* FeedbackCond + ASRS\_Total \* FeedbackCond + Diagnosis \* FeedbackCond + COHS \* FeedbackCond, data = myGoDay2Data, random = ~1 | Subject, method = "ML") | 4 | 19 | -629.2776 | -565.8644 | 333.6388 | 3 vs 4 | 10.130118 | 0.0716312450 |

In [ ]:

```

```
