# Supplementary material for: Habit Expression and Disruption as a Function of Attention-Deficit/Hyperactivity Disorder Symptomology
Source: Front Psychol. 2019 Sep 3;10:1997. doi: 10.3389/fpsyg.2019.01997 (PMC6733985; doi:10.3389/fpsyg.2019.01997)
Supplement: Supplementary file 1 [file Data_Sheet_1.ZIP › ASRS_Data_R_withBootstrap_Share.html]

ASRS\_Data\_R\_Share


# Habit Demonstration and Disruption as a Function of ADHD Symptom Severity study¶

# Analysis notebook¶

In [10]:

```
library(nlme)
#install.packages("MuMIn")
library(MuMIn)
#install.packages("reghelper")
library(reghelper)
#install.packages("car")
library(car)
#install.packages("stats")
library(stats)
#install.packages("lsr")
library(lsr)
#install.packages("psych")
library(psych)
#install.packages("devtools")
library(devtools)
#install_github("easyGgplot2", "kassambara")
library(easyGgplot2)
#install.packages("lme4")
library(lme4)
```

In [11]:

```
#Read csv with NoGo information (Days 1 and 2)
myNoGoData <- read.csv('Exp8_NoGo_Full.csv')
myNoGoDay1Data <- subset(myNoGoData, FeedbackCond=="NoFeedback")
```

# Analyses reported in the main text (variables adjust from pre-reg due to multicollinearity¶

## habit expression and ADHD symptomology - day 1 NoGo Acc¶

In [12]:

```
#Use ML instead of REML becuase we're concerned with comparing fixed effects between models.
nogo_nofb_model1_r <- lme(Acc_Diff ~ Gender + Cong_Order + Drive, random=~1|Subject, method="ML", data=myNoGoDay1Data)
nogo_nofb_model2_r <- lme(Acc_Diff ~ Gender + Cong_Order + Drive + ASRS_A + ASRS_B + Diagnosis + COHS, random=~1|Subject, method="ML", data=myNoGoDay1Data)
nogo_nofb_model3_r <- lme(Acc_Diff ~ Gender + Cong_Order + Drive + ASRS_A + ASRS_B + Diagnosis + COHS + StimulusType, random=~1|Subject, method="ML", data=myNoGoDay1Data)
nogo_nofb_model4_r <- lme(Acc_Diff ~ Gender + Cong_Order + Drive + ASRS_A + ASRS_B + Diagnosis + COHS + StimulusType + ASRS_A*StimulusType + ASRS_B*StimulusType + Diagnosis*StimulusType + COHS*StimulusType, random=~1|Subject, method="ML", data=myNoGoDay1Data)
```

In [13]:

```
#Check for outliers, beyond -3.3<x<3.3. No output means no outliers.
which(abs(residuals(nogo_nofb_model1_r, type="normalized")) > 3.3)
which(abs(residuals(nogo_nofb_model2_r, type="normalized")) > 3.3)
which(abs(residuals(nogo_nofb_model3_r, type="normalized")) > 3.3)
which(abs(residuals(nogo_nofb_model4_r, type="normalized")) > 3.3)
```

In [14]:

```
#Diagnostics. plot() checks for homoscedasticity violation, qqplot() checks for normality, vif() checks for multicollinearity
qqnorm(resid(nogo_nofb_model1_r))
qqnorm(resid(nogo_nofb_model2_r))
qqnorm(resid(nogo_nofb_model3_r))
qqnorm(resid(nogo_nofb_model4_r))
plot(nogo_nofb_model1_r)
plot(nogo_nofb_model2_r)
plot(nogo_nofb_model3_r)
plot(nogo_nofb_model4_r)
```

In [15]:

```
vif(nogo_nofb_model1_r)
vif(nogo_nofb_model2_r)
vif(nogo_nofb_model3_r)
vif(nogo_nofb_model4_r)
```

Gender
:   1.01476451801994

Cong\_Order
:   1.01415654061579

Drive
:   1.00383723096897

Gender
:   1.07951357845143

Cong\_Order
:   1.04126308412311

Drive
:   1.30402400280693

ASRS\_A
:   1.61665380591124

ASRS\_B
:   1.70620586645643

Diagnosis
:   1.30512798861589

COHS
:   1.06294714788362

Gender
:   1.07951357845143

Cong\_Order
:   1.04126308412311

Drive
:   1.30402400280693

ASRS\_A
:   1.61665380591124

ASRS\_B
:   1.70620586645643

Diagnosis
:   1.30512798861589

COHS
:   1.06294714788362

StimulusType
:   1

Gender
:   1.07951357845143

Cong\_Order
:   1.04126308412311

Drive
:   1.30402400280694

ASRS\_A
:   3.16772427579836

ASRS\_B
:   3.3154708063234

Diagnosis
:   2.3524610743276

COHS
:   2.11673448375455

StimulusType
:   64.792528515949

ASRS\_A:StimulusType
:   16.5110566009582

ASRS\_B:StimulusType
:   13.5979879566829

Diagnosis:StimulusType
:   2.15878860557628

COHS:StimulusType
:   57.3680235357639

In [16]:

```
#Use beta from reghelper, otherwise beta coefs won't be standardized
beta(nogo_nofb_model1_r)
beta(nogo_nofb_model2_r)
beta(nogo_nofb_model3_r)
beta(nogo_nofb_model4_r)
```

```
Linear mixed-effects model fit by maximum likelihood
 Data: data 
       AIC      BIC    logLik
  595.5241 615.5494 -291.7621

Random effects:
 Formula: ~1 | Subject
         (Intercept)  Residual
StdDev: 6.506766e-05 0.9838948

Fixed effects: Acc_Diff.z ~ Gender.z + Cong_Order.z + Drive.z 
                   Value  Std.Error  DF    t-value p-value
(Intercept)   0.00000000 0.06888642 104  0.0000000  1.0000
Gender.z     -0.14790918 0.06956051 100 -2.1263385  0.0359
Cong_Order.z -0.00605370 0.06953967 100 -0.0870539  0.9308
Drive.z       0.07976372 0.06918497 100  1.1529053  0.2517
 Correlation: 
             (Intr) Gndr.z Cng_O.
Gender.z      0.000              
Cong_Order.z  0.000 -0.110       
Drive.z       0.000 -0.045 -0.037

Standardized Within-Group Residuals:
        Min          Q1         Med          Q3         Max 
-2.85806391 -0.62931146  0.03794605  0.64593977  2.58521962 

Number of Observations: 208
Number of Groups: 104
```

```
Linear mixed-effects model fit by maximum likelihood
 Data: data 
       AIC      BIC    logLik
  602.8273 636.2027 -291.4136

Random effects:
 Formula: ~1 | Subject
         (Intercept) Residual
StdDev: 6.403148e-05 0.982248

Fixed effects: Acc_Diff.z ~ Gender.z + Cong_Order.z + Drive.z + ASRS_A.z + ASRS_B.z +      Diagnosis.z + COHS.z 
                   Value  Std.Error  DF    t-value p-value
(Intercept)   0.00000000 0.06945543 104  0.0000000  1.0000
Gender.z     -0.14439489 0.07233804  96 -1.9961129  0.0488
Cong_Order.z -0.01099693 0.07104490  96 -0.1547885  0.8773
Drive.z       0.08580893 0.07950519  96  1.0792872  0.2832
ASRS_A.z     -0.00675401 0.08852403  96 -0.0762957  0.9393
ASRS_B.z      0.04899281 0.09094281  96  0.5387211  0.5913
Diagnosis.z   0.01094491 0.07953883  96  0.1376046  0.8908
COHS.z       -0.04330044 0.07178084  96 -0.6032313  0.5478
 Correlation: 
             (Intr) Gndr.z Cng_O. Driv.z ASRS_A ASRS_B Dgnss.
Gender.z      0.000                                          
Cong_Order.z  0.000 -0.118                                   
Drive.z       0.000 -0.112 -0.073                            
ASRS_A.z      0.000 -0.178  0.114  0.011                     
ASRS_B.z      0.000  0.096 -0.152  0.165 -0.596              
Diagnosis.z   0.000  0.187  0.025 -0.422 -0.154  0.107       
COHS.z        0.000 -0.055  0.041 -0.062  0.159 -0.216  0.066

Standardized Within-Group Residuals:
       Min         Q1        Med         Q3        Max 
-2.7319821 -0.6041232  0.0595480  0.6404389  2.4855368 

Number of Observations: 208
Number of Groups: 104
```

```
Linear mixed-effects model fit by maximum likelihood
 Data: data 
       AIC      BIC    logLik
  583.2964 620.0093 -280.6482

Random effects:
 Formula: ~1 | Subject
        (Intercept) Residual
StdDev: 8.26843e-05 0.932703

Fixed effects: Acc_Diff.z ~ Gender.z + Cong_Order.z + Drive.z + ASRS_A.z + ASRS_B.z +      Diagnosis.z + COHS.z + StimulusTypeNovel.z 
                          Value  Std.Error  DF   t-value p-value
(Intercept)          0.00000000 0.06611756 103  0.000000  1.0000
Gender.z            -0.14439489 0.06886165  96 -2.096884  0.0386
Cong_Order.z        -0.01099693 0.06763065  96 -0.162603  0.8712
Drive.z              0.08580893 0.07568436  96  1.133774  0.2597
ASRS_A.z            -0.00675401 0.08426978  96 -0.080147  0.9363
ASRS_B.z             0.04899281 0.08657232  96  0.565918  0.5728
Diagnosis.z          0.01094491 0.07571639  96  0.144551  0.8854
COHS.z              -0.04330044 0.06833122  96 -0.633685  0.5278
StimulusTypeNovel.z  0.30876307 0.06627708 103  4.658671  0.0000
 Correlation: 
                    (Intr) Gndr.z Cng_O. Driv.z ASRS_A ASRS_B Dgnss. COHS.z
Gender.z             0.000                                                 
Cong_Order.z         0.000 -0.118                                          
Drive.z              0.000 -0.112 -0.073                                   
ASRS_A.z             0.000 -0.178  0.114  0.011                            
ASRS_B.z             0.000  0.096 -0.152  0.165 -0.596                     
Diagnosis.z          0.000  0.187  0.025 -0.422 -0.154  0.107              
COHS.z               0.000 -0.055  0.041 -0.062  0.159 -0.216  0.066       
StimulusTypeNovel.z  0.000  0.000  0.000  0.000  0.000  0.000  0.000  0.000

Standardized Within-Group Residuals:
        Min          Q1         Med          Q3         Max 
-3.18106498 -0.63393666  0.03172127  0.64761275  2.73355042 

Number of Observations: 208
Number of Groups: 104
```

```
Linear mixed-effects model fit by maximum likelihood
 Data: data 
       AIC      BIC    logLik
  585.1092 635.1723 -277.5546

Random effects:
 Formula: ~1 | Subject
        (Intercept)  Residual
StdDev: 0.000107266 0.9189336

Fixed effects: Acc_Diff.z ~ Gender.z + Cong_Order.z + Drive.z + ASRS_A.z + ASRS_B.z +      Diagnosis.z + COHS.z + StimulusTypeNovel.z + ASRS_A.z * StimulusTypeNovel.z +      ASRS_B.z * StimulusTypeNovel.z + Diagnosis.z * StimulusTypeNovel.z +      COHS.z * StimulusTypeNovel.z 
                                      Value  Std.Error DF   t-value p-value
(Intercept)                      0.00000000 0.06580620 99  0.000000  1.0000
Gender.z                        -0.14439489 0.06853736 96 -2.106805  0.0377
Cong_Order.z                    -0.01099693 0.06731217 96 -0.163372  0.8706
Drive.z                          0.08580893 0.07532795 96  1.139138  0.2575
ASRS_A.z                        -0.00675401 0.08387294 96 -0.080527  0.9360
ASRS_B.z                         0.04899281 0.08616464 96  0.568595  0.5710
Diagnosis.z                      0.01094491 0.07535983 96  0.145235  0.8848
COHS.z                          -0.04330044 0.06800944 96 -0.636683  0.5258
StimulusTypeNovel.z              0.30876307 0.06596496 99  4.680713  0.0000
ASRS_A.z:StimulusTypeNovel.z    -0.02371975 0.08235227 99 -0.288028  0.7739
ASRS_B.z:StimulusTypeNovel.z    -0.04722885 0.08388293 99 -0.563033  0.5747
Diagnosis.z:StimulusTypeNovel.z  0.10218688 0.06767094 99  1.510056  0.1342
COHS.z:StimulusTypeNovel.z       0.11797060 0.06787914 99  1.737951  0.0853
 Correlation: 
                                (Intr) Gndr.z Cng_O. Driv.z ASRS_A.z ASRS_B.z
Gender.z                         0.000                                       
Cong_Order.z                     0.000 -0.118                                
Drive.z                          0.000 -0.112 -0.073                         
ASRS_A.z                         0.000 -0.178  0.114  0.011                  
ASRS_B.z                         0.000  0.096 -0.152  0.165 -0.596           
Diagnosis.z                      0.000  0.187  0.025 -0.422 -0.154    0.107  
COHS.z                           0.000 -0.055  0.041 -0.062  0.159   -0.216  
StimulusTypeNovel.z              0.000  0.000  0.000  0.000  0.000    0.000  
ASRS_A.z:StimulusTypeNovel.z     0.000  0.000  0.000  0.000  0.000    0.000  
ASRS_B.z:StimulusTypeNovel.z     0.000  0.000  0.000  0.000  0.000    0.000  
Diagnosis.z:StimulusTypeNovel.z  0.000  0.000  0.000  0.000  0.000    0.000  
COHS.z:StimulusTypeNovel.z       0.000  0.000  0.000  0.000  0.000    0.000  
                                Dgnss. COHS.z StmTN. ASRS_A.: ASRS_B.: D.:STN
Gender.z                                                                     
Cong_Order.z                                                                 
Drive.z                                                                      
ASRS_A.z                                                                     
ASRS_B.z                                                                     
Diagnosis.z                                                                  
COHS.z                           0.066                                       
StimulusTypeNovel.z              0.000  0.000                                
ASRS_A.z:StimulusTypeNovel.z     0.000  0.000  0.000                         
ASRS_B.z:StimulusTypeNovel.z     0.000  0.000  0.000 -0.594                  
Diagnosis.z:StimulusTypeNovel.z  0.000  0.000  0.000 -0.143    0.186         
COHS.z:StimulusTypeNovel.z       0.000  0.000  0.000  0.149   -0.202    0.054

Standardized Within-Group Residuals:
        Min          Q1         Med          Q3         Max 
-3.21979618 -0.57288883  0.02172238  0.68720182  2.66393484 

Number of Observations: 208
Number of Groups: 104
```

In [17]:

```
if(!require(devtools)) install.packages("devtools")
devtools::install_github("aloy/lmeresampler")
packageVersion("lmeresampler")
install.packages("bootstrap")
library(bootstrap)
library(lmeresampler)
boot_nogo_nofb_model1_r<-bootstrap(model=nogo_nofb_model1_r,
                                fn=fixef,type="parametric",
                                B=1000)
boot_nogo_nofb_model2_r<-bootstrap(model=nogo_nofb_model2_r,
                                fn=fixef,type="parametric",
                                B=1000)
boot_nogo_nofb_model3_r<-bootstrap(model=nogo_nofb_model3_r,
                                fn=fixef,type="parametric",
                                B=1000)
boot_nogo_nofb_model4_r<-bootstrap(model=nogo_nofb_model4_r,
                                fn=fixef,type="parametric",
                                B=1000)
confint(boot_nogo_nofb_model1_r, level=0.95)
confint(boot_nogo_nofb_model2_r, level=0.95)
confint(boot_nogo_nofb_model3_r, level=0.95)
confint(boot_nogo_nofb_model4_r, level=0.95)
```

```
Skipping install of 'lmeresampler' from a github remote, the SHA1 (85629f03) has not changed since last install.
  Use `force = TRUE` to force installation
```

```
[1] '0.1.1'
```

```
Installing package into 'C:/Users/ahmet/Documents/R/win-library/3.5'
(as 'lib' is unspecified)
Warning message:
"unable to access index for repository http://www.stats.ox.ac.uk/pub/RWin/bin/windows/contrib/3.5:
  cannot open URL 'http://www.stats.ox.ac.uk/pub/RWin/bin/windows/contrib/3.5/PACKAGES'"
```

```
package 'bootstrap' successfully unpacked and MD5 sums checked

The downloaded binary packages are in
	C:\Users\ahmet\AppData\Local\Temp\RtmpiIfjOd\downloaded_packages
```

```
Attaching package: 'lmeresampler'

The following object is masked from 'package:bootstrap':

    bootstrap

Warning message in parametric_bootstrap.lme(model, fn, B):
"some bootstrap runs failed (73/1000)"Warning message in parametric_bootstrap.lme(model, fn, B):
"some bootstrap runs failed (79/1000)"Warning message in parametric_bootstrap.lme(model, fn, B):
"some bootstrap runs failed (81/1000)"Warning message in parametric_bootstrap.lme(model, fn, B):
"some bootstrap runs failed (82/1000)"Loading required namespace: boot
Warning message in confint.boot(boot_nogo_nofb_model1_r, level = 0.95):
"BCa method fails for this problem.  Using 'perc' instead"
```

|  | 2.5 % | 97.5 % |
| --- | --- | --- |
| (Intercept) | -0.0533166090 | 0.0534597900 |
| Gender | -0.1096339572 | -0.0029131011 |
| Cong\_Order | -0.0231891888 | 0.0179466393 |
| Drive | -0.0001677429 | 0.0007273584 |

```
Warning message in confint.boot(boot_nogo_nofb_model2_r, level = 0.95):
"BCa method fails for this problem.  Using 'perc' instead"
```

|  | 2.5 % | 97.5 % |
| --- | --- | --- |
| (Intercept) | -0.1538998905 | 0.2137419331 |
| Gender | -0.1111166142 | -0.0056243291 |
| Cong\_Order | -0.0209961866 | 0.0184084238 |
| Drive | -0.0002021782 | 0.0008282082 |
| ASRS\_A | -0.0055703254 | 0.0053334491 |
| ASRS\_B | -0.0038288003 | 0.0074511062 |
| Diagnosis | -0.1047946525 | 0.1209831034 |
| COHS | -0.0022267232 | 0.0012131178 |

```
Warning message in confint.boot(boot_nogo_nofb_model3_r, level = 0.95):
"BCa method fails for this problem.  Using 'perc' instead"
```

|  | 2.5 % | 97.5 % |
| --- | --- | --- |
| (Intercept) | -0.2004517985 | 0.1595214712 |
| Gender | -0.1054719110 | -0.0072288071 |
| Cong\_Order | -0.0212859539 | 0.0191283536 |
| Drive | -0.0002569501 | 0.0007798792 |
| ASRS\_A | -0.0053952069 | 0.0045009855 |
| ASRS\_B | -0.0033966084 | 0.0072509285 |
| Diagnosis | -0.0943488946 | 0.1081773461 |
| COHS | -0.0021940927 | 0.0010953400 |
| StimulusTypeNovel | 0.0621861726 | 0.1450958659 |

```
Warning message in confint.boot(boot_nogo_nofb_model4_r, level = 0.95):
"BCa method fails for this problem.  Using 'perc' instead"
```

|  | 2.5 % | 97.5 % |
| --- | --- | --- |
| (Intercept) | -0.1441584061 | 0.3246740437 |
| Gender | -0.1031020228 | -0.0065810714 |
| Cong\_Order | -0.0210123893 | 0.0160386579 |
| Drive | -0.0002040818 | 0.0007787908 |
| ASRS\_A | -0.0066531668 | 0.0073867230 |
| ASRS\_B | -0.0041873619 | 0.0109095830 |
| Diagnosis | -0.2131483134 | 0.0799767568 |
| COHS | -0.0040457083 | 0.0003426471 |
| StimulusTypeNovel | -0.4533767714 | 0.1949747060 |
| ASRS\_A:StimulusTypeNovel | -0.0113461710 | 0.0093870198 |
| ASRS\_B:StimulusTypeNovel | -0.0139607943 | 0.0071023650 |
| Diagnosis:StimulusTypeNovel | -0.0550639786 | 0.3357251713 |
| COHS:StimulusTypeNovel | -0.0001731045 | 0.0059282999 |

In [18]:

```
summary(nogo_nofb_model1_r)
```

```
Linear mixed-effects model fit by maximum likelihood
 Data: myNoGoDay1Data 
        AIC       BIC   logLik
  -147.0532 -127.0279 79.52658

Random effects:
 Formula: ~1 | Subject
         (Intercept)  Residual
StdDev: 6.044609e-06 0.1650878

Fixed effects: Acc_Diff ~ Gender + Cong_Order + Drive 
                  Value   Std.Error  DF    t-value p-value
(Intercept) -0.00068451 0.026996472 104 -0.0253557  0.9798
Gender      -0.05647042 0.026557586 100 -2.1263385  0.0359
Cong_Order  -0.00089947 0.010332321 100 -0.0870539  0.9308
Drive        0.00026805 0.000232497 100  1.1529053  0.2517
 Correlation: 
           (Intr) Gender Cng_Or
Gender     -0.652              
Cong_Order -0.487 -0.110       
Drive      -0.219 -0.045 -0.037

Standardized Within-Group Residuals:
        Min          Q1         Med          Q3         Max 
-2.85806392 -0.62931146  0.03794605  0.64593978  2.58521963 

Number of Observations: 208
Number of Groups: 104
```

In [20]:

```
#Extract the R^2 value of each model
r.squaredGLMM(nogo_nofb_model1_r)
r.squaredGLMM(nogo_nofb_model2_r)
r.squaredGLMM(nogo_nofb_model3_r)
r.squaredGLMM(nogo_nofb_model4_r)
```

```
Warning message:
"'r.squaredGLMM' now calculates a revised statistic. See the help page."
```

| R2m | R2c |
| --- | --- |
| 0.02740251 | 0.02740252 |

| R2m | R2c |
| --- | --- |
| 0.0306708 | 0.0306708 |

| R2m | R2c |
| --- | --- |
| 0.1263937 | 0.1263937 |

| R2m | R2c |
| --- | --- |
| 0.1521021 | 0.1521021 |

In [21]:

```
#Subtract from each other to derive delta R^2. First will be 2-1, next 3-2. 
r.squaredGLMM(nogo_nofb_model2_r) - r.squaredGLMM(nogo_nofb_model1_r)
r.squaredGLMM(nogo_nofb_model3_r) - r.squaredGLMM(nogo_nofb_model2_r)
r.squaredGLMM(nogo_nofb_model4_r) - r.squaredGLMM(nogo_nofb_model3_r)
```

| R2m | R2c |
| --- | --- |
| 0.003268284 | 0.003268283 |

| R2m | R2c |
| --- | --- |
| 0.09572286 | 0.09572286 |

| R2m | R2c |
| --- | --- |
| 0.0257084 | 0.0257084 |

In [22]:

```
#Compare the models to each other to extract log likelihood ratio Chi^2 values and the associated p-values. 
#Df is however many new variables are added to next model.
anova(nogo_nofb_model1_r, nogo_nofb_model2_r, nogo_nofb_model3_r, nogo_nofb_model4_r)
```

|  | call | Model | df | AIC | BIC | logLik | Test | L.Ratio | p-value |
| --- | --- | --- | --- | --- | --- | --- | --- | --- | --- |
| nogo\_nofb\_model1\_r | lme.formula(fixed = Acc\_Diff ~ Gender + Cong\_Order + Drive, data = myNoGoDay1Data, random = ~1 | Subject, method = "ML") | 1 | 6 | -147.0532 | -127.0279 | 79.52658 |  | NA | NA |
| nogo\_nofb\_model2\_r | lme.formula(fixed = Acc\_Diff ~ Gender + Cong\_Order + Drive + ASRS\_A + ASRS\_B + Diagnosis + COHS, data = myNoGoDay1Data, random = ~1 | Subject, method = "ML") | 2 | 10 | -139.7500 | -106.3747 | 79.87502 | 1 vs 2 | 0.6968646 | 9.517150e-01 |
| nogo\_nofb\_model3\_r | lme.formula(fixed = Acc\_Diff ~ Gender + Cong\_Order + Drive + ASRS\_A + ASRS\_B + Diagnosis + COHS + StimulusType, data = myNoGoDay1Data, random = ~1 | Subject, method = "ML") | 3 | 11 | -159.2810 | -122.5680 | 90.64048 | 2 vs 3 | 21.5309213 | 3.481692e-06 |
| nogo\_nofb\_model4\_r | lme.formula(fixed = Acc\_Diff ~ Gender + Cong\_Order + Drive + ASRS\_A + ASRS\_B + Diagnosis + COHS + StimulusType + ASRS\_A \* StimulusType + ASRS\_B \* StimulusType + Diagnosis \* StimulusType + COHS \* StimulusType, data = myNoGoDay1Data, random = ~1 | Subject, method = "ML") | 4 | 15 | -157.4681 | -107.4050 | 93.73405 | 3 vs 4 | 6.1871478 | 1.856011e-01 |

## Repeat for habit disruption data - day 2 (compared to 1) NoGo data¶

In [23]:

```
myNoGoDay2Data <- subset(myNoGoData, StimulusType=="Familiar")
nogo_fb_model1_r <- lme(Acc_Diff ~ Gender + Cong_Order + Drive, random=~1|Subject, method="ML", data=myNoGoDay2Data)
nogo_fb_model2_r <- lme(Acc_Diff ~ Gender + Cong_Order + Drive + ASRS_A + ASRS_B + Diagnosis + COHS, random=~1|Subject, method="ML", data=myNoGoDay2Data)
nogo_fb_model3_r <- lme(Acc_Diff ~ Gender + Cong_Order + Drive + ASRS_A + ASRS_B + Diagnosis + COHS + FeedbackCond, random=~1|Subject, method="ML", data=myNoGoDay2Data)
nogo_fb_model4_r <- lme(Acc_Diff ~ Gender + Cong_Order + Drive + ASRS_A + ASRS_B + Diagnosis + COHS + FeedbackCond + ASRS_A*FeedbackCond + ASRS_B*FeedbackCond + Diagnosis*FeedbackCond + COHS*FeedbackCond, random=~1|Subject, method="ML", data=myNoGoDay2Data)
```

In [24]:

```
#Check for outliers, beyond -3.3<x<3.3. No output means no outliers.
which(abs(residuals(nogo_fb_model1_r, type="normalized")) > 3.3)
which(abs(residuals(nogo_fb_model2_r, type="normalized")) > 3.3)
which(abs(residuals(nogo_fb_model3_r, type="normalized")) > 3.3)
which(abs(residuals(nogo_fb_model4_r, type="normalized")) > 3.3)
```

In [25]:

```
#Diagnostics. plot() checks for homoscedasticity violation, qqplot() checks for normality, vif() checks for multicollinearity
qqnorm(resid(nogo_fb_model1_r))
qqnorm(resid(nogo_fb_model2_r))
qqnorm(resid(nogo_fb_model3_r))
qqnorm(resid(nogo_fb_model4_r))
plot(nogo_fb_model1_r)
plot(nogo_fb_model2_r)
plot(nogo_fb_model3_r)
plot(nogo_fb_model4_r)
```

In [26]:

```
vif(nogo_fb_model1_r)
vif(nogo_fb_model2_r)
vif(nogo_fb_model3_r)
vif(nogo_fb_model4_r)
```

Gender
:   1.01476451801994

Cong\_Order
:   1.01415654061579

Drive
:   1.00383723096897

Gender
:   1.07951357845143

Cong\_Order
:   1.04126308412311

Drive
:   1.30402400280693

ASRS\_A
:   1.61665380591124

ASRS\_B
:   1.70620586645643

Diagnosis
:   1.30512798861589

COHS
:   1.06294714788362

Gender
:   1.07951357845143

Cong\_Order
:   1.04126308412311

Drive
:   1.30402400280693

ASRS\_A
:   1.61665380591124

ASRS\_B
:   1.70620586645643

Diagnosis
:   1.30512798861589

COHS
:   1.06294714788362

FeedbackCond
:   1

Gender
:   1.07951357845144

Cong\_Order
:   1.04126308412311

Drive
:   1.30402400280691

ASRS\_A
:   3.14619966372982

ASRS\_B
:   3.29313861431878

Diagnosis
:   2.33792695817091

COHS
:   2.10211080027417

FeedbackCond
:   64.79252851595

ASRS\_A:FeedbackCond
:   16.4895319888898

ASRS\_B:FeedbackCond
:   13.5756557646784

Diagnosis:FeedbackCond
:   2.14425448941961

COHS:FeedbackCond
:   57.3533998522843

In [27]:

```
beta(nogo_fb_model1_r)
beta(nogo_fb_model2_r)
beta(nogo_fb_model3_r)
beta(nogo_fb_model4_r)
```

```
Linear mixed-effects model fit by maximum likelihood
 Data: data 
       AIC      BIC    logLik
  598.4324 618.4577 -293.2162

Random effects:
 Formula: ~1 | Subject
        (Intercept)  Residual
StdDev:  9.3434e-05 0.9907974

Fixed effects: Acc_Diff.z ~ Gender.z + Cong_Order.z + Drive.z 
                   Value  Std.Error  DF    t-value p-value
(Intercept)   0.00000000 0.06936970 104  0.0000000  1.0000
Gender.z      0.04172635 0.07004851 100  0.5956779  0.5527
Cong_Order.z  0.10362198 0.07002753 100  1.4797321  0.1421
Drive.z      -0.01958506 0.06967034 100 -0.2811105  0.7792
 Correlation: 
             (Intr) Gndr.z Cng_O.
Gender.z      0.000              
Cong_Order.z  0.000 -0.110       
Drive.z       0.000 -0.045 -0.037

Standardized Within-Group Residuals:
        Min          Q1         Med          Q3         Max 
-2.77930050 -0.67232759  0.07283953  0.72933813  2.52177524 

Number of Observations: 208
Number of Groups: 104
```

```
Linear mixed-effects model fit by maximum likelihood
 Data: data 
      AIC      BIC   logLik
  603.238 636.6134 -291.619

Random effects:
 Formula: ~1 | Subject
         (Intercept)  Residual
StdDev: 6.953019e-05 0.9832184

Fixed effects: Acc_Diff.z ~ Gender.z + Cong_Order.z + Drive.z + ASRS_A.z + ASRS_B.z +      Diagnosis.z + COHS.z 
                   Value  Std.Error  DF    t-value p-value
(Intercept)   0.00000000 0.06952404 104  0.0000000  1.0000
Gender.z      0.04487993 0.07240950  96  0.6198072  0.5369
Cong_Order.z  0.08875925 0.07111508  96  1.2481073  0.2150
Drive.z       0.01950708 0.07958373  96  0.2451139  0.8069
ASRS_A.z     -0.06129631 0.08861148  96 -0.6917423  0.4908
ASRS_B.z      0.10244389 0.09103265  96  1.1253532  0.2632
Diagnosis.z  -0.04877463 0.07961741  96 -0.6126126  0.5416
COHS.z       -0.10135880 0.07185175  96 -1.4106657  0.1616
 Correlation: 
             (Intr) Gndr.z Cng_O. Driv.z ASRS_A ASRS_B Dgnss.
Gender.z      0.000                                          
Cong_Order.z  0.000 -0.118                                   
Drive.z       0.000 -0.112 -0.073                            
ASRS_A.z      0.000 -0.178  0.114  0.011                     
ASRS_B.z      0.000  0.096 -0.152  0.165 -0.596              
Diagnosis.z   0.000  0.187  0.025 -0.422 -0.154  0.107       
COHS.z        0.000 -0.055  0.041 -0.062  0.159 -0.216  0.066

Standardized Within-Group Residuals:
       Min         Q1        Med         Q3        Max 
-2.8189646 -0.6435487  0.1222429  0.6833001  2.6446726 

Number of Observations: 208
Number of Groups: 104
```

```
Linear mixed-effects model fit by maximum likelihood
 Data: data 
       AIC      BIC    logLik
  588.1394 624.8523 -283.0697

Random effects:
 Formula: ~1 | Subject
         (Intercept)  Residual
StdDev: 0.0009412662 0.9436245

Fixed effects: Acc_Diff.z ~ Gender.z + Cong_Order.z + Drive.z + ASRS_A.z + ASRS_B.z +      Diagnosis.z + COHS.z + FeedbackCondNoFeedback.z 
                               Value  Std.Error  DF   t-value p-value
(Intercept)               0.00000000 0.06689183 103  0.000000  1.0000
Gender.z                  0.04487993 0.06966805  96  0.644197  0.5210
Cong_Order.z              0.08875925 0.06842264  96  1.297221  0.1977
Drive.z                   0.01950708 0.07657066  96  0.254759  0.7995
ASRS_A.z                 -0.06129631 0.08525662  96 -0.718962  0.4739
ASRS_B.z                  0.10244389 0.08758612  96  1.169636  0.2450
Diagnosis.z              -0.04877463 0.07660306  96 -0.636719  0.5258
COHS.z                   -0.10135880 0.06913141  96 -1.466176  0.1459
FeedbackCondNoFeedback.z -0.27687353 0.06705314 103 -4.129165  0.0001
 Correlation: 
                         (Intr) Gndr.z Cng_O. Driv.z ASRS_A ASRS_B Dgnss.
Gender.z                  0.000                                          
Cong_Order.z              0.000 -0.118                                   
Drive.z                   0.000 -0.112 -0.073                            
ASRS_A.z                  0.000 -0.178  0.114  0.011                     
ASRS_B.z                  0.000  0.096 -0.152  0.165 -0.596              
Diagnosis.z               0.000  0.187  0.025 -0.422 -0.154  0.107       
COHS.z                    0.000 -0.055  0.041 -0.062  0.159 -0.216  0.066
FeedbackCondNoFeedback.z  0.000  0.000  0.000  0.000  0.000  0.000  0.000
                         COHS.z
Gender.z                       
Cong_Order.z                   
Drive.z                        
ASRS_A.z                       
ASRS_B.z                       
Diagnosis.z                    
COHS.z                         
FeedbackCondNoFeedback.z  0.000

Standardized Within-Group Residuals:
        Min          Q1         Med          Q3         Max 
-2.64453574 -0.62474559  0.03600258  0.61535912  2.79563255 

Number of Observations: 208
Number of Groups: 104
```

```
Linear mixed-effects model fit by maximum likelihood
 Data: data 
       AIC      BIC    logLik
  594.5752 644.6382 -282.2876

Random effects:
 Formula: ~1 | Subject
        (Intercept)  Residual
StdDev:  0.07858152 0.9368048

Fixed effects: Acc_Diff.z ~ Gender.z + Cong_Order.z + Drive.z + ASRS_A.z + ASRS_B.z +      Diagnosis.z + COHS.z + FeedbackCondNoFeedback.z + ASRS_A.z *      FeedbackCondNoFeedback.z + ASRS_B.z * FeedbackCondNoFeedback.z +      Diagnosis.z * FeedbackCondNoFeedback.z + COHS.z * FeedbackCondNoFeedback.z 
                                           Value  Std.Error DF   t-value
(Intercept)                           0.00000000 0.06755637 99  0.000000
Gender.z                              0.04487993 0.07036017 96  0.637860
Cong_Order.z                          0.08875925 0.06910239 96  1.284460
Drive.z                               0.01950708 0.07733135 96  0.252253
ASRS_A.z                             -0.06129631 0.08610360 96 -0.711890
ASRS_B.z                              0.10244389 0.08845625 96  1.158131
Diagnosis.z                          -0.04877463 0.07736408 96 -0.630456
COHS.z                               -0.10135880 0.06981820 96 -1.451753
FeedbackCondNoFeedback.z             -0.27687353 0.06724783 99 -4.117211
ASRS_A.z:FeedbackCondNoFeedback.z     0.05170341 0.08395384 99  0.615855
ASRS_B.z:FeedbackCondNoFeedback.z    -0.01411925 0.08551427 99 -0.165110
Diagnosis.z:FeedbackCondNoFeedback.z  0.01653684 0.06898699 99  0.239709
COHS.z:FeedbackCondNoFeedback.z      -0.05962028 0.06919923 99 -0.861574
                                     p-value
(Intercept)                           1.0000
Gender.z                              0.5251
Cong_Order.z                          0.2021
Drive.z                               0.8014
ASRS_A.z                              0.4783
ASRS_B.z                              0.2497
Diagnosis.z                           0.5299
COHS.z                                0.1498
FeedbackCondNoFeedback.z              0.0001
ASRS_A.z:FeedbackCondNoFeedback.z     0.5394
ASRS_B.z:FeedbackCondNoFeedback.z     0.8692
Diagnosis.z:FeedbackCondNoFeedback.z  0.8111
COHS.z:FeedbackCondNoFeedback.z       0.3910
 Correlation: 
                                     (Intr) Gndr.z Cng_O. Driv.z ASRS_A.z
Gender.z                              0.000                              
Cong_Order.z                          0.000 -0.118                       
Drive.z                               0.000 -0.112 -0.073                
ASRS_A.z                              0.000 -0.178  0.114  0.011         
ASRS_B.z                              0.000  0.096 -0.152  0.165 -0.596  
Diagnosis.z                           0.000  0.187  0.025 -0.422 -0.154  
COHS.z                                0.000 -0.055  0.041 -0.062  0.159  
FeedbackCondNoFeedback.z              0.000  0.000  0.000  0.000  0.000  
ASRS_A.z:FeedbackCondNoFeedback.z     0.000  0.000  0.000  0.000  0.000  
ASRS_B.z:FeedbackCondNoFeedback.z     0.000  0.000  0.000  0.000  0.000  
Diagnosis.z:FeedbackCondNoFeedback.z  0.000  0.000  0.000  0.000  0.000  
COHS.z:FeedbackCondNoFeedback.z       0.000  0.000  0.000  0.000  0.000  
                                     ASRS_B.z Dgnss. COHS.z FdCNF. ASRS_A.:
Gender.z                                                                   
Cong_Order.z                                                               
Drive.z                                                                    
ASRS_A.z                                                                   
ASRS_B.z                                                                   
Diagnosis.z                           0.107                                
COHS.z                               -0.216    0.066                       
FeedbackCondNoFeedback.z              0.000    0.000  0.000                
ASRS_A.z:FeedbackCondNoFeedback.z     0.000    0.000  0.000  0.000         
ASRS_B.z:FeedbackCondNoFeedback.z     0.000    0.000  0.000  0.000 -0.594  
Diagnosis.z:FeedbackCondNoFeedback.z  0.000    0.000  0.000  0.000 -0.143  
COHS.z:FeedbackCondNoFeedback.z       0.000    0.000  0.000  0.000  0.149  
                                     ASRS_B.: D.:FCN
Gender.z                                            
Cong_Order.z                                        
Drive.z                                             
ASRS_A.z                                            
ASRS_B.z                                            
Diagnosis.z                                         
COHS.z                                              
FeedbackCondNoFeedback.z                            
ASRS_A.z:FeedbackCondNoFeedback.z                   
ASRS_B.z:FeedbackCondNoFeedback.z                   
Diagnosis.z:FeedbackCondNoFeedback.z  0.186         
COHS.z:FeedbackCondNoFeedback.z      -0.202    0.054

Standardized Within-Group Residuals:
        Min          Q1         Med          Q3         Max 
-2.66642490 -0.61244423  0.05968763  0.64309019  2.80099620 

Number of Observations: 208
Number of Groups: 104
```

In [28]:

```
#Extract the R^2 value of each model
r.squaredGLMM(nogo_fb_model1_r)
r.squaredGLMM(nogo_fb_model2_r)
r.squaredGLMM(nogo_fb_model3_r)
r.squaredGLMM(nogo_fb_model4_r)
```

| R2m | R2c |
| --- | --- |
| 0.01364272 | 0.01364273 |

| R2m | R2c |
| --- | --- |
| 0.02874571 | 0.02874571 |

| R2m | R2c |
| --- | --- |
| 0.1057252 | 0.1057253 |

| R2m | R2c |
| --- | --- |
| 0.1124323 | 0.1186338 |

In [29]:

```
#Subtract from each other to derive delta R^2. First will be 2-1, next 3-2. 
r.squaredGLMM(nogo_fb_model2_r) - r.squaredGLMM(nogo_fb_model1_r)
r.squaredGLMM(nogo_fb_model3_r) - r.squaredGLMM(nogo_fb_model2_r)
r.squaredGLMM(nogo_fb_model4_r) - r.squaredGLMM(nogo_fb_model3_r)
```

| R2m | R2c |
| --- | --- |
| 0.01510298 | 0.01510298 |

| R2m | R2c |
| --- | --- |
| 0.07697949 | 0.07697961 |

| R2m | R2c |
| --- | --- |
| 0.006707055 | 0.01290847 |

In [30]:

```
#Compare models
anova(nogo_fb_model1_r, nogo_fb_model2_r, nogo_fb_model3_r, nogo_fb_model4_r)
```

|  | call | Model | df | AIC | BIC | logLik | Test | L.Ratio | p-value |
| --- | --- | --- | --- | --- | --- | --- | --- | --- | --- |
| nogo\_fb\_model1\_r | lme.formula(fixed = Acc\_Diff ~ Gender + Cong\_Order + Drive, data = myNoGoDay2Data, random = ~1 | Subject, method = "ML") | 1 | 6 | -133.0616 | -113.03641 | 72.53082 |  | NA | NA |
| nogo\_fb\_model2\_r | lme.formula(fixed = Acc\_Diff ~ Gender + Cong\_Order + Drive + ASRS\_A + ASRS\_B + Diagnosis + COHS, data = myNoGoDay2Data, random = ~1 | Subject, method = "ML") | 2 | 10 | -128.2560 | -94.88066 | 74.12802 | 1 vs 2 | 3.194402 | 5.258357e-01 |
| nogo\_fb\_model3\_r | lme.formula(fixed = Acc\_Diff ~ Gender + Cong\_Order + Drive + ASRS\_A + ASRS\_B + Diagnosis + COHS + FeedbackCond, data = myNoGoDay2Data, random = ~1 | Subject, method = "ML") | 3 | 11 | -143.3547 | -106.64176 | 82.67734 | 2 vs 3 | 17.098639 | 3.548773e-05 |
| nogo\_fb\_model4\_r | lme.formula(fixed = Acc\_Diff ~ Gender + Cong\_Order + Drive + ASRS\_A + ASRS\_B + Diagnosis + COHS + FeedbackCond + ASRS\_A \* FeedbackCond + ASRS\_B \* FeedbackCond + Diagnosis \* FeedbackCond + COHS \* FeedbackCond, data = myNoGoDay2Data, random = ~1 | Subject, method = "ML") | 4 | 15 | -136.9189 | -86.85585 | 83.45946 | 3 vs 4 | 1.564244 | 8.152037e-01 |

## Go analysis Day 1¶

In [31]:

```
myGoData <- read.csv('Exp8_Go_Full.csv')
myGoDay1Data <- subset(myGoData, FeedbackCond=="NoFeedback")
go_nofb_model1_r <- lme(Acc_Diff ~ Gender + Cong_Order + Drive, random=~1|Subject, method="ML", data=myGoDay1Data)
go_nofb_model2_r <- lme(Acc_Diff ~ Gender + Cong_Order + Drive + ASRS_A + ASRS_B + Diagnosis + COHS, random=~1|Subject, method="ML", data=myGoDay1Data)
go_nofb_model3_r <- lme(Acc_Diff ~ Gender + Cong_Order + Drive + ASRS_A + ASRS_B + Diagnosis + COHS + StimulusType, random=~1|Subject, method="ML", data=myGoDay1Data)
go_nofb_model4_r <- lme(Acc_Diff ~ Gender + Cong_Order + Drive + ASRS_A + ASRS_B + Diagnosis + COHS + StimulusType + ASRS_A*StimulusType + ASRS_B*StimulusType + Diagnosis*StimulusType + COHS*StimulusType, random=~1|Subject, method="ML", data=myGoDay1Data)
```

In [32]:

```
#Check for outliers, beyond -3.3<x<3.3. No output means no outliers.
which(abs(residuals(go_nofb_model1_r, type="normalized")) > 3.3)
which(abs(residuals(go_nofb_model2_r, type="normalized")) > 3.3)
which(abs(residuals(go_nofb_model3_r, type="normalized")) > 3.3)
which(abs(residuals(go_nofb_model4_r, type="normalized")) > 3.3)
```

79
:   181

106
:   208

79
:   181

106
:   208

79
:   181

106
:   208

76
:   178

79
:   181

106
:   208

In [33]:

```
#Rerun model without outliers for pub tables
myGoDay1Data_r_outrem <- subset(myGoDay1Data, Subject!=79 & Subject!=106)
go_nofb_model1_r_outrem <- lme(Acc_Diff ~ Gender + Cong_Order + Drive, random=~1|Subject, method="ML", data=myGoDay1Data_r_outrem)
go_nofb_model2_r_outrem <- lme(Acc_Diff ~ Gender + Cong_Order + Drive + ASRS_A + ASRS_B + Diagnosis + COHS, random=~1|Subject, method="ML", data=myGoDay1Data_r_outrem)
go_nofb_model3_r_outrem <- lme(Acc_Diff ~ Gender + Cong_Order + Drive + ASRS_A + ASRS_B + Diagnosis + COHS + StimulusType, random=~1|Subject, method="ML", data=myGoDay1Data_r_outrem)
go_nofb_model4_r_outrem <- lme(Acc_Diff ~ Gender + Cong_Order + Drive + ASRS_A + ASRS_B + Diagnosis + COHS + StimulusType + ASRS_A*StimulusType + ASRS_B*StimulusType + Diagnosis*StimulusType + COHS*StimulusType, random=~1|Subject, method="ML", data=myGoDay1Data_r_outrem)
```

In [34]:

```
#Diagnostics. plot() checks for homoscedasticity violation, qqplot() checks for normality, vif() checks for multicollinearity
qqnorm(resid(go_nofb_model1_r_outrem))
qqnorm(resid(go_nofb_model2_r_outrem))
qqnorm(resid(go_nofb_model3_r_outrem))
qqnorm(resid(go_nofb_model4_r_outrem))
plot(go_nofb_model1_r_outrem)
plot(go_nofb_model2_r_outrem)
plot(go_nofb_model3_r_outrem)
plot(go_nofb_model4_r_outrem)
```

In [35]:

```
vif(go_nofb_model1_r_outrem)
vif(go_nofb_model2_r_outrem)
vif(go_nofb_model3_r_outrem)
vif(go_nofb_model4_r_outrem)
```

Gender
:   1.01651302737298

Cong\_Order
:   1.0159858308694

Drive
:   1.00425208772379

Gender
:   1.08811197066133

Cong\_Order
:   1.04064577384437

Drive
:   1.30426448059456

ASRS\_A
:   1.59417599103768

ASRS\_B
:   1.65565333594042

Diagnosis
:   1.30465985115995

COHS
:   1.06523764111059

Gender
:   1.08811197066133

Cong\_Order
:   1.04064577384437

Drive
:   1.30426448059456

ASRS\_A
:   1.59417599103768

ASRS\_B
:   1.65565333594043

Diagnosis
:   1.30465985115995

COHS
:   1.06523764111059

StimulusType
:   1

Gender
:   1.08811197066129

Cong\_Order
:   1.04064577384437

Drive
:   1.30426448059452

ASRS\_A
:   3.1129430994079

ASRS\_B
:   3.22704715111562

Diagnosis
:   2.35338570290718

COHS
:   2.11973613346798

StimulusType
:   65.8352863045221

ASRS\_A:StimulusType
:   16.6470217718194

ASRS\_B:StimulusType
:   13.7124631395958

Diagnosis:StimulusType
:   2.16299706927254

COHS:StimulusType
:   57.0842624542421

In [36]:

```
beta(go_nofb_model1_r_outrem)
beta(go_nofb_model2_r_outrem)
beta(go_nofb_model3_r_outrem)
beta(go_nofb_model4_r_outrem)
```

```
Linear mixed-effects model fit by maximum likelihood
 Data: data 
       AIC      BIC    logLik
  588.0442 607.9529 -288.0221

Random effects:
 Formula: ~1 | Subject
         (Intercept)  Residual
StdDev: 8.350091e-05 0.9929593

Fixed effects: Acc_Diff.z ~ Gender.z + Cong_Order.z + Drive.z 
                   Value  Std.Error  DF    t-value p-value
(Intercept)   0.00000000 0.07021283 102  0.0000000  1.0000
Gender.z     -0.00028693 0.07096431  98 -0.0040432  0.9968
Cong_Order.z  0.07043994 0.07094591  98  0.9928683  0.3232
Drive.z       0.06180453 0.07053504  98  0.8762246  0.3831
 Correlation: 
             (Intr) Gndr.z Cng_O.
Gender.z      0.000              
Cong_Order.z  0.000 -0.117       
Drive.z       0.000 -0.046 -0.040

Standardized Within-Group Residuals:
        Min          Q1         Med          Q3         Max 
-3.38651142 -0.40707956 -0.03943542  0.42214446  3.54661646 

Number of Observations: 204
Number of Groups: 102
```

```
Linear mixed-effects model fit by maximum likelihood
 Data: data 
       AIC      BIC   logLik
  590.4881 623.6693 -285.244

Random effects:
 Formula: ~1 | Subject
         (Intercept) Residual
StdDev: 5.961434e-05 0.979529

Fixed effects: Acc_Diff.z ~ Gender.z + Cong_Order.z + Drive.z + ASRS_A.z + ASRS_B.z +      Diagnosis.z + COHS.z 
                   Value  Std.Error  DF    t-value p-value
(Intercept)   0.00000000 0.06996636 102  0.0000000  1.0000
Gender.z      0.02518214 0.07316327  94  0.3441910  0.7315
Cong_Order.z  0.08115675 0.07154970  94  1.1342710  0.2596
Drive.z      -0.01881066 0.08010123  94 -0.2348361  0.8148
ASRS_A.z      0.01931740 0.08855732  94  0.2181345  0.8278
ASRS_B.z     -0.03505251 0.09024872  94 -0.3883990  0.6986
Diagnosis.z   0.16888357 0.08011337  94  2.1080574  0.0377
COHS.z       -0.02989368 0.07239017  94 -0.4129522  0.6806
 Correlation: 
             (Intr) Gndr.z Cng_O. Driv.z ASRS_A ASRS_B Dgnss.
Gender.z      0.000                                          
Cong_Order.z  0.000 -0.126                                   
Drive.z       0.000 -0.116 -0.070                            
ASRS_A.z      0.000 -0.193  0.123  0.017                     
ASRS_B.z      0.000  0.092 -0.134  0.163 -0.583              
Diagnosis.z   0.000  0.184  0.028 -0.421 -0.151  0.109       
COHS.z        0.000 -0.068  0.049 -0.057  0.173 -0.212  0.066

Standardized Within-Group Residuals:
        Min          Q1         Med          Q3         Max 
-3.29613844 -0.42099741 -0.06278599  0.47009498  3.62092176 

Number of Observations: 204
Number of Groups: 102
```

```
Linear mixed-effects model fit by maximum likelihood
 Data: data 
       AIC     BIC    logLik
  588.0436 624.543 -283.0218

Random effects:
 Formula: ~1 | Subject
         (Intercept)  Residual
StdDev: 5.974083e-05 0.9689167

Fixed effects: Acc_Diff.z ~ Gender.z + Cong_Order.z + Drive.z + ASRS_A.z + ASRS_B.z +      Diagnosis.z + COHS.z + StimulusTypeNovel.z 
                          Value  Std.Error  DF    t-value p-value
(Intercept)          0.00000000 0.06938556 101  0.0000000  1.0000
Gender.z             0.02518214 0.07255594  94  0.3470721  0.7293
Cong_Order.z         0.08115675 0.07095576  94  1.1437655  0.2556
Drive.z             -0.01881066 0.07943630  94 -0.2368018  0.8133
ASRS_A.z             0.01931740 0.08782220  94  0.2199604  0.8264
ASRS_B.z            -0.03505251 0.08949956  94 -0.3916501  0.6962
Diagnosis.z          0.16888357 0.07944834  94  2.1257030  0.0361
COHS.z              -0.02989368 0.07178925  94 -0.4164088  0.6781
StimulusTypeNovel.z  0.14415070 0.06955625 101  2.0724333  0.0408
 Correlation: 
                    (Intr) Gndr.z Cng_O. Driv.z ASRS_A ASRS_B Dgnss. COHS.z
Gender.z             0.000                                                 
Cong_Order.z         0.000 -0.126                                          
Drive.z              0.000 -0.116 -0.070                                   
ASRS_A.z             0.000 -0.193  0.123  0.017                            
ASRS_B.z             0.000  0.092 -0.134  0.163 -0.583                     
Diagnosis.z          0.000  0.184  0.028 -0.421 -0.151  0.109              
COHS.z               0.000 -0.068  0.049 -0.057  0.173 -0.212  0.066       
StimulusTypeNovel.z  0.000  0.000  0.000  0.000  0.000  0.000  0.000  0.000

Standardized Within-Group Residuals:
         Min           Q1          Med           Q3          Max 
-3.480650371 -0.443375287 -0.009671479  0.515960913  3.512170911 

Number of Observations: 204
Number of Groups: 102
```

```
Linear mixed-effects model fit by maximum likelihood
 Data: data 
       AIC      BIC    logLik
  585.6387 635.4105 -277.8194

Random effects:
 Formula: ~1 | Subject
         (Intercept)  Residual
StdDev: 8.874519e-05 0.9445195

Fixed effects: Acc_Diff.z ~ Gender.z + Cong_Order.z + Drive.z + ASRS_A.z + ASRS_B.z +      Diagnosis.z + COHS.z + StimulusTypeNovel.z + ASRS_A.z * StimulusTypeNovel.z +      ASRS_B.z * StimulusTypeNovel.z + Diagnosis.z * StimulusTypeNovel.z +      COHS.z * StimulusTypeNovel.z 
                                      Value  Std.Error DF    t-value p-value
(Intercept)                      0.00000000 0.06834303 97  0.0000000  1.0000
Gender.z                         0.02518214 0.07146578 94  0.3523665  0.7254
Cong_Order.z                     0.08115675 0.06988964 94  1.1612129  0.2485
Drive.z                         -0.01881066 0.07824276 94 -0.2404141  0.8105
ASRS_A.z                         0.01931740 0.08650266 94  0.2233157  0.8238
ASRS_B.z                        -0.03505251 0.08815481 94 -0.3976245  0.6918
Diagnosis.z                      0.16888357 0.07825462 94  2.1581292  0.0335
COHS.z                          -0.02989368 0.07071061 94 -0.4227609  0.6734
StimulusTypeNovel.z              0.14415070 0.06851116 97  2.1040470  0.0380
ASRS_A.z:StimulusTypeNovel.z    -0.08070041 0.08463967 97 -0.9534584  0.3427
ASRS_B.z:StimulusTypeNovel.z     0.04538087 0.08609361 97  0.5271108  0.5993
Diagnosis.z:StimulusTypeNovel.z  0.19030185 0.07033304 97  2.7057249  0.0081
COHS.z:StimulusTypeNovel.z       0.10905775 0.07052634 97  1.5463406  0.1253
 Correlation: 
                                (Intr) Gndr.z Cng_O. Driv.z ASRS_A.z ASRS_B.z
Gender.z                         0.000                                       
Cong_Order.z                     0.000 -0.126                                
Drive.z                          0.000 -0.116 -0.070                         
ASRS_A.z                         0.000 -0.193  0.123  0.017                  
ASRS_B.z                         0.000  0.092 -0.134  0.163 -0.583           
Diagnosis.z                      0.000  0.184  0.028 -0.421 -0.151    0.109  
COHS.z                           0.000 -0.068  0.049 -0.057  0.173   -0.212  
StimulusTypeNovel.z              0.000  0.000  0.000  0.000  0.000    0.000  
ASRS_A.z:StimulusTypeNovel.z     0.000  0.000  0.000  0.000  0.000    0.000  
ASRS_B.z:StimulusTypeNovel.z     0.000  0.000  0.000  0.000  0.000    0.000  
Diagnosis.z:StimulusTypeNovel.z  0.000  0.000  0.000  0.000  0.000    0.000  
COHS.z:StimulusTypeNovel.z       0.000  0.000  0.000  0.000  0.000    0.000  
                                Dgnss. COHS.z StmTN. ASRS_A.: ASRS_B.: D.:STN
Gender.z                                                                     
Cong_Order.z                                                                 
Drive.z                                                                      
ASRS_A.z                                                                     
ASRS_B.z                                                                     
Diagnosis.z                                                                  
COHS.z                           0.066                                       
StimulusTypeNovel.z              0.000  0.000                                
ASRS_A.z:StimulusTypeNovel.z     0.000  0.000  0.000                         
ASRS_B.z:StimulusTypeNovel.z     0.000  0.000  0.000 -0.582                  
Diagnosis.z:StimulusTypeNovel.z  0.000  0.000  0.000 -0.136    0.189         
COHS.z:StimulusTypeNovel.z       0.000  0.000  0.000  0.160   -0.197    0.058

Standardized Within-Group Residuals:
         Min           Q1          Med           Q3          Max 
-3.536339365 -0.462987051  0.005372906  0.447981671  3.669554983 

Number of Observations: 204
Number of Groups: 102
```

In [37]:

```
#Extract the R^2 value of each model
r.squaredGLMM(go_nofb_model1_r_outrem)
r.squaredGLMM(go_nofb_model2_r_outrem)
r.squaredGLMM(go_nofb_model3_r_outrem)
r.squaredGLMM(go_nofb_model4_r_outrem)
```

| R2m | R2c |
| --- | --- |
| 0.0092196 | 0.0092196 |

| R2m | R2c |
| --- | --- |
| 0.03596644 | 0.03596644 |

| R2m | R2c |
| --- | --- |
| 0.05683873 | 0.05683873 |

| R2m | R2c |
| --- | --- |
| 0.103945 | 0.103945 |

In [38]:

```
#Subtract from each other to derive delta R^2. First will be 2-1, next 3-2. 
r.squaredGLMM(go_nofb_model2_r_outrem) - r.squaredGLMM(go_nofb_model1_r_outrem)
r.squaredGLMM(go_nofb_model3_r_outrem) - r.squaredGLMM(go_nofb_model2_r_outrem)
r.squaredGLMM(go_nofb_model4_r_outrem) - r.squaredGLMM(go_nofb_model3_r_outrem)
```

| R2m | R2c |
| --- | --- |
| 0.02674684 | 0.02674684 |

| R2m | R2c |
| --- | --- |
| 0.02087229 | 0.02087229 |

| R2m | R2c |
| --- | --- |
| 0.04710631 | 0.04710631 |

In [39]:

```
#Compare models
anova(go_nofb_model1_r_outrem, go_nofb_model2_r_outrem, go_nofb_model3_r_outrem, go_nofb_model4_r_outrem)
```

|  | call | Model | df | AIC | BIC | logLik | Test | L.Ratio | p-value |
| --- | --- | --- | --- | --- | --- | --- | --- | --- | --- |
| go\_nofb\_model1\_r\_outrem | lme.formula(fixed = Acc\_Diff ~ Gender + Cong\_Order + Drive, data = myGoDay1Data\_r\_outrem, random = ~1 | Subject, method = "ML") | 1 | 6 | -424.8888 | -404.9801 | 218.4444 |  | NA | NA |
| go\_nofb\_model2\_r\_outrem | lme.formula(fixed = Acc\_Diff ~ Gender + Cong\_Order + Drive + ASRS\_A + ASRS\_B + Diagnosis + COHS, data = myGoDay1Data\_r\_outrem, random = ~1 | Subject, method = "ML") | 2 | 10 | -422.4449 | -389.2637 | 221.2225 | 1 vs 2 | 5.556079 | 0.23484391 |
| go\_nofb\_model3\_r\_outrem | lme.formula(fixed = Acc\_Diff ~ Gender + Cong\_Order + Drive + ASRS\_A + ASRS\_B + Diagnosis + COHS + StimulusType, data = myGoDay1Data\_r\_outrem, random = ~1 | Subject, method = "ML") | 3 | 11 | -424.8894 | -388.3900 | 223.4447 | 2 vs 3 | 4.444442 | 0.03501504 |
| go\_nofb\_model4\_r\_outrem | lme.formula(fixed = Acc\_Diff ~ Gender + Cong\_Order + Drive + ASRS\_A + ASRS\_B + Diagnosis + COHS + StimulusType + ASRS\_A \* StimulusType + ASRS\_B \* StimulusType + Diagnosis \* StimulusType + COHS \* StimulusType, data = myGoDay1Data\_r\_outrem, random = ~1 | Subject, method = "ML") | 4 | 15 | -427.2943 | -377.5225 | 228.6471 | 3 vs 4 | 10.404924 | 0.03413215 |

### Post-hoc t-tests. Day 1 Go Acc¶

In [40]:

```
#Post-hoc t-tests. Compare Go Accuracy across phases (Congruency) in both Conditions
#Subject!=79 & Subject!=106 (outliers)
print("Familiar Go t-test")
myGoAccData <- read.csv("gostatssheet_full.csv")
myDay1CompareFam_outrem <- subset(myGoAccData, StimulusType=="Familiar" & FeedbackCond=="NoFeedback" & Subject!=79 & Subject!=106)
pairedSamplesTTest(formula=Accuracy~Congruency, data=myDay1CompareFam_outrem, id="Subject")
print("Novel Go t-test")
myDay1CompareNov_outrem <- subset(myGoAccData, StimulusType=="Novel" & Subject!=79 & Subject!=106)
pairedSamplesTTest(formula=Accuracy~Congruency, data=myDay1CompareNov_outrem, id="Subject")
```

```
[1] "Familiar Go t-test"
```

```
Warning message in pairedSamplesTTest(formula = Accuracy ~ Congruency, data = myDay1CompareFam_outrem, :
"id variable is not a factor"
```

```
   Paired samples t-test 

Outcome variable:   Accuracy 
Grouping variable:  Congruency 
ID variable:        Subject 

Descriptive statistics: 
            Congruent Incongruent difference
   mean         0.912       0.891      0.021
   std dev.     0.066       0.073      0.057

Hypotheses: 
   null:        population means equal for both measurements
   alternative: different population means for each measurement

Test results: 
   t-statistic:  3.805 
   degrees of freedom:  101 
   p-value:  <.001 

Other information: 
   two-sided 95% confidence interval:  [0.01, 0.033] 
   estimated effect size (Cohen's d):  0.377
```

```
[1] "Novel Go t-test"
```

```
Warning message in pairedSamplesTTest(formula = Accuracy ~ Congruency, data = myDay1CompareNov_outrem, :
"id variable is not a factor"
```

```
   Paired samples t-test 

Outcome variable:   Accuracy 
Grouping variable:  Congruency 
ID variable:        Subject 

Descriptive statistics: 
            Congruent Incongruent difference
   mean         0.890       0.893     -0.004
   std dev.     0.076       0.075      0.049

Hypotheses: 
   null:        population means equal for both measurements
   alternative: different population means for each measurement

Test results: 
   t-statistic:  -0.766 
   degrees of freedom:  101 
   p-value:  0.445 

Other information: 
   two-sided 95% confidence interval:  [-0.013, 0.006] 
   estimated effect size (Cohen's d):  0.076
```

## Second set of Go analyses: habit disruption, days 1 and 2, Familiar condition only¶

In [41]:

```
myGoDay2Data <- subset(myGoData, StimulusType=="Familiar")
go_fb_model1_r <- lme(Acc_Diff ~ Gender + Cong_Order + Drive, random=~1|Subject, method="ML", data=myGoDay2Data)
go_fb_model2_r <- lme(Acc_Diff ~ Gender + Cong_Order + Drive + ASRS_A + ASRS_B + Diagnosis + COHS, random=~1|Subject, method="ML", data=myGoDay2Data)
go_fb_model3_r <- lme(Acc_Diff ~ Gender + Cong_Order + Drive + ASRS_A + ASRS_B + Diagnosis + COHS + FeedbackCond, random=~1|Subject, method="ML", data=myGoDay2Data)
go_fb_model4_r <- lme(Acc_Diff ~ Gender + Cong_Order + Drive + ASRS_A + ASRS_B + Diagnosis + COHS + FeedbackCond + ASRS_A*FeedbackCond + ASRS_B*FeedbackCond + Diagnosis*FeedbackCond + COHS*FeedbackCond, random=~1|Subject, method="ML", data=myGoDay2Data)
```

In [42]:

```
#Check for outliers
which(abs(residuals(go_fb_model1_r, type="normalized"))>3.3)
which(abs(residuals(go_fb_model2_r, type="normalized"))>3.3)
which(abs(residuals(go_fb_model3_r, type="normalized"))>3.3)
which(abs(residuals(go_fb_model4_r, type="normalized"))>3.3)
```

72
:   174

79
:   181

72
:   174

79
:   181

72
:   174

79
:   181

72
:   174

79
:   181

In [43]:

```
#run with outliers excluded
myGoDay2Data_r_outrem <- subset(myGoDay2Data, Subject!=72 & Subject!=79)
go_fb_model1_r_outrem <- lme(Acc_Diff ~ Gender + Cong_Order + Drive, random=~1|Subject, method="ML", data=myGoDay2Data_r_outrem)
go_fb_model2_r_outrem <- lme(Acc_Diff ~ Gender + Cong_Order + Drive + ASRS_A + ASRS_B + Diagnosis + COHS, random=~1|Subject, method="ML", data=myGoDay2Data_r_outrem)
go_fb_model3_r_outrem <- lme(Acc_Diff ~ Gender + Cong_Order + Drive + ASRS_A + ASRS_B + Diagnosis + COHS + FeedbackCond, random=~1|Subject, method="ML", data=myGoDay2Data_r_outrem)
go_fb_model4_r_outrem <- lme(Acc_Diff ~ Gender + Cong_Order + Drive + ASRS_A + ASRS_B + Diagnosis + COHS + FeedbackCond + ASRS_A*FeedbackCond + ASRS_B*FeedbackCond + Diagnosis*FeedbackCond + COHS*FeedbackCond, random=~1|Subject, method="ML", data=myGoDay2Data_r_outrem)
```

In [44]:

```
#Diagnostics. plot() checks for homoscedasticity violation, qqplot() checks for normality, vif() checks for multicollinearity
qqnorm(resid(go_fb_model1_r_outrem))
qqnorm(resid(go_fb_model2_r_outrem))
qqnorm(resid(go_fb_model3_r_outrem))
qqnorm(resid(go_fb_model4_r_outrem))
plot(go_fb_model1_r_outrem)
plot(go_fb_model2_r_outrem)
plot(go_fb_model3_r_outrem)
plot(go_fb_model4_r_outrem)
```

In [45]:

```
vif(go_nofb_model1_r_outrem)
vif(go_nofb_model2_r_outrem)
vif(go_nofb_model3_r_outrem)
vif(go_nofb_model4_r_outrem)
```

Gender
:   1.01651302737298

Cong\_Order
:   1.0159858308694

Drive
:   1.00425208772379

Gender
:   1.08811197066133

Cong\_Order
:   1.04064577384437

Drive
:   1.30426448059456

ASRS\_A
:   1.59417599103768

ASRS\_B
:   1.65565333594042

Diagnosis
:   1.30465985115995

COHS
:   1.06523764111059

Gender
:   1.08811197066133

Cong\_Order
:   1.04064577384437

Drive
:   1.30426448059456

ASRS\_A
:   1.59417599103768

ASRS\_B
:   1.65565333594043

Diagnosis
:   1.30465985115995

COHS
:   1.06523764111059

StimulusType
:   1

Gender
:   1.08811197066129

Cong\_Order
:   1.04064577384437

Drive
:   1.30426448059452

ASRS\_A
:   3.1129430994079

ASRS\_B
:   3.22704715111562

Diagnosis
:   2.35338570290718

COHS
:   2.11973613346798

StimulusType
:   65.8352863045221

ASRS\_A:StimulusType
:   16.6470217718194

ASRS\_B:StimulusType
:   13.7124631395958

Diagnosis:StimulusType
:   2.16299706927254

COHS:StimulusType
:   57.0842624542421

In [46]:

```
beta(go_fb_model1_r_outrem)
beta(go_fb_model2_r_outrem)
beta(go_fb_model3_r_outrem)
beta(go_fb_model4_r_outrem)
```

```
Linear mixed-effects model fit by maximum likelihood
 Data: data 
       AIC     BIC    logLik
  586.5943 606.503 -287.2972

Random effects:
 Formula: ~1 | Subject
        (Intercept)  Residual
StdDev:   0.1676716 0.9753335

Fixed effects: Acc_Diff.z ~ Gender.z + Cong_Order.z + Drive.z 
                   Value  Std.Error  DF    t-value p-value
(Intercept)   0.00000000 0.07097545 102  0.0000000  1.0000
Gender.z     -0.02919439 0.07161729  98 -0.4076445  0.6844
Cong_Order.z  0.12139497 0.07159954  98  1.6954714  0.0932
Drive.z      -0.03511773 0.07131846  98 -0.4924072  0.6235
 Correlation: 
             (Intr) Gndr.z Cng_O.
Gender.z      0.000              
Cong_Order.z  0.000 -0.101       
Drive.z       0.000 -0.049 -0.043

Standardized Within-Group Residuals:
        Min          Q1         Med          Q3         Max 
-3.33540898 -0.58144623  0.08559325  0.57967977  3.41956014 

Number of Observations: 204
Number of Groups: 102
```

```
Linear mixed-effects model fit by maximum likelihood
 Data: data 
       AIC      BIC    logLik
  586.0322 619.2134 -283.0161

Random effects:
 Formula: ~1 | Subject
         (Intercept)  Residual
StdDev: 0.0001783826 0.9688894

Fixed effects: Acc_Diff.z ~ Gender.z + Cong_Order.z + Drive.z + ASRS_A.z + ASRS_B.z +      Diagnosis.z + COHS.z 
                   Value  Std.Error  DF    t-value p-value
(Intercept)   0.00000000 0.06920639 102  0.0000000  1.0000
Gender.z     -0.02988264 0.07207506  94 -0.4146045  0.6794
Cong_Order.z  0.13145133 0.07070306  94  1.8592028  0.0661
Drive.z      -0.05005629 0.07919223  94 -0.6320858  0.5289
ASRS_A.z      0.02374051 0.08816565  94  0.2692717  0.7883
ASRS_B.z     -0.14114643 0.09013352  94 -1.5659705  0.1207
Diagnosis.z  -0.04193152 0.07921978  94 -0.5293062  0.5978
COHS.z       -0.13952556 0.07160787  94 -1.9484670  0.0543
 Correlation: 
             (Intr) Gndr.z Cng_O. Driv.z ASRS_A ASRS_B Dgnss.
Gender.z      0.000                                          
Cong_Order.z  0.000 -0.111                                   
Drive.z       0.000 -0.115 -0.074                            
ASRS_A.z      0.000 -0.182  0.120  0.014                     
ASRS_B.z      0.000  0.103 -0.145  0.161 -0.594              
Diagnosis.z   0.000  0.187  0.025 -0.422 -0.154  0.108       
COHS.z        0.000 -0.059  0.050 -0.060  0.173 -0.217  0.064

Standardized Within-Group Residuals:
        Min          Q1         Med          Q3         Max 
-3.13005134 -0.59347185  0.05628674  0.62507000  3.77110426 

Number of Observations: 204
Number of Groups: 102
```

```
Linear mixed-effects model fit by maximum likelihood
 Data: data 
       AIC      BIC    logLik
  572.0203 608.5197 -275.0102

Random effects:
 Formula: ~1 | Subject
        (Intercept)  Residual
StdDev:   0.2376935 0.9017878

Fixed effects: Acc_Diff.z ~ Gender.z + Cong_Order.z + Drive.z + ASRS_A.z + ASRS_B.z +      Diagnosis.z + COHS.z + FeedbackCondNoFeedback.z 
                               Value  Std.Error  DF   t-value p-value
(Intercept)               0.00000000 0.06891904 101  0.000000  1.0000
Gender.z                 -0.02988264 0.07177580  94 -0.416333  0.6781
Cong_Order.z              0.13145133 0.07040950  94  1.866954  0.0650
Drive.z                  -0.05005629 0.07886342  94 -0.634721  0.5272
ASRS_A.z                  0.02374051 0.08779959  94  0.270394  0.7874
ASRS_B.z                 -0.14114643 0.08975928  94 -1.572499  0.1192
Diagnosis.z              -0.04193152 0.07889086  94 -0.531513  0.5963
COHS.z                   -0.13952556 0.07131055  94 -1.956591  0.0534
FeedbackCondNoFeedback.z -0.26337652 0.06473723 101 -4.068393  0.0001
 Correlation: 
                         (Intr) Gndr.z Cng_O. Driv.z ASRS_A ASRS_B Dgnss.
Gender.z                  0.000                                          
Cong_Order.z              0.000 -0.111                                   
Drive.z                   0.000 -0.115 -0.074                            
ASRS_A.z                  0.000 -0.182  0.120  0.014                     
ASRS_B.z                  0.000  0.103 -0.145  0.161 -0.594              
Diagnosis.z               0.000  0.187  0.025 -0.422 -0.154  0.108       
COHS.z                    0.000 -0.059  0.050 -0.060  0.173 -0.217  0.064
FeedbackCondNoFeedback.z  0.000  0.000  0.000  0.000  0.000  0.000  0.000
                         COHS.z
Gender.z                       
Cong_Order.z                   
Drive.z                        
ASRS_A.z                       
ASRS_B.z                       
Diagnosis.z                    
COHS.z                         
FeedbackCondNoFeedback.z  0.000

Standardized Within-Group Residuals:
        Min          Q1         Med          Q3         Max 
-2.93468477 -0.50986341  0.05461767  0.51579734  3.38115977 

Number of Observations: 204
Number of Groups: 102
```

```
Linear mixed-effects model fit by maximum likelihood
 Data: data 
       AIC      BIC    logLik
  570.3194 620.0912 -270.1597

Random effects:
 Formula: ~1 | Subject
        (Intercept)  Residual
StdDev:   0.3055942 0.8599082

Fixed effects: Acc_Diff.z ~ Gender.z + Cong_Order.z + Drive.z + ASRS_A.z + ASRS_B.z +      Diagnosis.z + COHS.z + FeedbackCondNoFeedback.z + ASRS_A.z *      FeedbackCondNoFeedback.z + ASRS_B.z * FeedbackCondNoFeedback.z +      Diagnosis.z * FeedbackCondNoFeedback.z + COHS.z * FeedbackCondNoFeedback.z 
                                           Value  Std.Error DF   t-value
(Intercept)                           0.00000000 0.06963697 97  0.000000
Gender.z                             -0.02988264 0.07252349 94 -0.412041
Cong_Order.z                          0.13145133 0.07114295 94  1.847707
Drive.z                              -0.05005629 0.07968494 94 -0.628178
ASRS_A.z                              0.02374051 0.08871419 94  0.267607
ASRS_B.z                             -0.14114643 0.09069430 94 -1.556288
Diagnosis.z                          -0.04193152 0.07971266 94 -0.526033
COHS.z                               -0.13952556 0.07205339 94 -1.936419
FeedbackCondNoFeedback.z             -0.26337652 0.06237384 97 -4.222548
ASRS_A.z:FeedbackCondNoFeedback.z     0.10305807 0.07771733 97  1.326063
ASRS_B.z:FeedbackCondNoFeedback.z    -0.06530351 0.07896593 97 -0.826983
Diagnosis.z:FeedbackCondNoFeedback.z  0.00131933 0.06396029 97  0.020627
COHS.z:FeedbackCondNoFeedback.z      -0.15814492 0.06423624 97 -2.461927
                                     p-value
(Intercept)                           1.0000
Gender.z                              0.6812
Cong_Order.z                          0.0678
Drive.z                               0.5314
ASRS_A.z                              0.7896
ASRS_B.z                              0.1230
Diagnosis.z                           0.6001
COHS.z                                0.0558
FeedbackCondNoFeedback.z              0.0001
ASRS_A.z:FeedbackCondNoFeedback.z     0.1879
ASRS_B.z:FeedbackCondNoFeedback.z     0.4103
Diagnosis.z:FeedbackCondNoFeedback.z  0.9836
COHS.z:FeedbackCondNoFeedback.z       0.0156
 Correlation: 
                                     (Intr) Gndr.z Cng_O. Driv.z ASRS_A.z
Gender.z                              0.000                              
Cong_Order.z                          0.000 -0.111                       
Drive.z                               0.000 -0.115 -0.074                
ASRS_A.z                              0.000 -0.182  0.120  0.014         
ASRS_B.z                              0.000  0.103 -0.145  0.161 -0.594  
Diagnosis.z                           0.000  0.187  0.025 -0.422 -0.154  
COHS.z                                0.000 -0.059  0.050 -0.060  0.173  
FeedbackCondNoFeedback.z              0.000  0.000  0.000  0.000  0.000  
ASRS_A.z:FeedbackCondNoFeedback.z     0.000  0.000  0.000  0.000  0.000  
ASRS_B.z:FeedbackCondNoFeedback.z     0.000  0.000  0.000  0.000  0.000  
Diagnosis.z:FeedbackCondNoFeedback.z  0.000  0.000  0.000  0.000  0.000  
COHS.z:FeedbackCondNoFeedback.z       0.000  0.000  0.000  0.000  0.000  
                                     ASRS_B.z Dgnss. COHS.z FdCNF. ASRS_A.:
Gender.z                                                                   
Cong_Order.z                                                               
Drive.z                                                                    
ASRS_A.z                                                                   
ASRS_B.z                                                                   
Diagnosis.z                           0.108                                
COHS.z                               -0.217    0.064                       
FeedbackCondNoFeedback.z              0.000    0.000  0.000                
ASRS_A.z:FeedbackCondNoFeedback.z     0.000    0.000  0.000  0.000         
ASRS_B.z:FeedbackCondNoFeedback.z     0.000    0.000  0.000  0.000 -0.591  
Diagnosis.z:FeedbackCondNoFeedback.z  0.000    0.000  0.000  0.000 -0.141  
COHS.z:FeedbackCondNoFeedback.z       0.000    0.000  0.000  0.000  0.162  
                                     ASRS_B.: D.:FCN
Gender.z                                            
Cong_Order.z                                        
Drive.z                                             
ASRS_A.z                                            
ASRS_B.z                                            
Diagnosis.z                                         
COHS.z                                              
FeedbackCondNoFeedback.z                            
ASRS_A.z:FeedbackCondNoFeedback.z                   
ASRS_B.z:FeedbackCondNoFeedback.z                   
Diagnosis.z:FeedbackCondNoFeedback.z  0.183         
COHS.z:FeedbackCondNoFeedback.z      -0.202    0.054

Standardized Within-Group Residuals:
        Min          Q1         Med          Q3         Max 
-2.86208752 -0.50805386  0.03919491  0.60864000  3.28946832 

Number of Observations: 204
Number of Groups: 102
```

In [47]:

```
#Extract the R^2 value of each model
r.squaredGLMM(go_fb_model1_r_outrem)
r.squaredGLMM(go_fb_model2_r_outrem)
r.squaredGLMM(go_fb_model3_r_outrem)
r.squaredGLMM(go_fb_model4_r_outrem)
```

| R2m | R2c |
| --- | --- |
| 0.01586275 | 0.04411275 |

| R2m | R2c |
| --- | --- |
| 0.05689205 | 0.05689205 |

| R2m | R2c |
| --- | --- |
| 0.1265383 | 0.1832796 |

| R2m | R2c |
| --- | --- |
| 0.1637392 | 0.2575117 |

In [48]:

```
#Subtract from each other to derive delta R^2. First will be 2-1, next 3-2. 
r.squaredGLMM(go_fb_model2_r_outrem) - r.squaredGLMM(go_fb_model1_r_outrem)
r.squaredGLMM(go_fb_model3_r_outrem) - r.squaredGLMM(go_fb_model2_r_outrem)
r.squaredGLMM(go_fb_model4_r_outrem) - r.squaredGLMM(go_fb_model3_r_outrem)
```

| R2m | R2c |
| --- | --- |
| 0.0410293 | 0.01277931 |

| R2m | R2c |
| --- | --- |
| 0.06964623 | 0.1263876 |

| R2m | R2c |
| --- | --- |
| 0.03720093 | 0.0742321 |

In [49]:

```
#Compare models
anova(go_fb_model1_r_outrem, go_fb_model2_r_outrem, go_fb_model3_r_outrem, go_fb_model4_r_outrem)
```

|  | call | Model | df | AIC | BIC | logLik | Test | L.Ratio | p-value |
| --- | --- | --- | --- | --- | --- | --- | --- | --- | --- |
| go\_fb\_model1\_r\_outrem | lme.formula(fixed = Acc\_Diff ~ Gender + Cong\_Order + Drive, data = myGoDay2Data\_r\_outrem, random = ~1 | Subject, method = "ML") | 1 | 6 | -660.7569 | -640.8482 | 336.3785 |  | NA | NA |
| go\_fb\_model2\_r\_outrem | lme.formula(fixed = Acc\_Diff ~ Gender + Cong\_Order + Drive + ASRS\_A + ASRS\_B + Diagnosis + COHS, data = myGoDay2Data\_r\_outrem, random = ~1 | Subject, method = "ML") | 2 | 10 | -661.3191 | -628.1379 | 340.6595 | 1 vs 2 | 8.562144 | 7.302576e-02 |
| go\_fb\_model3\_r\_outrem | lme.formula(fixed = Acc\_Diff ~ Gender + Cong\_Order + Drive + ASRS\_A + ASRS\_B + Diagnosis + COHS + FeedbackCond, data = myGoDay2Data\_r\_outrem, random = ~1 | Subject, method = "ML") | 3 | 11 | -675.3309 | -638.8316 | 348.6655 | 2 vs 3 | 16.011828 | 6.294799e-05 |
| go\_fb\_model4\_r\_outrem | lme.formula(fixed = Acc\_Diff ~ Gender + Cong\_Order + Drive + ASRS\_A + ASRS\_B + Diagnosis + COHS + FeedbackCond + ASRS\_A \* FeedbackCond + ASRS\_B \* FeedbackCond + Diagnosis \* FeedbackCond + COHS \* FeedbackCond, data = myGoDay2Data\_r\_outrem, random = ~1 | Subject, method = "ML") | 4 | 15 | -677.0319 | -627.2601 | 353.5159 | 3 vs 4 | 9.700939 | 4.577818e-02 |

## Correlation matrix - Go RT and surveys¶

In [50]:

```
#install.packages("corrplot")
library(corrplot)
```

```
corrplot 0.84 loaded
```

In [51]:

```
#Transfer the individual difference columns from the RT dataframe with match()
myGoRTLongData <- read.csv("gostatssheetRT_full.csv")
myGoRTData <- read.csv('Exp8_GoRT_Full.csv')
myGoRTDay1Data <- subset(myGoRTData, FeedbackCond=="NoFeedback")
myDay1FamCongData <- subset(myGoRTLongData, StimulusType=="Familiar" & FeedbackCond=="NoFeedback" & Congruency=="Congruent")
myDay1FamCongData$ASRS_A = myGoRTDay1Data[match(myDay1FamCongData$Subject, myGoRTDay1Data$Subject),"ASRS_A"]
myDay1FamCongData$ASRS_B = myGoRTDay1Data[match(myDay1FamCongData$Subject, myGoRTDay1Data$Subject),"ASRS_B"]
myDay1FamCongData$ASRS_Total = myGoRTDay1Data[match(myDay1FamCongData$Subject, myGoRTDay1Data$Subject),"ASRS_Total"]
myDay1FamCongData$COHS = myGoRTDay1Data[match(myDay1FamCongData$Subject, myGoRTDay1Data$Subject),"COHS"]
head(myDay1FamCongData)
```

|  | X | StimulusType | Congruency | FeedbackCond | Subject | RT | ASRS\_A | ASRS\_B | ASRS\_Total | COHS |
| --- | --- | --- | --- | --- | --- | --- | --- | --- | --- | --- |
| 105 | 105 | Familiar | Congruent | NoFeedback | 1 | 327.9506 | 18 | 10 | 28 | 131 |
| 106 | 106 | Familiar | Congruent | NoFeedback | 2 | 288.6598 | 12 | 10 | 22 | 111 |
| 107 | 107 | Familiar | Congruent | NoFeedback | 3 | 297.7396 | 10 | 10 | 20 | 83 |
| 108 | 108 | Familiar | Congruent | NoFeedback | 4 | 296.4783 | 18 | 13 | 31 | 105 |
| 109 | 109 | Familiar | Congruent | NoFeedback | 5 | 304.1134 | 22 | 17 | 39 | 77 |
| 110 | 110 | Familiar | Congruent | NoFeedback | 6 | 322.3548 | 8 | 18 | 26 | 105 |

In [52]:

```
#Generate correlation matrix with RT and survey measures
day1RTcorrcols <- myDay1FamCongData[,6:length(myDay1FamCongData)]
#day1RTcorrcols
#myDay1FamCongData
#corr.test automatically adjusts for multiple comparisons using Holm's method, unless you adjust="none"
corr.test(day1RTcorrcols$RT, day1RTcorrcols[,c("ASRS_A", "ASRS_B", "COHS")])[[4]]
#plot(day1RTcorrcols$ASRS_B, day1RTcorrcols$RT, main="Hyperactivity and Go RT correlation", 
#   xlab="ASRS_Hyperactivity ", ylab="Go RT in ms", pch=19)
#png(filename="RT_ASRSB_scatterplot.png", res=100)
ggplot2.scatterplot(data=day1RTcorrcols, xName='ASRS_B',yName='RT', backgroundColor="white", axisLine=c(1, "solid", "black"),
            addRegLine=TRUE, regLineColor="darkgreen", regLineSize=1, linetype="dashed", removePanelGrid=TRUE, removePanelBorder=TRUE, addConfidenceInterval=TRUE, xtitle="ASRS_Hyperactivity",
            ytitle="RT in ms") + ggtitle("Higher hyperactivity scores predict quicker Green-Go RT") +
  theme(plot.title = element_text(hjust = 0, size=rel(1.55))) + theme(axis.title.x = element_text(angle=0, hjust=0.5))
#dev.off()
```

| ASRS\_A | ASRS\_B | COHS |
| --- | --- | --- |
| 0.3465505 | 0.03233008 | 0.8489436 |

In [53]:

```
#Transfer the individual difference columns from the RT dataframe with match()
myDay1NovCongData <- subset(myGoRTLongData, StimulusType=="Novel" & Congruency=="Incongruent")
myDay1NovCongData$ASRS_A = myGoRTDay1Data[match(myDay1NovCongData$Subject, myGoRTDay1Data$Subject),"ASRS_A"]
myDay1NovCongData$ASRS_B = myGoRTDay1Data[match(myDay1NovCongData$Subject, myGoRTDay1Data$Subject),"ASRS_B"]
myDay1NovCongData$ASRS_Total = myGoRTDay1Data[match(myDay1NovCongData$Subject, myGoRTDay1Data$Subject),"ASRS_Total"]
myDay1NovCongData$COHS = myGoRTDay1Data[match(myDay1NovCongData$Subject, myGoRTDay1Data$Subject),"COHS"]
head(myDay1NovCongData)
```

|  | X | StimulusType | Congruency | FeedbackCond | Subject | RT | ASRS\_A | ASRS\_B | ASRS\_Total | COHS |
| --- | --- | --- | --- | --- | --- | --- | --- | --- | --- | --- |
| 521 | 525 | Novel | Incongruent | NoFeedback | 1 | 308.9759 | 18 | 10 | 28 | 131 |
| 522 | 526 | Novel | Incongruent | NoFeedback | 2 | 303.3100 | 12 | 10 | 22 | 111 |
| 523 | 527 | Novel | Incongruent | NoFeedback | 3 | 301.9890 | 10 | 10 | 20 | 83 |
| 524 | 528 | Novel | Incongruent | NoFeedback | 4 | 289.6593 | 18 | 13 | 31 | 105 |
| 525 | 529 | Novel | Incongruent | NoFeedback | 5 | 310.5684 | 22 | 17 | 39 | 77 |
| 526 | 530 | Novel | Incongruent | NoFeedback | 6 | 326.8427 | 8 | 18 | 26 | 105 |

In [54]:

```
panel.cor <- function(x, y, digits=2, cex.cor)
{
  usr <- par("usr"); on.exit(par(usr))
  par(usr = c(0, 1, 0, 1))
  r <- (cor(x, y))
  p <- round(corr.test(x,y)[[4]],3)
  txt <- format(c(r, 0.123456789), digits=digits)[1]
#  test <- correlate(x,y, p.adjust.method="holm")
  p <- ifelse(p<0.001,"p<0.001",paste("p=",p))
  text(0.5, 0.35, paste("r = ", txt), cex = 1.5)
  text(.5, .55, p, cex = 1.5)
}

panel.hist <- function(x, ...)
{
  usr <- par("usr"); on.exit(par(usr))
  par(usr = c(usr[1:2], 0, 1.5) )
  h <- hist(x, plot = FALSE)
  breaks <- h$breaks; nB <- length(breaks)
  y <- h$counts; y <- y/max(y)
  rect(breaks[-nB], 0, breaks[-1], y, col="gray", ...)
}
colors <- c("black", "black", "red", "black")
labelnames=c("RT", "Inattentiveness", "Hyperactivity", "COHS")
pairs(~RT+ASRS_A+ASRS_B+COHS, labels=labelnames, lower.panel=panel.cor, diag.panel=panel.hist, data=day1RTcorrcols, 
   main="Individual differences in Go RT")
```

## Extracting bootstrapped confidence interval values (and non-standardized coefficients)¶

In [55]:

```
boot_nogo_nofb_model1_r<-bootstrap(model=nogo_nofb_model1_r, fn=fixef,type="parametric", B=1000)
boot_nogo_nofb_model2_r<-bootstrap(model=nogo_nofb_model2_r, fn=fixef,type="parametric", B=1000)
boot_nogo_nofb_model3_r<-bootstrap(model=nogo_nofb_model3_r, fn=fixef,type="parametric", B=1000)
boot_nogo_nofb_model4_r<-bootstrap(model=nogo_nofb_model4_r, fn=fixef,type="parametric", B=1000)
confint(boot_nogo_nofb_model1_r, level=0.95)
confint(boot_nogo_nofb_model2_r, level=0.95)
confint(boot_nogo_nofb_model3_r, level=0.95)
confint(boot_nogo_nofb_model4_r, level=0.95)
```

```
Warning message in parametric_bootstrap.lme(model, fn, B):
"some bootstrap runs failed (67/1000)"Warning message in parametric_bootstrap.lme(model, fn, B):
"some bootstrap runs failed (69/1000)"Warning message in parametric_bootstrap.lme(model, fn, B):
"some bootstrap runs failed (89/1000)"Warning message in parametric_bootstrap.lme(model, fn, B):
"some bootstrap runs failed (80/1000)"Warning message in confint.boot(boot_nogo_nofb_model1_r, level = 0.95):
"BCa method fails for this problem.  Using 'perc' instead"
```

|  | 2.5 % | 97.5 % |
| --- | --- | --- |
| (Intercept) | -0.0493777383 | 0.0555856783 |
| Gender | -0.1042881884 | -0.0030854479 |
| Cong\_Order | -0.0205037200 | 0.0186356670 |
| Drive | -0.0001789754 | 0.0007193463 |

```
Warning message in confint.boot(boot_nogo_nofb_model2_r, level = 0.95):
"BCa method fails for this problem.  Using 'perc' instead"
```

|  | 2.5 % | 97.5 % |
| --- | --- | --- |
| (Intercept) | -0.1561461317 | 0.2060730382 |
| Gender | -0.1108535572 | -0.0012656456 |
| Cong\_Order | -0.0228429742 | 0.0164646674 |
| Drive | -0.0002188317 | 0.0007929789 |
| ASRS\_A | -0.0052646566 | 0.0049335515 |
| ASRS\_B | -0.0044675698 | 0.0070554310 |
| Diagnosis | -0.1044610723 | 0.1226397814 |
| COHS | -0.0022171814 | 0.0012018579 |

```
Warning message in confint.boot(boot_nogo_nofb_model3_r, level = 0.95):
"BCa method fails for this problem.  Using 'perc' instead"
```

|  | 2.5 % | 97.5 % |
| --- | --- | --- |
| (Intercept) | -0.1965613003 | 0.1615947842 |
| Gender | -0.1070604617 | -0.0023841204 |
| Cong\_Order | -0.0220164782 | 0.0174020544 |
| Drive | -0.0001988092 | 0.0007470675 |
| ASRS\_A | -0.0055121312 | 0.0049576884 |
| ASRS\_B | -0.0037614356 | 0.0066826687 |
| Diagnosis | -0.1013946894 | 0.1187836185 |
| COHS | -0.0022003115 | 0.0011428281 |
| StimulusTypeNovel | 0.0646164834 | 0.1451270192 |

```
Warning message in confint.boot(boot_nogo_nofb_model4_r, level = 0.95):
"BCa method fails for this problem.  Using 'perc' instead"
```

|  | 2.5 % | 97.5 % |
| --- | --- | --- |
| (Intercept) | -0.1604141837 | 0.3545895453 |
| Gender | -0.1072606753 | -0.0036065683 |
| Cong\_Order | -0.0217824444 | 0.0175146554 |
| Drive | -0.0001661029 | 0.0007580790 |
| ASRS\_A | -0.0063862003 | 0.0072044528 |
| ASRS\_B | -0.0040577577 | 0.0107411868 |
| Diagnosis | -0.2024535233 | 0.0697207219 |
| COHS | -0.0042684220 | 0.0003910813 |
| StimulusTypeNovel | -0.4902805186 | 0.2440358827 |
| ASRS\_A:StimulusTypeNovel | -0.0105528717 | 0.0079101558 |
| ASRS\_B:StimulusTypeNovel | -0.0135676979 | 0.0079519164 |
| Diagnosis:StimulusTypeNovel | -0.0385761702 | 0.3292145230 |
| COHS:StimulusTypeNovel | -0.0005667444 | 0.0063399651 |

In [56]:

```
summary(nogo_nofb_model1_r)$coefficients[1]
confint(boot_nogo_nofb_model1_r, level=0.95)
summary(nogo_nofb_model2_r)$coefficients[1]
confint(boot_nogo_nofb_model2_r, level=0.95)
summary(nogo_nofb_model3_r)$coefficients[1]
confint(boot_nogo_nofb_model3_r, level=0.95)
summary(nogo_nofb_model4_r)$coefficients[1]
confint(boot_nogo_nofb_model4_r, level=0.95)
```

**$fixed** =

(Intercept)
:   -0.000684513604438606

Gender
:   -0.0564704184003687

Cong\_Order
:   -0.000899468545217877

Drive
:   0.000268047273997946

```
Warning message in confint.boot(boot_nogo_nofb_model1_r, level = 0.95):
"BCa method fails for this problem.  Using 'perc' instead"
```

|  | 2.5 % | 97.5 % |
| --- | --- | --- |
| (Intercept) | -0.0493777383 | 0.0555856783 |
| Gender | -0.1042881884 | -0.0030854479 |
| Cong\_Order | -0.0205037200 | 0.0186356670 |
| Drive | -0.0001789754 | 0.0007193463 |

**$fixed** =

(Intercept)
:   0.0333151459519796

Gender
:   -0.0551286937635382

Cong\_Order
:   -0.0016339428651599

Drive
:   0.000288362311508415

ASRS\_A
:   -0.000209513307426661

ASRS\_B
:   0.00159264929727759

Diagnosis
:   0.00785736004320513

COHS
:   -0.000530006413623643

```
Warning message in confint.boot(boot_nogo_nofb_model2_r, level = 0.95):
"BCa method fails for this problem.  Using 'perc' instead"
```

|  | 2.5 % | 97.5 % |
| --- | --- | --- |
| (Intercept) | -0.1561461317 | 0.2060730382 |
| Gender | -0.1108535572 | -0.0012656456 |
| Cong\_Order | -0.0228429742 | 0.0164646674 |
| Drive | -0.0002188317 | 0.0007929789 |
| ASRS\_A | -0.0052646566 | 0.0049335515 |
| ASRS\_B | -0.0044675698 | 0.0070554310 |
| Diagnosis | -0.1044610723 | 0.1226397814 |
| COHS | -0.0022171814 | 0.0012018579 |

**$fixed** =

(Intercept)
:   -0.0183675463557134

Gender
:   -0.0551286937635382

Cong\_Order
:   -0.00163394286515989

Drive
:   0.000288362311508415

ASRS\_A
:   -0.000209513307426662

ASRS\_B
:   0.00159264929727759

Diagnosis
:   0.00785736004320519

COHS
:   -0.000530006413623636

StimulusTypeNovel
:   0.103365384615385

```
Warning message in confint.boot(boot_nogo_nofb_model3_r, level = 0.95):
"BCa method fails for this problem.  Using 'perc' instead"
```

|  | 2.5 % | 97.5 % |
| --- | --- | --- |
| (Intercept) | -0.1965613003 | 0.1615947842 |
| Gender | -0.1070604617 | -0.0023841204 |
| Cong\_Order | -0.0220164782 | 0.0174020544 |
| Drive | -0.0001988092 | 0.0007470675 |
| ASRS\_A | -0.0055121312 | 0.0049576884 |
| ASRS\_B | -0.0037614356 | 0.0066826687 |
| Diagnosis | -0.1013946894 | 0.1187836185 |
| COHS | -0.0022003115 | 0.0011428281 |
| StimulusTypeNovel | 0.0646164834 | 0.1451270192 |

**$fixed** =

(Intercept)
:   0.0968321124729274

Gender
:   -0.0551286937635382

Cong\_Order
:   -0.0016339428651599

Drive
:   0.000288362311508415

ASRS\_A
:   0.000524516731079181

ASRS\_B
:   0.0031242609128059

Diagnosis
:   -0.0653261288315964

COHS
:   -0.00197051568108484

StimulusTypeNovel
:   -0.127033933041896

ASRS\_A:StimulusTypeNovel
:   -0.0014680600770117

ASRS\_B:StimulusTypeNovel
:   -0.00306322323105661

Diagnosis:StimulusTypeNovel
:   0.146366977749603

COHS:StimulusTypeNovel
:   0.0028810185349224

```
Warning message in confint.boot(boot_nogo_nofb_model4_r, level = 0.95):
"BCa method fails for this problem.  Using 'perc' instead"
```

|  | 2.5 % | 97.5 % |
| --- | --- | --- |
| (Intercept) | -0.1604141837 | 0.3545895453 |
| Gender | -0.1072606753 | -0.0036065683 |
| Cong\_Order | -0.0217824444 | 0.0175146554 |
| Drive | -0.0001661029 | 0.0007580790 |
| ASRS\_A | -0.0063862003 | 0.0072044528 |
| ASRS\_B | -0.0040577577 | 0.0107411868 |
| Diagnosis | -0.2024535233 | 0.0697207219 |
| COHS | -0.0042684220 | 0.0003910813 |
| StimulusTypeNovel | -0.4902805186 | 0.2440358827 |
| ASRS\_A:StimulusTypeNovel | -0.0105528717 | 0.0079101558 |
| ASRS\_B:StimulusTypeNovel | -0.0135676979 | 0.0079519164 |
| Diagnosis:StimulusTypeNovel | -0.0385761702 | 0.3292145230 |
| COHS:StimulusTypeNovel | -0.0005667444 | 0.0063399651 |

In [57]:

```
boot_nogo_fb_model1_r<-bootstrap(model=nogo_fb_model1_r, fn=fixef,type="parametric", B=1000)
boot_nogo_fb_model2_r<-bootstrap(model=nogo_fb_model2_r, fn=fixef,type="parametric", B=1000)
boot_nogo_fb_model3_r<-bootstrap(model=nogo_fb_model3_r, fn=fixef,type="parametric", B=1000)
boot_nogo_fb_model4_r<-bootstrap(model=nogo_fb_model4_r, fn=fixef,type="parametric", B=1000)
summary(nogo_fb_model1_r)$coefficients[1]
confint(boot_nogo_fb_model1_r, level=0.95)
summary(nogo_fb_model2_r)$coefficients[1]
confint(boot_nogo_fb_model2_r, level=0.95)
summary(nogo_fb_model3_r)$coefficients[1]
confint(boot_nogo_fb_model3_r, level=0.95)
summary(nogo_fb_model4_r)$coefficients[1]
confint(boot_nogo_fb_model4_r, level=0.95)
```

```
Warning message in parametric_bootstrap.lme(model, fn, B):
"some bootstrap runs failed (70/1000)"Warning message in parametric_bootstrap.lme(model, fn, B):
"some bootstrap runs failed (58/1000)"Warning message in parametric_bootstrap.lme(model, fn, B):
"some bootstrap runs failed (73/1000)"Warning message in parametric_bootstrap.lme(model, fn, B):
"some bootstrap runs failed (71/1000)"
```

**$fixed** =

(Intercept)
:   -0.0732591459097869

Gender
:   0.0163608900900446

Cong\_Order
:   0.015812037376716

Drive
:   -6.75929874273682e-05

```
Warning message in confint.boot(boot_nogo_fb_model1_r, level = 0.95):
"BCa method fails for this problem.  Using 'perc' instead"
```

|  | 2.5 % | 97.5 % |
| --- | --- | --- |
| (Intercept) | -0.130617571 | -0.0204478456 |
| Gender | -0.034429456 | 0.0695074650 |
| Cong\_Order | -0.005935496 | 0.0378338026 |
| Drive | -0.000497196 | 0.0003650015 |

**$fixed** =

(Intercept)
:   0.0394720194019256

Gender
:   0.0175974066011086

Cong\_Order
:   0.0135440827156828

Drive
:   6.73238493850508e-05

ASRS\_A
:   -0.00195278846331064

ASRS\_B
:   0.00342014456602849

Diagnosis
:   -0.0359607801974542

COHS
:   -0.00127415091513424

```
Warning message in confint.boot(boot_nogo_fb_model2_r, level = 0.95):
"BCa method fails for this problem.  Using 'perc' instead"
```

|  | 2.5 % | 97.5 % |
| --- | --- | --- |
| (Intercept) | -0.151639831 | 0.2129794215 |
| Gender | -0.038016866 | 0.0744539359 |
| Cong\_Order | -0.006018388 | 0.0329744316 |
| Drive | -0.000495252 | 0.0005907662 |
| ASRS\_A | -0.007695163 | 0.0036610012 |
| ASRS\_B | -0.002165424 | 0.0094490773 |
| Diagnosis | -0.151956904 | 0.0719862573 |
| COHS | -0.002878448 | 0.0004786238 |

**$fixed** =

(Intercept)
:   0.0870681732480796

Gender
:   0.0175974066011086

Cong\_Order
:   0.0135440827156828

Drive
:   6.7323849385051e-05

ASRS\_A
:   -0.00195278846331064

ASRS\_B
:   0.00342014456602849

Diagnosis
:   -0.0359607801974542

COHS
:   -0.00127415091513424

FeedbackCondNoFeedback
:   -0.0951923076923076

```
Warning message in confint.boot(boot_nogo_fb_model3_r, level = 0.95):
"BCa method fails for this problem.  Using 'perc' instead"
```

|  | 2.5 % | 97.5 % |
| --- | --- | --- |
| (Intercept) | -0.0823375285 | 0.2803362479 |
| Gender | -0.0348904101 | 0.0678440380 |
| Cong\_Order | -0.0059594241 | 0.0325666806 |
| Drive | -0.0004605057 | 0.0005608322 |
| ASRS\_A | -0.0070551907 | 0.0029333350 |
| ASRS\_B | -0.0021083634 | 0.0084814669 |
| Diagnosis | -0.1359982220 | 0.0777954217 |
| COHS | -0.0029539409 | 0.0002615693 |
| FeedbackCondNoFeedback | -0.1368099293 | -0.0499303665 |

**$fixed** =

(Intercept)
:   0.0336503400399067

Gender
:   0.0175974066011086

Cong\_Order
:   0.0135440827156828

Drive
:   6.73238493850509e-05

ASRS\_A
:   -0.0035960004661936

ASRS\_B
:   0.00389038886513541

Diagnosis
:   -0.0481237899279399

COHS
:   -0.000526486067090314

FeedbackCondNoFeedback
:   0.0116433587240392

ASRS\_A:FeedbackCondNoFeedback
:   0.00328642400576591

ASRS\_B:FeedbackCondNoFeedback
:   -0.00094048859821384

Diagnosis:FeedbackCondNoFeedback
:   0.0243260194609713

COHS:FeedbackCondNoFeedback
:   -0.00149532969608786

```
Warning message in confint.boot(boot_nogo_fb_model4_r, level = 0.95):
"BCa method fails for this problem.  Using 'perc' instead"
```

|  | 2.5 % | 97.5 % |
| --- | --- | --- |
| (Intercept) | -0.2508377779 | 0.2873067608 |
| Gender | -0.0313107298 | 0.0713523152 |
| Cong\_Order | -0.0066656762 | 0.0344460376 |
| Drive | -0.0004537368 | 0.0005697439 |
| ASRS\_A | -0.0113672797 | 0.0040783851 |
| ASRS\_B | -0.0045761404 | 0.0122573720 |
| Diagnosis | -0.1826727655 | 0.0943035809 |
| COHS | -0.0030038867 | 0.0019487554 |
| FeedbackCondNoFeedback | -0.3610057762 | 0.3858154644 |
| ASRS\_A:FeedbackCondNoFeedback | -0.0067567307 | 0.0136965898 |
| ASRS\_B:FeedbackCondNoFeedback | -0.0119692130 | 0.0104708486 |
| Diagnosis:FeedbackCondNoFeedback | -0.1773185313 | 0.2076448268 |
| COHS:FeedbackCondNoFeedback | -0.0049201020 | 0.0019001076 |

In [58]:

```
boot_go_nofb_model1_r<-bootstrap(model=go_nofb_model1_r, fn=fixef,type="parametric", B=1000)
boot_go_nofb_model2_r<-bootstrap(model=go_nofb_model2_r, fn=fixef,type="parametric", B=1000)
boot_go_nofb_model3_r<-bootstrap(model=go_nofb_model3_r, fn=fixef,type="parametric", B=1000)
boot_go_nofb_model4_r<-bootstrap(model=go_nofb_model4_r, fn=fixef,type="parametric", B=1000)
summary(go_nofb_model1_r)$coefficients[1]
confint(boot_go_nofb_model1_r, level=0.95)
summary(go_nofb_model2_r)$coefficients[1]
confint(boot_go_nofb_model2_r, level=0.95)
summary(go_nofb_model3_r)$coefficients[1]
confint(boot_go_nofb_model3_r, level=0.95)
summary(go_nofb_model4_r)$coefficients[1]
confint(boot_go_nofb_model4_r, level=0.95)
```

```
Warning message in parametric_bootstrap.lme(model, fn, B):
"some bootstrap runs failed (109/1000)"Warning message in parametric_bootstrap.lme(model, fn, B):
"some bootstrap runs failed (105/1000)"Warning message in parametric_bootstrap.lme(model, fn, B):
"some bootstrap runs failed (85/1000)"Warning message in parametric_bootstrap.lme(model, fn, B):
"some bootstrap runs failed (84/1000)"
```

**$fixed** =

(Intercept)
:   -0.0174219161522281

Gender
:   0.00486053134397173

Cong\_Order
:   0.00292780376928215

Drive
:   9.7746118210836e-05

```
Warning message in confint.boot(boot_go_nofb_model1_r, level = 0.95):
"BCa method fails for this problem.  Using 'perc' instead"
```

|  | 2.5 % | 97.5 % |
| --- | --- | --- |
| (Intercept) | -0.0457462474 | 0.0137118835 |
| Gender | -0.0263476243 | 0.0344665690 |
| Cong\_Order | -0.0088384476 | 0.0136283351 |
| Drive | -0.0001786724 | 0.0003453361 |

**$fixed** =

(Intercept)
:   0.0323351885600753

Gender
:   0.0111041666973382

Cong\_Order
:   0.00373407515828195

Drive
:   -4.9131659748386e-05

ASRS\_A
:   -0.00047495334308369

ASRS\_B
:   -0.000885939370142808

Diagnosis
:   0.0581709685515789

COHS
:   -0.000355109670240959

```
Warning message in confint.boot(boot_go_nofb_model2_r, level = 0.95):
"BCa method fails for this problem.  Using 'perc' instead"
```

|  | 2.5 % | 97.5 % |
| --- | --- | --- |
| (Intercept) | -0.0693473259 | 0.1374519594 |
| Gender | -0.0196666164 | 0.0420844091 |
| Cong\_Order | -0.0080373235 | 0.0144612360 |
| Drive | -0.0003120989 | 0.0002535544 |
| ASRS\_A | -0.0034679332 | 0.0024718892 |
| ASRS\_B | -0.0042855049 | 0.0024170045 |
| Diagnosis | -0.0066991276 | 0.1232882488 |
| COHS | -0.0013055575 | 0.0005794607 |

**$fixed** =

(Intercept)
:   0.0152678808677675

Gender
:   0.0111041666973382

Cong\_Order
:   0.00373407515828194

Drive
:   -4.9131659748386e-05

ASRS\_A
:   -0.000474953343083689

ASRS\_B
:   -0.000885939370142809

Diagnosis
:   0.0581709685515789

COHS
:   -0.000355109670240958

StimulusTypeNovel
:   0.0341346153846154

```
Warning message in confint.boot(boot_go_nofb_model3_r, level = 0.95):
"BCa method fails for this problem.  Using 'perc' instead"
```

|  | 2.5 % | 97.5 % |
| --- | --- | --- |
| (Intercept) | -0.0961514452 | 0.1182099513 |
| Gender | -0.0189166407 | 0.0398265830 |
| Cong\_Order | -0.0078298529 | 0.0151099877 |
| Drive | -0.0003424833 | 0.0002238832 |
| ASRS\_A | -0.0034159929 | 0.0024857324 |
| ASRS\_B | -0.0040724951 | 0.0021938147 |
| Diagnosis | -0.0044267180 | 0.1216213529 |
| COHS | -0.0013063107 | 0.0006399752 |
| StimulusTypeNovel | 0.0074907915 | 0.0598519221 |

**$fixed** =

(Intercept)
:   0.0300818706943473

Gender
:   0.0111041666973382

Cong\_Order
:   0.00373407515828194

Drive
:   -4.91316597483859e-05

ASRS\_A
:   0.00194387633276752

ASRS\_B
:   -0.00186986476489177

Diagnosis
:   -0.00386862957306658

COHS
:   -0.000726150332588014

StimulusTypeNovel
:   0.004506635731456

ASRS\_A:StimulusTypeNovel
:   -0.00483765935170242

ASRS\_B:StimulusTypeNovel
:   0.00196785078949793

Diagnosis:StimulusTypeNovel
:   0.124079196249291

COHS:StimulusTypeNovel
:   0.00074208132469411

```
Warning message in confint.boot(boot_go_nofb_model4_r, level = 0.95):
"BCa method fails for this problem.  Using 'perc' instead"
```

|  | 2.5 % | 97.5 % |
| --- | --- | --- |
| (Intercept) | -0.1127701923 | 0.1667279015 |
| Gender | -0.0178016091 | 0.0417764652 |
| Cong\_Order | -0.0074727612 | 0.0139575898 |
| Drive | -0.0003113695 | 0.0002364362 |
| ASRS\_A | -0.0023344650 | 0.0058645480 |
| ASRS\_B | -0.0059315453 | 0.0027762588 |
| Diagnosis | -0.0837346285 | 0.0710962532 |
| COHS | -0.0020339383 | 0.0005369558 |
| StimulusTypeNovel | -0.1894895041 | 0.2068286757 |
| ASRS\_A:StimulusTypeNovel | -0.0104153777 | 0.0009987766 |
| ASRS\_B:StimulusTypeNovel | -0.0040198015 | 0.0081640348 |
| Diagnosis:StimulusTypeNovel | 0.0139167190 | 0.2263566468 |
| COHS:StimulusTypeNovel | -0.0010959825 | 0.0026957965 |

In [59]:

```
boot_go_fb_model1_r<-bootstrap(model=go_fb_model1_r, fn=fixef,type="parametric", B=1000)
boot_go_fb_model2_r<-bootstrap(model=go_fb_model2_r, fn=fixef,type="parametric", B=1000)
boot_go_fb_model3_r<-bootstrap(model=go_fb_model3_r, fn=fixef,type="parametric", B=1000)
boot_go_fb_model4_r<-bootstrap(model=go_fb_model4_r, fn=fixef,type="parametric", B=1000)
summary(go_fb_model1_r)$coefficients[1]
confint(boot_go_fb_model1_r, level=0.95)
summary(go_fb_model2_r)$coefficients[1]
confint(boot_go_fb_model2_r, level=0.95)
summary(go_fb_model3_r)$coefficients[1]
confint(boot_go_fb_model3_r, level=0.95)
summary(go_fb_model4_r)$coefficients[1]
confint(boot_go_fb_model4_r, level=0.95)
```

```
Warning message in parametric_bootstrap.lme(model, fn, B):
"some bootstrap runs failed (110/1000)"Warning message in parametric_bootstrap.lme(model, fn, B):
"some bootstrap runs failed (126/1000)"Warning message in parametric_bootstrap.lme(model, fn, B):
"some bootstrap runs failed (106/1000)"Warning message in parametric_bootstrap.lme(model, fn, B):
"some bootstrap runs failed (139/1000)"
```

**$fixed** =

(Intercept)
:   -0.0170054371463556

Gender
:   -0.00293738132509628

Cong\_Order
:   0.00640881018012359

Drive
:   -3.37286237972177e-05

```
Warning message in confint.boot(boot_go_fb_model1_r, level = 0.95):
"BCa method fails for this problem.  Using 'perc' instead"
```

|  | 2.5 % | 97.5 % |
| --- | --- | --- |
| (Intercept) | -3.479489e-02 | 0.0009499469 |
| Gender | -1.941349e-02 | 0.0140189290 |
| Cong\_Order | -6.998292e-05 | 0.0131844642 |
| Drive | -1.838916e-04 | 0.0001116499 |

**$fixed** =

(Intercept)
:   0.0175877944470712

Gender
:   -0.00429549591148491

Cong\_Order
:   0.0071073492484185

Drive
:   -4.13982850130473e-05

ASRS\_A
:   0.000860463751304387

ASRS\_B
:   -0.00138414940594271

Diagnosis
:   -0.00931123160922751

COHS
:   -0.000297009995489315

```
Warning message in confint.boot(boot_go_fb_model2_r, level = 0.95):
"BCa method fails for this problem.  Using 'perc' instead"
```

|  | 2.5 % | 97.5 % |
| --- | --- | --- |
| (Intercept) | -0.0374800191 | 0.0769254143 |
| Gender | -0.0203332408 | 0.0132725713 |
| Cong\_Order | 0.0006890824 | 0.0134691349 |
| Drive | -0.0002013369 | 0.0001244892 |
| ASRS\_A | -0.0006746151 | 0.0026194948 |
| ASRS\_B | -0.0030965877 | 0.0001821130 |
| Diagnosis | -0.0441484563 | 0.0257914417 |
| COHS | -0.0008437561 | 0.0002190157 |

**$fixed** =

(Intercept)
:   0.0303762559855328

Gender
:   -0.0042954959114849

Cong\_Order
:   0.00710734924841851

Drive
:   -4.13982850130473e-05

ASRS\_A
:   0.000860463751304386

ASRS\_B
:   -0.00138414940594271

Diagnosis
:   -0.00931123160922751

COHS
:   -0.000297009995489316

FeedbackCondNoFeedback
:   -0.0255769230769231

```
Warning message in confint.boot(boot_go_fb_model3_r, level = 0.95):
"BCa method fails for this problem.  Using 'perc' instead"
```

|  | 2.5 % | 97.5 % |
| --- | --- | --- |
| (Intercept) | -0.0310975924 | 0.0853693900 |
| Gender | -0.0214253268 | 0.0116343481 |
| Cong\_Order | 0.0012315735 | 0.0129684683 |
| Drive | -0.0001942760 | 0.0001098460 |
| ASRS\_A | -0.0007461653 | 0.0025584422 |
| ASRS\_B | -0.0032833687 | 0.0003154969 |
| Diagnosis | -0.0442434547 | 0.0257463321 |
| COHS | -0.0007956026 | 0.0002859777 |
| FeedbackCondNoFeedback | -0.0395855992 | -0.0111908273 |

**$fixed** =

(Intercept)
:   0.00408799175888025

Gender
:   -0.00429549591148491

Cong\_Order
:   0.0071073492484185

Drive
:   -4.13982850130474e-05

ASRS\_A
:   -0.000561770769026743

ASRS\_B
:   -0.000626462892866431

Diagnosis
:   -0.00976746499234544

COHS
:   9.651851996201e-05

FeedbackCondNoFeedback
:   0.026999605376382

ASRS\_A:FeedbackCondNoFeedback
:   0.00284446904066226

ASRS\_B:FeedbackCondNoFeedback
:   -0.00151537302615255

Diagnosis:FeedbackCondNoFeedback
:   0.000912466766235861

COHS:FeedbackCondNoFeedback
:   -0.00078705703090265

```
Warning message in confint.boot(boot_go_fb_model4_r, level = 0.95):
"BCa method fails for this problem.  Using 'perc' instead"
```

|  | 2.5 % | 97.5 % |
| --- | --- | --- |
| (Intercept) | -7.801254e-02 | 0.0813029471 |
| Gender | -1.923189e-02 | 0.0120979263 |
| Cong\_Order | 7.819410e-04 | 0.0135645360 |
| Drive | -1.961920e-04 | 0.0001189740 |
| ASRS\_A | -2.835186e-03 | 0.0015810548 |
| ASRS\_B | -3.106742e-03 | 0.0015622291 |
| Diagnosis | -5.409189e-02 | 0.0345520831 |
| COHS | -6.248764e-04 | 0.0008643224 |
| FeedbackCondNoFeedback | -8.818313e-02 | 0.1425093288 |
| ASRS\_A:FeedbackCondNoFeedback | -5.564803e-05 | 0.0062024094 |
| ASRS\_B:FeedbackCondNoFeedback | -4.709821e-03 | 0.0017736416 |
| Diagnosis:FeedbackCondNoFeedback | -5.913359e-02 | 0.0639714305 |
| COHS:FeedbackCondNoFeedback | -1.980082e-03 | 0.0002309799 |

In [81]:

```
#install.packages("metafor")
#library(metafor)
options(scipen=999)
"print"(confint(boot_go_fb_model4_r, level=0.95), digits=2)
confint(boot_go_fb_model4_r, level=0.95)
#round(summary(go_fb_model4_r)$coefficients[1], 2)
#round(confint(boot_go_fb_model4_r, level=0.95), 2)
```

```
Warning message in confint.boot(boot_go_fb_model4_r, level = 0.95):
"BCa method fails for this problem.  Using 'perc' instead"
```

```
Bootstrap percent confidence intervals

                                     2.5 %  97.5 %
(Intercept)                      -0.078013 0.08130
Gender                           -0.019232 0.01210
Cong_Order                        0.000782 0.01356
Drive                            -0.000196 0.00012
ASRS_A                           -0.002835 0.00158
ASRS_B                           -0.003107 0.00156
Diagnosis                        -0.054092 0.03455
COHS                             -0.000625 0.00086
FeedbackCondNoFeedback           -0.088183 0.14251
ASRS_A:FeedbackCondNoFeedback    -0.000056 0.00620
ASRS_B:FeedbackCondNoFeedback    -0.004710 0.00177
Diagnosis:FeedbackCondNoFeedback -0.059134 0.06397
COHS:FeedbackCondNoFeedback      -0.001980 0.00023
```

```
Warning message in confint.boot(boot_go_fb_model4_r, level = 0.95):
"BCa method fails for this problem.  Using 'perc' instead"
```

|  | 2.5 % | 97.5 % |
| --- | --- | --- |
| (Intercept) | -0.07801253879 | 0.0813029471 |
| Gender | -0.01923189061 | 0.0120979263 |
| Cong\_Order | 0.00078194098 | 0.0135645360 |
| Drive | -0.00019619200 | 0.0001189740 |
| ASRS\_A | -0.00283518569 | 0.0015810548 |
| ASRS\_B | -0.00310674188 | 0.0015622291 |
| Diagnosis | -0.05409188787 | 0.0345520831 |
| COHS | -0.00062487641 | 0.0008643224 |
| FeedbackCondNoFeedback | -0.08818312728 | 0.1425093288 |
| ASRS\_A:FeedbackCondNoFeedback | -0.00005564803 | 0.0062024094 |
| ASRS\_B:FeedbackCondNoFeedback | -0.00470982086 | 0.0017736416 |
| Diagnosis:FeedbackCondNoFeedback | -0.05913358867 | 0.0639714305 |
| COHS:FeedbackCondNoFeedback | -0.00198008211 | 0.0002309799 |

In [ ]:

```

```
