# Supplementary material for: Habit Expression and Disruption as a Function of Attention-Deficit/Hyperactivity Disorder Symptomology
Source: Front Psychol. 2019 Sep 3;10:1997. doi: 10.3389/fpsyg.2019.01997 (PMC6733985; doi:10.3389/fpsyg.2019.01997)
Supplement: Supplementary file 1 [file Data_Sheet_1.ZIP › Signal_detection_analyses.html]

ASRS signal detection analyses


## ASRS signal detection analyses¶

In [1]:

```
#Load packages
library(nlme)
#install.packages("MuMIn")
library(MuMIn)
#install.packages("reghelper")
library(reghelper)
#install.packages("car")
library(car)
#install.packages("stats")
library(stats)
#install.packages("lsr")
library(lsr)
#install.packages("psych")
library(psych)
#install.packages("devtools")
library(devtools)
#install_github("easyGgplot2", "kassambara")
library(easyGgplot2)
#install.packages("lme4")
library(lme4)
```

```
Attaching package: 'reghelper'

The following object is masked from 'package:base':

    beta

Loading required package: carData
Warning message:
"package 'carData' was built under R version 3.5.2"
Attaching package: 'psych'

The following object is masked from 'package:car':

    logit

The following object is masked from 'package:reghelper':

    ICC

Loading required package: ggplot2

Attaching package: 'ggplot2'

The following objects are masked from 'package:psych':

    %+%, alpha

Loading required package: Matrix

Attaching package: 'lme4'

The following object is masked from 'package:nlme':

    lmList
```

#### Familiar congruent day 1 phase d prime dataframe¶

In [2]:

```
myNoGoData_d <- read.csv('nogostatssheet_full.csv')
myGoData_d <- read.csv('gostatssheet_full.csv')
#head(myNoGoData_d)
#head(myGoData_d)
df_FamCongNoGo_d <- subset(myNoGoData_d, StimulusType=="Familiar" & Congruency=="Congruent" & FeedbackCond=="NoFeedback")
df_FamCongGo_d <- subset(myGoData_d, StimulusType=="Familiar" & Congruency=="Congruent" & FeedbackCond=="NoFeedback")
df_FamCong_dprime <- data.frame(Subject = df_FamCongNoGo_d[, c("Subject")], Perc_Hit = df_FamCongGo_d[, c("Accuracy")], Perc_FA = (1-df_FamCongNoGo_d[, c("Accuracy")]), Perc_Miss = (1-df_FamCongGo_d[, c("Accuracy")]), Perc_CR = df_FamCongNoGo_d[, c("Accuracy")])
head(df_FamCong_dprime)
nrow(df_FamCong_dprime)
```

| Subject | Perc\_Hit | Perc\_FA | Perc\_Miss | Perc\_CR |
| --- | --- | --- | --- | --- |
| 1 | 0.81 | 0.30 | 0.19 | 0.70 |
| 2 | 0.97 | 0.15 | 0.03 | 0.85 |
| 3 | 0.96 | 0.45 | 0.04 | 0.55 |
| 4 | 0.92 | 0.30 | 0.08 | 0.70 |
| 5 | 0.97 | 0.15 | 0.03 | 0.85 |
| 6 | 0.93 | 0.35 | 0.07 | 0.65 |

104

#### Familiar incongruent day 1 phase d prime dataframe¶

In [3]:

```
df_FamIncongNoGo_d <- subset(myNoGoData_d, StimulusType=="Familiar" & Congruency=="Incongruent" & FeedbackCond=="NoFeedback")
df_FamIncongGo_d <- subset(myGoData_d, StimulusType=="Familiar" & Congruency=="Incongruent" & FeedbackCond=="NoFeedback")
df_FamIncong_dprime <- data.frame(Subject = df_FamIncongNoGo_d[, c("Subject")], Perc_Hit = df_FamIncongGo_d[, c("Accuracy")], Perc_FA = (1-df_FamIncongNoGo_d[, c("Accuracy")]), Perc_Miss = (1-df_FamIncongGo_d[, c("Accuracy")]), Perc_CR = df_FamIncongNoGo_d[, c("Accuracy")])
head(df_FamIncong_dprime)
nrow(df_FamIncong_dprime)
```

| Subject | Perc\_Hit | Perc\_FA | Perc\_Miss | Perc\_CR |
| --- | --- | --- | --- | --- |
| 1 | 0.77 | 0.75 | 0.23 | 0.25 |
| 2 | 0.96 | 0.25 | 0.04 | 0.75 |
| 3 | 0.93 | 0.55 | 0.07 | 0.45 |
| 4 | 0.95 | 0.25 | 0.05 | 0.75 |
| 5 | 0.95 | 0.25 | 0.05 | 0.75 |
| 6 | 0.92 | 0.50 | 0.08 | 0.50 |

104

#### Novel congruent day 1 phase d prime dataframe¶

In [4]:

```
df_NovCongNoGo_d <- subset(myNoGoData_d, StimulusType=="Novel" & Congruency=="Congruent" & FeedbackCond=="NoFeedback")
df_NovCongGo_d <- subset(myGoData_d, StimulusType=="Novel" & Congruency=="Congruent" & FeedbackCond=="NoFeedback")
df_NovCong_dprime <- data.frame(Subject = df_NovCongNoGo_d[, c("Subject")], Perc_Hit = df_NovCongGo_d[, c("Accuracy")], Perc_FA = (1-df_NovCongNoGo_d[, c("Accuracy")]), Perc_Miss = (1-df_NovCongGo_d[, c("Accuracy")]), Perc_CR = df_NovCongNoGo_d[, c("Accuracy")])
head(df_NovCong_dprime)
nrow(df_NovCong_dprime)
```

| Subject | Perc\_Hit | Perc\_FA | Perc\_Miss | Perc\_CR |
| --- | --- | --- | --- | --- |
| 1 | 0.77 | 0.45 | 0.23 | 0.55 |
| 2 | 0.95 | 0.25 | 0.05 | 0.75 |
| 3 | 0.93 | 0.40 | 0.07 | 0.60 |
| 4 | 0.92 | 0.30 | 0.08 | 0.70 |
| 5 | 0.93 | 0.25 | 0.07 | 0.75 |
| 6 | 0.93 | 0.35 | 0.07 | 0.65 |

104

#### Novel incongruent day 1 phase d prime dataframe¶

In [5]:

```
df_NovIncongNoGo_d <- subset(myNoGoData_d, StimulusType=="Novel" & Congruency=="Incongruent" & FeedbackCond=="NoFeedback")
df_NovIncongGo_d <- subset(myGoData_d, StimulusType=="Novel" & Congruency=="Incongruent" & FeedbackCond=="NoFeedback")
df_NovIncong_dprime <- data.frame(Subject = df_NovIncongNoGo_d[, c("Subject")], Perc_Hit = df_NovIncongGo_d[, c("Accuracy")], Perc_FA = (1-df_NovIncongNoGo_d[, c("Accuracy")]), Perc_Miss = (1-df_NovIncongGo_d[, c("Accuracy")]), Perc_CR = df_NovIncongNoGo_d[, c("Accuracy")])
head(df_NovIncong_dprime)
nrow(df_NovIncong_dprime)
```

| Subject | Perc\_Hit | Perc\_FA | Perc\_Miss | Perc\_CR |
| --- | --- | --- | --- | --- |
| 1 | 0.83 | 0.60 | 0.17 | 0.40 |
| 2 | 1.00 | 0.25 | 0.00 | 0.75 |
| 3 | 0.91 | 0.35 | 0.09 | 0.65 |
| 4 | 0.91 | 0.45 | 0.09 | 0.55 |
| 5 | 0.95 | 0.10 | 0.05 | 0.90 |
| 6 | 0.89 | 0.50 | 0.11 | 0.50 |

104

#### Familiar congruent day 2 phase d prime dataframe¶

In [6]:

```
df_FamCongNoGoFB_d <- subset(myNoGoData_d, StimulusType=="Familiar" & Congruency=="Congruent" & FeedbackCond=="Feedback")
df_FamCongGoFB_d <- subset(myGoData_d, StimulusType=="Familiar" & Congruency=="Congruent" & FeedbackCond=="Feedback")
df_FamCongFB_dprime <- data.frame(Subject = df_FamCongNoGoFB_d[, c("Subject")], Perc_Hit = df_FamCongGoFB_d[, c("Accuracy")], Perc_FA = (1-df_FamCongNoGoFB_d[, c("Accuracy")]), Perc_Miss = (1-df_FamCongGoFB_d[, c("Accuracy")]), Perc_CR = df_FamCongNoGoFB_d[, c("Accuracy")])
head(df_FamCongFB_dprime)
nrow(df_FamCongFB_dprime)
```

| Subject | Perc\_Hit | Perc\_FA | Perc\_Miss | Perc\_CR |
| --- | --- | --- | --- | --- |
| 1 | 0.92 | 0.20 | 0.08 | 0.80 |
| 2 | 0.98 | 0.10 | 0.02 | 0.90 |
| 3 | 0.99 | 0.45 | 0.01 | 0.55 |
| 4 | 0.89 | 0.20 | 0.11 | 0.80 |
| 5 | 1.00 | 0.25 | 0.00 | 0.75 |
| 6 | 0.97 | 0.45 | 0.03 | 0.55 |

104

#### Familiar incongruent day 2 phase d prime dataframe¶

In [7]:

```
df_FamIncongNoGoFB_d <- subset(myNoGoData_d, StimulusType=="Familiar" & Congruency=="Incongruent" & FeedbackCond=="Feedback")
df_FamIncongGoFB_d <- subset(myGoData_d, StimulusType=="Familiar" & Congruency=="Incongruent" & FeedbackCond=="Feedback")
df_FamIncongFB_dprime <- data.frame(Subject = df_FamIncongNoGoFB_d[, c("Subject")], Perc_Hit = df_FamIncongGoFB_d[, c("Accuracy")], Perc_FA = (1-df_FamIncongNoGoFB_d[, c("Accuracy")]), Perc_Miss = (1-df_FamIncongGoFB_d[, c("Accuracy")]), Perc_CR = df_FamIncongNoGoFB_d[, c("Accuracy")])
head(df_FamIncongFB_dprime)
nrow(df_FamIncongFB_dprime)
```

| Subject | Perc\_Hit | Perc\_FA | Perc\_Miss | Perc\_CR |
| --- | --- | --- | --- | --- |
| 1 | 0.88 | 0.65 | 0.12 | 0.35 |
| 2 | 0.99 | 0.15 | 0.01 | 0.85 |
| 3 | 0.94 | 0.30 | 0.06 | 0.70 |
| 4 | 0.98 | 0.50 | 0.02 | 0.50 |
| 5 | 0.96 | 0.45 | 0.04 | 0.55 |
| 6 | 1.00 | 0.55 | 0.00 | 0.45 |

104

### Familiar Congruent Day 1 d prime analysis¶

In [8]:

```
#install.packages("psycho")
library(psycho)
```

In [9]:

```
FamCong_dprime_indices <- psycho::dprime(df_FamCong_dprime$Perc_Hit, df_FamCong_dprime$Perc_FA, df_FamCong_dprime$Perc_Miss, df_FamCong_dprime$Perc_CR)
FamCong_dprime <- cbind(df_FamCong_dprime, FamCong_dprime_indices)
head(FamCong_dprime)
```

| Subject | Perc\_Hit | Perc\_FA | Perc\_Miss | Perc\_CR | dprime | beta | aprime | bppd | c |
| --- | --- | --- | --- | --- | --- | --- | --- | --- | --- |
| 1 | 0.81 | 0.30 | 0.19 | 0.70 | 0.5278942 | 1.113557 | 0.8395503 | -0.2925532 | 0.20375169 |
| 2 | 0.97 | 0.15 | 0.03 | 0.85 | 0.8657486 | 1.199872 | 0.9525167 | -0.7017544 | 0.21047109 |
| 3 | 0.96 | 0.45 | 0.04 | 0.55 | 0.5176180 | 1.024452 | 0.8646307 | -0.9030837 | 0.04667179 |
| 4 | 0.92 | 0.30 | 0.08 | 0.70 | 0.6389834 | 1.099331 | 0.8899068 | -0.6626506 | 0.14820711 |
| 5 | 0.97 | 0.15 | 0.03 | 0.85 | 0.8657486 | 1.199872 | 0.9525167 | -0.7017544 | 0.21047109 |
| 6 | 0.93 | 0.35 | 0.07 | 0.65 | 0.5939314 | 1.071005 | 0.8789909 | -0.7547170 | 0.11549741 |

### Familiar Incongruent Day 1 d prime analysis¶

In [10]:

```
FamIncong_dprime_indices <- psycho::dprime(df_FamIncong_dprime$Perc_Hit, df_FamIncong_dprime$Perc_FA, df_FamIncong_dprime$Perc_Miss, df_FamIncong_dprime$Perc_CR)
FamIncong_dprime <- cbind(df_FamIncong_dprime, FamIncong_dprime_indices)
head(FamIncong_dprime)
```

| Subject | Perc\_Hit | Perc\_FA | Perc\_Miss | Perc\_CR | dprime | beta | aprime | bppd | c |
| --- | --- | --- | --- | --- | --- | --- | --- | --- | --- |
| 1 | 0.77 | 0.75 | 0.23 | 0.25 | 0.02005437 | 0.9997989 | 0.5264935 | -0.8188976 | -0.01002719 |
| 2 | 0.96 | 0.25 | 0.04 | 0.75 | 0.73653771 | 1.1218699 | 0.9215625 | -0.7777778 | 0.15613166 |
| 3 | 0.93 | 0.55 | 0.07 | 0.45 | 0.38336180 | 1.0039228 | 0.8132616 | -0.8839779 | 0.01021258 |
| 4 | 0.95 | 0.25 | 0.05 | 0.75 | 0.72629399 | 1.1242515 | 0.9175439 | -0.7272727 | 0.16125352 |
| 5 | 0.95 | 0.25 | 0.05 | 0.75 | 0.72629399 | 1.1242515 | 0.9175439 | -0.7272727 | 0.16125352 |
| 6 | 0.92 | 0.50 | 0.08 | 0.50 | 0.42463169 | 1.0175758 | 0.8241304 | -0.8400000 | 0.04103126 |

### Novel Congruent Day 1 d prime analysis¶

In [11]:

```
NovCong_dprime_indices <- psycho::dprime(df_NovCong_dprime$Perc_Hit, df_NovCong_dprime$Perc_FA, df_NovCong_dprime$Perc_Miss, df_NovCong_dprime$Perc_CR)
NovCong_dprime <- cbind(df_NovCong_dprime, NovCong_dprime_indices)
head(NovCong_dprime)
```

| Subject | Perc\_Hit | Perc\_FA | Perc\_Miss | Perc\_CR | dprime | beta | aprime | bppd | c |
| --- | --- | --- | --- | --- | --- | --- | --- | --- | --- |
| 1 | 0.77 | 0.45 | 0.23 | 0.55 | 0.3255352 | 1.047554 | 0.7493506 | -0.4651163 | 0.14271321 |
| 2 | 0.95 | 0.25 | 0.05 | 0.75 | 0.7262940 | 1.124251 | 0.9175439 | -0.7272727 | 0.16125352 |
| 3 | 0.93 | 0.40 | 0.07 | 0.60 | 0.5399271 | 1.048941 | 0.8633065 | -0.7971014 | 0.08849524 |
| 4 | 0.92 | 0.30 | 0.08 | 0.70 | 0.6389834 | 1.099331 | 0.8899068 | -0.6626506 | 0.14820711 |
| 5 | 0.93 | 0.25 | 0.07 | 0.75 | 0.7058688 | 1.128662 | 0.9094624 | -0.6315789 | 0.17146610 |
| 6 | 0.93 | 0.35 | 0.07 | 0.65 | 0.5939314 | 1.071005 | 0.8789909 | -0.7547170 | 0.11549741 |

### Novel Incongruent Day 1 d prime analysis¶

In [12]:

```
NovIncong_dprime_indices <- psycho::dprime(df_NovIncong_dprime$Perc_Hit, df_NovIncong_dprime$Perc_FA, df_NovIncong_dprime$Perc_Miss, df_NovIncong_dprime$Perc_CR)
NovIncong_dprime <- cbind(df_NovIncong_dprime, NovIncong_dprime_indices)
head(NovIncong_dprime)
```

| Subject | Perc\_Hit | Perc\_FA | Perc\_Miss | Perc\_CR | dprime | beta | aprime | bppd | c |
| --- | --- | --- | --- | --- | --- | --- | --- | --- | --- |
| 1 | 0.83 | 0.60 | 0.17 | 0.40 | 0.2312675 | 1.008205 | 0.7130271 | -0.7597173 | 0.03533545 |
| 2 | 1.00 | 0.25 | 0.00 | 0.75 | 0.7777476 | 1.111161 | 0.9375000 | -1.0000000 | 0.13552670 |
| 3 | 0.91 | 0.35 | 0.09 | 0.65 | 0.5735817 | 1.074745 | 0.8692308 | -0.6896552 | 0.12567227 |
| 4 | 0.91 | 0.45 | 0.09 | 0.55 | 0.4665994 | 1.034253 | 0.8354645 | -0.7843137 | 0.07218110 |
| 5 | 0.95 | 0.10 | 0.05 | 0.90 | 0.9081960 | 1.257406 | 0.9597953 | -0.3571429 | 0.25220454 |
| 6 | 0.89 | 0.50 | 0.11 | 0.50 | 0.3941825 | 1.022423 | 0.8045506 | -0.7800000 | 0.05625587 |

### Familiar Congruent Day 2 d prime analysis¶

In [13]:

```
FamCongFB_dprime_indices <- psycho::dprime(df_FamCongFB_dprime$Perc_Hit, df_FamCongFB_dprime$Perc_FA, df_FamCongFB_dprime$Perc_Miss, df_FamCongFB_dprime$Perc_CR)
FamCongFB_dprime <- cbind(df_FamCongFB_dprime, FamCongFB_dprime_indices)
head(FamCongFB_dprime)
```

| Subject | Perc\_Hit | Perc\_FA | Perc\_Miss | Perc\_CR | dprime | beta | aprime | bppd | c |
| --- | --- | --- | --- | --- | --- | --- | --- | --- | --- |
| 1 | 0.92 | 0.20 | 0.08 | 0.80 | 0.7541261 | 1.167872 | 0.9206522 | -0.4838710 | 0.20577846 |
| 2 | 0.98 | 0.10 | 0.02 | 0.90 | 0.9389953 | 1.249019 | 0.9689342 | -0.6896552 | 0.23680491 |
| 3 | 0.99 | 0.45 | 0.01 | 0.55 | 0.5484878 | 1.017281 | 0.8818182 | -0.9756098 | 0.03123691 |
| 4 | 0.89 | 0.20 | 0.11 | 0.80 | 0.7236769 | 1.173434 | 0.9094452 | -0.3383459 | 0.22100307 |
| 5 | 1.00 | 0.25 | 0.00 | 0.75 | 0.7777476 | 1.111161 | 0.9375000 | -1.0000000 | 0.13552670 |
| 6 | 0.97 | 0.45 | 0.03 | 0.55 | 0.5278840 | 1.022170 | 0.8703843 | -0.9271523 | 0.04153878 |

### Familiar Incongruent Day 2 d prime analysis¶

In [14]:

```
FamIncongFB_dprime_indices <- psycho::dprime(df_FamIncongFB_dprime$Perc_Hit, df_FamIncongFB_dprime$Perc_FA, df_FamIncongFB_dprime$Perc_Miss, df_FamIncongFB_dprime$Perc_CR)
FamIncongFB_dprime <- cbind(df_FamIncongFB_dprime, FamIncongFB_dprime_indices)
head(FamIncongFB_dprime)
```

| Subject | Perc\_Hit | Perc\_FA | Perc\_Miss | Perc\_CR | dprime | beta | aprime | bppd | c |
| --- | --- | --- | --- | --- | --- | --- | --- | --- | --- |
| 1 | 0.88 | 0.65 | 0.12 | 0.35 | 0.2311497 | 0.9965063 | 0.7296266 | -0.8631922 | -0.01514112 |
| 2 | 0.99 | 0.15 | 0.01 | 0.85 | 0.8863524 | 1.1941331 | 0.9591800 | -0.8917197 | 0.20016922 |
| 3 | 0.94 | 0.30 | 0.06 | 0.70 | 0.6593697 | 1.0952714 | 0.8987842 | -0.7407407 | 0.13801395 |
| 4 | 0.98 | 0.50 | 0.02 | 0.50 | 0.4860399 | 1.0050320 | 0.8624490 | -0.9600000 | 0.01032718 |
| 5 | 0.96 | 0.45 | 0.04 | 0.55 | 0.5176180 | 1.0244523 | 0.8646307 | -0.9030837 | 0.04667179 |
| 6 | 1.00 | 0.55 | 0.00 | 0.45 | 0.4552406 | 0.9883564 | 0.8625000 | -1.0000000 | -0.02572681 |

## D prime analysis master sheet¶

In [15]:

```
df_dprime = data.frame(Subject = df_FamCongNoGo_d[, c("Subject")], FamCongDay1_dprime = FamCong_dprime[, ("dprime")], FamIncongDay1_dprime = FamIncong_dprime[, ("dprime")], NovCongDay1_dprime = NovCong_dprime[, ("dprime")], NovIncongDay1_dprime = NovIncong_dprime[, ("dprime")], FamCongDay2_dprime = FamCongFB_dprime[, ("dprime")], FamIncongDay2_dprime = FamIncongFB_dprime[, ("dprime")])
head(df_dprime)
#write to csv so it can be melted in python
#write.csv(df_dprime, file = "df_dprime_wide.csv")
```

| Subject | FamCongDay1\_dprime | FamIncongDay1\_dprime | NovCongDay1\_dprime | NovIncongDay1\_dprime | FamCongDay2\_dprime | FamIncongDay2\_dprime |
| --- | --- | --- | --- | --- | --- | --- |
| 1 | 0.5278942 | 0.02005437 | 0.3255352 | 0.2312675 | 0.7541261 | 0.2311497 |
| 2 | 0.8657486 | 0.73653771 | 0.7262940 | 0.7777476 | 0.9389953 | 0.8863524 |
| 3 | 0.5176180 | 0.38336180 | 0.5399271 | 0.5735817 | 0.5484878 | 0.6593697 |
| 4 | 0.6389834 | 0.72629399 | 0.6389834 | 0.4665994 | 0.7236769 | 0.4860399 |
| 5 | 0.8657486 | 0.72629399 | 0.7058688 | 0.9081960 | 0.7777476 | 0.5176180 |
| 6 | 0.5939314 | 0.42463169 | 0.5939314 | 0.3941825 | 0.5278840 | 0.4552406 |

In [16]:

```
#read long dprime data from python csv
df_dprime_long <- read.csv("dprime_long.csv")
head(df_dprime_long)
```

| X | Subject | StimulusType | Congruency | FeedbackCond | DV | dprime |
| --- | --- | --- | --- | --- | --- | --- |
| 0 | 1 | Familiar | Congruent | NoFeedback | FamCongDay1\_dprime | 0.5278942 |
| 1 | 2 | Familiar | Congruent | NoFeedback | FamCongDay1\_dprime | 0.8657486 |
| 2 | 3 | Familiar | Congruent | NoFeedback | FamCongDay1\_dprime | 0.5176180 |
| 3 | 4 | Familiar | Congruent | NoFeedback | FamCongDay1\_dprime | 0.6389834 |
| 4 | 5 | Familiar | Congruent | NoFeedback | FamCongDay1\_dprime | 0.8657486 |
| 5 | 6 | Familiar | Congruent | NoFeedback | FamCongDay1\_dprime | 0.5939314 |

### ANOVAs to compare StimulusType, FeedbackCond, and Congruency with dprime values¶

In [18]:

```
library(ez)
print("Day 1 (no feedback) StimulusType*Congruency interaction of dprime values")
dprime_anova  <- ezANOVA(
    data = subset(df_dprime_long, FeedbackCond=="NoFeedback")
    , dv = .(dprime)
    , wid = .(Subject)
    , within = .(StimulusType, Congruency)
    , type = 1
)
print(dprime_anova)

print("Day 2 (feedback) FeedbackCond*Congruency interaction of dprime values")
dprime_anova_fb  <- ezANOVA(
    data = subset(df_dprime_long, StimulusType=="Familiar")
    , dv = .(dprime)
    , wid = .(Subject)
    , within = .(FeedbackCond, Congruency)
    , type = 1
)
print(dprime_anova_fb)
```

```
Warning message:
"package 'ez' was built under R version 3.5.3"
```

```
[1] "Day 1 (no feedback) StimulusType*Congruency interaction of dprime values"
```

```
Warning message:
"Converting "Subject" to factor for ANOVA."
```

```
$ANOVA
                   Effect DFn DFd          F            p p<.05         ges
1            StimulusType   1 103  0.2653472 6.075739e-01       0.001092392
2              Congruency   1 103 13.0718664 4.658828e-04     * 0.030638504
3 StimulusType:Congruency   1 103 25.4235121 1.980098e-06     * 0.074570098

[1] "Day 2 (feedback) FeedbackCond*Congruency interaction of dprime values"
```

```
Warning message:
"Converting "Subject" to factor for ANOVA."
```

```
$ANOVA
                   Effect DFn DFd         F            p p<.05        ges
1            FeedbackCond   1 103  3.954874 4.938841e-02     * 0.01832967
2              Congruency   1 103 14.967710 1.919456e-04     * 0.03688528
3 FeedbackCond:Congruency   1 103 24.202606 3.297861e-06     * 0.05551951
```

#### Post hoc t-tests across phases and conditions¶

In [19]:

```
print("Familiar stimuli, congruent vs. incongruent paired t-test")
t.test(subset(df_dprime_long, StimulusType=="Familiar" & FeedbackCond=="NoFeedback" & Congruency=="Congruent")$dprime, subset(df_dprime_long, StimulusType=="Familiar" & FeedbackCond=="NoFeedback" & Congruency=="Incongruent")$dprime, paired=TRUE)[1:3]
print("Novel stimuli, congruent vs. incongruent paired t-test")
t.test(subset(df_dprime_long, StimulusType=="Novel" & FeedbackCond=="NoFeedback" & Congruency=="Congruent")$dprime, subset(df_dprime_long, StimulusType=="Novel" & FeedbackCond=="NoFeedback" & Congruency=="Incongruent")$dprime, paired=TRUE)[1:3]
print("Congruent phase, Familiar vs. Novel paired t-test")
t.test(subset(df_dprime_long, StimulusType=="Familiar" & FeedbackCond=="NoFeedback" & Congruency=="Congruent")$dprime, subset(df_dprime_long, StimulusType=="Novel" & FeedbackCond=="NoFeedback" & Congruency=="Congruent")$dprime, paired=TRUE)[1:3]
print("Incongruent phase, Familiar vs. Novel paired t-test")
t.test(subset(df_dprime_long, StimulusType=="Familiar" & FeedbackCond=="NoFeedback" & Congruency=="Incongruent")$dprime, subset(df_dprime_long, StimulusType=="Novel" & FeedbackCond=="NoFeedback" & Congruency=="Incongruent")$dprime, paired=TRUE)[1:3]
print("Familiar stimuli, Feedback condition, congruent vs. incongruent paired t-test")
t.test(subset(df_dprime_long, StimulusType=="Familiar" & FeedbackCond=="Feedback" & Congruency=="Congruent")$dprime, subset(df_dprime_long, StimulusType=="Familiar" & FeedbackCond=="Feedback" & Congruency=="Incongruent")$dprime, paired=TRUE)[1:3]
```

```
[1] "Familiar stimuli, congruent vs. incongruent paired t-test"
```

$statistic
:   **t:** 5.97961770876442

$parameter
:   **df:** 103

$p.value
:   3.25757278008154e-08

```
[1] "Novel stimuli, congruent vs. incongruent paired t-test"
```

$statistic
:   **t:** -1.4690435379711

$parameter
:   **df:** 103

$p.value
:   0.144868654826563

```
[1] "Congruent phase, Familiar vs. Novel paired t-test"
```

$statistic
:   **t:** 3.84062009290549

$parameter
:   **df:** 103

$p.value
:   0.000212263501823778

```
[1] "Incongruent phase, Familiar vs. Novel paired t-test"
```

$statistic
:   **t:** -2.84483538297293

$parameter
:   **df:** 103

$p.value
:   0.00536121427995792

```
[1] "Familiar stimuli, Feedback condition, congruent vs. incongruent paired t-test"
```

$statistic
:   **t:** -0.688905225046506

$parameter
:   **df:** 103

$p.value
:   0.492431810317542

In [20]:

```
summary(subset(df_dprime_long, FeedbackCond=="NoFeedback" & Congruency=="Congruent" & StimulusType=="Familiar")$dprime)
summary(subset(df_dprime_long, FeedbackCond=="NoFeedback" & Congruency=="Incongruent" & StimulusType=="Familiar")$dprime)
summary(subset(df_dprime_long, FeedbackCond=="NoFeedback" & Congruency=="Congruent" & StimulusType=="Novel")$dprime)
summary(subset(df_dprime_long, FeedbackCond=="NoFeedback" & Congruency=="Incongruent" & StimulusType=="Novel")$dprime)
summary(subset(df_dprime_long, FeedbackCond=="Feedback" & Congruency=="Congruent" & StimulusType=="Familiar")$dprime)
summary(subset(df_dprime_long, FeedbackCond=="Feedback" & Congruency=="Incongruent" & StimulusType=="Familiar")$dprime)
```

```
   Min. 1st Qu.  Median    Mean 3rd Qu.    Max. 
0.09036 0.52788 0.69569 0.68291 0.85548 1.06402
```

```
   Min. 1st Qu.  Median    Mean 3rd Qu.    Max. 
0.02005 0.37376 0.55683 0.56194 0.74467 1.00489
```

```
   Min. 1st Qu.  Median    Mean 3rd Qu.    Max. 
 0.0000  0.4246  0.6165  0.5999  0.7486  1.0743
```

```
   Min. 1st Qu.  Median    Mean 3rd Qu.    Max. 
 0.0407  0.4860  0.6463  0.6277  0.7794  1.0640
```

```
   Min. 1st Qu.  Median    Mean 3rd Qu.    Max. 
 0.0000  0.5130  0.7059  0.6537  0.8284  1.0950
```

```
   Min. 1st Qu.  Median    Mean 3rd Qu.    Max. 
0.03014 0.47824 0.71215 0.66660 0.85294 1.09497
```

### Mixed models using dprime data with individual difference measures¶

In [21]:

```
##take individual difference variables from Exp8_NoGo_Full.csv, join them with the dprime long dataframe
#head(read.csv("Exp8_NoGo_Full.csv"))
idvar <- read.csv("Exp8_NoGo_Full.csv")[, 5:14]
idvar_long <- rbind(idvar,idvar[])
df_dprime_long_mm <- cbind(df_dprime_long, idvar_long)
nrow(df_dprime_long_mm)
ncol(df_dprime_long_mm)
df_dprime_long_mm$X <- NULL
head(df_dprime_long_mm)
#write.csv(df_dprime_long_mm, file="df_dprime_long_mm.csv")
```

624

17

| Subject | StimulusType | Congruency | FeedbackCond | DV | dprime | Cong\_Order | Stim\_Order | ASRS\_A | ASRS\_B | ASRS\_Total | COHS | Age | Gender | Drive | Diagnosis |
| --- | --- | --- | --- | --- | --- | --- | --- | --- | --- | --- | --- | --- | --- | --- | --- |
| 1 | Familiar | Congruent | NoFeedback | FamCongDay1\_dprime | 0.5278942 | 0 | 0 | 18 | 10 | 28 | 131 | 23 | 0 | 84 | 0 |
| 2 | Familiar | Congruent | NoFeedback | FamCongDay1\_dprime | 0.8657486 | 0 | 0 | 12 | 10 | 22 | 111 | 19 | 0 | 16 | 0 |
| 3 | Familiar | Congruent | NoFeedback | FamCongDay1\_dprime | 0.5176180 | 1 | 0 | 10 | 10 | 20 | 83 | 19 | 1 | 1 | 0 |
| 4 | Familiar | Congruent | NoFeedback | FamCongDay1\_dprime | 0.6389834 | 1 | 0 | 18 | 13 | 31 | 105 | 18 | 0 | 18 | 0 |
| 5 | Familiar | Congruent | NoFeedback | FamCongDay1\_dprime | 0.8657486 | 2 | 1 | 22 | 17 | 39 | 77 | 20 | 0 | 27 | 1 |
| 6 | Familiar | Congruent | NoFeedback | FamCongDay1\_dprime | 0.5939314 | 2 | 1 | 8 | 18 | 26 | 105 | 18 | 1 | 6 | 0 |

In [22]:

```
#Day 1 model with dprime
dprime_nofb_model1_r <- lme(dprime ~ Gender + Cong_Order + Drive, random=~1|Subject, method="ML", data=subset(df_dprime_long_mm, FeedbackCond=="NoFeedback"))
dprime_nofb_model2_r <- lme(dprime ~ Gender + Cong_Order + Drive + ASRS_A + ASRS_B + Diagnosis + COHS, random=~1|Subject, method="ML", data=subset(df_dprime_long_mm, FeedbackCond=="NoFeedback"))
dprime_nofb_model3_r <- lme(dprime ~ Gender + Cong_Order + Drive + ASRS_A + ASRS_B + Diagnosis + COHS + StimulusType + Congruency + StimulusType*Congruency, random=~1|Subject, method="ML", data=subset(df_dprime_long_mm, FeedbackCond=="NoFeedback"))
dprime_nofb_model4_r <- lme(dprime ~ Gender + Cong_Order + Drive + ASRS_A + ASRS_B + Diagnosis + COHS + StimulusType + Congruency + StimulusType*Congruency + ASRS_A*StimulusType*Congruency + ASRS_B*StimulusType*Congruency + Diagnosis*StimulusType*Congruency + COHS*StimulusType*Congruency, random=~1|Subject, method="ML", data=subset(df_dprime_long_mm, FeedbackCond=="NoFeedback"))
```

In [23]:

```
#Check for outliers, beyond -3.3<x<3.3. No output means no outliers.
which(abs(residuals(dprime_nofb_model1_r, type="normalized")) > 3.3)
which(abs(residuals(dprime_nofb_model2_r, type="normalized")) > 3.3)
which(abs(residuals(dprime_nofb_model3_r, type="normalized")) > 3.3)
which(abs(residuals(dprime_nofb_model4_r, type="normalized")) > 3.3)
```

In [24]:

```
#Diagnostics. plot() checks for homoscedasticity violation, qqplot() checks for normality, vif() checks for multicollinearity
qqnorm(resid(dprime_nofb_model1_r))
qqnorm(resid(dprime_nofb_model2_r))
qqnorm(resid(dprime_nofb_model3_r))
qqnorm(resid(dprime_nofb_model4_r))
plot(dprime_nofb_model1_r)
plot(dprime_nofb_model2_r)
plot(dprime_nofb_model3_r)
plot(dprime_nofb_model4_r)
```

In [25]:

```
vif(dprime_nofb_model1_r)
vif(dprime_nofb_model2_r)
vif(dprime_nofb_model3_r)
vif(dprime_nofb_model4_r)
```

Gender
:   1.01476451801994

Cong\_Order
:   1.01415654061579

Drive
:   1.00383723096897

Gender
:   1.07951357845143

Cong\_Order
:   1.04126308412311

Drive
:   1.30402400280693

ASRS\_A
:   1.61665380591124

ASRS\_B
:   1.70620586645643

Diagnosis
:   1.30512798861589

COHS
:   1.06294714788362

Gender
:   1.07951357845143

Cong\_Order
:   1.04126308412311

Drive
:   1.30402400280693

ASRS\_A
:   1.61665380591124

ASRS\_B
:   1.70620586645643

Diagnosis
:   1.30512798861589

COHS
:   1.06294714788362

StimulusType
:   2

Congruency
:   2

StimulusType:Congruency
:   3

Gender
:   1.0795135784514

Cong\_Order
:   1.04126308412311

Drive
:   1.30402400280691

ASRS\_A
:   2.36496892078297

ASRS\_B
:   2.48259694714233

Diagnosis
:   1.81041462359411

COHS
:   1.57134764070584

StimulusType
:   129.585057031892

Congruency
:   129.585057031904

StimulusType:Congruency
:   194.377585547843

ASRS\_A:StimulusType
:   30.4188490053886

ASRS\_A:Congruency
:   30.418849005391

ASRS\_B:StimulusType
:   24.4950400874214

ASRS\_B:Congruency
:   24.4950400874229

Diagnosis:StimulusType
:   2.55976879638125

Diagnosis:Congruency
:   2.55976879638125

COHS:StimulusType
:   112.967406061662

COHS:Congruency
:   112.967406061672

ASRS\_A:StimulusType:Congruency
:   45.1293967648362

ASRS\_B:StimulusType:Congruency
:   36.2249660773423

Diagnosis:StimulusType:Congruency
:   3.50279543791979

COHS:StimulusType:Congruency
:   169.112175430617

In [26]:

```
#Use beta from reghelper, otherwise beta coefs won't be standardized
beta(dprime_nofb_model1_r)
beta(dprime_nofb_model2_r)
beta(dprime_nofb_model3_r)
beta(dprime_nofb_model4_r)
```

```
Linear mixed-effects model fit by maximum likelihood
 Data: data 
       AIC      BIC    logLik
  1053.502 1077.686 -520.7511

Random effects:
 Formula: ~1 | Subject
        (Intercept)  Residual
StdDev:    0.716217 0.6858888

Fixed effects: dprime.z ~ Gender.z + Cong_Order.z + Drive.z 
                  Value  Std.Error  DF   t-value p-value
(Intercept)  0.00000000 0.07824394 312 0.0000000  1.0000
Gender.z     0.07082378 0.07891434 100 0.8974767  0.3716
Cong_Order.z 0.00163141 0.07889070 100 0.0206794  0.9835
Drive.z      0.09229977 0.07848831 100 1.1759684  0.2424
 Correlation: 
             (Intr) Gndr.z Cng_O.
Gender.z      0.000              
Cong_Order.z  0.000 -0.110       
Drive.z       0.000 -0.045 -0.037

Standardized Within-Group Residuals:
        Min          Q1         Med          Q3         Max 
-2.40116512 -0.60916418  0.06488038  0.61371615  2.24711491 

Number of Observations: 416
Number of Groups: 104
```

```
Linear mixed-effects model fit by maximum likelihood
 Data: data 
       AIC      BIC    logLik
  1060.535 1100.841 -520.2673

Random effects:
 Formula: ~1 | Subject
        (Intercept)  Residual
StdDev:   0.7121285 0.6858888

Fixed effects: dprime.z ~ Gender.z + Cong_Order.z + Drive.z + ASRS_A.z + ASRS_B.z +      Diagnosis.z + COHS.z 
                   Value  Std.Error  DF    t-value p-value
(Intercept)   0.00000000 0.07826162 312  0.0000000  1.0000
Gender.z      0.06406711 0.08141145  96  0.7869546  0.4332
Cong_Order.z  0.01012538 0.07995611  96  0.1266367  0.8995
Drive.z       0.08634509 0.08947758  96  0.9649913  0.3370
ASRS_A.z      0.02202359 0.09962766  96  0.2210590  0.8255
ASRS_B.z     -0.08612770 0.10234983  96 -0.8415031  0.4022
Diagnosis.z  -0.02888103 0.08951545  96 -0.3226374  0.7477
COHS.z        0.02867349 0.08078436  96  0.3549386  0.7234
 Correlation: 
             (Intr) Gndr.z Cng_O. Driv.z ASRS_A ASRS_B Dgnss.
Gender.z      0.000                                          
Cong_Order.z  0.000 -0.118                                   
Drive.z       0.000 -0.112 -0.073                            
ASRS_A.z      0.000 -0.178  0.114  0.011                     
ASRS_B.z      0.000  0.096 -0.152  0.165 -0.596              
Diagnosis.z   0.000  0.187  0.025 -0.422 -0.154  0.107       
COHS.z        0.000 -0.055  0.041 -0.062  0.159 -0.216  0.066

Standardized Within-Group Residuals:
        Min          Q1         Med          Q3         Max 
-2.37763800 -0.61796068  0.06366376  0.62082250  2.25492182 

Number of Observations: 416
Number of Groups: 104
```

```
Linear mixed-effects model fit by maximum likelihood
 Data: data 
       AIC      BIC    logLik
  1033.054 1085.453 -503.5269

Random effects:
 Formula: ~1 | Subject
        (Intercept)  Residual
StdDev:   0.7204819 0.6500573

Fixed effects: dprime.z ~ Gender.z + Cong_Order.z + Drive.z + ASRS_A.z + ASRS_B.z +      Diagnosis.z + COHS.z + StimulusTypeNovel.z + CongruencyIncongruent.z +      StimulusTypeNovel.z * CongruencyIncongruent.z 
                                                  Value  Std.Error  DF
(Intercept)                                  0.00000000 0.07855094 309
Gender.z                                     0.06406711 0.08171242  96
Cong_Order.z                                 0.01012538 0.08025170  96
Drive.z                                      0.08634509 0.08980837  96
ASRS_A.z                                     0.02202359 0.09999597  96
ASRS_B.z                                    -0.08612770 0.10272821  96
Diagnosis.z                                 -0.02888103 0.08984637  96
COHS.z                                       0.02867349 0.08108300  96
StimulusTypeNovel.z                         -0.01863938 0.03234050 309
CongruencyIncongruent.z                     -0.10020647 0.03234050 309
StimulusTypeNovel.z:CongruencyIncongruent.z  0.16019098 0.03237944 309
                                              t-value p-value
(Intercept)                                  0.000000  1.0000
Gender.z                                     0.784056  0.4349
Cong_Order.z                                 0.126170  0.8999
Drive.z                                      0.961437  0.3387
ASRS_A.z                                     0.220245  0.8261
ASRS_B.z                                    -0.838404  0.4039
Diagnosis.z                                 -0.321449  0.7486
COHS.z                                       0.353631  0.7244
StimulusTypeNovel.z                         -0.576348  0.5648
CongruencyIncongruent.z                     -3.098482  0.0021
StimulusTypeNovel.z:CongruencyIncongruent.z  4.947306  0.0000
 Correlation: 
                                            (Intr) Gndr.z Cng_O. Driv.z ASRS_A
Gender.z                                     0.000                            
Cong_Order.z                                 0.000 -0.118                     
Drive.z                                      0.000 -0.112 -0.073              
ASRS_A.z                                     0.000 -0.178  0.114  0.011       
ASRS_B.z                                     0.000  0.096 -0.152  0.165 -0.596
Diagnosis.z                                  0.000  0.187  0.025 -0.422 -0.154
COHS.z                                       0.000 -0.055  0.041 -0.062  0.159
StimulusTypeNovel.z                          0.000  0.000  0.000  0.000  0.000
CongruencyIncongruent.z                      0.000  0.000  0.000  0.000  0.000
StimulusTypeNovel.z:CongruencyIncongruent.z  0.000  0.000  0.000  0.000  0.000
                                            ASRS_B Dgnss. COHS.z StmTN. CngrI.
Gender.z                                                                      
Cong_Order.z                                                                  
Drive.z                                                                       
ASRS_A.z                                                                      
ASRS_B.z                                                                      
Diagnosis.z                                  0.107                            
COHS.z                                      -0.216  0.066                     
StimulusTypeNovel.z                          0.000  0.000  0.000              
CongruencyIncongruent.z                      0.000  0.000  0.000  0.000       
StimulusTypeNovel.z:CongruencyIncongruent.z  0.000  0.000  0.000  0.000  0.000

Standardized Within-Group Residuals:
        Min          Q1         Med          Q3         Max 
-2.55479336 -0.58456294  0.07278563  0.63535710  2.34778924 

Number of Observations: 416
Number of Groups: 104
```

```
Linear mixed-effects model fit by maximum likelihood
 Data: data 
       AIC     BIC    logLik
  1041.383 1142.15 -495.6914

Random effects:
 Formula: ~1 | Subject
        (Intercept) Residual
StdDev:   0.7240645 0.633935

Fixed effects: dprime.z ~ Gender.z + Cong_Order.z + Drive.z + ASRS_A.z + ASRS_B.z +      Diagnosis.z + COHS.z + StimulusTypeNovel.z + CongruencyIncongruent.z +      StimulusTypeNovel.z * CongruencyIncongruent.z + ASRS_A.z *      StimulusTypeNovel.z * CongruencyIncongruent.z + ASRS_B.z *      StimulusTypeNovel.z * CongruencyIncongruent.z + Diagnosis.z *      StimulusTypeNovel.z * CongruencyIncongruent.z + COHS.z *      StimulusTypeNovel.z * CongruencyIncongruent.z 
                                                              Value  Std.Error
(Intercept)                                              0.00000000 0.07974117
Gender.z                                                 0.06406711 0.08295055
Cong_Order.z                                             0.01012538 0.08146770
Drive.z                                                  0.08634509 0.09116918
ASRS_A.z                                                 0.02202359 0.10151115
ASRS_B.z                                                -0.08612770 0.10428478
Diagnosis.z                                             -0.02888103 0.09120776
COHS.z                                                   0.02867349 0.08231160
StimulusTypeNovel.z                                     -0.01863938 0.03201630
CongruencyIncongruent.z                                 -0.10020647 0.03201630
StimulusTypeNovel.z:CongruencyIncongruent.z              0.16019098 0.03205485
ASRS_A.z:StimulusTypeNovel.z                            -0.08492669 0.03992174
ASRS_A.z:CongruencyIncongruent.z                        -0.01424861 0.03992174
ASRS_B.z:StimulusTypeNovel.z                             0.08288701 0.04066376
ASRS_B.z:CongruencyIncongruent.z                         0.00924979 0.04066376
Diagnosis.z:StimulusTypeNovel.z                          0.00072237 0.03280470
Diagnosis.z:CongruencyIncongruent.z                      0.03795031 0.03280470
COHS.z:StimulusTypeNovel.z                              -0.01453741 0.03290563
COHS.z:CongruencyIncongruent.z                          -0.02605947 0.03290563
ASRS_A.z:StimulusTypeNovel.z:CongruencyIncongruent.z    -0.03275842 0.03996981
ASRS_B.z:StimulusTypeNovel.z:CongruencyIncongruent.z    -0.01032024 0.04071272
Diagnosis.z:StimulusTypeNovel.z:CongruencyIncongruent.z  0.05515102 0.03284420
COHS.z:StimulusTypeNovel.z:CongruencyIncongruent.z       0.06098196 0.03294525
                                                         DF   t-value p-value
(Intercept)                                             297  0.000000  1.0000
Gender.z                                                 96  0.772353  0.4418
Cong_Order.z                                             96  0.124287  0.9013
Drive.z                                                  96  0.947086  0.3460
ASRS_A.z                                                 96  0.216957  0.8287
ASRS_B.z                                                 96 -0.825889  0.4109
Diagnosis.z                                              96 -0.316651  0.7522
COHS.z                                                   96  0.348353  0.7283
StimulusTypeNovel.z                                     297 -0.582184  0.5609
CongruencyIncongruent.z                                 297 -3.129858  0.0019
StimulusTypeNovel.z:CongruencyIncongruent.z             297  4.997403  0.0000
ASRS_A.z:StimulusTypeNovel.z                            297 -2.127329  0.0342
ASRS_A.z:CongruencyIncongruent.z                        297 -0.356914  0.7214
ASRS_B.z:StimulusTypeNovel.z                            297  2.038351  0.0424
ASRS_B.z:CongruencyIncongruent.z                        297  0.227470  0.8202
Diagnosis.z:StimulusTypeNovel.z                         297  0.022020  0.9824
Diagnosis.z:CongruencyIncongruent.z                     297  1.156856  0.2483
COHS.z:StimulusTypeNovel.z                              297 -0.441791  0.6590
COHS.z:CongruencyIncongruent.z                          297 -0.791946  0.4290
ASRS_A.z:StimulusTypeNovel.z:CongruencyIncongruent.z    297 -0.819579  0.4131
ASRS_B.z:StimulusTypeNovel.z:CongruencyIncongruent.z    297 -0.253489  0.8001
Diagnosis.z:StimulusTypeNovel.z:CongruencyIncongruent.z 297  1.679171  0.0942
COHS.z:StimulusTypeNovel.z:CongruencyIncongruent.z      297  1.851009  0.0652
 Correlation: 
                                                        (Intr) Gndr.z Cng_O.
Gender.z                                                 0.000              
Cong_Order.z                                             0.000 -0.118       
Drive.z                                                  0.000 -0.112 -0.073
ASRS_A.z                                                 0.000 -0.178  0.114
ASRS_B.z                                                 0.000  0.096 -0.152
Diagnosis.z                                              0.000  0.187  0.025
COHS.z                                                   0.000 -0.055  0.041
StimulusTypeNovel.z                                      0.000  0.000  0.000
CongruencyIncongruent.z                                  0.000  0.000  0.000
StimulusTypeNovel.z:CongruencyIncongruent.z              0.000  0.000  0.000
ASRS_A.z:StimulusTypeNovel.z                             0.000  0.000  0.000
ASRS_A.z:CongruencyIncongruent.z                         0.000  0.000  0.000
ASRS_B.z:StimulusTypeNovel.z                             0.000  0.000  0.000
ASRS_B.z:CongruencyIncongruent.z                         0.000  0.000  0.000
Diagnosis.z:StimulusTypeNovel.z                          0.000  0.000  0.000
Diagnosis.z:CongruencyIncongruent.z                      0.000  0.000  0.000
COHS.z:StimulusTypeNovel.z                               0.000  0.000  0.000
COHS.z:CongruencyIncongruent.z                           0.000  0.000  0.000
ASRS_A.z:StimulusTypeNovel.z:CongruencyIncongruent.z     0.000  0.000  0.000
ASRS_B.z:StimulusTypeNovel.z:CongruencyIncongruent.z     0.000  0.000  0.000
Diagnosis.z:StimulusTypeNovel.z:CongruencyIncongruent.z  0.000  0.000  0.000
COHS.z:StimulusTypeNovel.z:CongruencyIncongruent.z       0.000  0.000  0.000
                                                        Driv.z ASRS_A.z
Gender.z                                                               
Cong_Order.z                                                           
Drive.z                                                                
ASRS_A.z                                                 0.011         
ASRS_B.z                                                 0.165 -0.596  
Diagnosis.z                                             -0.422 -0.154  
COHS.z                                                  -0.062  0.159  
StimulusTypeNovel.z                                      0.000  0.000  
CongruencyIncongruent.z                                  0.000  0.000  
StimulusTypeNovel.z:CongruencyIncongruent.z              0.000  0.000  
ASRS_A.z:StimulusTypeNovel.z                             0.000  0.000  
ASRS_A.z:CongruencyIncongruent.z                         0.000  0.000  
ASRS_B.z:StimulusTypeNovel.z                             0.000  0.000  
ASRS_B.z:CongruencyIncongruent.z                         0.000  0.000  
Diagnosis.z:StimulusTypeNovel.z                          0.000  0.000  
Diagnosis.z:CongruencyIncongruent.z                      0.000  0.000  
COHS.z:StimulusTypeNovel.z                               0.000  0.000  
COHS.z:CongruencyIncongruent.z                           0.000  0.000  
ASRS_A.z:StimulusTypeNovel.z:CongruencyIncongruent.z     0.000  0.000  
ASRS_B.z:StimulusTypeNovel.z:CongruencyIncongruent.z     0.000  0.000  
Diagnosis.z:StimulusTypeNovel.z:CongruencyIncongruent.z  0.000  0.000  
COHS.z:StimulusTypeNovel.z:CongruencyIncongruent.z       0.000  0.000  
                                                        ASRS_B.z Dgnss. COHS.z
Gender.z                                                                      
Cong_Order.z                                                                  
Drive.z                                                                       
ASRS_A.z                                                                      
ASRS_B.z                                                                      
Diagnosis.z                                              0.107                
COHS.z                                                  -0.216    0.066       
StimulusTypeNovel.z                                      0.000    0.000  0.000
CongruencyIncongruent.z                                  0.000    0.000  0.000
StimulusTypeNovel.z:CongruencyIncongruent.z              0.000    0.000  0.000
ASRS_A.z:StimulusTypeNovel.z                             0.000    0.000  0.000
ASRS_A.z:CongruencyIncongruent.z                         0.000    0.000  0.000
ASRS_B.z:StimulusTypeNovel.z                             0.000    0.000  0.000
ASRS_B.z:CongruencyIncongruent.z                         0.000    0.000  0.000
Diagnosis.z:StimulusTypeNovel.z                          0.000    0.000  0.000
Diagnosis.z:CongruencyIncongruent.z                      0.000    0.000  0.000
COHS.z:StimulusTypeNovel.z                               0.000    0.000  0.000
COHS.z:CongruencyIncongruent.z                           0.000    0.000  0.000
ASRS_A.z:StimulusTypeNovel.z:CongruencyIncongruent.z     0.000    0.000  0.000
ASRS_B.z:StimulusTypeNovel.z:CongruencyIncongruent.z     0.000    0.000  0.000
Diagnosis.z:StimulusTypeNovel.z:CongruencyIncongruent.z  0.000    0.000  0.000
COHS.z:StimulusTypeNovel.z:CongruencyIncongruent.z       0.000    0.000  0.000
                                                        StmTN. CngrI. STN.:C
Gender.z                                                                    
Cong_Order.z                                                                
Drive.z                                                                     
ASRS_A.z                                                                    
ASRS_B.z                                                                    
Diagnosis.z                                                                 
COHS.z                                                                      
StimulusTypeNovel.z                                                         
CongruencyIncongruent.z                                  0.000              
StimulusTypeNovel.z:CongruencyIncongruent.z              0.000  0.000       
ASRS_A.z:StimulusTypeNovel.z                             0.000  0.000  0.000
ASRS_A.z:CongruencyIncongruent.z                         0.000  0.000  0.000
ASRS_B.z:StimulusTypeNovel.z                             0.000  0.000  0.000
ASRS_B.z:CongruencyIncongruent.z                         0.000  0.000  0.000
Diagnosis.z:StimulusTypeNovel.z                          0.000  0.000  0.000
Diagnosis.z:CongruencyIncongruent.z                      0.000  0.000  0.000
COHS.z:StimulusTypeNovel.z                               0.000  0.000  0.000
COHS.z:CongruencyIncongruent.z                           0.000  0.000  0.000
ASRS_A.z:StimulusTypeNovel.z:CongruencyIncongruent.z     0.000  0.000  0.000
ASRS_B.z:StimulusTypeNovel.z:CongruencyIncongruent.z     0.000  0.000  0.000
Diagnosis.z:StimulusTypeNovel.z:CongruencyIncongruent.z  0.000  0.000  0.000
COHS.z:StimulusTypeNovel.z:CongruencyIncongruent.z       0.000  0.000  0.000
                                                        ASRS_A.z:STN. ASRS_A.:C
Gender.z                                                                       
Cong_Order.z                                                                   
Drive.z                                                                        
ASRS_A.z                                                                       
ASRS_B.z                                                                       
Diagnosis.z                                                                    
COHS.z                                                                         
StimulusTypeNovel.z                                                            
CongruencyIncongruent.z                                                        
StimulusTypeNovel.z:CongruencyIncongruent.z                                    
ASRS_A.z:StimulusTypeNovel.z                                                   
ASRS_A.z:CongruencyIncongruent.z                         0.000                 
ASRS_B.z:StimulusTypeNovel.z                            -0.594         0.000   
ASRS_B.z:CongruencyIncongruent.z                         0.000        -0.594   
Diagnosis.z:StimulusTypeNovel.z                         -0.143         0.000   
Diagnosis.z:CongruencyIncongruent.z                      0.000        -0.143   
COHS.z:StimulusTypeNovel.z                               0.149         0.000   
COHS.z:CongruencyIncongruent.z                           0.000         0.149   
ASRS_A.z:StimulusTypeNovel.z:CongruencyIncongruent.z     0.000         0.000   
ASRS_B.z:StimulusTypeNovel.z:CongruencyIncongruent.z     0.000         0.000   
Diagnosis.z:StimulusTypeNovel.z:CongruencyIncongruent.z  0.000         0.000   
COHS.z:StimulusTypeNovel.z:CongruencyIncongruent.z       0.000         0.000   
                                                        ASRS_B.z:STN. ASRS_B.:C
Gender.z                                                                       
Cong_Order.z                                                                   
Drive.z                                                                        
ASRS_A.z                                                                       
ASRS_B.z                                                                       
Diagnosis.z                                                                    
COHS.z                                                                         
StimulusTypeNovel.z                                                            
CongruencyIncongruent.z                                                        
StimulusTypeNovel.z:CongruencyIncongruent.z                                    
ASRS_A.z:StimulusTypeNovel.z                                                   
ASRS_A.z:CongruencyIncongruent.z                                               
ASRS_B.z:StimulusTypeNovel.z                                                   
ASRS_B.z:CongruencyIncongruent.z                         0.000                 
Diagnosis.z:StimulusTypeNovel.z                          0.186         0.000   
Diagnosis.z:CongruencyIncongruent.z                      0.000         0.186   
COHS.z:StimulusTypeNovel.z                              -0.202         0.000   
COHS.z:CongruencyIncongruent.z                           0.000        -0.202   
ASRS_A.z:StimulusTypeNovel.z:CongruencyIncongruent.z     0.000         0.000   
ASRS_B.z:StimulusTypeNovel.z:CongruencyIncongruent.z     0.000         0.000   
Diagnosis.z:StimulusTypeNovel.z:CongruencyIncongruent.z  0.000         0.000   
COHS.z:StimulusTypeNovel.z:CongruencyIncongruent.z       0.000         0.000   
                                                        Dg.:STN. D.:CI.
Gender.z                                                               
Cong_Order.z                                                           
Drive.z                                                                
ASRS_A.z                                                               
ASRS_B.z                                                               
Diagnosis.z                                                            
COHS.z                                                                 
StimulusTypeNovel.z                                                    
CongruencyIncongruent.z                                                
StimulusTypeNovel.z:CongruencyIncongruent.z                            
ASRS_A.z:StimulusTypeNovel.z                                           
ASRS_A.z:CongruencyIncongruent.z                                       
ASRS_B.z:StimulusTypeNovel.z                                           
ASRS_B.z:CongruencyIncongruent.z                                       
Diagnosis.z:StimulusTypeNovel.z                                        
Diagnosis.z:CongruencyIncongruent.z                      0.000         
COHS.z:StimulusTypeNovel.z                               0.054    0.000
COHS.z:CongruencyIncongruent.z                           0.000    0.054
ASRS_A.z:StimulusTypeNovel.z:CongruencyIncongruent.z     0.000    0.000
ASRS_B.z:StimulusTypeNovel.z:CongruencyIncongruent.z     0.000    0.000
Diagnosis.z:StimulusTypeNovel.z:CongruencyIncongruent.z  0.000    0.000
COHS.z:StimulusTypeNovel.z:CongruencyIncongruent.z       0.000    0.000
                                                        COHS.z:STN. COHS.:C
Gender.z                                                                   
Cong_Order.z                                                               
Drive.z                                                                    
ASRS_A.z                                                                   
ASRS_B.z                                                                   
Diagnosis.z                                                                
COHS.z                                                                     
StimulusTypeNovel.z                                                        
CongruencyIncongruent.z                                                    
StimulusTypeNovel.z:CongruencyIncongruent.z                                
ASRS_A.z:StimulusTypeNovel.z                                               
ASRS_A.z:CongruencyIncongruent.z                                           
ASRS_B.z:StimulusTypeNovel.z                                               
ASRS_B.z:CongruencyIncongruent.z                                           
Diagnosis.z:StimulusTypeNovel.z                                            
Diagnosis.z:CongruencyIncongruent.z                                        
COHS.z:StimulusTypeNovel.z                                                 
COHS.z:CongruencyIncongruent.z                           0.000             
ASRS_A.z:StimulusTypeNovel.z:CongruencyIncongruent.z     0.000       0.000 
ASRS_B.z:StimulusTypeNovel.z:CongruencyIncongruent.z     0.000       0.000 
Diagnosis.z:StimulusTypeNovel.z:CongruencyIncongruent.z  0.000       0.000 
COHS.z:StimulusTypeNovel.z:CongruencyIncongruent.z       0.000       0.000 
                                                        ASRS_A.:STN.:
Gender.z                                                             
Cong_Order.z                                                         
Drive.z                                                              
ASRS_A.z                                                             
ASRS_B.z                                                             
Diagnosis.z                                                          
COHS.z                                                               
StimulusTypeNovel.z                                                  
CongruencyIncongruent.z                                              
StimulusTypeNovel.z:CongruencyIncongruent.z                          
ASRS_A.z:StimulusTypeNovel.z                                         
ASRS_A.z:CongruencyIncongruent.z                                     
ASRS_B.z:StimulusTypeNovel.z                                         
ASRS_B.z:CongruencyIncongruent.z                                     
Diagnosis.z:StimulusTypeNovel.z                                      
Diagnosis.z:CongruencyIncongruent.z                                  
COHS.z:StimulusTypeNovel.z                                           
COHS.z:CongruencyIncongruent.z                                       
ASRS_A.z:StimulusTypeNovel.z:CongruencyIncongruent.z                 
ASRS_B.z:StimulusTypeNovel.z:CongruencyIncongruent.z    -0.594       
Diagnosis.z:StimulusTypeNovel.z:CongruencyIncongruent.z -0.143       
COHS.z:StimulusTypeNovel.z:CongruencyIncongruent.z       0.149       
                                                        ASRS_B.:STN.: D.:STN.:
Gender.z                                                                      
Cong_Order.z                                                                  
Drive.z                                                                       
ASRS_A.z                                                                      
ASRS_B.z                                                                      
Diagnosis.z                                                                   
COHS.z                                                                        
StimulusTypeNovel.z                                                           
CongruencyIncongruent.z                                                       
StimulusTypeNovel.z:CongruencyIncongruent.z                                   
ASRS_A.z:StimulusTypeNovel.z                                                  
ASRS_A.z:CongruencyIncongruent.z                                              
ASRS_B.z:StimulusTypeNovel.z                                                  
ASRS_B.z:CongruencyIncongruent.z                                              
Diagnosis.z:StimulusTypeNovel.z                                               
Diagnosis.z:CongruencyIncongruent.z                                           
COHS.z:StimulusTypeNovel.z                                                    
COHS.z:CongruencyIncongruent.z                                                
ASRS_A.z:StimulusTypeNovel.z:CongruencyIncongruent.z                          
ASRS_B.z:StimulusTypeNovel.z:CongruencyIncongruent.z                          
Diagnosis.z:StimulusTypeNovel.z:CongruencyIncongruent.z  0.186                
COHS.z:StimulusTypeNovel.z:CongruencyIncongruent.z      -0.202         0.054  

Standardized Within-Group Residuals:
        Min          Q1         Med          Q3         Max 
-2.27145310 -0.62629495  0.08271762  0.59802681  2.56527704 

Number of Observations: 416
Number of Groups: 104
```

In [27]:

```
#Extract the R^2 value of each model
r.squaredGLMM(dprime_nofb_model1_r)
r.squaredGLMM(dprime_nofb_model2_r)
r.squaredGLMM(dprime_nofb_model3_r)
r.squaredGLMM(dprime_nofb_model4_r)
```

```
Warning message:
"'r.squaredGLMM' now calculates a revised statistic. See the help page."
```

| R2m | R2c |
| --- | --- |
| 0.01425388 | 0.5284392 |

| R2m | R2c |
| --- | --- |
| 0.02012145 | 0.5284458 |

| R2m | R2c |
| --- | --- |
| 0.05618978 | 0.5764645 |

| R2m | R2c |
| --- | --- |
| 0.07178549 | 0.5972275 |

In [28]:

```
#Subtract from each other to derive delta R^2. First will be 2-1, next 3-2. 
r.squaredGLMM(dprime_nofb_model2_r) - r.squaredGLMM(dprime_nofb_model1_r)
r.squaredGLMM(dprime_nofb_model3_r) - r.squaredGLMM(dprime_nofb_model2_r)
r.squaredGLMM(dprime_nofb_model4_r) - r.squaredGLMM(dprime_nofb_model3_r)
```

| R2m | R2c |
| --- | --- |
| 0.005867572 | 6.651851e-06 |

| R2m | R2c |
| --- | --- |
| 0.03606833 | 0.04801871 |

| R2m | R2c |
| --- | --- |
| 0.01559571 | 0.02076297 |

In [29]:

```
#Compare the models to each other to extract log likelihood ratio Chi^2 values and the associated p-values. 
#Df is however many new variables are added to next model.
anova(dprime_nofb_model1_r, dprime_nofb_model2_r, dprime_nofb_model3_r, dprime_nofb_model4_r)
```

|  | call | Model | df | AIC | BIC | logLik | Test | L.Ratio | p-value |
| --- | --- | --- | --- | --- | --- | --- | --- | --- | --- |
| dprime\_nofb\_model1\_r | lme.formula(fixed = dprime ~ Gender + Cong\_Order + Drive, data = subset(df\_dprime\_long\_mm, FeedbackCond == "NoFeedback"), random = ~1 | Subject, method = "ML") | 1 | 6 | -159.5025 | -135.31841 | 85.75126 |  | NA | NA |
| dprime\_nofb\_model2\_r | lme.formula(fixed = dprime ~ Gender + Cong\_Order + Drive + ASRS\_A + ASRS\_B + Diagnosis + COHS, data = subset(df\_dprime\_long\_mm, FeedbackCond == "NoFeedback"), random = ~1 | Subject, method = "ML") | 2 | 10 | -152.4702 | -112.16332 | 86.23508 | 1 vs 2 | 0.967648 | 9.146613e-01 |
| dprime\_nofb\_model3\_r | lme.formula(fixed = dprime ~ Gender + Cong\_Order + Drive + ASRS\_A + ASRS\_B + Diagnosis + COHS + StimulusType + Congruency + StimulusType \* Congruency, data = subset(df\_dprime\_long\_mm, FeedbackCond == "NoFeedback"), random = ~1 | Subject, method = "ML") | 3 | 13 | -179.9509 | -127.55199 | 102.97545 | 2 vs 3 | 33.480729 | 2.549906e-07 |
| dprime\_nofb\_model4\_r | lme.formula(fixed = dprime ~ Gender + Cong\_Order + Drive + ASRS\_A + ASRS\_B + Diagnosis + COHS + StimulusType + Congruency + StimulusType \* Congruency + ASRS\_A \* StimulusType \* Congruency + ASRS\_B \* StimulusType \* Congruency + Diagnosis \* StimulusType \* Congruency + COHS \* StimulusType \* Congruency, data = subset(df\_dprime\_long\_mm, FeedbackCond == "NoFeedback"), random = ~1 | Subject, method = "ML") | 4 | 25 | -171.6220 | -70.85487 | 110.81100 | 3 vs 4 | 15.671104 | 2.067687e-01 |

### Day 2 (feedback) data mixed model¶

In [30]:

```
#Day 1 model with dprime
dprime_fb_model1_r <- lme(dprime ~ Gender + Cong_Order + Drive, random=~1|Subject, method="ML", data=subset(df_dprime_long_mm, StimulusType=="Familiar"))
dprime_fb_model2_r <- lme(dprime ~ Gender + Cong_Order + Drive + ASRS_A + ASRS_B + Diagnosis + COHS, random=~1|Subject, method="ML", data=subset(df_dprime_long_mm, StimulusType=="Familiar"))
dprime_fb_model3_r <- lme(dprime ~ Gender + Cong_Order + Drive + ASRS_A + ASRS_B + Diagnosis + COHS + FeedbackCond + Congruency + FeedbackCond*Congruency, random=~1|Subject, method="ML", data=subset(df_dprime_long_mm, StimulusType=="Familiar"))
dprime_fb_model4_r <- lme(dprime ~ Gender + Cong_Order + Drive + ASRS_A + ASRS_B + Diagnosis + COHS + FeedbackCond + Congruency + FeedbackCond*Congruency + ASRS_A*FeedbackCond*Congruency + ASRS_B*FeedbackCond*Congruency + Diagnosis*FeedbackCond*Congruency + COHS*FeedbackCond*Congruency, random=~1|Subject, method="ML", data=subset(df_dprime_long_mm, StimulusType=="Familiar"))
```

In [31]:

```
#Check for outliers, beyond -3.3<x<3.3. No output means no outliers.
which(abs(residuals(dprime_fb_model1_r, type="normalized")) > 3.3)
which(abs(residuals(dprime_fb_model2_r, type="normalized")) > 3.3)
which(abs(residuals(dprime_fb_model3_r, type="normalized")) > 3.3)
which(abs(residuals(dprime_fb_model4_r, type="normalized")) > 3.3)
```

In [32]:

```
#Diagnostics. plot() checks for homoscedasticity violation, qqplot() checks for normality, vif() checks for multicollinearity
qqnorm(resid(dprime_fb_model1_r))
qqnorm(resid(dprime_fb_model2_r))
qqnorm(resid(dprime_fb_model3_r))
qqnorm(resid(dprime_fb_model4_r))
plot(dprime_fb_model1_r)
plot(dprime_fb_model2_r)
plot(dprime_fb_model3_r)
plot(dprime_fb_model4_r)
```

In [33]:

```
vif(dprime_fb_model1_r)
vif(dprime_fb_model2_r)
vif(dprime_fb_model3_r)
vif(dprime_fb_model4_r)
```

Gender
:   1.01476451801994

Cong\_Order
:   1.01415654061579

Drive
:   1.00383723096897

Gender
:   1.07951357845143

Cong\_Order
:   1.04126308412311

Drive
:   1.30402400280693

ASRS\_A
:   1.61665380591124

ASRS\_B
:   1.70620586645643

Diagnosis
:   1.30512798861589

COHS
:   1.06294714788362

Gender
:   1.07951357845143

Cong\_Order
:   1.04126308412311

Drive
:   1.30402400280693

ASRS\_A
:   1.61665380591124

ASRS\_B
:   1.70620586645643

Diagnosis
:   1.30512798861589

COHS
:   1.06294714788362

FeedbackCond
:   2

Congruency
:   2

FeedbackCond:Congruency
:   3

Gender
:   1.07951357845133

Cong\_Order
:   1.04126308412315

Drive
:   1.30402400280698

ASRS\_A
:   2.39009826965186

ASRS\_B
:   2.50866912173493

Diagnosis
:   1.82738277494749

COHS
:   1.58842035926431

FeedbackCond
:   129.585057031894

Congruency
:   129.585057031893

FeedbackCond:Congruency
:   194.377585547838

ASRS\_A:FeedbackCond
:   30.4356019046351

ASRS\_A:Congruency
:   30.4356019046346

ASRS\_B:FeedbackCond
:   24.5124215371502

ASRS\_B:Congruency
:   24.5124215371498

Diagnosis:FeedbackCond
:   2.57108089728354

Diagnosis:Congruency
:   2.57108089728353

COHS:FeedbackCond
:   112.978787874037

COHS:Congruency
:   112.978787874035

ASRS\_A:FeedbackCond:Congruency
:   45.137773214458

ASRS\_B:FeedbackCond:Congruency
:   36.2336568022057

Diagnosis:FeedbackCond:Congruency
:   3.50845148837092

COHS:FeedbackCond:Congruency
:   169.117866336799

In [34]:

```
#Use beta from reghelper, otherwise beta coefs won't be standardized
beta(dprime_fb_model1_r)
beta(dprime_fb_model2_r)
beta(dprime_fb_model3_r)
beta(dprime_fb_model4_r)
```

```
Linear mixed-effects model fit by maximum likelihood
 Data: data 
       AIC      BIC    logLik
  1049.229 1073.413 -518.6146

Random effects:
 Formula: ~1 | Subject
        (Intercept)  Residual
StdDev:   0.7142287 0.6819645

Fixed effects: dprime.z ~ Gender.z + Cong_Order.z + Drive.z 
                   Value  Std.Error  DF    t-value p-value
(Intercept)   0.00000000 0.07798379 312  0.0000000  1.0000
Gender.z      0.07076684 0.07865197 100  0.8997466  0.3704
Cong_Order.z -0.06899935 0.07862840 100 -0.8775372  0.3823
Drive.z       0.11684500 0.07822735 100  1.4936592  0.1384
 Correlation: 
             (Intr) Gndr.z Cng_O.
Gender.z      0.000              
Cong_Order.z  0.000 -0.110       
Drive.z       0.000 -0.045 -0.037

Standardized Within-Group Residuals:
        Min          Q1         Med          Q3         Max 
-2.46791949 -0.57861226  0.08083738  0.60171354  2.39627185 

Number of Observations: 416
Number of Groups: 104
```

```
Linear mixed-effects model fit by maximum likelihood
 Data: data 
      AIC      BIC    logLik
  1054.13 1094.437 -517.0649

Random effects:
 Formula: ~1 | Subject
        (Intercept)  Residual
StdDev:   0.7012353 0.6819645

Fixed effects: dprime.z ~ Gender.z + Cong_Order.z + Drive.z + ASRS_A.z + ASRS_B.z +      Diagnosis.z + COHS.z 
                   Value  Std.Error  DF    t-value p-value
(Intercept)   0.00000000 0.07720611 312  0.0000000  1.0000
Gender.z      0.05393971 0.08031346  96  0.6716149  0.5034
Cong_Order.z -0.05569006 0.07887775  96 -0.7060300  0.4819
Drive.z       0.11977716 0.08827080  96  1.3569284  0.1780
ASRS_A.z      0.04441637 0.09828399  96  0.4519187  0.6523
ASRS_B.z     -0.14323894 0.10096945  96 -1.4186365  0.1592
Diagnosis.z  -0.07559333 0.08830816  96 -0.8560176  0.3941
COHS.z        0.05526106 0.07969482  96  0.6934085  0.4897
 Correlation: 
             (Intr) Gndr.z Cng_O. Driv.z ASRS_A ASRS_B Dgnss.
Gender.z      0.000                                          
Cong_Order.z  0.000 -0.118                                   
Drive.z       0.000 -0.112 -0.073                            
ASRS_A.z      0.000 -0.178  0.114  0.011                     
ASRS_B.z      0.000  0.096 -0.152  0.165 -0.596              
Diagnosis.z   0.000  0.187  0.025 -0.422 -0.154  0.107       
COHS.z        0.000 -0.055  0.041 -0.062  0.159 -0.216  0.066

Standardized Within-Group Residuals:
       Min         Q1        Med         Q3        Max 
-2.5514504 -0.5802645  0.0734819  0.6077699  2.4273641 

Number of Observations: 416
Number of Groups: 104
```

```
Linear mixed-effects model fit by maximum likelihood
 Data: data 
       AIC      BIC    logLik
  1025.957 1078.356 -499.9783

Random effects:
 Formula: ~1 | Subject
        (Intercept)  Residual
StdDev:   0.7097839 0.6456212

Fixed effects: dprime.z ~ Gender.z + Cong_Order.z + Drive.z + ASRS_A.z + ASRS_B.z +      Diagnosis.z + COHS.z + FeedbackCondNoFeedback.z + CongruencyIncongruent.z +      FeedbackCondNoFeedback.z * CongruencyIncongruent.z 
                                                       Value  Std.Error  DF
(Intercept)                                       0.00000000 0.07749153 309
Gender.z                                          0.05393971 0.08061037  96
Cong_Order.z                                     -0.05569006 0.07916935  96
Drive.z                                           0.11977716 0.08859712  96
ASRS_A.z                                          0.04441637 0.09864733  96
ASRS_B.z                                         -0.14323894 0.10134272  96
Diagnosis.z                                      -0.07559333 0.08863462  96
COHS.z                                            0.05526106 0.07998944  96
FeedbackCondNoFeedback.z                         -0.07649367 0.03211980 309
CongruencyIncongruent.z                          -0.10955155 0.03211980 309
FeedbackCondNoFeedback.z:CongruencyIncongruent.z -0.13588764 0.03215848 309
                                                   t-value p-value
(Intercept)                                       0.000000  1.0000
Gender.z                                          0.669141  0.5050
Cong_Order.z                                     -0.703430  0.4835
Drive.z                                           1.351931  0.1796
ASRS_A.z                                          0.450254  0.6535
ASRS_B.z                                         -1.413411  0.1608
Diagnosis.z                                      -0.852865  0.3959
COHS.z                                            0.690854  0.4913
FeedbackCondNoFeedback.z                         -2.381511  0.0178
CongruencyIncongruent.z                          -3.410717  0.0007
FeedbackCondNoFeedback.z:CongruencyIncongruent.z -4.225562  0.0000
 Correlation: 
                                                 (Intr) Gndr.z Cng_O. Driv.z
Gender.z                                          0.000                     
Cong_Order.z                                      0.000 -0.118              
Drive.z                                           0.000 -0.112 -0.073       
ASRS_A.z                                          0.000 -0.178  0.114  0.011
ASRS_B.z                                          0.000  0.096 -0.152  0.165
Diagnosis.z                                       0.000  0.187  0.025 -0.422
COHS.z                                            0.000 -0.055  0.041 -0.062
FeedbackCondNoFeedback.z                          0.000  0.000  0.000  0.000
CongruencyIncongruent.z                           0.000  0.000  0.000  0.000
FeedbackCondNoFeedback.z:CongruencyIncongruent.z  0.000  0.000  0.000  0.000
                                                 ASRS_A ASRS_B Dgnss. COHS.z
Gender.z                                                                    
Cong_Order.z                                                                
Drive.z                                                                     
ASRS_A.z                                                                    
ASRS_B.z                                         -0.596                     
Diagnosis.z                                      -0.154  0.107              
COHS.z                                            0.159 -0.216  0.066       
FeedbackCondNoFeedback.z                          0.000  0.000  0.000  0.000
CongruencyIncongruent.z                           0.000  0.000  0.000  0.000
FeedbackCondNoFeedback.z:CongruencyIncongruent.z  0.000  0.000  0.000  0.000
                                                 FdCNF. CngrI.
Gender.z                                                      
Cong_Order.z                                                  
Drive.z                                                       
ASRS_A.z                                                      
ASRS_B.z                                                      
Diagnosis.z                                                   
COHS.z                                                        
FeedbackCondNoFeedback.z                                      
CongruencyIncongruent.z                           0.000       
FeedbackCondNoFeedback.z:CongruencyIncongruent.z  0.000  0.000

Standardized Within-Group Residuals:
        Min          Q1         Med          Q3         Max 
-2.49611536 -0.58180706  0.06750361  0.62914623  2.41376678 

Number of Observations: 416
Number of Groups: 104
```

```
Linear mixed-effects model fit by maximum likelihood
 Data: data 
       AIC      BIC    logLik
  1040.391 1141.158 -495.1953

Random effects:
 Formula: ~1 | Subject
        (Intercept)  Residual
StdDev:    0.711997 0.6357991

Fixed effects: dprime.z ~ Gender.z + Cong_Order.z + Drive.z + ASRS_A.z + ASRS_B.z +      Diagnosis.z + COHS.z + FeedbackCondNoFeedback.z + CongruencyIncongruent.z +      FeedbackCondNoFeedback.z * CongruencyIncongruent.z + ASRS_A.z *      FeedbackCondNoFeedback.z * CongruencyIncongruent.z + ASRS_B.z *      FeedbackCondNoFeedback.z * CongruencyIncongruent.z + Diagnosis.z *      FeedbackCondNoFeedback.z * CongruencyIncongruent.z + COHS.z *      FeedbackCondNoFeedback.z * CongruencyIncongruent.z 
                                                                   Value
(Intercept)                                                   0.00000000
Gender.z                                                      0.05393971
Cong_Order.z                                                 -0.05569006
Drive.z                                                       0.11977716
ASRS_A.z                                                      0.04441637
ASRS_B.z                                                     -0.14323894
Diagnosis.z                                                  -0.07559333
COHS.z                                                        0.05526106
FeedbackCondNoFeedback.z                                     -0.07649367
CongruencyIncongruent.z                                      -0.10955155
FeedbackCondNoFeedback.z:CongruencyIncongruent.z             -0.13588764
ASRS_A.z:FeedbackCondNoFeedback.z                             0.04996994
ASRS_A.z:CongruencyIncongruent.z                             -0.01633354
ASRS_B.z:FeedbackCondNoFeedback.z                             0.00125566
ASRS_B.z:CongruencyIncongruent.z                              0.03036929
Diagnosis.z:FeedbackCondNoFeedback.z                          0.03029550
Diagnosis.z:CongruencyIncongruent.z                          -0.02351840
COHS.z:FeedbackCondNoFeedback.z                              -0.01844219
COHS.z:CongruencyIncongruent.z                               -0.05071679
ASRS_A.z:FeedbackCondNoFeedback.z:CongruencyIncongruent.z     0.03378486
ASRS_B.z:FeedbackCondNoFeedback.z:CongruencyIncongruent.z    -0.01194815
Diagnosis.z:FeedbackCondNoFeedback.z:CongruencyIncongruent.z  0.00737595
COHS.z:FeedbackCondNoFeedback.z:CongruencyIncongruent.z      -0.03129923
                                                              Std.Error  DF
(Intercept)                                                  0.07866571 297
Gender.z                                                     0.08183180  96
Cong_Order.z                                                 0.08036895  96
Drive.z                                                      0.08993958  96
ASRS_A.z                                                     0.10014208  96
ASRS_B.z                                                     0.10287830  96
Diagnosis.z                                                  0.08997765  96
COHS.z                                                       0.08120147  96
FeedbackCondNoFeedback.z                                     0.03211044 297
CongruencyIncongruent.z                                      0.03211044 297
FeedbackCondNoFeedback.z:CongruencyIncongruent.z             0.03214910 297
ASRS_A.z:FeedbackCondNoFeedback.z                            0.04003913 297
ASRS_A.z:CongruencyIncongruent.z                             0.04003913 297
ASRS_B.z:FeedbackCondNoFeedback.z                            0.04078333 297
ASRS_B.z:CongruencyIncongruent.z                             0.04078333 297
Diagnosis.z:FeedbackCondNoFeedback.z                         0.03290116 297
Diagnosis.z:CongruencyIncongruent.z                          0.03290116 297
COHS.z:FeedbackCondNoFeedback.z                              0.03300239 297
COHS.z:CongruencyIncongruent.z                               0.03300239 297
ASRS_A.z:FeedbackCondNoFeedback.z:CongruencyIncongruent.z    0.04008734 297
ASRS_B.z:FeedbackCondNoFeedback.z:CongruencyIncongruent.z    0.04083243 297
Diagnosis.z:FeedbackCondNoFeedback.z:CongruencyIncongruent.z 0.03294078 297
COHS.z:FeedbackCondNoFeedback.z:CongruencyIncongruent.z      0.03304212 297
                                                               t-value p-value
(Intercept)                                                   0.000000  1.0000
Gender.z                                                      0.659153  0.5114
Cong_Order.z                                                 -0.692930  0.4900
Drive.z                                                       1.331751  0.1861
ASRS_A.z                                                      0.443534  0.6584
ASRS_B.z                                                     -1.392314  0.1670
Diagnosis.z                                                  -0.840135  0.4029
COHS.z                                                        0.680543  0.4978
FeedbackCondNoFeedback.z                                     -2.382206  0.0178
CongruencyIncongruent.z                                      -3.411711  0.0007
FeedbackCondNoFeedback.z:CongruencyIncongruent.z             -4.226794  0.0000
ASRS_A.z:FeedbackCondNoFeedback.z                             1.248028  0.2130
ASRS_A.z:CongruencyIncongruent.z                             -0.407939  0.6836
ASRS_B.z:FeedbackCondNoFeedback.z                             0.030789  0.9755
ASRS_B.z:CongruencyIncongruent.z                              0.744650  0.4571
Diagnosis.z:FeedbackCondNoFeedback.z                          0.920803  0.3579
Diagnosis.z:CongruencyIncongruent.z                          -0.714820  0.4753
COHS.z:FeedbackCondNoFeedback.z                              -0.558814  0.5767
COHS.z:CongruencyIncongruent.z                               -1.536761  0.1254
ASRS_A.z:FeedbackCondNoFeedback.z:CongruencyIncongruent.z     0.842781  0.4000
ASRS_B.z:FeedbackCondNoFeedback.z:CongruencyIncongruent.z    -0.292614  0.7700
Diagnosis.z:FeedbackCondNoFeedback.z:CongruencyIncongruent.z  0.223916  0.8230
COHS.z:FeedbackCondNoFeedback.z:CongruencyIncongruent.z      -0.947252  0.3443
 Correlation: 
                                                             (Intr) Gndr.z
Gender.z                                                      0.000       
Cong_Order.z                                                  0.000 -0.118
Drive.z                                                       0.000 -0.112
ASRS_A.z                                                      0.000 -0.178
ASRS_B.z                                                      0.000  0.096
Diagnosis.z                                                   0.000  0.187
COHS.z                                                        0.000 -0.055
FeedbackCondNoFeedback.z                                      0.000  0.000
CongruencyIncongruent.z                                       0.000  0.000
FeedbackCondNoFeedback.z:CongruencyIncongruent.z              0.000  0.000
ASRS_A.z:FeedbackCondNoFeedback.z                             0.000  0.000
ASRS_A.z:CongruencyIncongruent.z                              0.000  0.000
ASRS_B.z:FeedbackCondNoFeedback.z                             0.000  0.000
ASRS_B.z:CongruencyIncongruent.z                              0.000  0.000
Diagnosis.z:FeedbackCondNoFeedback.z                          0.000  0.000
Diagnosis.z:CongruencyIncongruent.z                           0.000  0.000
COHS.z:FeedbackCondNoFeedback.z                               0.000  0.000
COHS.z:CongruencyIncongruent.z                                0.000  0.000
ASRS_A.z:FeedbackCondNoFeedback.z:CongruencyIncongruent.z     0.000  0.000
ASRS_B.z:FeedbackCondNoFeedback.z:CongruencyIncongruent.z     0.000  0.000
Diagnosis.z:FeedbackCondNoFeedback.z:CongruencyIncongruent.z  0.000  0.000
COHS.z:FeedbackCondNoFeedback.z:CongruencyIncongruent.z       0.000  0.000
                                                             Cng_O. Driv.z
Gender.z                                                                  
Cong_Order.z                                                              
Drive.z                                                      -0.073       
ASRS_A.z                                                      0.114  0.011
ASRS_B.z                                                     -0.152  0.165
Diagnosis.z                                                   0.025 -0.422
COHS.z                                                        0.041 -0.062
FeedbackCondNoFeedback.z                                      0.000  0.000
CongruencyIncongruent.z                                       0.000  0.000
FeedbackCondNoFeedback.z:CongruencyIncongruent.z              0.000  0.000
ASRS_A.z:FeedbackCondNoFeedback.z                             0.000  0.000
ASRS_A.z:CongruencyIncongruent.z                              0.000  0.000
ASRS_B.z:FeedbackCondNoFeedback.z                             0.000  0.000
ASRS_B.z:CongruencyIncongruent.z                              0.000  0.000
Diagnosis.z:FeedbackCondNoFeedback.z                          0.000  0.000
Diagnosis.z:CongruencyIncongruent.z                           0.000  0.000
COHS.z:FeedbackCondNoFeedback.z                               0.000  0.000
COHS.z:CongruencyIncongruent.z                                0.000  0.000
ASRS_A.z:FeedbackCondNoFeedback.z:CongruencyIncongruent.z     0.000  0.000
ASRS_B.z:FeedbackCondNoFeedback.z:CongruencyIncongruent.z     0.000  0.000
Diagnosis.z:FeedbackCondNoFeedback.z:CongruencyIncongruent.z  0.000  0.000
COHS.z:FeedbackCondNoFeedback.z:CongruencyIncongruent.z       0.000  0.000
                                                             ASRS_A.z ASRS_B.z
Gender.z                                                                      
Cong_Order.z                                                                  
Drive.z                                                                       
ASRS_A.z                                                                      
ASRS_B.z                                                     -0.596           
Diagnosis.z                                                  -0.154    0.107  
COHS.z                                                        0.159   -0.216  
FeedbackCondNoFeedback.z                                      0.000    0.000  
CongruencyIncongruent.z                                       0.000    0.000  
FeedbackCondNoFeedback.z:CongruencyIncongruent.z              0.000    0.000  
ASRS_A.z:FeedbackCondNoFeedback.z                             0.000    0.000  
ASRS_A.z:CongruencyIncongruent.z                              0.000    0.000  
ASRS_B.z:FeedbackCondNoFeedback.z                             0.000    0.000  
ASRS_B.z:CongruencyIncongruent.z                              0.000    0.000  
Diagnosis.z:FeedbackCondNoFeedback.z                          0.000    0.000  
Diagnosis.z:CongruencyIncongruent.z                           0.000    0.000  
COHS.z:FeedbackCondNoFeedback.z                               0.000    0.000  
COHS.z:CongruencyIncongruent.z                                0.000    0.000  
ASRS_A.z:FeedbackCondNoFeedback.z:CongruencyIncongruent.z     0.000    0.000  
ASRS_B.z:FeedbackCondNoFeedback.z:CongruencyIncongruent.z     0.000    0.000  
Diagnosis.z:FeedbackCondNoFeedback.z:CongruencyIncongruent.z  0.000    0.000  
COHS.z:FeedbackCondNoFeedback.z:CongruencyIncongruent.z       0.000    0.000  
                                                             Dgnss. COHS.z
Gender.z                                                                  
Cong_Order.z                                                              
Drive.z                                                                   
ASRS_A.z                                                                  
ASRS_B.z                                                                  
Diagnosis.z                                                               
COHS.z                                                        0.066       
FeedbackCondNoFeedback.z                                      0.000  0.000
CongruencyIncongruent.z                                       0.000  0.000
FeedbackCondNoFeedback.z:CongruencyIncongruent.z              0.000  0.000
ASRS_A.z:FeedbackCondNoFeedback.z                             0.000  0.000
ASRS_A.z:CongruencyIncongruent.z                              0.000  0.000
ASRS_B.z:FeedbackCondNoFeedback.z                             0.000  0.000
ASRS_B.z:CongruencyIncongruent.z                              0.000  0.000
Diagnosis.z:FeedbackCondNoFeedback.z                          0.000  0.000
Diagnosis.z:CongruencyIncongruent.z                           0.000  0.000
COHS.z:FeedbackCondNoFeedback.z                               0.000  0.000
COHS.z:CongruencyIncongruent.z                                0.000  0.000
ASRS_A.z:FeedbackCondNoFeedback.z:CongruencyIncongruent.z     0.000  0.000
ASRS_B.z:FeedbackCondNoFeedback.z:CongruencyIncongruent.z     0.000  0.000
Diagnosis.z:FeedbackCondNoFeedback.z:CongruencyIncongruent.z  0.000  0.000
COHS.z:FeedbackCondNoFeedback.z:CongruencyIncongruent.z       0.000  0.000
                                                             FdCNF. CngrI.
Gender.z                                                                  
Cong_Order.z                                                              
Drive.z                                                                   
ASRS_A.z                                                                  
ASRS_B.z                                                                  
Diagnosis.z                                                               
COHS.z                                                                    
FeedbackCondNoFeedback.z                                                  
CongruencyIncongruent.z                                       0.000       
FeedbackCondNoFeedback.z:CongruencyIncongruent.z              0.000  0.000
ASRS_A.z:FeedbackCondNoFeedback.z                             0.000  0.000
ASRS_A.z:CongruencyIncongruent.z                              0.000  0.000
ASRS_B.z:FeedbackCondNoFeedback.z                             0.000  0.000
ASRS_B.z:CongruencyIncongruent.z                              0.000  0.000
Diagnosis.z:FeedbackCondNoFeedback.z                          0.000  0.000
Diagnosis.z:CongruencyIncongruent.z                           0.000  0.000
COHS.z:FeedbackCondNoFeedback.z                               0.000  0.000
COHS.z:CongruencyIncongruent.z                                0.000  0.000
ASRS_A.z:FeedbackCondNoFeedback.z:CongruencyIncongruent.z     0.000  0.000
ASRS_B.z:FeedbackCondNoFeedback.z:CongruencyIncongruent.z     0.000  0.000
Diagnosis.z:FeedbackCondNoFeedback.z:CongruencyIncongruent.z  0.000  0.000
COHS.z:FeedbackCondNoFeedback.z:CongruencyIncongruent.z       0.000  0.000
                                                             FCNF.:
Gender.z                                                           
Cong_Order.z                                                       
Drive.z                                                            
ASRS_A.z                                                           
ASRS_B.z                                                           
Diagnosis.z                                                        
COHS.z                                                             
FeedbackCondNoFeedback.z                                           
CongruencyIncongruent.z                                            
FeedbackCondNoFeedback.z:CongruencyIncongruent.z                   
ASRS_A.z:FeedbackCondNoFeedback.z                             0.000
ASRS_A.z:CongruencyIncongruent.z                              0.000
ASRS_B.z:FeedbackCondNoFeedback.z                             0.000
ASRS_B.z:CongruencyIncongruent.z                              0.000
Diagnosis.z:FeedbackCondNoFeedback.z                          0.000
Diagnosis.z:CongruencyIncongruent.z                           0.000
COHS.z:FeedbackCondNoFeedback.z                               0.000
COHS.z:CongruencyIncongruent.z                                0.000
ASRS_A.z:FeedbackCondNoFeedback.z:CongruencyIncongruent.z     0.000
ASRS_B.z:FeedbackCondNoFeedback.z:CongruencyIncongruent.z     0.000
Diagnosis.z:FeedbackCondNoFeedback.z:CongruencyIncongruent.z  0.000
COHS.z:FeedbackCondNoFeedback.z:CongruencyIncongruent.z       0.000
                                                             ASRS_A.z:FCNF.
Gender.z                                                                   
Cong_Order.z                                                               
Drive.z                                                                    
ASRS_A.z                                                                   
ASRS_B.z                                                                   
Diagnosis.z                                                                
COHS.z                                                                     
FeedbackCondNoFeedback.z                                                   
CongruencyIncongruent.z                                                    
FeedbackCondNoFeedback.z:CongruencyIncongruent.z                           
ASRS_A.z:FeedbackCondNoFeedback.z                                          
ASRS_A.z:CongruencyIncongruent.z                              0.000        
ASRS_B.z:FeedbackCondNoFeedback.z                            -0.594        
ASRS_B.z:CongruencyIncongruent.z                              0.000        
Diagnosis.z:FeedbackCondNoFeedback.z                         -0.143        
Diagnosis.z:CongruencyIncongruent.z                           0.000        
COHS.z:FeedbackCondNoFeedback.z                               0.149        
COHS.z:CongruencyIncongruent.z                                0.000        
ASRS_A.z:FeedbackCondNoFeedback.z:CongruencyIncongruent.z     0.000        
ASRS_B.z:FeedbackCondNoFeedback.z:CongruencyIncongruent.z     0.000        
Diagnosis.z:FeedbackCondNoFeedback.z:CongruencyIncongruent.z  0.000        
COHS.z:FeedbackCondNoFeedback.z:CongruencyIncongruent.z       0.000        
                                                             ASRS_A.:C
Gender.z                                                              
Cong_Order.z                                                          
Drive.z                                                               
ASRS_A.z                                                              
ASRS_B.z                                                              
Diagnosis.z                                                           
COHS.z                                                                
FeedbackCondNoFeedback.z                                              
CongruencyIncongruent.z                                               
FeedbackCondNoFeedback.z:CongruencyIncongruent.z                      
ASRS_A.z:FeedbackCondNoFeedback.z                                     
ASRS_A.z:CongruencyIncongruent.z                                      
ASRS_B.z:FeedbackCondNoFeedback.z                             0.000   
ASRS_B.z:CongruencyIncongruent.z                             -0.594   
Diagnosis.z:FeedbackCondNoFeedback.z                          0.000   
Diagnosis.z:CongruencyIncongruent.z                          -0.143   
COHS.z:FeedbackCondNoFeedback.z                               0.000   
COHS.z:CongruencyIncongruent.z                                0.149   
ASRS_A.z:FeedbackCondNoFeedback.z:CongruencyIncongruent.z     0.000   
ASRS_B.z:FeedbackCondNoFeedback.z:CongruencyIncongruent.z     0.000   
Diagnosis.z:FeedbackCondNoFeedback.z:CongruencyIncongruent.z  0.000   
COHS.z:FeedbackCondNoFeedback.z:CongruencyIncongruent.z       0.000   
                                                             ASRS_B.z:FCNF.
Gender.z                                                                   
Cong_Order.z                                                               
Drive.z                                                                    
ASRS_A.z                                                                   
ASRS_B.z                                                                   
Diagnosis.z                                                                
COHS.z                                                                     
FeedbackCondNoFeedback.z                                                   
CongruencyIncongruent.z                                                    
FeedbackCondNoFeedback.z:CongruencyIncongruent.z                           
ASRS_A.z:FeedbackCondNoFeedback.z                                          
ASRS_A.z:CongruencyIncongruent.z                                           
ASRS_B.z:FeedbackCondNoFeedback.z                                          
ASRS_B.z:CongruencyIncongruent.z                              0.000        
Diagnosis.z:FeedbackCondNoFeedback.z                          0.186        
Diagnosis.z:CongruencyIncongruent.z                           0.000        
COHS.z:FeedbackCondNoFeedback.z                              -0.202        
COHS.z:CongruencyIncongruent.z                                0.000        
ASRS_A.z:FeedbackCondNoFeedback.z:CongruencyIncongruent.z     0.000        
ASRS_B.z:FeedbackCondNoFeedback.z:CongruencyIncongruent.z     0.000        
Diagnosis.z:FeedbackCondNoFeedback.z:CongruencyIncongruent.z  0.000        
COHS.z:FeedbackCondNoFeedback.z:CongruencyIncongruent.z       0.000        
                                                             ASRS_B.:C
Gender.z                                                              
Cong_Order.z                                                          
Drive.z                                                               
ASRS_A.z                                                              
ASRS_B.z                                                              
Diagnosis.z                                                           
COHS.z                                                                
FeedbackCondNoFeedback.z                                              
CongruencyIncongruent.z                                               
FeedbackCondNoFeedback.z:CongruencyIncongruent.z                      
ASRS_A.z:FeedbackCondNoFeedback.z                                     
ASRS_A.z:CongruencyIncongruent.z                                      
ASRS_B.z:FeedbackCondNoFeedback.z                                     
ASRS_B.z:CongruencyIncongruent.z                                      
Diagnosis.z:FeedbackCondNoFeedback.z                          0.000   
Diagnosis.z:CongruencyIncongruent.z                           0.186   
COHS.z:FeedbackCondNoFeedback.z                               0.000   
COHS.z:CongruencyIncongruent.z                               -0.202   
ASRS_A.z:FeedbackCondNoFeedback.z:CongruencyIncongruent.z     0.000   
ASRS_B.z:FeedbackCondNoFeedback.z:CongruencyIncongruent.z     0.000   
Diagnosis.z:FeedbackCondNoFeedback.z:CongruencyIncongruent.z  0.000   
COHS.z:FeedbackCondNoFeedback.z:CongruencyIncongruent.z       0.000   
                                                             Dg.:FCNF. D.:CI.
Gender.z                                                                     
Cong_Order.z                                                                 
Drive.z                                                                      
ASRS_A.z                                                                     
ASRS_B.z                                                                     
Diagnosis.z                                                                  
COHS.z                                                                       
FeedbackCondNoFeedback.z                                                     
CongruencyIncongruent.z                                                      
FeedbackCondNoFeedback.z:CongruencyIncongruent.z                             
ASRS_A.z:FeedbackCondNoFeedback.z                                            
ASRS_A.z:CongruencyIncongruent.z                                             
ASRS_B.z:FeedbackCondNoFeedback.z                                            
ASRS_B.z:CongruencyIncongruent.z                                             
Diagnosis.z:FeedbackCondNoFeedback.z                                         
Diagnosis.z:CongruencyIncongruent.z                           0.000          
COHS.z:FeedbackCondNoFeedback.z                               0.054     0.000
COHS.z:CongruencyIncongruent.z                                0.000     0.054
ASRS_A.z:FeedbackCondNoFeedback.z:CongruencyIncongruent.z     0.000     0.000
ASRS_B.z:FeedbackCondNoFeedback.z:CongruencyIncongruent.z     0.000     0.000
Diagnosis.z:FeedbackCondNoFeedback.z:CongruencyIncongruent.z  0.000     0.000
COHS.z:FeedbackCondNoFeedback.z:CongruencyIncongruent.z       0.000     0.000
                                                             COHS.z:FCNF.
Gender.z                                                                 
Cong_Order.z                                                             
Drive.z                                                                  
ASRS_A.z                                                                 
ASRS_B.z                                                                 
Diagnosis.z                                                              
COHS.z                                                                   
FeedbackCondNoFeedback.z                                                 
CongruencyIncongruent.z                                                  
FeedbackCondNoFeedback.z:CongruencyIncongruent.z                         
ASRS_A.z:FeedbackCondNoFeedback.z                                        
ASRS_A.z:CongruencyIncongruent.z                                         
ASRS_B.z:FeedbackCondNoFeedback.z                                        
ASRS_B.z:CongruencyIncongruent.z                                         
Diagnosis.z:FeedbackCondNoFeedback.z                                     
Diagnosis.z:CongruencyIncongruent.z                                      
COHS.z:FeedbackCondNoFeedback.z                                          
COHS.z:CongruencyIncongruent.z                                0.000      
ASRS_A.z:FeedbackCondNoFeedback.z:CongruencyIncongruent.z     0.000      
ASRS_B.z:FeedbackCondNoFeedback.z:CongruencyIncongruent.z     0.000      
Diagnosis.z:FeedbackCondNoFeedback.z:CongruencyIncongruent.z  0.000      
COHS.z:FeedbackCondNoFeedback.z:CongruencyIncongruent.z       0.000      
                                                             COHS.:C
Gender.z                                                            
Cong_Order.z                                                        
Drive.z                                                             
ASRS_A.z                                                            
ASRS_B.z                                                            
Diagnosis.z                                                         
COHS.z                                                              
FeedbackCondNoFeedback.z                                            
CongruencyIncongruent.z                                             
FeedbackCondNoFeedback.z:CongruencyIncongruent.z                    
ASRS_A.z:FeedbackCondNoFeedback.z                                   
ASRS_A.z:CongruencyIncongruent.z                                    
ASRS_B.z:FeedbackCondNoFeedback.z                                   
ASRS_B.z:CongruencyIncongruent.z                                    
Diagnosis.z:FeedbackCondNoFeedback.z                                
Diagnosis.z:CongruencyIncongruent.z                                 
COHS.z:FeedbackCondNoFeedback.z                                     
COHS.z:CongruencyIncongruent.z                                      
ASRS_A.z:FeedbackCondNoFeedback.z:CongruencyIncongruent.z     0.000 
ASRS_B.z:FeedbackCondNoFeedback.z:CongruencyIncongruent.z     0.000 
Diagnosis.z:FeedbackCondNoFeedback.z:CongruencyIncongruent.z  0.000 
COHS.z:FeedbackCondNoFeedback.z:CongruencyIncongruent.z       0.000 
                                                             ASRS_A.:FCNF.:
Gender.z                                                                   
Cong_Order.z                                                               
Drive.z                                                                    
ASRS_A.z                                                                   
ASRS_B.z                                                                   
Diagnosis.z                                                                
COHS.z                                                                     
FeedbackCondNoFeedback.z                                                   
CongruencyIncongruent.z                                                    
FeedbackCondNoFeedback.z:CongruencyIncongruent.z                           
ASRS_A.z:FeedbackCondNoFeedback.z                                          
ASRS_A.z:CongruencyIncongruent.z                                           
ASRS_B.z:FeedbackCondNoFeedback.z                                          
ASRS_B.z:CongruencyIncongruent.z                                           
Diagnosis.z:FeedbackCondNoFeedback.z                                       
Diagnosis.z:CongruencyIncongruent.z                                        
COHS.z:FeedbackCondNoFeedback.z                                            
COHS.z:CongruencyIncongruent.z                                             
ASRS_A.z:FeedbackCondNoFeedback.z:CongruencyIncongruent.z                  
ASRS_B.z:FeedbackCondNoFeedback.z:CongruencyIncongruent.z    -0.594        
Diagnosis.z:FeedbackCondNoFeedback.z:CongruencyIncongruent.z -0.143        
COHS.z:FeedbackCondNoFeedback.z:CongruencyIncongruent.z       0.149        
                                                             ASRS_B.:FCNF.:
Gender.z                                                                   
Cong_Order.z                                                               
Drive.z                                                                    
ASRS_A.z                                                                   
ASRS_B.z                                                                   
Diagnosis.z                                                                
COHS.z                                                                     
FeedbackCondNoFeedback.z                                                   
CongruencyIncongruent.z                                                    
FeedbackCondNoFeedback.z:CongruencyIncongruent.z                           
ASRS_A.z:FeedbackCondNoFeedback.z                                          
ASRS_A.z:CongruencyIncongruent.z                                           
ASRS_B.z:FeedbackCondNoFeedback.z                                          
ASRS_B.z:CongruencyIncongruent.z                                           
Diagnosis.z:FeedbackCondNoFeedback.z                                       
Diagnosis.z:CongruencyIncongruent.z                                        
COHS.z:FeedbackCondNoFeedback.z                                            
COHS.z:CongruencyIncongruent.z                                             
ASRS_A.z:FeedbackCondNoFeedback.z:CongruencyIncongruent.z                  
ASRS_B.z:FeedbackCondNoFeedback.z:CongruencyIncongruent.z                  
Diagnosis.z:FeedbackCondNoFeedback.z:CongruencyIncongruent.z  0.186        
COHS.z:FeedbackCondNoFeedback.z:CongruencyIncongruent.z      -0.202        
                                                             D.:FCNF.:
Gender.z                                                              
Cong_Order.z                                                          
Drive.z                                                               
ASRS_A.z                                                              
ASRS_B.z                                                              
Diagnosis.z                                                           
COHS.z                                                                
FeedbackCondNoFeedback.z                                              
CongruencyIncongruent.z                                               
FeedbackCondNoFeedback.z:CongruencyIncongruent.z                      
ASRS_A.z:FeedbackCondNoFeedback.z                                     
ASRS_A.z:CongruencyIncongruent.z                                      
ASRS_B.z:FeedbackCondNoFeedback.z                                     
ASRS_B.z:CongruencyIncongruent.z                                      
Diagnosis.z:FeedbackCondNoFeedback.z                                  
Diagnosis.z:CongruencyIncongruent.z                                   
COHS.z:FeedbackCondNoFeedback.z                                       
COHS.z:CongruencyIncongruent.z                                        
ASRS_A.z:FeedbackCondNoFeedback.z:CongruencyIncongruent.z             
ASRS_B.z:FeedbackCondNoFeedback.z:CongruencyIncongruent.z             
Diagnosis.z:FeedbackCondNoFeedback.z:CongruencyIncongruent.z          
COHS.z:FeedbackCondNoFeedback.z:CongruencyIncongruent.z       0.054   

Standardized Within-Group Residuals:
        Min          Q1         Med          Q3         Max 
-2.43316782 -0.59970244  0.06578658  0.62969155  2.41758883 

Number of Observations: 416
Number of Groups: 104
```

In [35]:

```
#Extract the R^2 value of each model
r.squaredGLMM(dprime_fb_model1_r)
r.squaredGLMM(dprime_fb_model2_r)
r.squaredGLMM(dprime_fb_model3_r)
r.squaredGLMM(dprime_fb_model4_r)
```

| R2m | R2c |
| --- | --- |
| 0.02250472 | 0.533829 |

| R2m | R2c |
| --- | --- |
| 0.04098239 | 0.5338497 |

| R2m | R2c |
| --- | --- |
| 0.07733334 | 0.5822466 |

| R2m | R2c |
| --- | --- |
| 0.08681478 | 0.59487 |

In [36]:

```
#Subtract from each other to derive delta R^2. First will be 2-1, next 3-2. 
r.squaredGLMM(dprime_fb_model2_r) - r.squaredGLMM(dprime_fb_model1_r)
r.squaredGLMM(dprime_fb_model3_r) - r.squaredGLMM(dprime_fb_model2_r)
r.squaredGLMM(dprime_fb_model4_r) - r.squaredGLMM(dprime_fb_model3_r)
```

| R2m | R2c |
| --- | --- |
| 0.01847766 | 2.070854e-05 |

| R2m | R2c |
| --- | --- |
| 0.03635095 | 0.04839692 |

| R2m | R2c |
| --- | --- |
| 0.009481447 | 0.01262341 |

In [37]:

```
#Compare the models to each other to extract log likelihood ratio Chi^2 values and the associated p-values. 
#Df is however many new variables are added to next model.
anova(dprime_fb_model1_r, dprime_fb_model2_r, dprime_fb_model3_r, dprime_fb_model4_r)
```

|  | call | Model | df | AIC | BIC | logLik | Test | L.Ratio | p-value |
| --- | --- | --- | --- | --- | --- | --- | --- | --- | --- |
| dprime\_fb\_model1\_r | lme.formula(fixed = dprime ~ Gender + Cong\_Order + Drive, data = subset(df\_dprime\_long\_mm, StimulusType == "Familiar"), random = ~1 | Subject, method = "ML") | 1 | 6 | -114.6162 | -90.43208 | 63.30810 |  | NA | NA |
| dprime\_fb\_model2\_r | lme.formula(fixed = dprime ~ Gender + Cong\_Order + Drive + ASRS\_A + ASRS\_B + Diagnosis + COHS, data = subset(df\_dprime\_long\_mm, StimulusType == "Familiar"), random = ~1 | Subject, method = "ML") | 2 | 10 | -109.7155 | -69.40866 | 64.85775 | 1 vs 2 | 3.099313 | 5.413453e-01 |
| dprime\_fb\_model3\_r | lme.formula(fixed = dprime ~ Gender + Cong\_Order + Drive + ASRS\_A + ASRS\_B + Diagnosis + COHS + FeedbackCond + Congruency + FeedbackCond \* Congruency, data = subset(df\_dprime\_long\_mm, StimulusType == "Familiar"), random = ~1 | Subject, method = "ML") | 3 | 13 | -137.8887 | -85.48980 | 81.94435 | 2 vs 3 | 34.173195 | 1.821212e-07 |
| dprime\_fb\_model4\_r | lme.formula(fixed = dprime ~ Gender + Cong\_Order + Drive + ASRS\_A + ASRS\_B + Diagnosis + COHS + FeedbackCond + Congruency + FeedbackCond \* Congruency + ASRS\_A \* FeedbackCond \* Congruency + ASRS\_B \* FeedbackCond \* Congruency + Diagnosis \* FeedbackCond \* Congruency + COHS \* FeedbackCond \* Congruency, data = subset(df\_dprime\_long\_mm, StimulusType == "Familiar"), random = ~1 | Subject, method = "ML") | 4 | 25 | -123.4548 | -22.68766 | 86.72739 | 3 vs 4 | 9.566084 | 6.539687e-01 |
