# Supplementary material for: Habit Expression and Disruption as a Function of Attention-Deficit/Hyperactivity Disorder Symptomology
Source: Front Psychol. 2019 Sep 3;10:1997. doi: 10.3389/fpsyg.2019.01997 (PMC6733985; doi:10.3389/fpsyg.2019.01997)
Supplement: Supplementary file 1 [file Data_Sheet_1.ZIP › Signal_detection_plots.html]

Signal detection plots


## Signal detection analyses: Plotting notebook¶

In [1]:

```
library(ggplot2)
#read long dprime data from python csv
df_dprime_long <- read.csv("dprime_long.csv")
head(df_dprime_long)
```

| X | Subject | StimulusType | Congruency | FeedbackCond | DV | dprime |
| --- | --- | --- | --- | --- | --- | --- |
| 0 | 1 | Familiar | Congruent | NoFeedback | FamCongDay1\_dprime | 0.5278942 |
| 1 | 2 | Familiar | Congruent | NoFeedback | FamCongDay1\_dprime | 0.8657486 |
| 2 | 3 | Familiar | Congruent | NoFeedback | FamCongDay1\_dprime | 0.5176180 |
| 3 | 4 | Familiar | Congruent | NoFeedback | FamCongDay1\_dprime | 0.6389834 |
| 4 | 5 | Familiar | Congruent | NoFeedback | FamCongDay1\_dprime | 0.8657486 |
| 5 | 6 | Familiar | Congruent | NoFeedback | FamCongDay1\_dprime | 0.5939314 |

In [2]:

```
data_summary <- function(data, varname, groupnames){
  require(plyr)
  summary_func <- function(x, col){
    c(mean = mean(x[[col]], na.rm=TRUE),
      sd = sd(x[[col]], na.rm=TRUE))
  }
  data_sum<-ddply(data, groupnames, .fun=summary_func,
                  varname)
  data_sum <- rename(data_sum, c("mean" = varname))
 return(data_sum)
}
```

In [3]:

```
df_dprime_long_se <- data_summary(subset(df_dprime_long, StimulusType=="Familiar"), varname="dprime", 
                    groupnames=c("FeedbackCond", "Congruency"))
df_dprime_long_se$FeedbackCond=as.factor(df_dprime_long_se$FeedbackCond)
df_dprime_long_se$Congruency=as.factor(df_dprime_long_se$Congruency)
df_dprime_long_se["se"]=df_dprime_long_se["sd"]/sqrt(104)
head(df_dprime_long_se)
```

```
Loading required package: plyr
```

| FeedbackCond | Congruency | dprime | sd | se |
| --- | --- | --- | --- | --- |
| Feedback | Congruent | 0.6536956 | 0.2418145 | 0.02371187 |
| Feedback | Incongruent | 0.6666031 | 0.2595149 | 0.02544753 |
| NoFeedback | Congruent | 0.6829062 | 0.2222728 | 0.02179564 |
| NoFeedback | Incongruent | 0.5619444 | 0.2478688 | 0.02430554 |

In [4]:

```
options(repr.plot.width=4, repr.plot.height=3.5)
pfb <- ggplot(df_dprime_long_se, aes(x=FeedbackCond, y=dprime, fill=Congruency)) + scale_fill_manual(values=c("grey55", "grey4", "grey1", "grey0")) + 
geom_bar(stat="identity", position=position_dodge()) + 
geom_errorbar(aes(ymin=dprime-se, ymax=dprime+se), width=.2, position=position_dodge(.9)) +
ggtitle("Signal Detection Analysis:\nFeedback vs. No-Feedback") + ylab("Sensitivity (d')") + 
theme_update(plot.title = element_text(hjust = 0.5)) +
theme(legend.position = "none", panel.grid.major = element_blank(), panel.grid.minor = element_blank(), 
      panel.border = element_blank(), axis.line = element_line(color = 'black'), axis.text.x = element_text(face="bold", 
                           size=13), axis.title.y = element_text(face="bold", size=13), axis.text.y = element_text(face="bold", size=11), plot.title = element_text(size=16, face="bold"))
pfb + theme(axis.title.x = element_blank(), plot.background = element_blank(), panel.background = element_rect(fill = 'white')) + 
annotate(geom="text", size=3.4, fontface="bold", color="white", x=0.77, y=0.05, label="GR/RED") +
annotate(geom="text", size=3.4, fontface="bold", color="white", x=1.22, y=0.05, label="RED/GR") +
annotate(geom="text", size=3.4, fontface="bold", color="white", x=1.77, y=0.05, label="PR/BL") +
annotate(geom="text", size=3.4, fontface="bold", color="white", x=2.22, y=0.05, label="BL/PR")
```

In [5]:

```
df_dprime_long_se2 <- data_summary(subset(df_dprime_long, FeedbackCond=="NoFeedback"), varname="dprime", 
                    groupnames=c("StimulusType", "Congruency"))
df_dprime_long_se2$StimulusType=as.factor(df_dprime_long_se2$StimulusType)
df_dprime_long_se2$Congruency=as.factor(df_dprime_long_se2$Congruency)
df_dprime_long_se2["se"]=df_dprime_long_se2["sd"]/sqrt(104)
head(df_dprime_long_se2)
```

| StimulusType | Congruency | dprime | sd | se |
| --- | --- | --- | --- | --- |
| Familiar | Congruent | 0.6829062 | 0.2222728 | 0.02179564 |
| Familiar | Incongruent | 0.5619444 | 0.2478688 | 0.02430554 |
| Novel | Congruent | 0.5998626 | 0.2244009 | 0.02200431 |
| Novel | Incongruent | 0.6276581 | 0.2216999 | 0.02173946 |

In [6]:

```
p <- ggplot(df_dprime_long_se2, aes(x=StimulusType, y=dprime, fill=Congruency)) + scale_fill_manual(values=c("grey55", "grey4", "grey1", "grey0")) + 
geom_bar(stat="identity", position=position_dodge()) + 
geom_errorbar(aes(ymin=dprime-se, ymax=dprime+se), width=.2, position=position_dodge(.9)) +
ggtitle("Signal Detection Analysis:\nFamiliar and Novel Stimuli") + ylab("Sensitivity (d')") + 
theme_update(plot.title = element_text(hjust = 0.5)) +
theme(legend.position = "none", panel.grid.major = element_blank(), panel.grid.minor = element_blank(), 
      panel.border = element_blank(), axis.line = element_line(color = 'black'), axis.text.x = element_text(face="bold", 
                           size=13), axis.title.y = element_text(face="bold", size=13), axis.text.y = element_text(face="bold", size=11), plot.title = element_text(size=16, face="bold"))
p + theme(axis.title.x = element_blank(), plot.background = element_blank(), panel.background = element_rect(fill = 'white')) + 
annotate(geom="text", size=3.4, fontface="bold", color="white", x=0.77, y=0.05, label="GR/RED") +
annotate(geom="text", size=3.4, fontface="bold", color="white", x=1.22, y=0.05, label="RED/GR") +
annotate(geom="text", size=3.4, fontface="bold", color="white", x=1.77, y=0.05, label="PR/BL") +
annotate(geom="text", size=3.4, fontface="bold", color="white", x=2.22, y=0.05, label="BL/PR")
```

In [ ]:

```

```
