# Supplementary material for: Habit Expression and Disruption as a Function of Attention-Deficit/Hyperactivity Disorder Symptomology
Source: Front Psychol. 2019 Sep 3;10:1997. doi: 10.3389/fpsyg.2019.01997 (PMC6733985; doi:10.3389/fpsyg.2019.01997)
Supplement: Supplementary file 2 [file Table_1.docx]

**Habit Expression and Disruption as a Function of Attention-Deficit/Hyperactivity Disorder Symptomology**

**Ahmet O. Ceceli^1*^, Giavanna Esposito^2^, & Elizabeth Tricomi^1^**

^1^Department of Psychology, Rutgers University-Newark, Newark, NJ, United States of America

^2^New Jersey Institute of Technology, Newark, NJ, United States of America

**Supplemental Materials**

Our pre-registered hierarchical mixed models did not meet multicollinearity assumptions due to high correlations between the following regressors: Age and Driving; Phase_Order and Stim_Familiarity_Order; ASRS_Total and ASRS_Inattentive/Hyperactive. We removed the redundant regressors from our analyses (i.e., Age, Stim_Familiarity_Order, and ASRS_Total) that were not integral for our hypotheses to meet the assumption of non-multicollinearity, and included these corrected analyses in the main text. We report below the complete, multicollinear set of regressors for consistency with the pre-registration document. Because of the potentially inflated regression coefficients due to multicollinearity, we refrain from speculating on significant effects in these analyses, and refer readers to the corrected models included in the main text.

Supplemental Table 1. Hierarchical Mixed Model of ADHD Symptomology and Habit Expression: ΔNoGo_Accuracy (As Pre-registered).

| Variable | *VIF* | *β* | *t* | *sig.* |
| --- | --- | --- | --- | --- |
| **Model 1** |  |  |  |  |
| Age | 6.77 | -.25 (.18) | -1.42 | .160 |
| Gender | 1.10 | -.11 (.07) | -1.49 | .140 |
| Phase_Order | 3.98 | .17 (.14) | 1.27 | .206 |
| Stim_Familiarity_Order | 4.11 | -.21 (.14) | -1.53 | .130 |
| Driving | 6.71 | .32 (.18) | 1.79 | .077 |
| **Model 2** |  |  |  |  |
| Age | 7.21 | -.27 (.19) | -1.46 | .167 |
| Gender | **1.21** | -.09 (.08) | -1.24 | .220 |
| Phase_Order | 4.21 | .17 (.14) | 1.24 | .218 |
| Stim_Familiarity_Order | 4.36 | -.22 (.14) | -1.52 | .131 |
| Driving | 6.99 | .33 (.18) | 1.79 | .077 |
| ASRS_Inattentive | 9.00 | -.05 (.21) | -0.23 | .822 |
| ASRS_Hyperactive | 8.74 | .02 (.20) | 0.08 | .934 |
| ASRS_Total | 23.53 | .03 (.34) | 0.10 | .917 |
| Diagnosis | 1.33 | .03 (.08) | 0.40 | .692 |
| COHS | 1.08 | -.06 (.07) | -0.77 | .442 |
| **Model 3** |  |  |  |  |
| Age | 7.21 | -.27 (.18) | -1.54 | .128 |
| Gender | **1.21** | -.09 (.07) | -1.30 | .197 |
| Phase_Order | 4.21 | .17 (.13) | 1.30 | .196 |
| Stim_Familiarity_Order | 4.36 | -.22 (.14) | -1.60 | .113 |
| Driving | 6.99 | .33 (.17) | 1.88 | .063 |
| ASRS_Inattentive | 9.00 | -.05 (.20) | -0.24 | .813 |
| ASRS_Hyperactive | 8.74 | .02 (.19) | 0.09 | .931 |
| ASRS_Total | 23.53 | .03 (.32) | 0.11 | .913 |
| Diagnosis | 1.33 | .03 (.08) | 0.42 | .677 |
| COHS | 1.08 | -.06 (.07) | -0.81 | .419 |
| Stim_Familiarity | **1** | **.31 (.07)** | **4.68** | **<.001** |
| **Model 4** |  |  |  |  |
| Age | 7.21 | -.27 (.18) | -1.55 | .126 |
| Gender | **1.21** | -.09 (.07) | -1.31 | .194 |
| Phase_Order | 4.21 | .18 (.13) | 1.31 | .193 |
| Stim_Familiarity_Order | 4.36 | -.22 (.14) | -1.61 | .111 |
| Driving | 6.99 | .33 (.17) | 1.89 | .062 |
| ASRS_Inattentive | 17.56 | -.05 (.20) | -0.24 | .812 |
| ASRS_Hyperactive | 17.22 | .02 (.19) | 0.09 | .931 |
| ASRS_Total | 46.31 | .03 (.32) | 0.11 | .912 |
| Diagnosis | 2.38 | .03 (.08) | 0.42 | .675 |
| COHS | 2.13 | -.06 (.07) | -0.82 | .417 |
| Stim_Familiarity | **64.97** | **.31 (.07)** | **4.70** | **<.001** |
| ASRS_Inattentive x Stim_Familiarity | 91.11 | .17 (.19) | 0.89 | .376 |
| ASRS_Hyperactive x Stim_Familiarity | 71.66 | .15 (.19) | 0.76 | .448 |
| ASRS_Total x Stim_Familiarity | 265.78 | -.35(.31) | -1.12 | .265 |
| Diagnosis x Stim_Familiarity | 2.16 | .10 (.07) | 1.54 | .126 |
| COHS x Stim_Familiarity | 57.37 | .12 (.07) | 1.74 | .085 |

| Model | *R^2^* | *Log likel.* | χ^2^ | χ^2^ *sig.* | *ΔR^2^* |
| --- | --- | --- | --- | --- | --- |
| Model 1 | .05 | 81.55 |  |  |  |
| Model 2 | .05 | 81.99 | 0.89 | .971 | <.01 |
| Model 3 | **.15** | **92.99** | **22.00** | **<.001** | **.10** |
| Model 4 | .18 | 96.83 | 7.69 | .174 | .03 |

**Model Comparisons**

*Note: Top layer of table depicts all regressors included in the hierarchical model. Standard errors are given in parentheses. Bottom layer of table, Model Comparisons, depicts the predictive strength of each model, as compared to its previous step. VIF = Variance Inflation Factor. Log likel. = Log likelihood. Significant p-values depicted in bold typeface.*

Supplemental Table 2. Hierarchical Mixed Model of ADHD Symptomology and Habit Disruption: ΔNoGo_Accuracy (As Pre-registered).

| Variable | *VIF* | *β* | *t* | *sig.* |
| --- | --- | --- | --- | --- |
| **Model 1** |  |  |  |  |
| Age | 6.77 | .29 (.18) | 1.61 | .111 |
| Gender | 1.10 | .04 (.07) | 0.60 | .550 |
| **Phase_Order** | **3.98** | **.30 (.14)** | **2.20** | **.030** |
| Stim_Familiarity_Order | 4.11 | -.23 (.14) | -1.67 | .098 |
| Driving | 6.71 | -.28 (.18) | -1.54 | .126 |
| **Model 2** |  |  |  |  |
| Age | 7.21 | .32 (.18) | 1.72 | .088 |
| Gender | 1.21 | .05 (.08) | 0.62 | .537 |
| Phase_Order | **4.21** | **.30 (.14)** | **2.14** | **.035** |
| Stim_Familiarity_Order | 4.36 | -.25 (.14) | -1.71 | .091 |
| Driving | 6.99 | -.26 (.18) | -1.45 | .151 |
| ASRS_Inattentive | 9.00 | .05 (.21) | 0.24 | .809 |
| ASRS_Hyperactive | 8.74 | .28 (.20) | 1.37 | .174 |
| ASRS_Total | 23.53 | -.27 (.33) | -0.80 | .423 |
| Diagnosis | 1.33 | -.04 (.08) | -0.53 | .595 |
| COHS | 1.08 | -.08 (.07) | -1.18 | .239 |
| **Model 3** |  |  |  |  |
| Age | 7.21 | .32 (.18) | 1.80 | .076 |
| Gender | 1.21 | .05 (.07) | 0.65 | .520 |
| Phase_Order | **4.21** | **.30 (.14)** | **2.23** | **.028** |
| Stim_Familiarity_Order | 4.36 | -.25 (.14) | -1.78 | .078 |
| Driving | 6.99 | -.26 (.17) | -1.51 | .135 |
| ASRS_Inattentive | 9.00 | .05 (.20) | 0.25 | .801 |
| ASRS_Hyperactive | 8.74 | .28 (.20) | 1.43 | .157 |
| ASRS_Total | 23.53 | -.27 (.32) | -0.84 | .404 |
| Diagnosis | 1.33 | -.04 (.08) | -0.55 | .580 |
| COHS | 1.08 | -.08 (.07) | -1.23 | .221 |
| Feedback | **1** | **-.28 (.07)** | **-4.18** | **<.001** |
| **Model 4** |  |  |  |  |
| Age | 7.21 | .32 (.18) | 1.79 | .076 |
| Gender | 1.21 | .05 (.07) | 0.64 | .521 |
| Phase_Order | **4.21** | **.30 (.14)** | **2.22** | **.029** |
| Stim_Familiarity_Order | 4.36 | -.25 (.14) | -1.78 | .079 |
| Driving | 6.99 | -.26 (.18) | -1.50 | .136 |
| ASRS_Inattentive | 17.56 | .05 (.20) | 0.25 | .802 |
| ASRS_Hyperactive | 17.22 | .28 (.20) | 1.42 | .158 |
| ASRS_Total | 46.31 | -.27 (.32) | -0.84 | .405 |
| Diagnosis | 2.38 | -.04 (.08) | -0.55 | .581 |
| COHS | 2.13 | -.08 (.07) | -1.23 | .222 |
| Feedback | **64.97** | **-.28 (.07)** | **-4.17** | **<.001** |
| ASRS_Inattentive x Feedback | 91.11 | -.23 (.19) | -1.20 | .232 |
| ASRS_Hyperactive x Feedback | 71.66 | -.30 (.19) | -1.53 | .128 |
| ASRS_Total x Feedback | 265.78 | .52 (.32) | 1.62 | .108 |
| Diagnosis x Feedback | 2.16 | .01 (.07) | 0.21 | .837 |
| COHS x Feedback | 57.37 | -.06 (.07) | -0.87 | .388 |

| Model | *R^2^* | *Log likel.* | χ^2^ | χ^2^ *sig.* | *ΔR^2^* |
| --- | --- | --- | --- | --- | --- |
| Model 1 | .04 | 75.55 |  |  |  |
| Model 2 | .06 | 77.72 | 4.35 | .501 | .02 |
| Model 3 | **.14** | **86.58** | **17.73** | **<.001** | **.08** |
| Model 4 | .16 | 88.82 | 4.47 | .484 | .02 |

**Model Comparisons**

*Note: Top layer of table depicts all regressors included in the hierarchical model. Standard errors are given in parentheses. Bottom layer of table, Model Comparisons, depicts the predictive strength of each model, as compared to its previous step. VIF = Variance Inflation Factor. Log likel. = Log likelihood. Significant p-values depicted in bold typeface.*

Supplemental Table 3. Hierarchical Mixed Model of ADHD Symptomology and Habit Expression: ΔGo_Accuracy (As Pre-registered).

| Variable | *VIF* | *β* | *t* | *sig.* |
| --- | --- | --- | --- | --- |
| **Model 1** |  |  |  |  |
| Age | 6.77 | -.07 (.18) | -0.41 | .686 |
| Gender | 1.10 | .03 (.07) | 0.39 | .693 |
| Phase_Order | 3.98 | .04 (.14) | 0.31 | .756 |
| Stim_Familiarity_Order | 4.11 | -.01 (.14) | -0.07 | .941 |
| Driving | 6.71 | .12 (.18) | 0.66 | .512 |
| **Model 2** |  |  |  |  |
| Age | 7.21 | -.19 (.19) | -1.04 | .301 |
| Gender | 1.21 | .08 (.08) | 1.06 | .291 |
| Phase_Order | 4.21 | .05 (.14) | 0.39 | .697 |
| Stim_Familiarity_Order | 4.36 | -.04 (.14) | -0.29 | .773 |
| Driving | 6.99 | .15 (.18) | 0.81 | .421 |
| ASRS_Inattentive | **9.00** | **-.47 (.21)** | **-2.25** | **.027** |
| ASRS_Hyperactive | **8.74** | **-.48 (.20)** | **-2.37** | **.020** |
| ASRS_Total | **23.53** | **.78 (.33)** | **2.33** | **.022** |
| Diagnosis | 1.33 | .15 (.08) | 1.85 | .067 |
| COHS | 1.08 | -.06 (.07) | -0.85 | .397 |
| **Model 3** |  |  |  |  |
| Age | 7.21 | -.19 (.18) | -1.05 | .294 |
| Gender | 1.21 | .08 (.07) | 1.08 | .284 |
| Phase_Order | 4.21 | .05 (.14) | 0.40 | .692 |
| Stim_Familiarity_Order | 4.36 | -.04 (.14) | -0.29 | .770 |
| Driving | 6.99 | .15 (.18) | 0.82 | .414 |
| ASRS_Inattentive | **9.00** | **-.47 (.20)** | **-2.28** | **.025** |
| ASRS_Hyperactive | **8.74** | **-.48 (.20)** | **-2.41** | **.018** |
| ASRS_Total | **23.53** | **.78 (.33)** | **2.37** | **.020** |
| Diagnosis | 1.33 | .15 (.08) | 1.88 | .063 |
| COHS | 1.08 | -.06 (.07) | -0.86 | .390 |
| Stim_Familiarity | **1** | **.18 (.07)** | **2.64** | **<.009** |
| **Model 4** |  |  |  |  |
| Age | 7.21 | -.19 (.18) | -1.06 | .290 |
| Gender | **1.21** | .08 (.07) | 1.08 | .281 |
| Phase_Order | 4.21 | .05 (.14) | 0.40 | .690 |
| Stim_Familiarity_Order | 4.36 | -.04 (.14) | -0.30 | .768 |
| Driving | 6.99 | .15 (.18) | 0.83 | .410 |
| ASRS_Inattentive | **17.56** | **-.47 (.20)** | **-2.29** | **.024** |
| ASRS_Hyperactive | **17.22** | **-.48 (.20)** | **-2.42** | **.017** |
| ASRS_Total | **46.31** | **.78 (.33)** | **2.38** | **.019** |
| Diagnosis | 2.38 | .15 (.08) | 1.89 | .061 |
| COHS | 2.13 | -.06 (.07) | -0.87 | .386 |
| Stim_Familiarity | **64.97** | **.18 (.07)** | **2.66** | **<.009** |
| ASRS_Inattentive x Stim_Familiarity | 91.11 | -.22 (.20) | -1.09 | .279 |
| ASRS_Hyperactive x Stim_Familiarity | 71.66 | -.02 (.20) | -0.12 | .906 |
| ASRS_Total x Stim_Familiarity | 265.78 | .14 (.32) | 0.43 | .666 |
| Diagnosis x Stim_Familiarity | **2.16** | **.15 (.07)** | **2.19** | **.031** |
| COHS x Stim_Familiarity | 57.37 | .05 (.07) | 0.77 | .442 |

| Model | *R^2^* | *Log likel.* | χ^2^ | χ^2^ *sig.* | *ΔR^2^* |
| --- | --- | --- | --- | --- | --- |
| Model 1 | .01 | 195.33 |  |  |  |
| Model 2 | **.06** | **201.12** | **11.59** | **.041** | **.05** |
| Model 3 | **.09** | **204.76** | **7.28** | **.007** | **.03** |
| odel 4 | .13 | 208.98 | 8.44 | .133 | .04 |

**Model Comparisons**

*Note: Top layer of table depicts all regressors included in the hierarchical model. Standard errors are given in parentheses. Bottom layer of table, Model Comparisons, depicts the predictive strength of each model, as compared to its previous step. VIF = Variance Inflation Factor. Log likel. = Log likelihood. Significant p-values depicted in bold typeface.*

Supplemental Table 4. Hierarchical Mixed Model of ADHD Symptomology and Habit Disruption: ΔGo_Accuracy (As Pre-registered).

| Variable | *VIF* | *β* | *t* | *sig.* |
| --- | --- | --- | --- | --- |
| **Model 1** |  |  |  |  |
| Age | 6.77 | -.06 (.18) | -0.33 | .737 |
| Gender | 1.10 | .01 (.07) | 0.09 | .928 |
| Phase_Order | **3.98** | **.36 (.14)** | **2.54** | **.012** |
| Stim_Familiarity_Order | 4.11 | -.26 (.14) | -1.83 | .071 |
| Driving | 6.71 | .03 (.18) | 0.18 | .855 |
| **Model 2** |  |  |  |  |
| Age | 7.21 | -.16 (.18) | -0.89 | .377 |
| Gender | 1.21 | .01 (.07) | 0.20 | .845 |
| Phase_Order | **4.21** | **.34 (.14)** | **2.44** | **.016** |
| Stim_Familiarity_Order | 4.36 | -.25 (.14) | -1.73 | .086 |
| Driving | 6.99 | .11 (.18) | 0.60 | .550 |
| ASRS_Inattentive | 9.00 | -.23 (.21) | -1.13 | .261 |
| ASRS_Hyperactive | **8.74** | **-.42 (.20)** | **-2.07** | **.041** |
| ASRS_Total | 23.53 | -.52 (.33) | 1.57 | .120 |
| Diagnosis | 1.33 | -.02 (.08) | -0.30 | .766 |
| COHS | 1.08 | -.08 (.07) | -1.17 | .245 |
| **Model 3** |  |  |  |  |
| Age | 7.21 | -.16 (.18) | -0.89 | .377 |
| Gender | 1.21 | .01 (.08) | 0.20 | .845 |
| Phase_Order | **4.21** | **.34 (.14)** | **2.44** | **.016** |
| Stim_Familiarity_Order | 4.36 | -.25 (.14) | -1.73 | .086 |
| Driving | 6.99 | .11 (.18) | 0.60 | .550 |
| ASRS_Inattentive | 9.00 | -.23 (.21) | -1.13 | .261 |
| ASRS_Hyperactive | **8.74** | **-.42 (.20)** | **-2.07** | **.041** |
| ASRS_Total | 23.53 | -.52 (.33) | 1.57 | .121 |
| Diagnosis | 1.33 | -.02 (.08) | -0.30 | .766 |
| COHS | 1.08 | -.08 (.07) | -1.17 | .245 |
| Feedback | **1** | **-.24 (.06)** | **-3.70** | **<.001** |
| **Model 4** |  |  |  |  |
| Age | 7.21 | -.16 (.19) | -0.88 | .383 |
| Gender | 1.21 | .01 (.08) | 0.19 | .847 |
| Phase_Order | **4.21** | **.34 (.14)** | **2.40** | **.018** |
| Stim_Familiarity_Order | 4.36 | -.25 (.15) | -1.71 | .090 |
| Driving | 6.99 | .11 (.18) | 0.59 | .556 |
| ASRS_Inattentive | 17.56 | -.23 (.21) | -1.12 | .267 |
| ASRS_Hyperactive | **17.22** | **-.42 (.21)** | **-2.04** | **.044** |
| ASRS_Total | 46.31 | -.52 (.34) | 1.55 | .125 |
| Diagnosis | 2.38 | -.02 (.08) | -0.29 | .769 |
| COHS | 2.13 | -.08 (.07) | -1.15 | .251 |
| Feedback | **64.97** | **-.24 (.06)** | **-3.84** | **<.001** |
| ASRS_Inattentive x Feedback | 91.11 | -.12 (.18) | -0.68 | .496 |
| ASRS_Hyperactive x Feedback | 71.66 | -.34 (.18) | -1.86 | .065 |
| ASRS_Total x Feedback | 265.78 | .48 (.30) | 1.62 | .107 |
| Diagnosis x Feedback | 2.16 | <-.01 (.06) | <-0.01 | .996 |
| COHS x Feedback | 57.37 | -.10 (.06) | -1.57 | .120 |

| Model | *R^2^* | *Log likel.* | χ^2^ | χ^2^ *sig.* | *ΔR^2^* |
| --- | --- | --- | --- | --- | --- |
| Model 1 | .04 | 318.23 |  |  |  |
| Model 2 | .07 | 321.76 | 7.06 | .216 | .03 |
| Model 3 | **.13** | **328.57** | **13.63** | **<.001** | **.06** |
| Model 4 | .16 | 333.64 | 10.13 | .072 | .04 |

**Model Comparisons**

*Note: Top layer of table depicts all regressors included in the hierarchical model. Standard errors are given in parentheses. Bottom layer of table, Model Comparisons, depicts the predictive strength of each model, as compared to its previous step. VIF = Variance Inflation Factor. Log likel. = Log likelihood. Significant p-values depicted in bold typeface.*
